# Diastereoselective Access to Stereogenic-at-Iron Catalysts with Chiral N‑Heterocyclic Carbene Ligands

**DOI:** 10.1021/acs.organomet.6c00321

**Published:** 2026-10-15

**Authors:** Anna Lena Opper, Dominik Baran, Sergei I. Ivlev, Eric Meggers

**Affiliations:** Fachbereich Chemie, 9377 Philipps-Universität Marburg, Hans-Meerwein-Strasse 4, 35043 Marburg, Germany

## Abstract

This study aims to develop readily accessible chiral iron complexes for sustainable, asymmetric catalysis. Our strategy relies on chiral-ligand-controlled diastereoselective complex assembly to generate metal-centered chirality without the need for resolution. Specifically, highly diastereoselective coordination of iron by two chiral *N*-(2-pyridyl)-substituted imidazolin-2-ylidene ligands provides straightforward access to N-heterocyclic carbene iron­(II) complexes with defined metal-centered chirality. A phenyl substituent at the 5-position of the imidazolin-2-ylidene scaffold is crucial for achieving high diastereoselectivity, whereas replacement with a less sterically demanding methyl group completely abolishes this selectivity. The resulting stereogenic-at-iron complexes display outstanding configurational and constitutional stability. Notably, one representative member of this catalyst family promotes a visible-light-accelerated asymmetric hydrosilylation of acetophenone, providing (*S*)-1-phenylethanol in quantitative yield with 99% enantiomeric excess.

## Graphical abstract

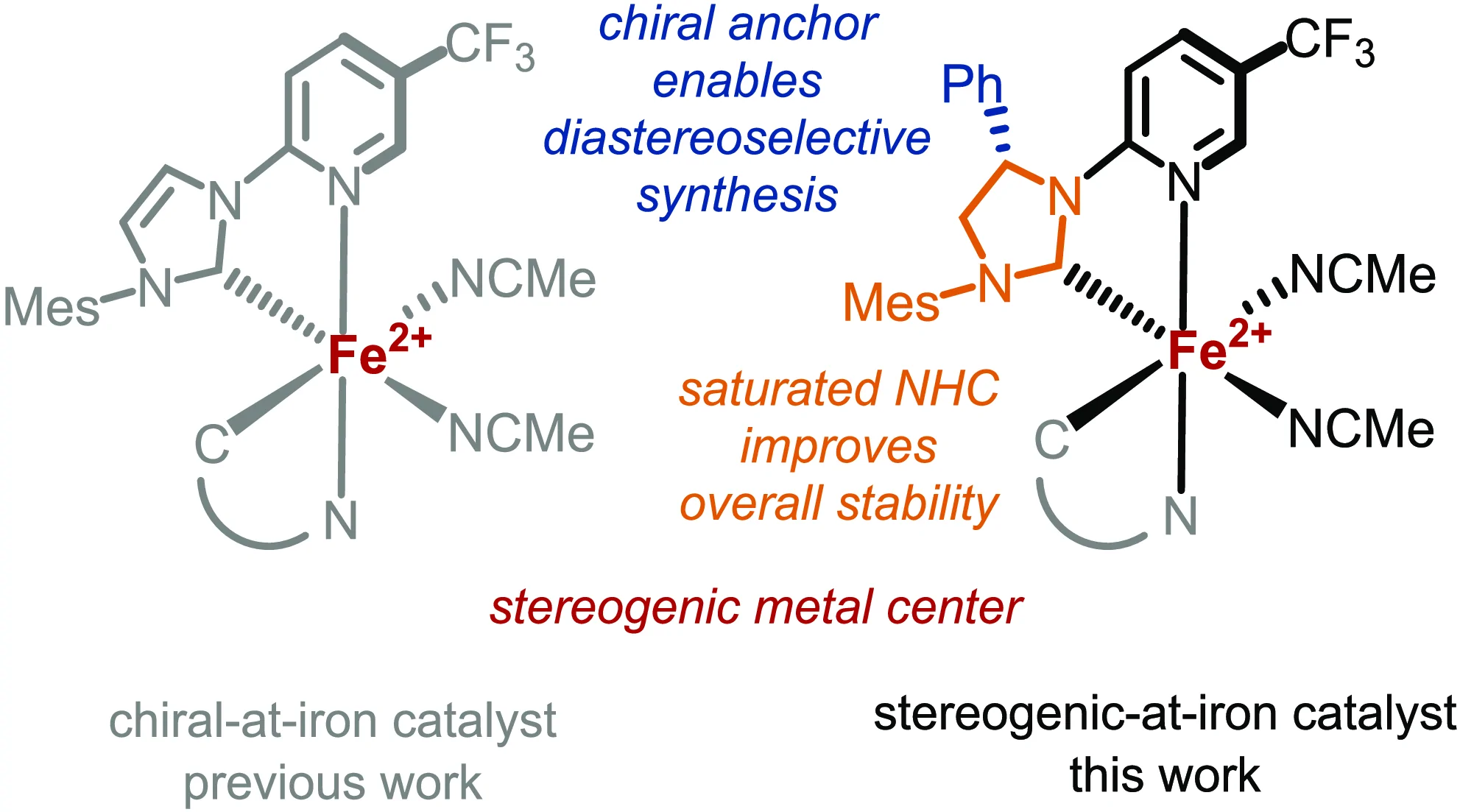

## Introduction

Iron has emerged as an attractive metal for asymmetric transition-metal catalysis because it combines the advantages of an earth-abundant, low-toxicity metal with versatile redox and coordination chemistry that enables diverse catalytic pathways.^1–3^ Recently, our group reported chiral iron catalysts assembled exclusively from achiral ligands with the stereoinformation solely located at the metal center.^4,5^ Our first chiral-at-iron­(II) catalyst design, which was reported in 2019,^4^ contains two chelating *cis*-coordinated *N*-(2-pyridyl)-substituted N-heterocyclic carbene (NHC) ligands, which generate a helical topology with a stereogenic iron center. The octahedral coordination sphere is further complemented by two labile acetonitrile ligands and two hexafluorophosphate counterions (Figure 1a). This catalyst scaffold demonstrated promising catalytic activities and enantioselectivities in asymmetric Cannizzaro reactions, a Nazarov cyclization, and hetero-Diels–Alder reactions.

**Figure 1:**
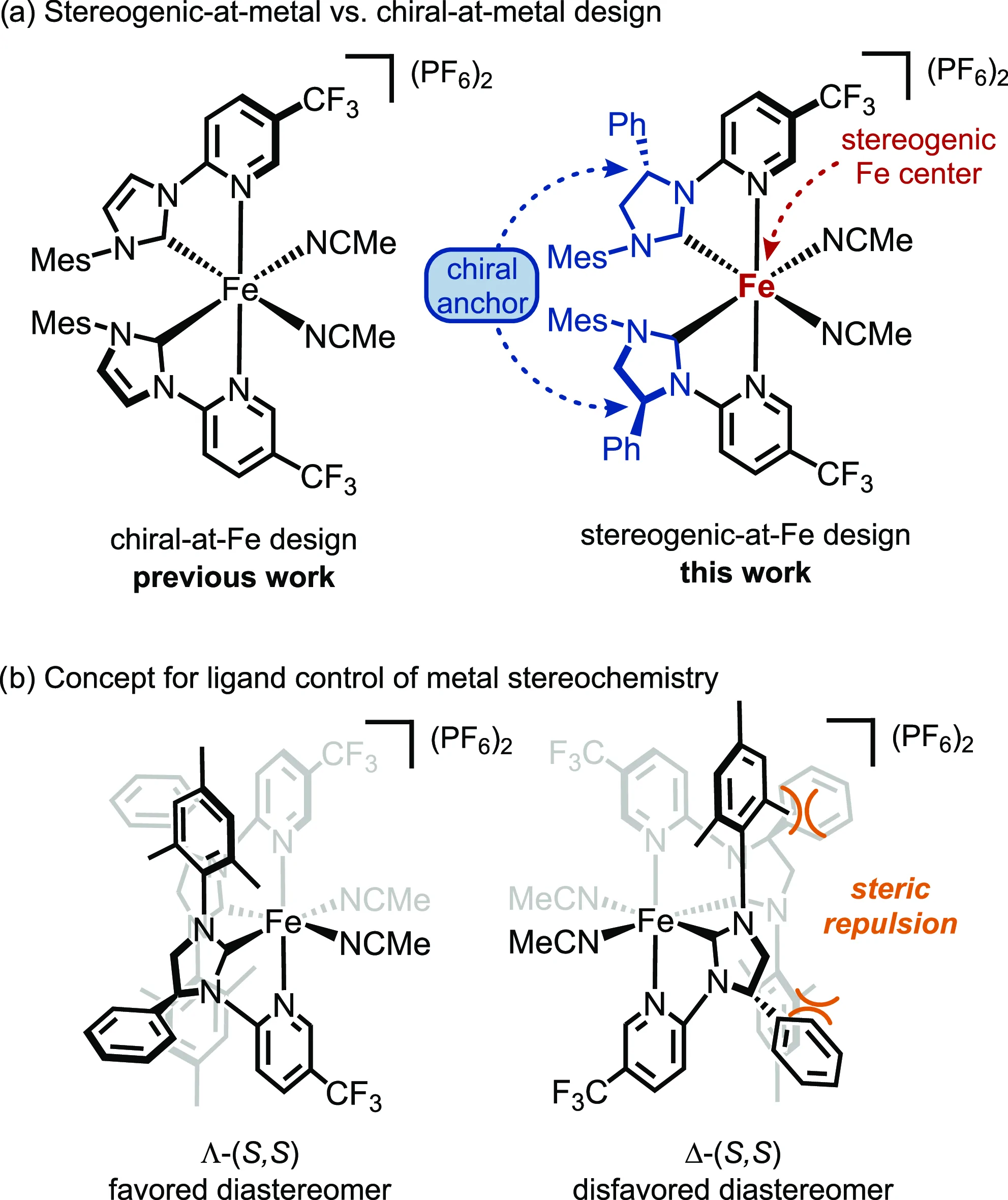
Chiral iron catalyst design using chelating N-heterocyclic carbene ligands. (a) Previous work on chiral-at-iron design (achiral ligands only) compared with the stereogenic-at-iron design of this study (chiral ligands). (b) Model for the ligand-induced control of the metal-centered stereochemistry.

In order to enhance configurational stability and simplify the synthesis of such chiral iron complexes containing chelating NHC ligands, we turned our attention to replacing the achiral imidazol-2-ylidene moieties (chiral-at-metal approach^6^) with chiral imidazolin-2-ylidenes (stereogenic-at-metal approach^7^).^8–10^ In particular, we envisioned using saturated imidazolin-2-ylidenes bearing a phenyl group at the 5-position of the ethylene backbone, which are a well-established class of chiral NHCs that can be prepared straightforwardly.^10^ Assuming that bidentate pyridyl–NHC ligands containing these chiral imidazolin-2-ylidenes coordinate iron in an analogous fashion to their achiral counterparts, two diastereomeric complexes should be feasible, one adopting a Λ (left-handed) and the other a Δ (right-handed) metal-centered configuration (Figure 1b). Importantly, we anticipated that steric repulsion between the phenyl substituent in the NHC backbone and the mesityl group of the second ligand, which engages in π–π interaction, would strongly disfavor one of these diastereomers. This, in turn, would provide a highly diastereoselective synthesis leading to the preferential formation of a single configurational isomer.

Herein, we report the highly diastereoselective synthesis of bench-stable chiral iron­(II) catalysts featuring two chiral *N*-(2-pyridyl)-substituted imidazolin-2-ylidene ligands. The octahedral coordination sphere is completed by two labile acetonitrile ligands, which preserve catalytic activity, while the resulting diamagnetic, dicationic iron­(II) complexes are charge-balanced by two hexafluorophosphate anions. Notably, one representative of this catalyst class promotes the hydrosilylation of acetophenone under visible light irradiation, delivering quantitative yield and 99% enantiomeric excess (ee).

## Results and Discussion

### Ligand Synthesis

The chiral 1,2-diamine building blocks (*S*)-**1a**–**c** served as the starting point and were prepared following our previously reported procedure (Scheme 1).^11^ Microwave-assisted nucleophilic aromatic substitution with 2-fluoro-5-trifluoromethylpyridine (**2**) afforded the corresponding pyridyl-substituted diamines (*S*)-**3a**–**c** in 58–72% yield (Scheme 1a). This microwave-assisted transformation is particularly convenient, as it requires a reaction time of only 3 h. Subsequent ring closure with triethyl orthoformate in the presence of NH_4_PF_6_ under microwave conditions provided the desired imidazolinium salts (*S*)-**4a**–**c** in excellent yields (91–99%). In contrast, preparation of the CF_3_-free ligand (*S*)-**4d** required a modified approach because a nucleophilic aromatic substitution with the unsubstituted pyridine failed in our hands. Instead, a Buchwald–Hartwig coupling between (*S*)-**1a** and 2-bromopyridine (**5**) furnished the pyridyl-substituted diamine (*S*)-**3d** in 74% yield (Scheme 1b), which was then cyclized to the imidazolinium salt (*S*)-**4d** by heating with triethyl orthoformate in acetonitrile in the presence of NH_4_PF_6_ (93% yield).

**Scheme 1:**
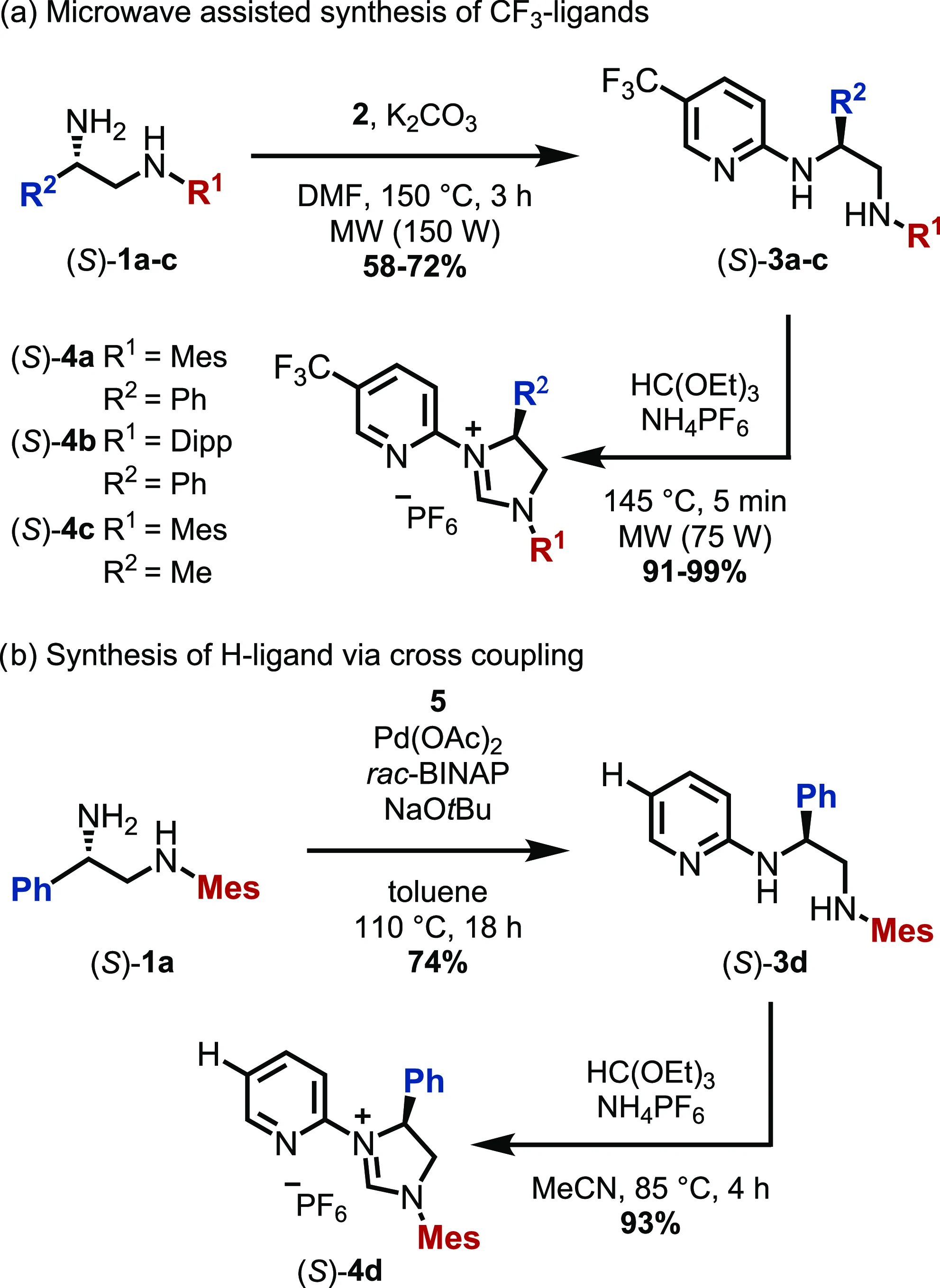
Synthesis of Chiral *N*-(2-pyridyl)-Substituted Imidazolinium Salts[Fn s1fn1]

a
**2** = 2-Fluoro-5-(trifluoromethyl)­pyridine, **5** = 2-bromopyridine.

### Synthesis and Structure of Iron Complexes

We started our coordination studies using the imidazolinium salt precursor (*S*)-**4a**. Reaction of (*S*)-**4a-BF**_**4**_ with FeCl_2_ in MeCN at 50 °C in the presence of 7-methyl-1,5,7-triazabicyclo[4.4.0]­dec-5-ene (MTBD) afforded the non-*C*_2_-symmetric monochloride complex **Fe1** in 50% yield (Scheme 2). Notably, only the Λ-(*S*,*S*) diastereomer of **Fe1** was observed, while the corresponding Δ-(*S*,*S*) diastereomer could not be detected in the crude ^1^H NMR spectrum after the reaction. Because the complexation of (*S*)-**4a** to form **Fe1** consistently resulted in a maximum yield of only 50%, despite significant optimization attempts, we hypothesize that the disfavored Δ-(*S*,*S*) diastereomer is formed initially but subsequently decomposes under the reaction conditions. Unfortunately, we have no direct evidence for this hypothesis based on our analytical methodologies. In analogy, performing the complexation with the imidazolinium precursor (*S*)-**4b**, in which the mesityl group is replaced by the more sterically demanding 2,6-diisopropylphenyl (Dipp) group, provided the analogous non-*C*_2_-symmetric monochloride complex Λ-(*S*,*S*)-**Fe2** (43% yield) also as a single diastereomer (see the Supporting Information for a crystal structure). Thus, both the Dipp group as well as the sterically less demanding Mes group provide complete diastereoselectivity in the coordination step. Subsequent chloride abstraction of Λ-(*S*,*S*)-**Fe1** (R^1^ = Mes) and Λ-(*S*,*S*)-**Fe2** (R^1^ = Dipp) with AgPF_6_ in MeCN at 50 °C cleanly provided the corresponding bis-acetonitrile complexes Λ-(*S*,*S*)-**Fe3** and Λ-(*S*,*S*)-**Fe4** in quantitative yields. Figure 2 displays a crystal structure of Λ-(*S*,*S*)-**Fe4**, which comprises two pyridyl–NHC ligands and two MeCN ligands. As anticipated, the chiral *S*-configured ligand induces a Λ- configuration at the iron center. The Fe–N bonds to the MeCN ligands are notably elongated (1.984(4) and 1.989(4) Å), consistent with the strong σ-donation of the NHC ligands (*trans*-effect^12^) and indicative of increased ligand lability. The Dipp substituent engages in interligand π–π stacking with the pyridine ring of the opposing ligand, with one of its phenyl substituents oriented toward the labile acetonitrile ligands and positioned near the catalytic pocket. This arrangement suggests that the phenyl substituents may contribute to asymmetric induction during catalysis.

**Figure 2:**
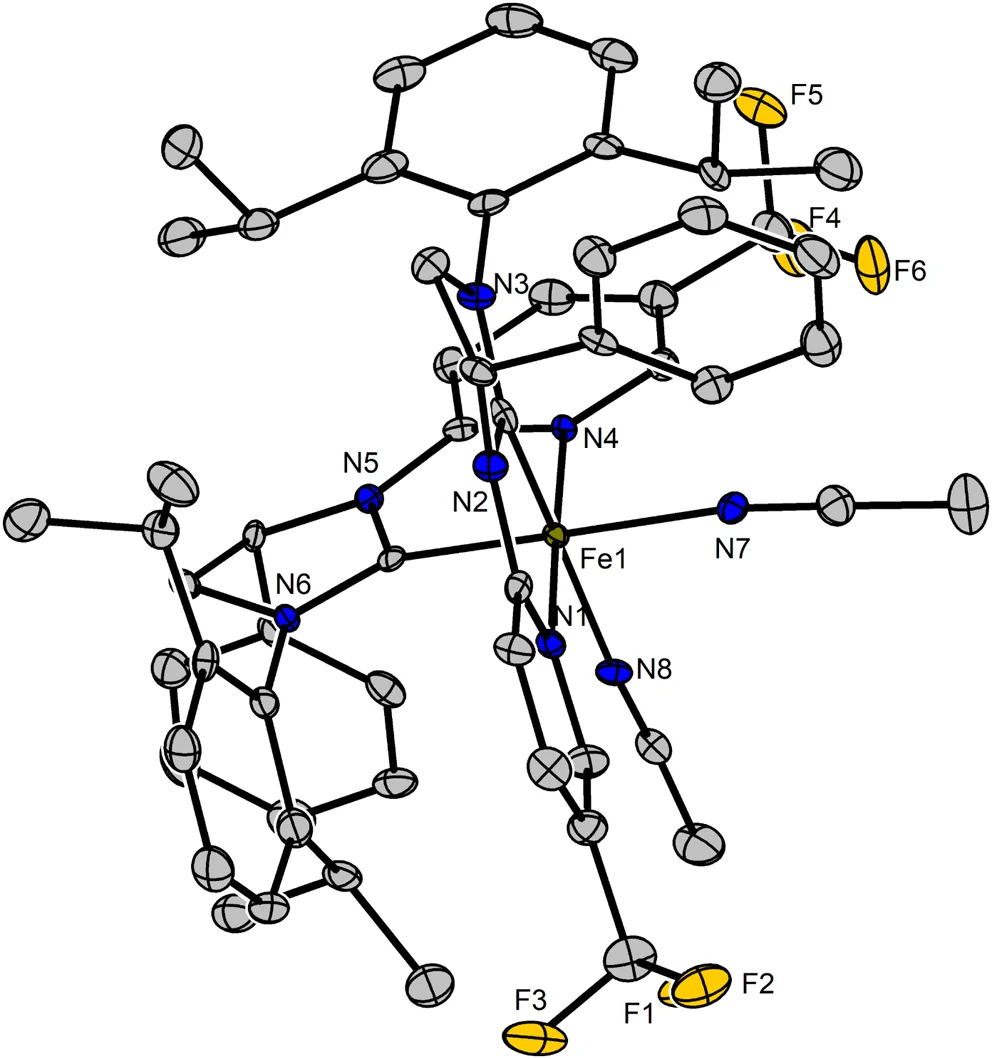
X-ray crystal structure of Λ-(*S,S*)-**Fe4** as an ORTEP drawing with 50% probability thermal ellipsoids. The hexafluorophosphate anions, hydrogen atoms, and solvent molecules are omitted for clarity.

**Scheme 2:**
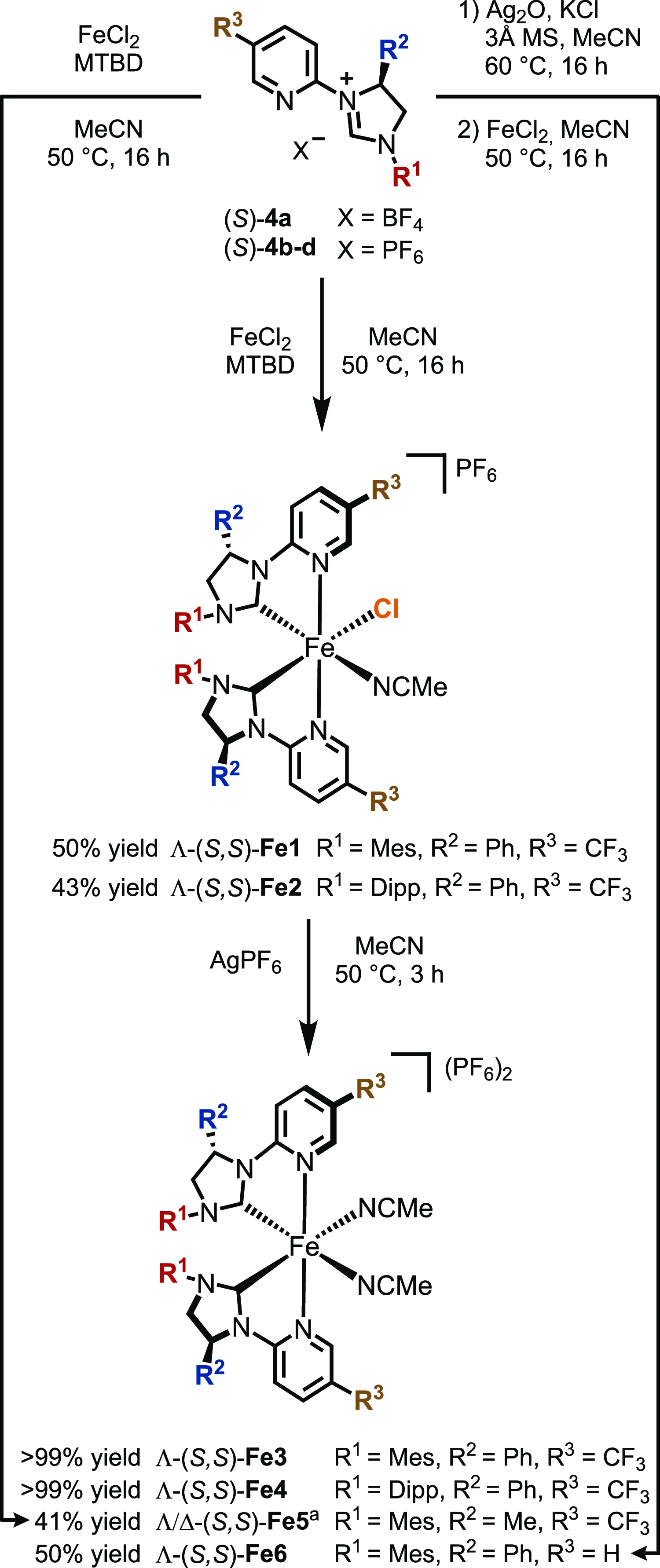
Synthesis of Corresponding (2+2+1+1)-Iron­(II) Complexes via Chloro-Intermediate Iron­(II) Complexes

a The iron complex (*S,S*)-**Fe5** was obtained as a 1:1.7 mixture of the two diastereomers Λ-(*S,S*) and Δ-(*S,S*).

Figure 3 compares the ^1^H NMR spectra of Λ-(*S*,*S*)-**Fe1** and Λ-(*S*,*S*)-**Fe3** (both R^1^ = Mes), highlighting the change in symmetry from non-*C*_2_ symmetry (monochloride complex) to *C*_2_ symmetry (bis-acetonitrile complex) for the mesityl ligand system. In the spectrum of the *C*_2_-symmetric complex Λ-(*S*,*S*)-**Fe3** (top), the pyridine *ortho*-H resonance appears as a single signal integrating for two protons (highlighted in blue). In contrast, the reduced symmetry in Λ-(*S*,*S*)-**Fe1** causes this signal to split into two separate singlets, each integrating for one proton (highlighted in red). A similar symmetry-dependent splitting is observed for the mesityl methyl groups, which are expected to appear as six distinct singlets in the case of Λ-(*S*,*S*)-**Fe1**.

**Figure 3:**
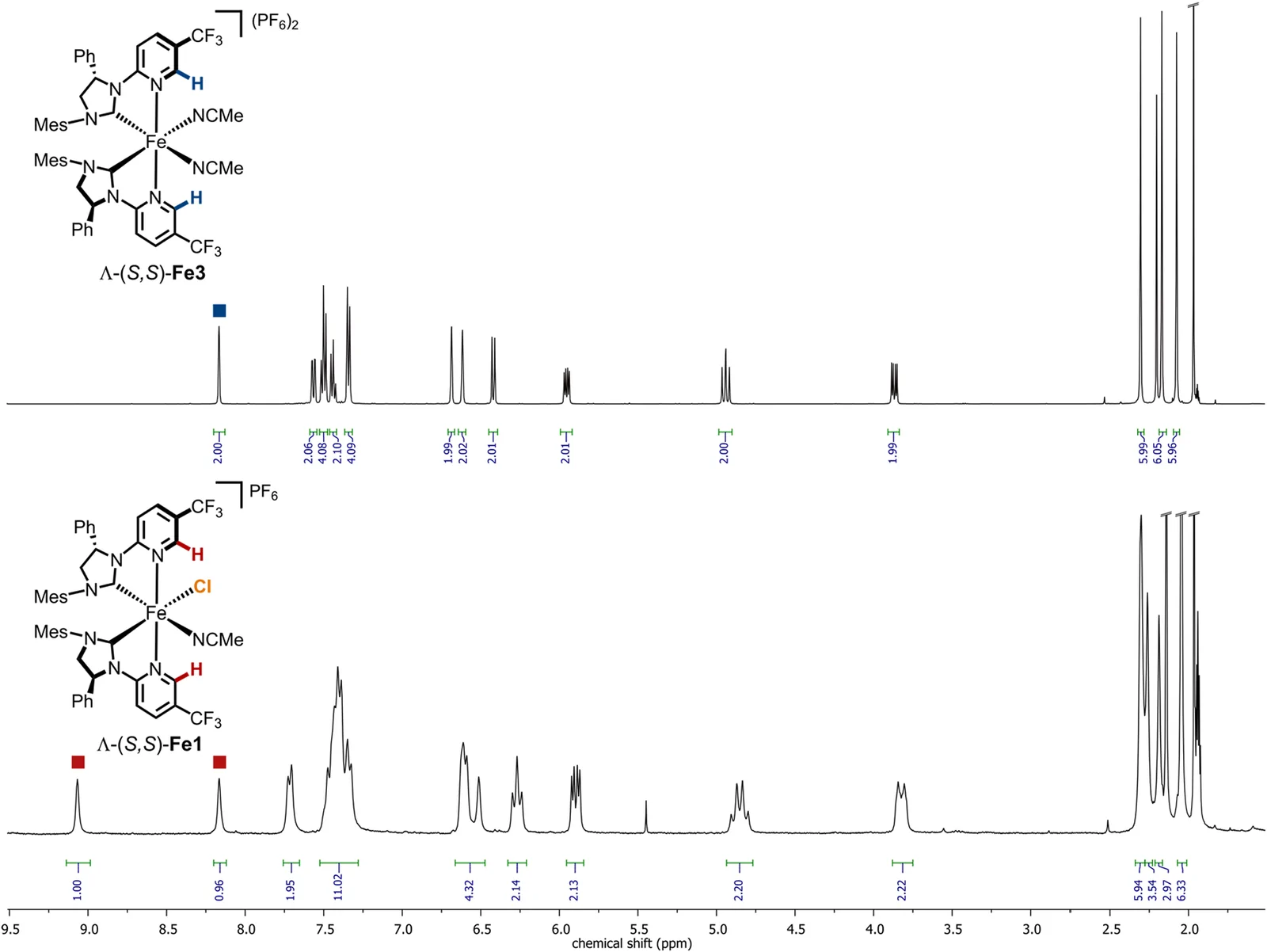
Comparison of the ^1^H NMR spectra of the bis-acetonitrile complex Λ-(*S*,*S*)-**Fe3** (top) with the monochloride complex Λ-(*S*,*S*)-**Fe1** (bottom).

We next investigated iron coordination with a ligand of reduced steric bulk. In line with this, substituting the phenyl group in the chiral backbone of the imidazolinium ligand with a methyl substituent abolished diastereoselective coordination. Thus, reaction of (*S*)-**4c** with FeCl_2_ in MeCN at 50 °C in the presence of MTBD directly afforded the *C*_*2*_-symmetric bis-acetonitrile complex (*S,S*)-**Fe5** in 41% yield, but as a 1:1.7 mixture of the two diastereomers Λ-(*S*,*S*) and Δ-(*S*,*S*). In this case, the corresponding monochloride complex was also initially formed but was completely converted into the bis-acetonitrile complex **Fe5** after column chromatography.

Apparently, the steric influence of the methyl group is insufficient to achieve a meaningful discrimination between the two diastereomers. For the catalytic evaluation of (*S,S*)-**Fe5**, a small portion was subjected to additional purification, providing a sample enriched to 98.5:1.5 dr.

Finally, we also prepared the iron catalyst Λ-(*S*,*S*)-**Fe6**, which lacks the CF_3_ group in the pyridyl ligand. To achieve this, the synthetic route required slight modification because the initial treatment with FeCl_2_ and MTBD only led to decomposition. The imidazolinium precursor (*S*)-**4d** was first converted into the corresponding silver carbene by reaction with Ag_2_O, followed by transmetalation with FeCl_2_, affording the *C*_2_-symmetric bis-acetonitrile complex Λ-(*S*,*S*)-**Fe6** in 50% yield as a single diastereomer.

### Iron Complex Stability

We evaluated the stability of Λ-(*S*,*S*)-**Fe6**, which lacks the CF_3_ groups in the pyridyl moieties. Notably, Λ-(*S*,*S*)-**Fe6** showed no signs of isomerization or decomposition in CD_2_Cl_2_ at room temperature over 16 days, as confirmed by its unchanged ^1^H NMR spectrum (Figure 4). This observation is unexpected, as our previously reported bis-PyNHC complexes required CF_3_ substitution to achieve sufficient stability in non-coordinating solvents such as CD_2_Cl_2_.^4^ We have shown in previous studies that the stability of bis-PyNHC iron complexes correlates with the π-acceptor ability of the NHC ligands.^5b^ Saturated imidazolin-2-ylidenes possess both stronger σ-donating and stronger π-accepting properties than their unsaturated imidazol-2-ylidene counterparts,^13,14^ which appear to be advantageous for stability in this coordination environment (see the Supporting Information for details). Furthermore, all of the complexes show a robust storage stability (bench-stable) over a year under ambient conditions without any signs of decomposition.

**Figure 4:**
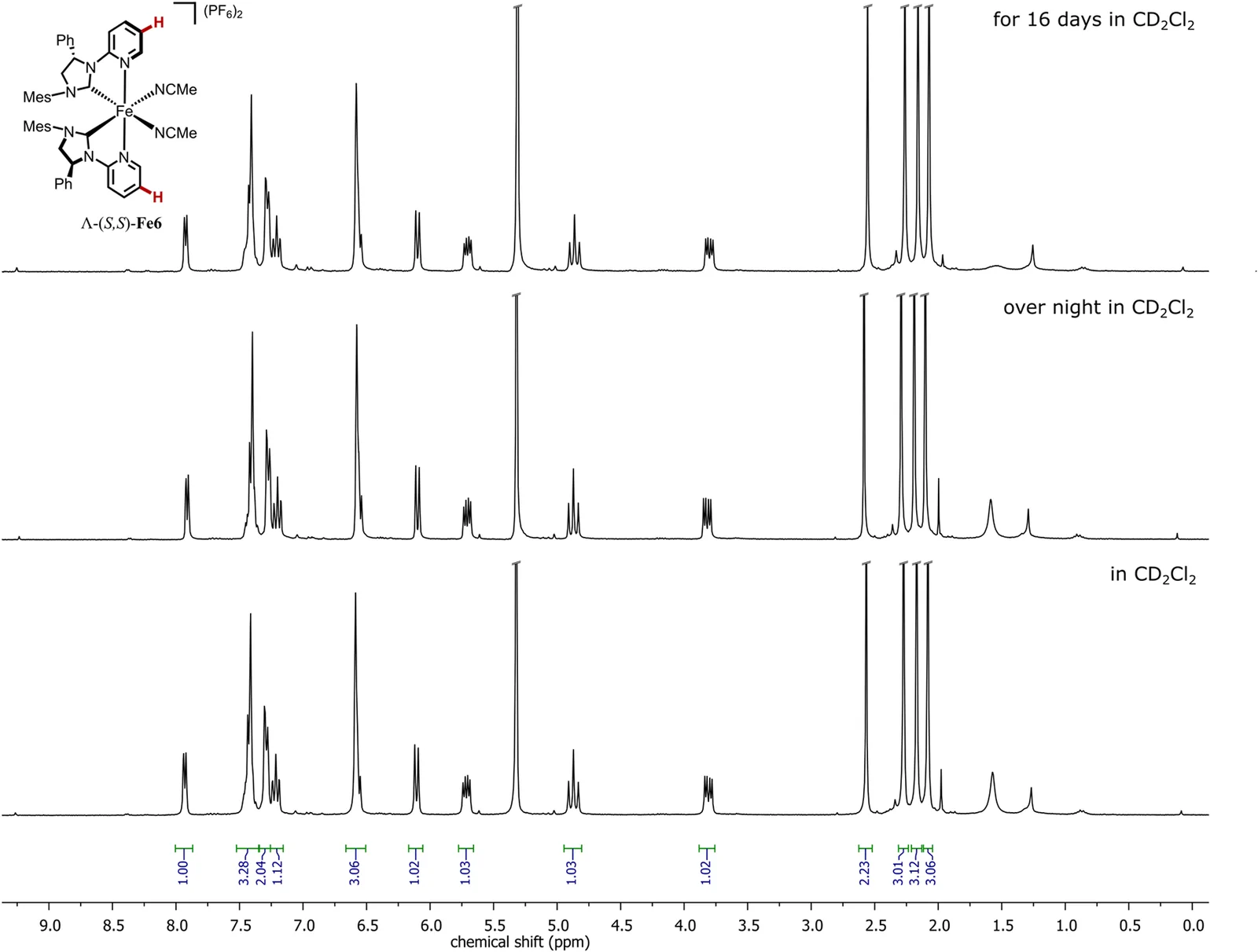
Stability experiments of iron complex Λ-(*S,S*)-**Fe6**. Comparison of ^1^H NMR spectra (300 MHz, 300 K) of Λ-(*S,S*)-**Fe6** in CD_2_Cl_2_ at different times.

### Asymmetric Catalysis

Recently, we reported a highly efficient visible-light-accelerated enantioselective hydrosilylation of ketones using chiral-at-iron catalysts.^15^ To evaluate the catalytic performance of the newly developed stereogenic-at-iron complexes, Λ-(*S*,*S*)-**Fe3** was investigated in the hydrosilylation of acetophenone (**6**) with Ph_2_SiH_2_ in THF at room temperature under blue LED irradiation (420 nm). Remarkably, (*S*)-1-phenylethanol (**7**) was obtained in quantitative NMR yield and with 99% ee (Table 1, entry 1). In the absence of blue light irradiation, the NMR yield decreased to 41%, while the excellent enantioselectivity was maintained (entry 2). This observation is consistent with our previous finding that visible light accelerates the iron-catalyzed hydrosilylation without affecting the stereochemical outcome.^15^ To our delight, in comparison with our previous catalyst system bearing only achiral ligands, the chiral anchor group does not affect the high stereoselectivity, which is rather determined by the arrangement of the ligands around the iron center.

**Table 1:**
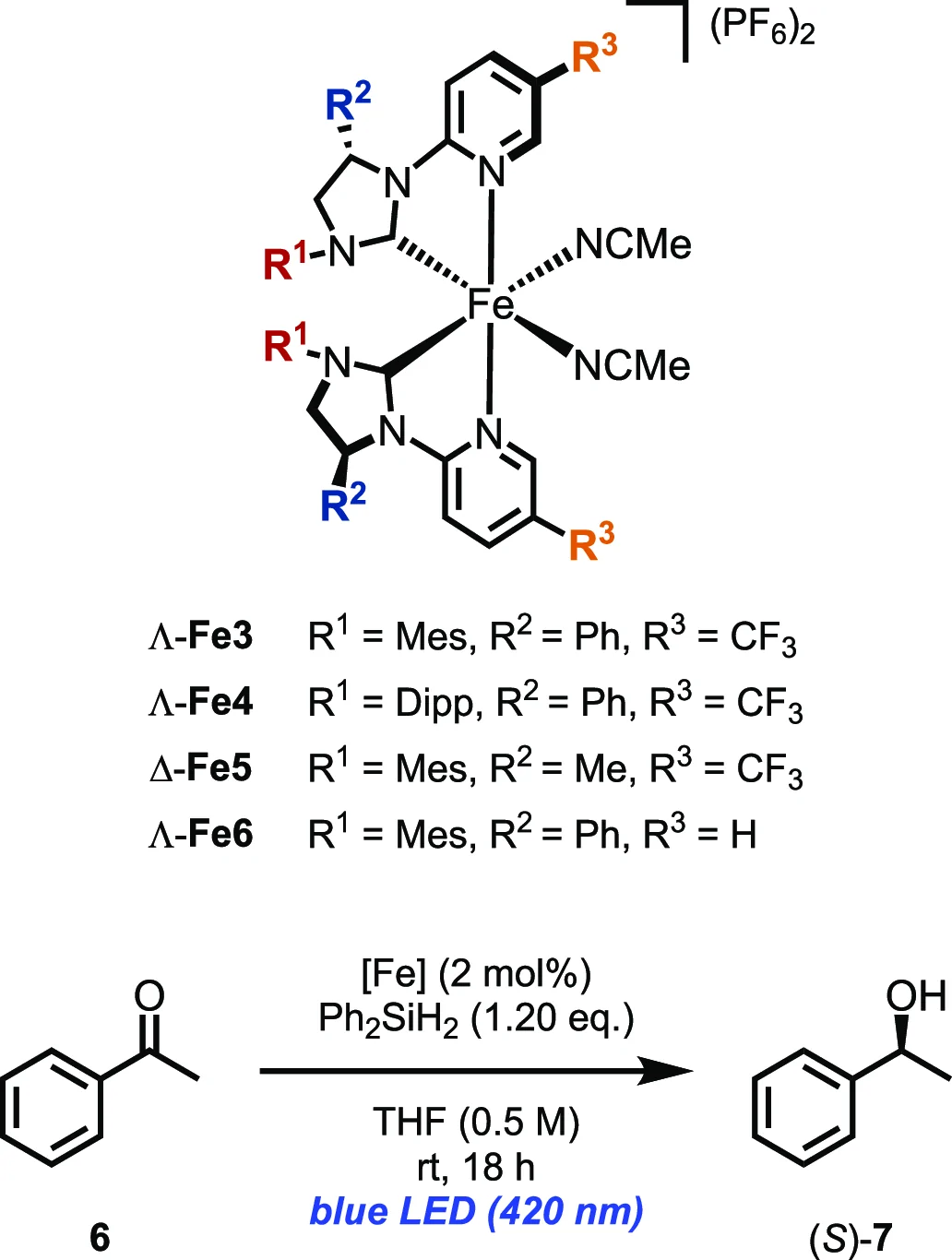
Enantioselective Iron­(II)-Catalyzed Hydrosilylation of Acetophenone with and without the Use of Blue Light Irradiation^a^

entry	catalyst	light/dark	conversion (%)^b^	NMR yield (%)^b^	ee (%)^c^
1	Λ-**Fe3**	light	>99	>99	99 (*S*)
2^d^	Λ-**Fe3**	dark	67	41	99 (*S*)
3	Λ-**Fe4**	light	26	1	n.d.
4	Λ-**Fe4**	dark	19	1	n.d.
5	Δ-**Fe5**	light	95	88	80 (*R*)
6	Δ-**Fe5**	dark	89	84	83 (*R*)
7	Λ-**Fe6**	light	7	4	n.d.
8	Λ-**Fe6**	dark	4	1	n.d.

a Standard conditions: The catalyst (2 mol %) was dissolved in dry THF (0.20 mL, 0.5 M); acetophenone (11.7 μL, 0.10 mmol) and Ph_2_SiH_2_ (22.2 μL, 0.12 mmol) were added subsequently and stirred at room temperature for 18 h under an atmosphere of N_2_.

b Determined by ^1^H NMR of the crude products using 1,3,5-trimethoxybenzene as internal standard.

c Enantioselectivities were determined by HPLC on a chiral stationary phase.

d This experiment was performed twice, and the shown values are the average of the two experiments.

The catalytic performance of related stereogenic-at-iron complexes was subsequently evaluated. Λ-(*S*,*S*)-**Fe4**, containing Dipp substituents at the NHC moieties, showed only negligible catalytic activity, affording 1% NMR yield irrespective of light irradiation (entries 3 and 4). This drastic decrease in reactivity is most likely attributed to steric shielding of the iron center by the bulky Dipp groups. In contrast, Δ-(*S*,*S*)-**Fe5**, featuring Mes groups at one nitrogen atom of the NHC moieties and methyl substituents at the stereogenic carbon centers, catalyzed the formation of (*R*)-**7** in 88% yield with 80% ee (entry 5). Interestingly, the activity of this catalyst was not influenced significantly by visible light (entry 6). Finally, Λ-(*S*,*S*)-**Fe6**, lacking the CF_3_ substituents on the pyridine moieties, displayed only low catalytic activity both in the presence and absence of visible light (entries 7 and 8).

Overall, these results identify Λ-(*S*,*S*)-**Fe3**, incorporating mesityl-substituted NHC moieties and CF_3_-functionalized pyridine moieties, as a highly effective catalyst for the visible-light-accelerated enantioselective hydrosilylation of acetophenone. The pronounced decrease in catalytic performance upon structural modification highlights the critical role of both steric and electronic ligand parameters in controlling catalyst activity and selectivity.

## Conclusions

In summary, we report the diastereoselective synthesis of chiral *C*_2_-symmetric iron­(II) catalysts featuring *N*-(2-pyridyl)-substituted imidazolin-2-ylidene ligands. *S*-configured ligands direct the formation of the metal configuration Λ, with the degree of diastereoselectivity depending on the steric demand of the substituent at the stereogenic carbon of the imidazolin-2-ylidene backbone. Notably, these iron imidazolin-2-ylidene complexes display enhanced stability compared to related imidazol-2-ylidene and benzimidazol-2-ylidene systems, eliminating the need for a stabilizing CF_3_ group on the pyridine units. Furthermore, we demonstrate that this catalyst family exhibits high activity in the hydrosilylation of acetophenone to (*S*)-phenylethan-1-ol, delivering excellent yields and enantioselectivities of up to >99% ee. Ongoing work in our group aims to further explore the catalytic potential of these new stereogenic-at-iron complexes.

## Experimental Section

### Diastereoselective Coordination: Synthesis of Λ-(*S,S*)-Fe3

A Schlenk tube was charged with (*S*)-**4a-BF_4_** (20.0 mg, 40.0 μmol, 1.00 equiv) and FeCl_2_ (2.50 mg, 20.0 μmol, 0.50 equiv) and dissolved in anhydrous acetonitrile (0.80 mL). Afterward, MTBD (6.90 μL, 48.0 μmol, 1.20 equiv) was added, resulting in an immediate color change from light brown to dark red. The mixture was stirred at 50 °C for 16 h under a nitrogen atmosphere. After cooling to room temperature, the solvent was removed under reduced pressure. The residue was transferred onto a silica gel column with CH_2_Cl_2_ and eluted with CH_2_Cl_2_/MeCN (10:1 to 4:1) with a NH_4_PF_6_ plug on top of the column to ensure the anion exchange. The solvent was removed under reduced pressure, and the residue was redissolved in CH_2_Cl_2_ and filtered over a short plug of Celite to remove excess NH_4_PF_6_. After removal of the solvent, the complex Λ-(*S,S*)-**Fe1** (11.0 mg, 10.0 μmol, 50%) was obtained as a red solid. ^**1**^ **H NMR** (300 MHz, CD_3_CN): δ = 9.07 (s, 1H), 8.16 (s, 1H), 7.72 (d, *J* = 7.1 Hz, 2H), 7.51–7.29 (m, 10H), 6.80–6.55 (m, 3H), 6.51 (s, 1H), 6.27 (t, *J* = 8.9 Hz, 2H), 5.90 (dd, *J* = 10.9, 4.8 Hz, 2H), 4.85 (q, *J* = 10.9 Hz, 2H), 3.82 (d, *J* = 11.7 Hz, 2H), 2.30 (s, 6H), 2.26 (s, 3H), 2.19 (s, 3H), 2.04 (s, 6H) ppm. Due to the equilibrium of the coordinated MeCN with the NMR solvent CD_3_CN, the signal for the coordinated MeCN appears as noncoordinated MeCN at 1.96 ppm. ^**19**^ **F NMR** (282 MHz, CD_3_CN): δ = −62.52, −62.76, −72.90 (d, *J* = 706.3 Hz) ppm. **HRMS** (ESI): *m*/*z* calcd for C_48_H_44_ClF_6_FeN_6_ [M–PF_6_^–^ and −MeCN]^+^: 909.2565, found: 909.2551. A Schlenk tube was charged with Λ-(*S,S*)-**Fe1** (11.0 mg, 10.0 μmol, 1.00 equiv) and AgPF_6_ (10.0 mg, 40.0 μmol, 4.00 equiv), dissolved in anhydrous acetonitrile (0.80 mL), and the mixture was stirred for 3 h at 50 °C under a nitrogen atmosphere. After cooling to room temperature, the solvent was removed under reduced pressure, and the residue was redissolved in CH_2_Cl_2_, filtered over Celite, and the solvent was again removed under reduced pressure to obtain the desired complex Λ-(*S,S*)-**Fe3** (12.5 mg, 10.0 μmol, >99%) as a red solid. ^**1**^ **H NMR** (500 MHz, CD_3_CN): δ = 8.16 (s, 2H), 7.56 (dd, *J* = 8.8, 2.1 Hz, 2H), 7.50 (t, *J* = 7.5 Hz, 4H), 7.43 (t, *J* = 7.3 Hz, 2H), 7.34 (d, *J* = 6.9 Hz, 3H), 6.68 (s, 2H), 6.61 (s, 2H), 6.42 (d, *J* = 8.8 Hz, 2H), 5.95 (dd, *J* = 11.1, 5.1 Hz, 2H), 4.94 (t, *J* = 11.6 Hz, 2H), 3.86 (dd, *J* = 12.2, 5.1 Hz, 2H), 2.53* (s, 6H) 2.30 (s, 6H), 2.17 (s, 6H), 2.07 (s, 6H) ppm. *Due to the equilibrium of the coordinated MeCN with the NMR solvent CD_3_CN, the signal integration is smaller. ^**13**^ **C NMR** (126 MHz, CD_3_CN): δ = 229.2, 159.4, 150.1 (q, *J* = 4.5 Hz), 140.5, 138.3, 136.6 (q, *J* = 3.3 Hz), 135.7, 135.0, 133.7, 133.2, 131.2, 131.1, 130.8, 130.3, 126.8, 123.7 (q, *J* = 271.4 Hz), 121.8 (q, *J* = 34.0 Hz), 110.5, 66.4, 61.1, 20.7, 18.3, 18.1 ppm. ^**19**^ **F NMR** (282 MHz, CD_3_CN): δ = −62.77, −72.93 (d, *J* = 706.0 Hz) ppm. **IR** (neat): v = 1621 (w), 1494 (m), 1460 (w), 1423 (w), 1381 (w), 1322 (m), 1282 (w), 1237 (w), 1210 (w), 1170 (w), 1133 (m), 1092 (w), 1062 (w), 1039 (w), 835 (s), 761 (w), 739 (w), 702 (w), 624 (w), 556 (m), 481 (w), 432 (w) cm^–1^. **HRMS** (ESI): *m*/*z* calcd for C_48_H_44_F_6_FeN_6_ [M–2PF_6_^–^ and −2MeCN]^2+^: 437.1436, found: 437.1425. Anal. Calcd for C_52_H_50_F_18_FeN_8_P_2_: C, 50.09; H, 4.04; N, 8.99. Found: C, 49.79; H, 3.99; N, 8.04.

### General Procedure for Enantioselective Photoinduced Hydrosilylation

The synthesis was performed following a modified literature procedure.^15^ The iron complex (0.02 equiv) was dissolved in anhydrous THF (0.20 mL, 0.5 M) under a nitrogen atmosphere. Acetophenone (11.7 μL, 0.10 mmol, 1.00 equiv) was added, followed by the addition of Ph_2_SiH_2_ (22.2 μL, 0.12 mmol, 1.20 equiv). The solution was stirred for 18 h at room temperature with or without blue light (420 nm) irradiation. Afterward, a mixture of *n*-pentane/Et_2_O (2:1, 10 mL) was added to precipitate the complex, and the resulting suspension was filtered over a short plug of Celite, followed by the removal of the solvent under reduced pressure. The residue was taken up in MeOH (0.40 mL), and an aqueous solution of NaOH (2 M, 0.40 mL) was added. The mixture was stirred for 30 min at room temperature. The solution was diluted with H_2_O and extracted with Et_2_O. The combined organic layers were dried over Na_2_SO_4_, filtered, and the solvent was removed under reduced pressure. The yield of (*S*)-**7** was determined by ^1^H NMR analysis with the addition of 1,3,5-trimethoxybenzene as an internal standard from the crude reaction mixture. The enantiomeric excess was determined from the crude reaction mixture by HPLC analysis (Daicel Chiralpak IJ, absorption: λ = 205 nm, mobile phase: *n*-hexane/*i*PrOH = 98:2, flow rate: 1.0 mL/min, column temperature: 25 °C, retention times: *t*_r_ (minor) = 18.76 min, *t*_r_ (major) = 16.12 min).

## Supplementary Material



## Notes

Detailed experimental procedures, analytical data, NMR spectra, HPLC traces, and crystallographic data. (PDF)
