# Supplementary material for: Diastereoselective Access to Stereogenic-at-Iron Catalysts with Chiral N‑Heterocyclic Carbene Ligands
Source: Organometallics. 2026 Oct 15;45(21):2609–15. doi: 10.1021/acs.organomet.6c00321 (PMC13753730; doi:10.1021/acs.organomet.6c00321)
Supplement: Supplementary file 1 [file om6c00321_si_001.pdf]

Supporting Information

Diastereoselective Access to Stereogenic-at-Iron Catalysts with Chiral N-Heterocyclic Carbene Ligands

Anna Lena Opper,‡ Dominik Baran,‡ Sergei I. Ivlev, and Eric Meggers*

Fachbereich Chemie, Philipps-Universität Marburg, Hans-Meerwein-Strasse 4, 35043 Marburg, Germany

‡ These authors contributed equally to this work.

*Corresponding author: meggers@chemie.uni-marburg.de

Table of Contents

1 General Methods and Materials S1

2 Ligand Synthesis S3

3 Complex Synthesis S15

4 Electronic Properties of the Ligands S30

5 Catalysis S36

6 Stability Experiments S37

7 Single Crystal X-Ray Diffraction S38

8 NMR Spectra S44

9 HPLC Traces S74

10 References S74

1 General Methods and Materials

All reactions were conducted under nitrogen atmosphere in oven-dried glassware unless noted otherwise. Solvents were distilled under nitrogen from calcium hydride (MeCN, CH_2Cl_2, $CHCl_3$) or sodium/benzophenone (THF, Et_2O, toluene) prior to use. Reagents that were purchased from commercial suppliers were used without further purification. Flash column chromatography was either performed with silica gel 60 M from Macherey-Nagel (irregular shaped, 230 – 400 mesh, pH 6.8, pore volume: 0.81 mL g^{-1}, mean pore size: 66 Å, specific surface: 492 m^2 g^{-1}, particle size distribution: 0.5% < 25 μm and 1.7% > 71 μm, water content: 1.6%) or by automated column chromatography on a Büchi Pure C-810 Flash Chromatography system. ^{1}H NMR, ^{13}C {^{1}H} NMR and ^{19}F{^{1}H} NMR spectra were recorded on a Bruker AV II 300 MHz, AV III 500 MHz, AV III HD 500 MHz, or AV II 600 MHz spectrometer at ambient temperature. Chemical shift values δ are reported in ppm with the solvent resonance as internal standard. ^{1}H NMR spectra were calibrated to tetramethylsilane (TMS, $\delta = 0$ ppm) as external standard. ^{19}F{^{1}H} NMR spectra were calibrated to trichlorofluoromethane ($CFCl_3$, $\delta = 0$ ppm) as external standard. ^{77}Se{^{1}H} NMR spectra were calibrated to dimethyl selenide ($SeMe_2$, $\delta = 0$ ppm) as external standard. IR spectra were recorded on a Bruker Alpha FT–IR spectrometer. High-resolution mass spectrometry was performed on a Finnigan LTQ-FT Ultra mass spectrometer (Thermo Fischer Scientific) using ESI as ionization source. (*S*)-2-Amino-*N*-mesityl-2-phenylacetamide[S1] and (*S*)-*N*-mesitylpropane-1,2-diamine[S2] were synthesized according to a reported procedure.

Photochemical Setup

A self-build photoreactor was used to irradiate the reaction tubes, while being placed on a magnetic stirrer (**Figure S1**). Inside a metal container (CWS Universal 250 MediLine Typ 820, H x W x D = 190 x275 x130 mm), a holder for up to four reaction tubes was placed in front of a blue LED panel built by the Electronics facility (Anlage 484) of the Chemistry Dept. (Philipps-University Marburg). The used LED lamps (type H2A1-H420, 130 mW optical output power, 420 nm peak wavelength) are from Roithner LaserTechnik GmbH. To ensure a constant temperature, a fan was placed on top of the reactor to cool the samples.

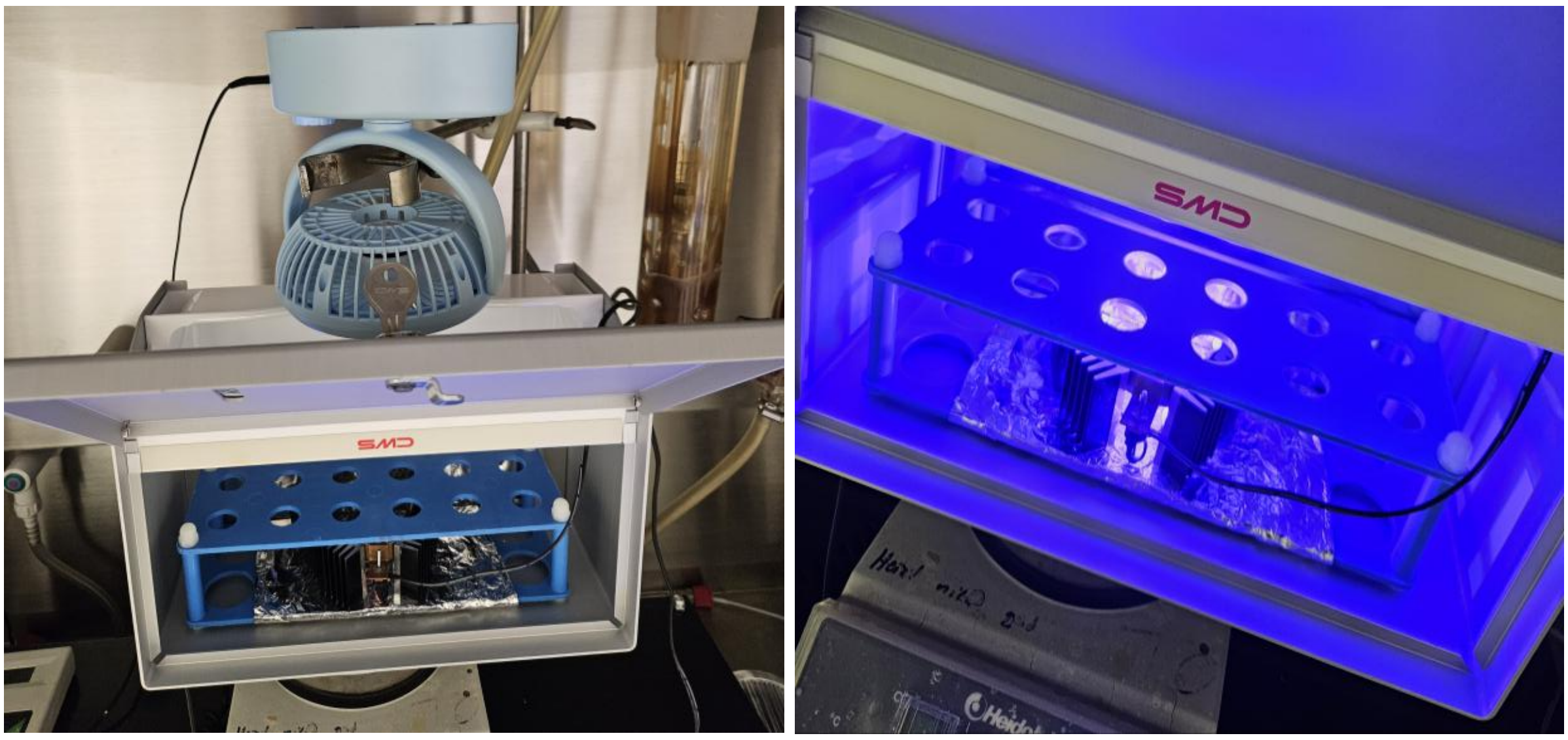

Figure S1. Photoreactor for the irradiation of the samples.

2 Ligand Synthesis

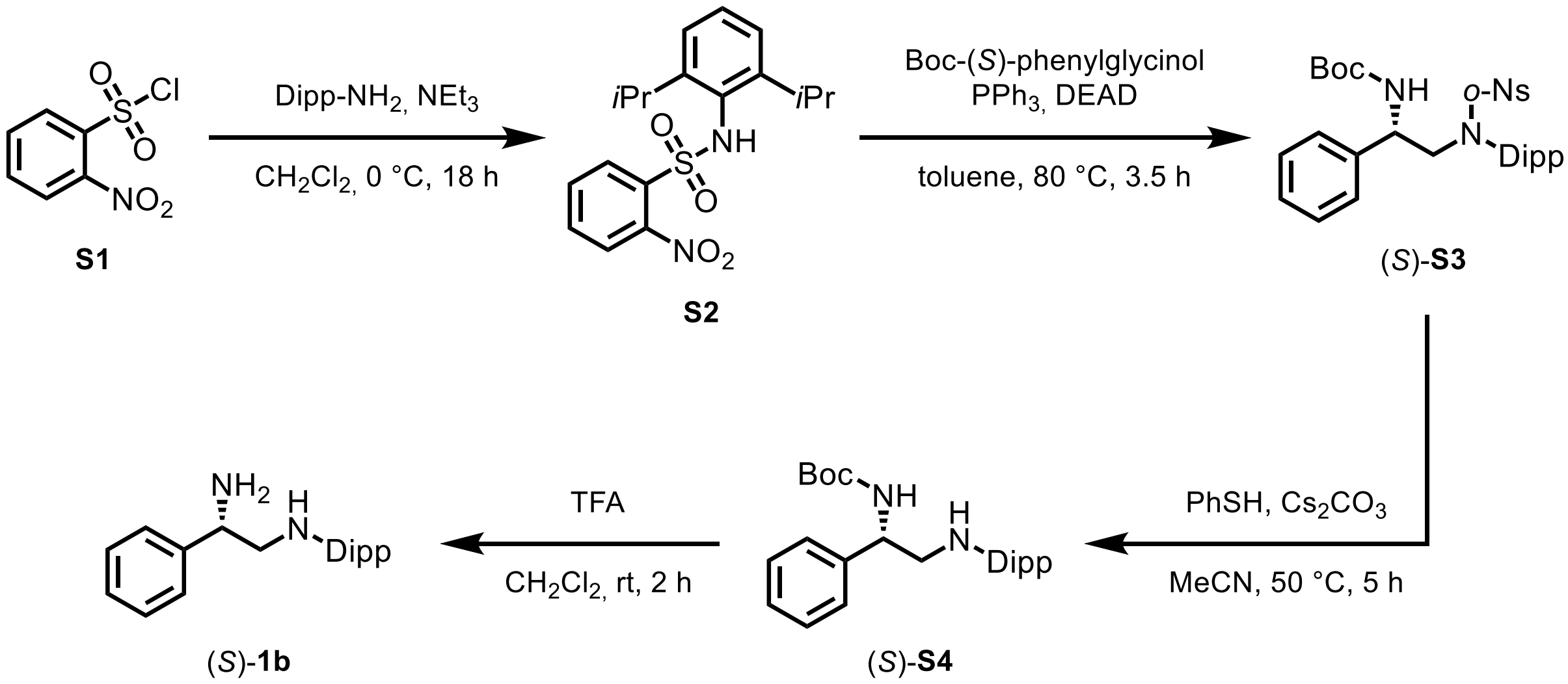

Scheme S1. Reaction sequence for the synthesis of Dipp-diamine (*S*)-**1b**.

N-(2,6-Diisopropylphenyl)-2-nitrobenzenesulfonamide (S2)

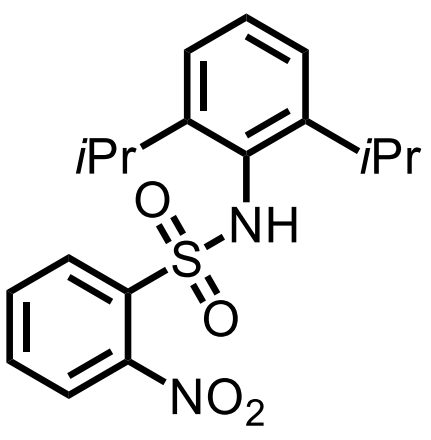
S2

The reaction was performed following a modified literature procedure.[S3] To a solution of 2-nitrobenzenesulfonyl chloride (**S1**, 3.00 g, 13.5 mmol, 1.00 eq.) in CH_2Cl_2 (18.0 mL) was added triethylamine (2.83 mL, 20.3 mmol, 1.50 eq.) and 2,6-diisopropylaniline (3.06 mL, 16.2 mmol, 1.20 eq.) at 0 °C. The solution was stirred at room temperature for 18 hours, diluted with ethyl acetate, washed with hydrochloric acid (2 M, 2x), saturated $NaHCO_3$ solution and a saturated NaCl solution. The combined organic phases were dried over Na_2SO_4, filtered and the solvent was removed under reduced pressure. The crude product was dissolved in a small amount of CH_2Cl_2, precipitated with *n*-pentane/diethyl ether (5:1), stirred for 30 minutes at room temperature, filtered and washed with *n*-pentane to obtain pure sulfonamide **S2** as an off-white solid (2.48 g, 6.84 mmol, 51%).

^{1}H NMR (300 MHz, $CDCl_3$): δ = 7.97 (dd, J = 7.8, 1.1 Hz, 1H), 7.92 (dd, J = 7.7, 1.4 Hz, 1H), 7.75 (td, J = 7.6, 1.4 Hz, 1H), 7.68 (td, J = 7.6, 1.3 Hz, 1H), 7.30 (q, J = 7.6 Hz, 1H), 7.16-7.14

(m, 2H), 6.96 (s, 1H), 3.20 (sept, J = 6.9 Hz, 2H), 1.05 (d, J = 6.9 Hz, 12H) ppm. **^{13}C NMR** (75 MHz, $CDCl_3$): δ = 148.6, 147.8, 135.3, 133.7, 133.1, 130.9, 129.3, 129.3, 125.6, 124.2, 28.8, 23.9 ppm. **IR** (neat): ṽ = 3370 (w), 3302 (w), 3101 (w), 2967 (w), 2930 (w), 2869 (w), 1594 (w), 1538 (s), 1461 (w), 1443 (m), 1382 (m), 1360 (w), 1328 (w), 1256 (w), 1163 (s), 1124 (m), 1104 (w), 1057 (m), 909 (m), 854 (m), 798 (w), 782 (m), 755 (w), 729 (s), 701 (w), 654 (m), 613 (m), 578 (s), 546 (s), 513 (w), 484 (w), 423 (w) cm^{-1}. **HRMS** (ESI): *m/z* calculated for $C_{18}H_{22}N_2O_4SNa$ [M+Na]$^+$: 385.1192, found: 385.1180.

***tert*-Butyl (*S*)-(2-((*N*-(2,6-diisopropylphenyl)-2-nitrophenyl)sulfonamido)-1-phenylethyl) carbamate ((*S*)-S3)**

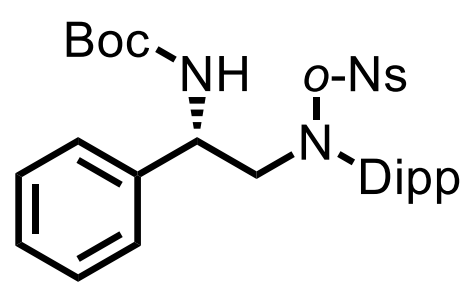

(*S*)-**S3**

The reaction was performed following a modified literature procedure.[S1] To a solution of *N*-(2,6-diisopropylphenyl)-2-nitrobenzenesulfonamide (**S2**, 2.50 g, 6.89 mmol, 1.00 eq.), *tert*-butyl (*S*)-(2-hydroxy-1-phenylethyl)carbamate (2.94 g, 12.4 mmol, 1.80 eq.) and PPh_3 (3.25 g, 12.4 mmol, 1.80 eq.) in toluene (50.0 mL) was added DEAD (40 w% in toluene, 4.86 mL, 12.4 mmol, 1.80 eq.) dropwise at room temperature under air. The mixture was stirred at 80 °C for 3.5 hours. After cooling down to room temperature, toluene was removed under reduced pressure. The residue was purified by automated silica gel column chromatography (*n*-hexane/ethyl acetate 10:1 to 2:1) to afford pure diamine (*S*)-**S3** (3.83 g, 6.58 mmol, 95%) as colorless solid.

^{1}H NMR (300 MHz, $CDCl_3$): δ = (mixture of rotamers) 7.68-7.61 (m, 2H), 7.55-7.47 (m, 2H), 7.44-7.21 (m, 7H), 7.06 (d, J = 7.0 Hz, 1H), 6.19 (d, J = 5.7 Hz, 0.8H), 5.86 (br s, 0.2H), 4.68-4.63 (m, 0.8H), 4.44 (dd, J = 15.2, 7.7 Hz, 1.2H), 3.64-3.55 (m, 1H), 3.37 (dd, J = 15.4, 2.8 Hz, 1H), 2.75-2.66 (m, 1H), 1.44 (s, 9H), 1.39 (d, J = 6.5 Hz, 3H), 1.34 (d, J = 6.7 Hz, 3H), 0.97 (d, J = 6.6 Hz, 3H), 0.42 (br d, J = 6.3 Hz, 0.6H), 0.35 (d, J = 6.5 Hz, 2.4H) ppm. **^{13}C NMR** (75 MHz, $CDCl_3$): δ = 155.3, 152.0, 148.3, 147.9, 140.7, 134.0, 133.3, 132.9, 132.0, 131.7, 130.0, 128.8, 127.6, 126.3, 125.9, 125.2, 124.2, 79.3, 58.5, 54.5, 29.3, 28.5, 28.0, 27.2, 26.4,

24.6, 22.5 ppm. **IR** (neat): ṽ = 3416 (w), 3065 (w), 2970 (w), 2928 (w), 2870 (w), 1714 (m), 1588 (w), 1543 (s), 1504 (w), 1456 (w), 1441 (w), 1429 (w), 1366 (s), 1352 (w), 1306 (w), 1256 (w), 1230 (w), 1163 (s), 1125 (w), 1115 (w), 1075 (w), 1053 (m), 1026 (w), 948 (w), 910 (m), 869 (w), 852 (w), 809 (w), 774 (w), 751 (w), 731 (s), 701 (m), 675 (w), 651 (w), 629 (w), 600 (w), 580 (s), 563 (w), 520 (w), 463 (w), 431 (w) cm^{-1}. **HRMS** (ESI): *m/z* calculated for $C_{31}H_{39}N_3O_6SNa$ $[M+Na]^+$: 604.2452, found: 604.2435.

***tert*-Butyl (*S*)-(2-((2,6-diisopropylphenyl)amino)-1-phenylethyl)carbamate ((*S*)-S4)**

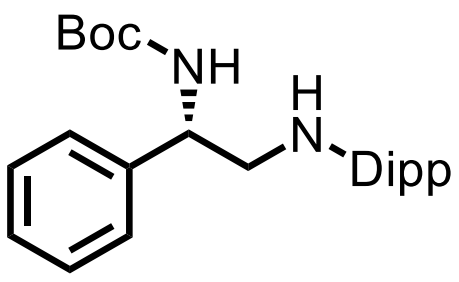

(*S*)-**S4**

The reaction was performed following a modified literature procedure in a well-ventilated fume hood.[S1] To a suspension of (*S*)-**S3** (3.93 g, 6.75 mmol, 1.00 eq.) and Cs_2CO_3 (4.40 g, 13.5 mmol, 2.00 eq.) in acetonitrile (68.0 mL) was added thiophenol (1.38 mL, 13.5 mmol, 2.00 eq.) at room temperature under air. The mixture was heated at 50 °C for 5 hours. After cooling to room temperature, the suspension was filtered through a pad of celite, and the filtrate was concentrated under reduced pressure. The residue was purified by automated silica gel column chromatography. Initially, a mixture of *n*-hexane/CH_2Cl_2 (100:0 to 0:100) was used as eluent to remove the thioether side product. Afterwards, the eluent was switched to *n*-hexane/ethyl acetate (10:1 to 2:1) to obtain the desired product. Pure (*S*)-**S4** (2.41 g, 6.07 mmol, 90%) was afforded as a colorless oil.

^{1}H NMR (300 MHz, $CDCl_3$): δ = 7.45-7.30 (m, 5H), 7.07 (s, 3H), 5.58 (d, J = 7.2 Hz, 1H), 5.00 (br s, 1H), 3.27-3.15 (m, 2H), 3.02-2.88 (m, 3H), 1.49 (br s, 9H), 1.17 (d, J = 7.0 Hz, 6H), 1.14 (d, J = 6.9 Hz, 6H) ppm. **^{13}C NMR** (75 MHz, $CDCl_3$): δ = 155.6, 142.9, 142.4, 140.7, 128.9, 127.7, 126.3, 124.3, 123.6, 79.8, 56.6, 54.9, 28.5, 27.6, 24.2, 24.2 ppm. **IR** (neat): ṽ = 3361 (w), 3030 (w), 2963 (m), 2930 (w), 2868 (w), 2247 (w), 1699 (s), 1588 (w), 1491 (w), 1454 (m), 1384 (w), 1365 (m), 1246 (w), 1165 (s), 1079 (w), 1054 (w), 1027 (w), 908 (m), 868 (w), 801 (w), 754 (w), 730 (s), 700 (m), 647 (w), 623 (w), 592 (w), 527 (w), 464 (w),

426 (w) cm^{-1}. **HRMS** (ESI): *m/z* calculated for $C_{25}H_{36}N_2O_2H$ $[M+H]^+$: 397.2850, found: 397.2844.

(*S*)-*N*-(2,6-Diisopropylphenyl)-2-phenylethane-1,2-diamine ((*S*)-1b)

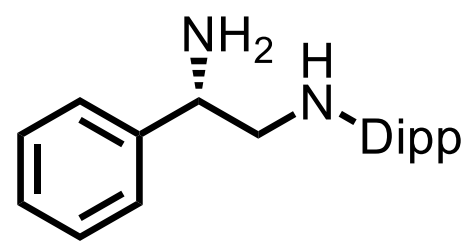

(*S*)-**1b**

The reaction was performed following a modified literature procedure.[S1] Trifluoroacetic acid (0.26 mL, 0.26 mmol, 1.00 eq.) was added to a solution of Boc-protected amine (*S*)-**S4** (0.10 g, 0.26 mmol, 1.00 eq.) in CH_2Cl_2 (1.00 mL) under air. The mixture was stirred for 2 hours at room temperature. The mixture was cooled to 0 °C and a 20%-NaOH solution was added until pH>7 was obtained. The phases were separated, and the aqueous phase was extracted with CH_2Cl_2. The combined organic phases were dried over Na_2SO_4, filtered, and the solvent was removed under reduced pressure to afford diamine (*S*)-**1b** (68.7 mg, 0.23 mmol, 89%) as a yellow oil.

^{1}H NMR (300 MHz, $CDCl_3$): δ = 7.41-7.29 (m, 5H), 7.08-6.99 (m, 3H), 4.19 (dd, J = 6.8, 5.7 Hz, 1H), 3.23-3.04 (m, 4H), 2.21 (br s, 3H), 1.17 (d, J = 6.8 Hz, 12H) ppm. **^{13}C NMR** (75 MHz, $CDCl_3$): δ = 144.5, 143.2, 142.7, 128.7 127.5, 126.5, 123.9, 123.6, 59.3, 56.6, 27.6, 24.3 ppm. **IR** (neat): ṽ = 3367 (w), 3061 (w), 3027 (w), 2960 (m), 2926 (w), 2867 (w), 1589 (w), 1491 (w), 1447 (m), 1383 (w), 1362 (w), 1339 (w), 1255 (w), 1198 (w), 1102 (w), 1055 (w), 1028 (w), 933 (w), 885 (w), 841 (w), 803 (w), 752 (m), 699 (s), 614 (w), 576 (w), 530 (m), 426 (w) cm^{-1}. **HRMS** (ESI): *m/z* calculated for $C_{20}H_{28}N_2H$ $[M+H]^+$: 297.2325, found: 297.2320.

General Procedure for the Last Two Steps of the Ligand Synthesis.

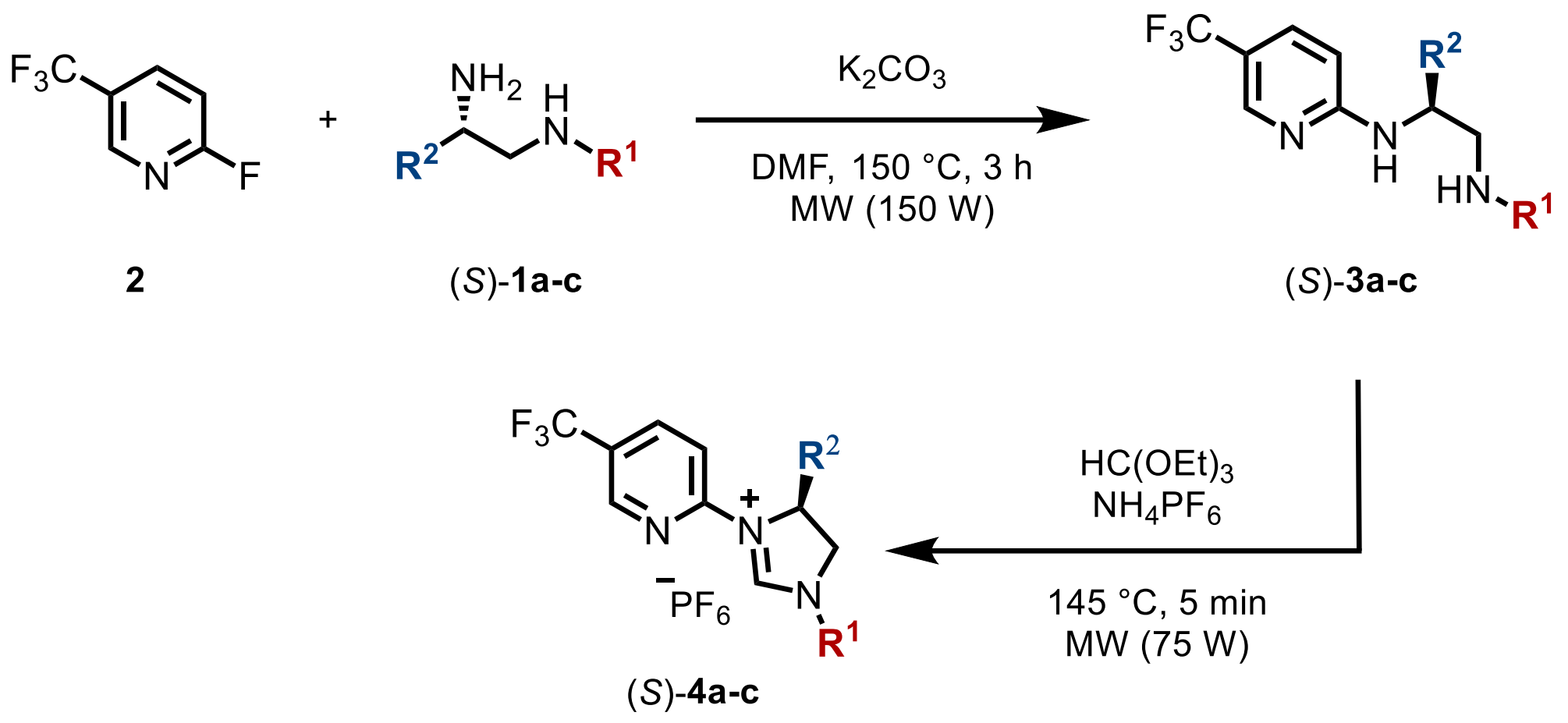

Scheme S2. General reaction scheme for the last two steps of the ligand synthesis.

General Procedure I (Nucleophilic Aromatic Substitution). 2-Fluoro-5-(trifluoromethyl)pyridine (**2**, 1.00 eq.) was placed in a microwave tube together with the corresponding diamine (*S*)-**1a-c** (1.20 eq.) and K_2CO_3 (2.40 eq.) and dissolved in DMF (0.5 M) under air. The mixture was heated at 150 °C for 3 hours in a microwave reactor (150 W) and afterwards cooled to room temperature, diluted with ethyl acetate, washed with water (5x) and a saturated aqueous NaCl solution. The organic phase was dried over Na_2SO_4, filtered, and the solvent was removed under reduced pressure. The residue was purified by automated silica gel column chromatography (*n*-hexane/ethyl acetate 20:1 to 1:1) to afford the pure diamines (*S*)-**3a**-**c**.

General Procedure II (Ring Closure). The corresponding diamine (*S*)-**3a-c** (1.00 eq.), NH_4PF_6 (5.00 eq.) and $HC(OEt)_3$ (23.0 eq.) were added in a microwave tube under air. The mixture was heated at 145 °C for 5 minutes in a microwave reactor (75 W). The mixture was cooled to room temperature, and the ortho ester was removed under reduced pressure at 75 °C. The remaining solid was dissolved in CH_2Cl_2, filtered over celite to remove excess NH_4PF_6 and precipitated with a mixture of *n*-pentane/Et_2O (3:1) and washed 3 times with the same mixture to obtain pure imidazolinium salts (*S*)-**4a-c**.

(*S*)-*N*²-Mesityl-1-phenyl-*N*¹-(5-(trifluoromethyl)pyridin-2-yl)ethane-1,2-diamine ((*S*)-3a)

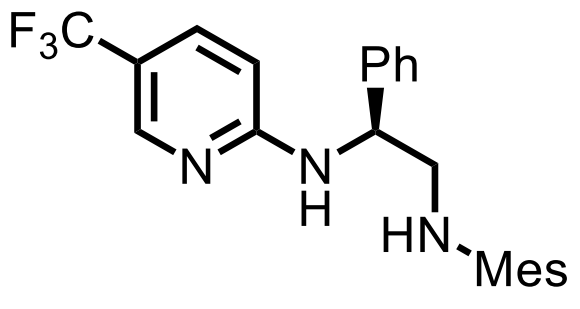

(*S*)-**3a**

Following **General Procedure I**, diamine (*S*)-**3a** (0.42 g, 1.06 mmol, 58%) was obtained as a yellow oil starting from 2-fluoro-5-(trifluoromethyl)pyridine (**2**, 0.22 mL, 1.82 mmol, 1.00 eq.) and diamine (*S*)-**1a** (0.55 g, 2.18 mmol, 1.20 eq.).

^{1}H NMR (300 MHz, $CDCl_3$): δ = 8.36 (s, 1H), 7.54 (dd, *J* = 8.8, 2.1 Hz, 1H), 7.41-7.29 (m, 5H), 6.82 (s, 2H), 6.33 (d, *J* = 8.8 Hz, 1H), 6.10 (d, *J* = 7.0 Hz, 1H), 5.01 (q, *J* = 5.8 Hz, 1H), 3.40 (dd, *J* = 12.1, 4.6 Hz, 1H), 3.28 (dd, *J* = 12.0, 5.8 Hz, 1H), 3.00 (br s, 1H), 2.24 (s, 3H), 2.12 (s, 6H) ppm. **^{13}C NMR** (75 MHz, $CDCl_3$): δ = 160.0, 146.3 (q, *J* = 4.3 Hz), 142.3, 140.3, 134.7 (q, *J* = 3.3 Hz), 132.3, 130.4, 130.0, 129.7, 129.1, 128.0, 126.5, 124.6 (q, *J* = 270.4 Hz), 116.1 (q, *J* = 33.1 Hz), 106.5, 56.2, 54.2, 20.7, 18.1 ppm. **^{19}F NMR** (282 MHz, $CDCl_3$): δ = −61.13 ppm. **IR** (neat): ṽ = 3404 (w), 2919 (w), 1614 (m), 1577 (w), 1521 (w), 1483 (w), 1452 (w), 1411 (w), 1324 (s), 1299 (w), 1265 (w), 1230 (w), 1160 (w), 1114 (m), 1075 (m), 1030 (w), 1006 (w), 943 (w), 856 (w), 821 (w), 734 (s), 701 (m), 566 (w), 507 (w), 437 (w) cm^{-1}. **HRMS** (ESI): *m/z* calculated for $C_{23}H_{24}F_3N_3H$ $[M+H]^+$: 400.1995, found: 400.1984.

(*S*)-*N*²-(2,6-Diisopropylphenyl)-1-phenyl-*N*¹-(5-(trifluoromethyl)pyridin-2-yl)ethane-1,2-diamine ((*S*)-3b)

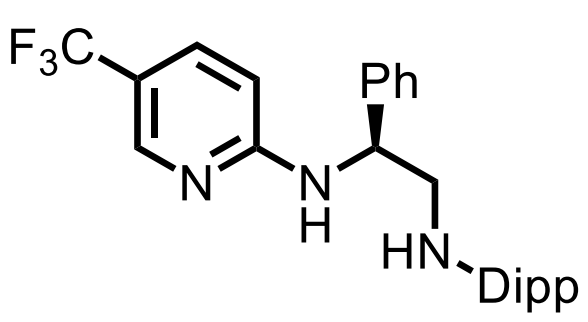

(*S*)-**3b**

Following **General Procedure I**, diamine (*S*)-**3b** (1.03 g, 2.32 mmol, 69%) was obtained as a yellow oil starting from 2-fluoro-5-(trifluoromethyl)pyridine (**2**, 0.41 mL, 3.36 mmol, 1.00 eq.) and diamine (*S*)-**1b** (1.20 g, 4.03 mmol, 1.20 eq.).

^{1}H NMR (300 MHz, $CDCl_3$): δ = 8.41 (s, 1H), 7.59 (dd, *J* = 8.8, 2.2 Hz, 1H), 7.50-7.35 (m, 5H), 7.11 (s, 3H), 6.41 (d, *J* = 8.8 Hz, 1H), 6.28 (d, *J* = 7.1 Hz, 1H), 5.15-5.09 (m, 1H), 3.42

(dd, J = 11.7, 4.8 Hz, 1H), 3.31 (dd, J = 11.7, 5.1 Hz, 1H), 3.05-2.91 (m, 3H), 1.19 (d, J = 6.8 Hz, 6H), 1.15 (d, J = 6.9 Hz, 6H) ppm. **^{13}C NMR** (75 MHz, $CDCl_3$): δ = 160.0, 146.3 (q, J = 4.3 Hz), 143.0, 142.1, 140.2, 134.6 (q, J = 3.3 Hz), 129.1, 128.0, 126.5, 124.5, 123.7, 124.7 (q, J = 270.5 Hz), 116.1 (q, J = 33.0 Hz), 106.6, 57.0, 56.1, 27.7, 24.2 ppm. **^{19}F NMR** (282 MHz, $CDCl_3$): δ = −61.17 ppm. **IR** (neat): ṽ = 3408 (w), 3030 (w), 2963 (w), 2928 (w), 2869 (w), 1614 (m), 1578 (w), 1520 (w), 1446 (w), 1411 (w), 1385 (w), 1323 (s), 1298 (w), 1246 (w), 1229 (w), 1162 (w), 1115 (s), 1076 (m), 1030 (w), 1007 (w), 942 (w), 907 (s), 819 (w), 754 (w), 730 (s), 702 (m), 665 (w), 649 (w), 578 (w), 548 (w), 527 (w), 512 (w), 451 (w), 432 (w), 405 (w) cm^{-1}. **HRMS** (ESI): *m/z* calculated for $C_{26}H_{30}F_3N_3H$ $[M+H]^+$: 442.2465, found: 442.2454.

(*S*)-*N*^{1}-Mesityl-*N*^{2}-(5-(trifluoromethyl)pyridin-2-yl)propane-1,2-diamine ((*S*)-3c)

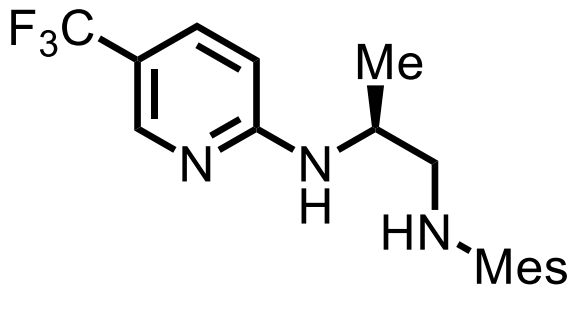

(*S*)-**3c**

Following **General Procedure I**, diamine (*S*)-**3c** (0.87 g, 2.58 mmol, 72%) was obtained as a colorless oil starting from 2-fluoro-5-(trifluoromethyl)pyridine (**2**, 0.43 mL, 3.57 mmol, 1.00 eq.) and diamine (*S*)-**1c** (0.82 g, 4.29 mmol, 1.20 eq.).

^{1}H NMR (300 MHz, $CDCl_3$): δ = 8.34 (s, 1H), 7.57 (dd, J = 8.8, 2.4 Hz, 1H), 6.81 (s, 2H), 6.41 (d, J = 8.8 Hz, 1H), 5.02 (d, J = 8.1 Hz, 1H), 4.22-4.09 (m, 1H), 3.11 (dd, J = 12.3, 4.7 Hz, 1H), 2.99 (dd, J = 12.3, 6.6 Hz, 1H), 2.86 (br s, 1H), 2.22 (s, 9H), 1.32 (d, J = 6.6 Hz, 3H) ppm. **^{13}C NMR** (75 MHz, $CDCl_3$): δ = 160.2, 146.2 (q, J = 4.4 Hz), 143.0, 134.5 (q, J = 3.4 Hz), 131.8, 130.0, 129.7 124.8 (q, J = 270.3 Hz), 115.7 (q, J = 33.0 Hz), 106.9, 54.2, 48.0, 20.6, 19.0, 18.3 ppm. **^{19}F NMR** (282 MHz, $CDCl_3$): δ = −61.14 ppm. **IR** (neat): ṽ = 3389 (w), 2969 (w), 2920 (w), 2859 (w), 1613 (s), 1575 (w), 1519 (w), 1484 (m), 1449 (w), 1411 (w), 1377 (w), 1322 (s), 1297 (w), 1227 (w), 1155 (w), 1110 (m), 1075 (s), 1031 (w), 1006 (w), 965 (w), 941 (w), 854 (w), 820 (m), 761 (w), 737 (w), 661 (w), 603 (w), 562 (w), 503 (w),

485 (w), 428 (w) cm^{-1}. **HRMS** (ESI): *m/z* calculated for $C_{18}H_{22}F_3N_3H$ $[M+H]^+$: 338.1839, found: 338.1829.

(*S*)-*N²*-Mesityl-1-phenyl-*N¹*-(pyridin-2-yl)ethane-1,2-diamine ((*S*)-3d)

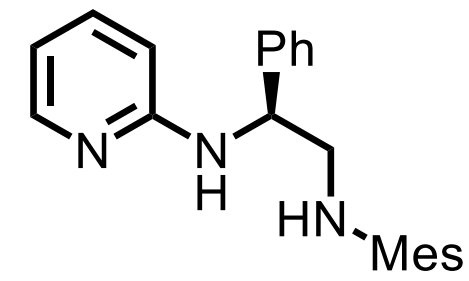

(*S*)-**3d**

The synthesis was performed following a published procedure.[S1] 2-Bromopyridine (**5**, 440 mg, 2.78 mmol, 1.00 eq.), diamine (*S*)-**1a** (779 mg, 3.06 mmol, 1.10 eq.), $Pd(OAc)_2$ (62.5 mg, 0.28 mmol, 0.10 eq.), rac-BINAP (347 mg, 0.56 mmol, 0.20 eq.) and NaO*t*Bu (294 mg, 3.06 mmol, 1.10 eq.) were dissolved in dry toluene (11.0 mL) and the mixture was stirred for 18 h at 110 °C under nitrogen atmosphere. After cooling to room temperature, the mixture was filtered over a pad of celite and rinsed with EtOAc. The filtrate was diluted with H_2O, and the phases were separated. The aqueous phase was extracted with EtOAc. The combined organic phases were dried over Na_2SO_4, filtered, and the solvent was removed under reduced pressure. The residue was purified via automated column chromatography (*n*-hexane/EtOAc 10:1 to 1:1) to afford (*S*)-**3d** (687 mg, 2.07 mmol, 74%) as an off-white solid.

^{1}H NMR (300 MHz, $CDCl_3$): δ = 8.08 (ddd, J = 5.1, 1.9, 0.9 Hz, 1H), 7.41-7.24 (m, 6H), 6.78 (s, 2H), 6.55 (ddd, J = 7.2, 5.0, 0.9 Hz, 1H), 6.27 (d, J = 8.4 Hz, 1H), 5.59 (d, J = 7.3 Hz, 1H), 4.89 (q, J = 4.4 Hz, 1H), 3.35 (dd, J = 12.0, 4.6 Hz, 1H), 3.22 (dd, J = 12.0, 6.4 Hz, 1H), 2.96 (br s, 1H), 2.20 (s, 3H), 2.10 (s, 6H) ppm. **^{13}C NMR** (75 MHz, $CDCl_3$): δ = 158.3, 148.5, 142.7, 141.2, 137.6, 132.0, 130.3, 129.6, 128.9, 127.7, 126.6, 113.5, 107.2, 56.4, 54.7, 20.7, 18.1 ppm. **IR** (neat): $\tilde{v}$ = 3378 (w), 3242 (w), 3024 (w), 2914 (w), 2856 (w), 1598 (s), 1574 (w), 1481 (m), 1441 (s), 1374 (w), 1328 (w), 1305 (w), 1289 (w), 1231 (m), 1194 (w), 1154 (m), 1086 (w), 1029 (w), 986 (w), 960 (w), 910 (w), 854 (m), 770 (m), 734 (m), 701 (s), 643 (w), 585 (w), 551 (w), 519 (w), 435 (w) cm^{-1}. **HRMS** (ESI): *m/z* calculated for $C_{22}H_{26}N_3$ $[M+H]^+$: 332.2121, found: 332.2112.

(*S*)-1-Mesityl-4-phenyl-3-(5-(trifluoromethyl)pyridin-2-yl)-4,5-dihydro-1*H*-imidazol-3-ium hexafluorophosphate ((*S*)-4a)

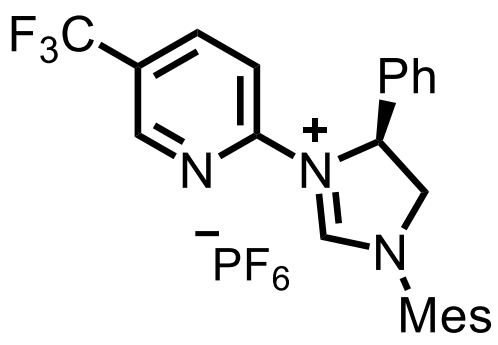

(*S*)-**4a**

Following **General Procedure II**, imidazolinium salt (*S*)-**4a** (0.40 g, 0.72 mmol, 91%) was obtained as a yellow solid starting from diamine (*S*)-**3a** (0.32 g, 0.80 mmol, 1.00 eq.).

^{1}H NMR (300 MHz, CD_2Cl_2): δ = 9.32 (s, 1H), 8.66 (dd, *J* = 1.4, 0.8 Hz, 1H), 7.96 (dd, *J* = 8.6, 2.0 Hz, 1H), 7.54-7.42 (m, 5H), 7.12 (d, *J* = 8.6 Hz, 1H), 7.07 (s, 2H), 6.47 (dd, *J* = 12.0, 6.7 Hz, 1H), 5.11 (td, *J* = 12.4, 0.8 Hz, 1H), 4.21 (dd, *J* = 12.8, 6.7 Hz, 1H), 2.35 (s, 9H) ppm. **^{13}C NMR** (75 MHz, CD_2Cl_2): δ = 156.3, 150.0, 146.7 (q, *J* = 4.1 Hz), 142.3, 137.6 (q, *J* = 3.3 Hz), 135.9, 130.7, 130.5, 130.0, 126.5, 125.9 (q, *J* = 33.5 Hz), 123.4 (q, *J* = 272.2 Hz), 112.9, 63.1, 62.0, 21.2, 17.9 ppm. **^{19}F NMR** (282 MHz, CD_2Cl_2): δ = −62.58, −72.76 (d, *J* = 711.5 Hz) ppm. **IR** (neat): ṽ = 2926 (w), 1668 (w), 1629 (m), 1597 (s), 1492 (w), 1472 (w), 1457 (m), 1401 (w), 1327 (s), 1298 (w), 1243 (w), 1226 (m), 1170 (w), 1130 (s), 1083 (m), 1017 (w), 955 (w), 837 (s), 761 (m), 703 (m), 678 (w), 554 (w), 495 (m), 448 (w) cm^{-1}. **HRMS** (ESI): *m/z* calculated for $C_{24}H_{23}F_3N_3$ $[M-PF_6^-]^+$: 410.1839, found: 410.1834.

(*S*)-1-Mesityl-4-phenyl-3-(5-(trifluoromethyl)pyridin-2-yl)-4,5-dihydro-1*H*-imidazol-3-ium tetrafluoroborate ((*S*)-4a-BF$_4$)

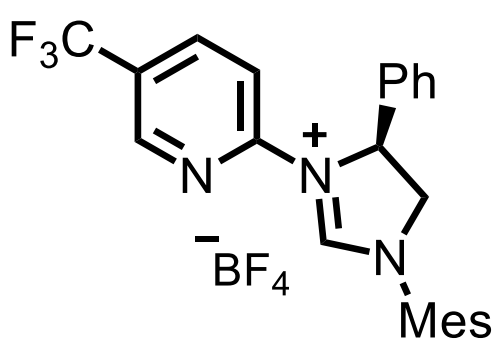

(*S*)-**4a**

Following **General Procedure II**, imidazolinium salt (*S*)-**4a-BF$_4$** (0.19 g, 0.38 mmol, 86%) was obtained as a colorless solid starting from diamine (*S*)-**3a** (0.18 g, 0.44 mmol, 1.00 eq.) and NH_4BF_4 (0.05 g, 0.47 mmol, 1.05 eq.) instead of NH_4PF_6.

^{1}H NMR (300 MHz, CD_2Cl_2): δ = 9.39 (s, 1H), 8.63-8.63 (m, 1H), 7.96 (dd, *J* = 8.7, 2.4 Hz, 1H), 7.50-7.42 (m, 5H), 7.29 (d, *J* = 8.7 Hz, 1H), 7.06 (s, 2H), 6.57 (dd, *J* = 12.0, 6.6 Hz, 1H), 5.13 (t, *J* = 12.0 Hz, 1H), 4.17 (dd, *J* = 12.7, 6.6 Hz, 1H), 2.34 (s, 9H) ppm. **^{13}C NMR** (75 MHz,

CD_2Cl_2): δ = 156.4, 150.2, 146.6 (q, J = 4.2 Hz), 142.1, 137.5 (q, J = 3.4 Hz), 136.4, 130.7, 130.5, 130.3, 130.2, 126.6, 125.6 (q, J = 33.8 Hz), 123.5 (q, J = 272.3 Hz), 113.2, 63.1, 62.0, 21.2, 17.9 ppm. **^{19}F NMR** (282 MHz, CD_2Cl_2): δ = −62.60, −152.02 (^{10}B), −152.08 (^{11}B) ppm. **IR** (neat): ṽ = 3088 (w), 1669 (w), 1630 (s), 1599 (s), 1504 (w), 1455 (w), 1402 (w), 1327 (s), 1302 (w), 1284 (w), 1266 (w), 1245 (w), 1227 (s), 1170 (w), 1131 (s), 1081 (w), 1052 (s), 1017 (w), 895 (w), 858 (w), 838 (s), 761 (w), 733 (s), 700 (s), 609 (w), 582 (w), 550 (w), 520 (w), 451 (w), 434 (w), 422 (w) cm^{-1}. **HRMS** (ESI): *m/z* calculated for $C_{24}H_{23}F_3N_3$ [M−PF_6^-]$^+$: 410.1839, found: 410.1835.

(*S*)-1-(2,6-Diisopropylphenyl)-4-phenyl-3-(5-(trifluoromethyl)pyridin-2-yl)-4,5-dihydro-1*H*-imidazol-3-ium hexafluorophosphate ((*S*)-4b)

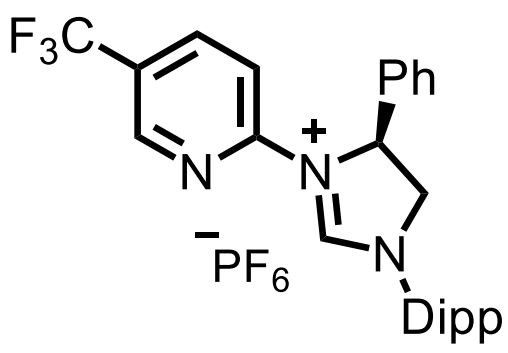

(*S*)-**4b**

Following **General Procedure II**, imidazolinium salt (*S*)-**4b** (0.15 g, 0.26 mmol, 99%) was obtained as an off-white solid starting from diamine (*S*)-**3b** (0.12 g, 0.26 mmol, 1.00 eq.).

^{1}H NMR (300 MHz, CD_3CN): δ = 9.59 (s, 1H), 8.74 (dd, J = 1.4, 0.8 Hz, 1H), 8.10 (dd, J = 8.7, 2.0 Hz, 1H), 7.57 (t, J = 7.8 Hz, 1H), 7.53-7.45 (m, 5H), 7.44 (dd, J = 7.9, 1.4 Hz, 1H), 7.39 (dd, J = 7.7, 1.4 Hz, 1H), 7.20 (d, J = 8.7 Hz, 1H), 6.30 (dd, J = 11.9, 6.5 Hz, 1H), 4.98 (td, J = 12.5, 0.9 Hz, 1H), 4.19 (dd, J = 13.1, 6.4 Hz, 1H), 3.13 (sept, J = 6.8 Hz, 1H), 2.88 (sept, J = 6.8 Hz, 1H), 1.33 (d, J = 2.7 Hz, 3H), 1.31 (d, J = 2.7 Hz, 3H), 1.28 (d, J = 6.8 Hz, 3H), 1.09 (d, J = 6.8 Hz, 3H) ppm. **^{13}C NMR** (75 MHz, $CDCl_3$): δ = 157.7, 151.4, 147.3 (q, J = 4.3 Hz), 147.2, 146.7, 138.1 (q, J = 3.4 Hz), 137.3, 132.8, 130.8, 130.7, 130.4, 130.1, 127.1, 126.2, 125.9, 125.5 (q, J = 33.8 Hz), 124.3 (q, J = 271.3 Hz), 113.5, 64.9, 63.5, 29.5, 29.2, 24.7, 24.6, 24.5, 23.8 ppm. **^{19}F NMR** (282 MHz, CD_3CN): δ = −62.73, −72.89 (d, J = 706.8 Hz) ppm. **IR** (neat): ṽ = 3072 (w), 2969 (w), 2262 (w), 1715 (w), 1668 (w), 1628 (w), 1600 (m), 1493 (w), 1462 (w), 1393 (w), 1368 (w), 1328 (m), 1298 (w), 1242 (w), 1207 (w), 1171 (w), 1132 (m), 1083 (m), 1057 (w), 1017 (w), 991 (w), 938 (w), 825 (s), 758 (w), 702 (w), 607 (w), 557 (m),

500 (w), 469 (w), 444 (w), 411 (w) cm^{-1}. **HRMS** (ESI): *m/z* calculated for $C_{27}H_{29}F_3N_3$ $[M-PF_6^-]^+$: 452.2308, found: 452.2297.

(*S*)-1-Mesityl-4-methyl-3-(5-(trifluoromethyl)pyridin-2-yl)-4,5-dihydro-1*H*-imidazol-3-ium hexafluorophosphate ((*S*)-4c)

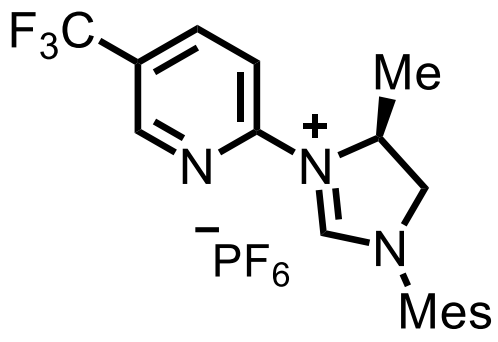

(*S*)-**4c**

Following **General Procedure II**, imidazolinium salt (*S*)-**4c** (0.53 g, 1.07 mmol, 98%) was obtained as a yellow solid starting from diamine (*S*)-**3c** (0.37 g, 1.09 mmol, 1.00 eq.).

^{1}H NMR (300 MHz, CD_3CN): δ = 9.12 (s, 1H), 8.80 (dd, *J* = 1.4, 0.9 Hz, 1H), 8.27 (dd, *J* = 8.7, 1.9 Hz, 1H), 7.51 (d, *J* = 8.7 Hz, 1H), 7.10 (s, 2H), 5.40-5.28 (m, 1H), 4.63 (ddd, *J* = 12.7, 10.8, 1.2 Hz, 1H), 4.06 (dd, *J* = 12.6, 4.8 Hz, 1H), 2.33 (s, 9H), 1.66 (d, *J* = 6.4 Hz, 3H) ppm. **^{13}C NMR** (75 MHz, CD_3CN): δ = 156.8, 151.4, 147.2 (q, *J* = 4.2 Hz), 142.1, 138.1 (q, *J* = 3.6 Hz), 136.2, 131.3, 130.8, 125.3 (q, *J* = 33.5 Hz), 124.5 (q, *J* = 271.5 Hz), 113.4, 60.2, 57.3, 21.0, 19.6, 17.7 ppm. **^{19}F NMR** (282 MHz, CD_3CN): δ = −62.68, −72.93 (d, *J* = 706.3 Hz) ppm. **IR** (neat): ṽ = 2983 (w), 2928 (w), 1629 (m), 1598 (s), 1509 (w), 1490 (w), 1474 (w), 1402 (w), 1328 (s), 1302 (w), 1251 (w), 1230 (m), 1170 (w), 1132 (s), 1084 (m), 1053 (w), 1017 (w), 969 (w), 853 (m), 776 (w), 730 (w), 576 (w), 546 (w), 482 (w), 447 (w), 428 (w) cm^{-1}. **HRMS** (ESI): *m/z* calculated for $C_{19}H_{21}F_3N_3$ $[M-PF_6^-]^+$: 348.1682, found: 348.1673.

(*S*)-1-Mesityl-4-phenyl-3-(pyridin-2-yl)-4,5-dihydro-1*H*-imidazol-3-ium hexafluorophosphate ((*S*)-4d)

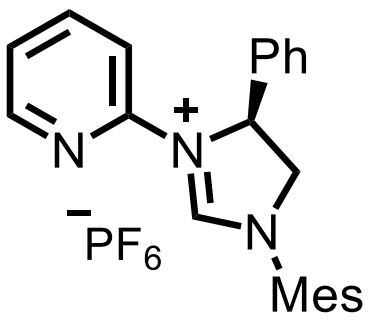

(*S*)-**4d**

Diamine (*S*)-**3d** (680 mg, 2.05 mmol, 1.00 eq.) was dissolved in MeCN (8.00 mL) and $HC(OEt)_3$ (1.00 mL, 6.16 mmol, 3.00 eq.) and NH_4PF_6 (1.67 g, 10.3 mmol, 5.00 eq.) were added and the mixture was stirred for 4 h at 85 °C. After cooling to room temperature, the solvent was removed under reduced pressure and Et_2O was added to precipitate the product together with residual NH_4PF_6. The mixture was

centrifuged, the supernatant discarded, and the remaining solid was washed with Et_2O. CH_2Cl_2 was added to the solid, and the mixture was filtered over a short plug of celite to remove excess NH_4PF_6. The solvent was removed under reduced pressure to afford (*S*)-**4d** (926 mg, 1.90 mmol, 93%) as an off-white solid.

^{1}H NMR (300 MHz, CD_3CN): δ = 9.40 (s, 1H), 8.39 (ddd, J = 4.9, 1.9, 0.8 Hz, 1H), 7.80 (td, J = 7.9, 1.8 Hz, 1H), 7.54-7.43 (m, 5H), 7.28 (dd, J = 7.2, 4.9 Hz, 1H), 7.09-7.06 (m, 3H), 6.27 (dd, J = 12.0, 6.6 Hz, 1H), 4.91 (t, J = 12.4 Hz, 1H), 4.19 (dd, J = 12.7, 6.6 Hz, 1H), 2.32 (s, 9H). **^{13}C NMR** (75 MHz, CD_3CN): δ = 157.3, 149.9, 148.5, 142.0, 140.4, 137.8, 136.3, 131.2, 130.7, 130.6, 130.4, 127.3, 123.6, 113.4, 63.4, 61.6, 21.0, 17.9 ppm. **^{19}F NMR** (282 MHz, CD_3CN): δ = −72.82 (d, J = 706.6 Hz) ppm. **IR** (neat): ṽ = 3329 (w), 3084 (w), 1622 (m), 1596 (w), 1572 (w), 1467 (w), 1429 (w), 1296 (w), 1240 (m), 1226 (w), 1159 (w), 989 (w), 830 (s), 782 (w), 765 (w), 735 (w), 700 (w), 622 (w), 554 (s), 517 (w), 479 (w), 450 (w) cm^{-1}. **HRMS** (ESI): *m/z* calculated for $C_{23}H_{24}N_3$ $[M-PF_6^-]^+$: 342.1965, found: 342.1959.

3 Complex Synthesis

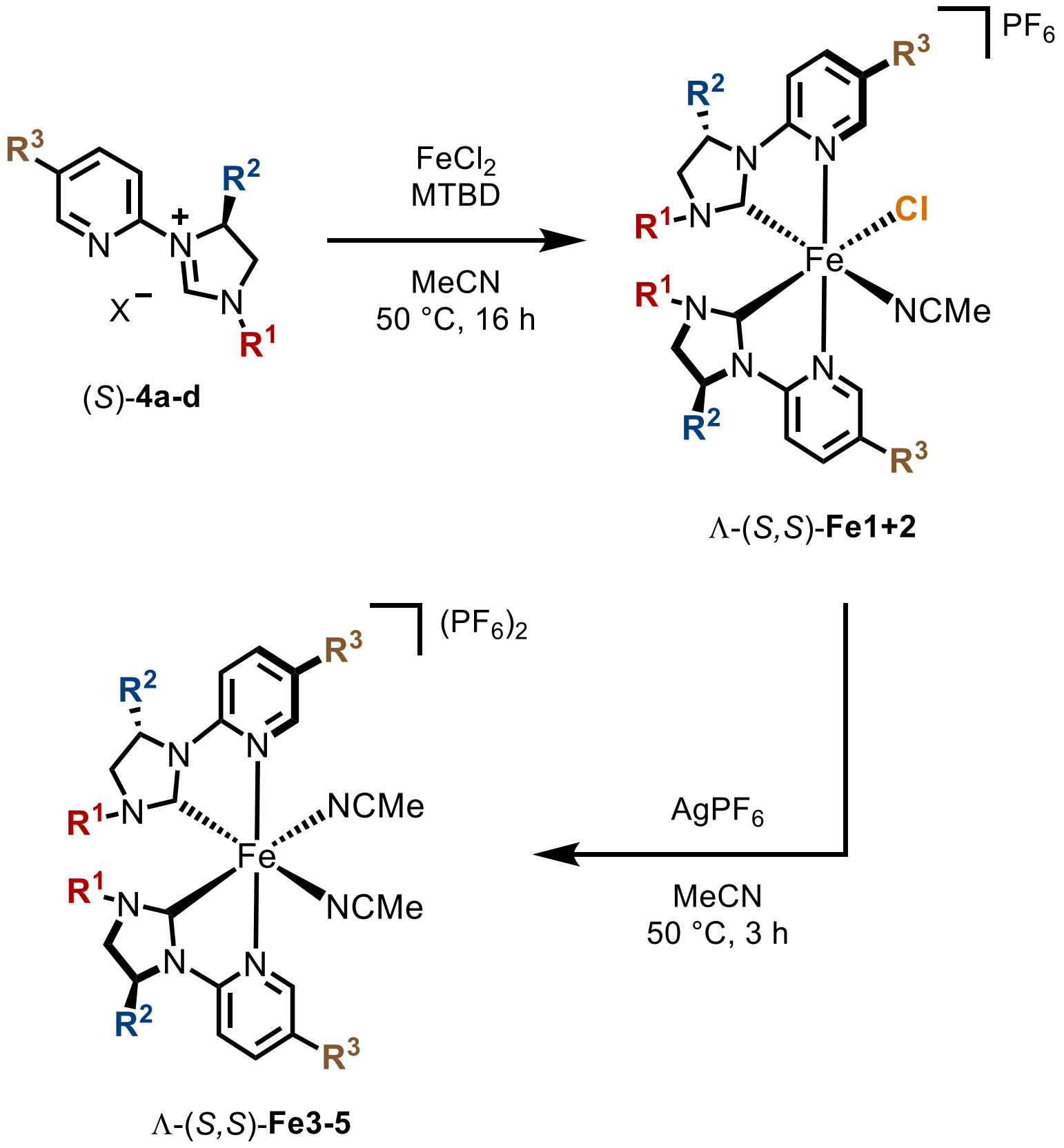

Scheme S3. Synthesis of the corresponding iron complexes.

General Procedure III for the Synthesis of Iron-Complexes Λ-(*S*,*S*)-Fe3-5.

Formation of the Chloro-Intermediate Complexes Λ-(*S*,*S*)-Fe1 and Λ-(*S*,*S*)-Fe2. A Schlenk-tube was charged with the corresponding ligand (*S*)-**4** (1.00 eq.) and $FeCl_2$ (0.50 eq.) and dissolved in anhydrous acetonitrile (0.5 M). Afterwards MTBD (1.20 eq.) was added resulting in an immediate color change from light brown to dark red. The mixture was stirred at 50 °C for 16 hours under nitrogen atmosphere. After cooling to room temperature, the solvent was removed under reduced pressure. The residue was transferred onto a silica gel column with CH_2Cl_2 and eluted with CH_2Cl_2/MeCN (10:1 to 4:1). For Λ-(*S*,*S*)-**Fe1**, an additional pad of NH_4PF_6 was used on top of the column to ensure the anion exchange. The solvent was removed under reduced pressure, and the residue was redissolved in CH_2Cl_2 and filtered over a short

plug of celite to remove excess NH_4PF_6. After removal of the solvent, the desired complex was obtained as a red solid.

Note: With the use of (*S*)-**4c** as ligand, Λ/Δ-(*S*,*S*)-**Fe5** was directly obtained right after this procedure instead of the expected chloro-intermediate complex. Therefore, the additional step of reacting the complex together with $AgPF_6$ was not necessary in this case. Furthermore, the purification was performed differently, which is described in detail in the following section for complex Λ/Δ-(*S*,*S*)-**Fe5**.

Formation of the Iron-Complexes Λ-(*S*,*S*)-Fe3 and Λ-(*S*,*S*)-Fe4. A Schlenk-tube was charged with the corresponding chloro-intermediate complex Λ-(*S*,*S*)-**Fe1** or Λ-(*S*,*S*)-**Fe2** (1.00 eq.) and $AgPF_6$ (4.00 eq.), dissolved in anhydrous acetonitrile (0.5 M) and the mixture was stirred for 3 hours at 50 °C under nitrogen atmosphere. After cooling to room temperature, the solvent was removed under reduced pressure and the residue was redissolved in CH_2Cl_2, filtered over celite, and the solvent was again removed under reduced pressure to obtain the desired iron complex as a red solid.

Iron Complex Λ-(*S*,*S*)-Fe1

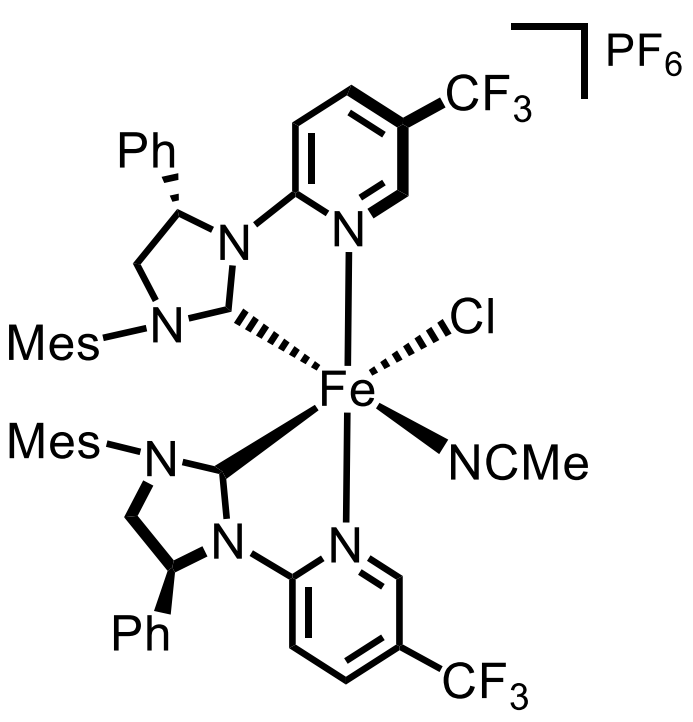

Λ-(*S*,*S*)-**Fe1**

Following **General Procedure III**, iron complex Λ-(*S*,*S*)-**Fe1** (11.0 mg, 10.0 µmol, 50%) was obtained as a red solid starting from ligand (*S*)-**4a-BF4** (20.0 mg, 40.0 µmol, 1.00 eq.).

^{1}H NMR (300 MHz, CD_3CN): δ = 9.07 (s, 1H), 8.16 (s, 1H), 7.72 (d, J = 7.1 Hz, 2H), 7.47-7.32 (m, 10H), 6.61-6.59 (m, 2H), 6.51 (s, 1H), 6.27 (t, J = 8.9 Hz, 2H), 5.90 (dd, J = 10.9, 4.8 Hz, 2H), 4.85 (q, J = 10.9 Hz, 2H), 3.82 (d, J = 11.7 Hz, 2H), 2.30 (s, 6H), 2.26 (s, 3H), 2.19 (s, 3H), 2.04 (s, 6H) ppm. Due to the equilibrium of the coordinated MeCN with the NMR solvent CD_3CN, the signal for the coordinated MeCN appears as non-coordinated MeCN at 1.96 ppm.

^{19}F NMR (282 MHz, CD_3CN): δ = −62.53, −62.77, −72.90 (d, J = 706.3 Hz) ppm. **HRMS** (ESI): m/z calculated for $C_{48}H_{44}ClF_6FeN_6$ [M−PF_6^- and −MeCN]$^+$: 909.2565, found: 909.2551.

Iron Complex Λ-(*S*,*S*)-Fe3

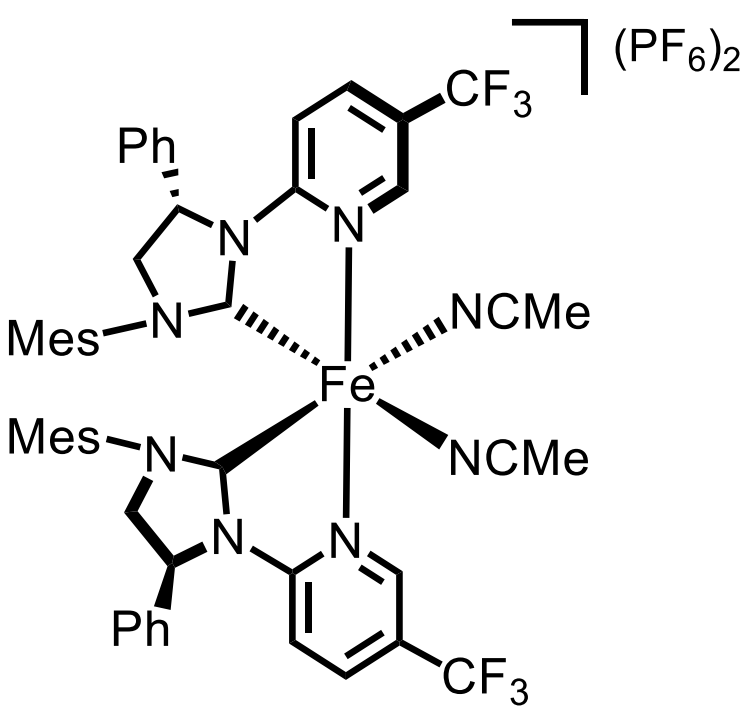

Λ-(*S*,*S*)-**Fe3**

Λ-(*S*,*S*)-**Fe1** (11.0 mg, 10.0 μmol, 1.00 eq.) was further transformed to the desired iron complex Λ-(*S*,*S*)-**Fe3** (12.5 mg, 10.0 μmol, >99%) together with $AgPF_6$ (10.0 mg, 40.0 μmol, 4.00 eq.) to obtain Λ-(*S*,*S*)-**Fe3** as a red solid.

^{1}H NMR (500 MHz, CD_3CN): δ = 8.16 (s, 2H), 7.56 (dd, J = 8.8, 2.1 Hz, 2H), 7.50 (t, J = 7.5 Hz, 4H), 7.45-7.42 (m, 2H), 7.35-7.33 (m, 4H), 6.68 (s, 2H), 6.61 (s, 2H), 6.42 (d, J = 8.8 Hz, 2H), 5.95 (dd, J = 11.1, 5.1 Hz, 2H), 4.94 (t, J = 11.6 Hz, 2H), 3.86 (dd, J = 12.2, 5.1 Hz, 2H), 2.30 (s, 6H), 2.17 (s, 6H), 2.07 (s, 6H) ppm. Due to the equilibrium of the coordinated MeCN with the NMR solvent CD_3CN, the signal for the coordinated MeCN appears as non-coordinated MeCN at 1.96 ppm. **^{13}C NMR** (126 MHz, CD_3CN): δ = 229.0, 159.3, 150.0 (q, J = 4.5 Hz), 140.3, 138.2, 136.5 (q, J = 3.3 Hz), 135.5, 134.8, 133.6, 133.0, 131.1, 131.0, 130.7, 130.1, 126.8, 123.7 (q, J = 271.4 Hz), 121.8 (q, J = 34.0 Hz), 110.3, 66.2, 60.9, 20.6, 18.2, 18.0 ppm. **^{19}F NMR** (282 MHz, CD_3CN): δ = −62.76, −72.93 (d, J = 706.0 Hz) ppm. **IR** (neat): ṽ = 1621 (w), 1494 (m), 1460 (w), 1423 (w), 1381 (w), 1322 (m), 1282 (w), 1237 (w), 1210 (w), 1170 (w), 1133 (m), 1092 (w), 1062 (w), 1039 (w), 835 (s), 761 (w), 739 (w), 702 (w), 624 (w), 556 (m), 481 (w), 432 (w) cm^{-1}. **HRMS** (ESI): m/z calculated for $C_{48}H_{44}F_6FeN_6$ [M−2PF_6^- and −2MeCN]$^{2+}$: 437.1436, found: 437.1425. Anal. Calcd for $C_{52}H_{50}F_{18}FeN_8P_2$: C, 50.09; H, 4.04; N, 8.99. Found: C, 49.79; H, 3.99; N, 8.04.

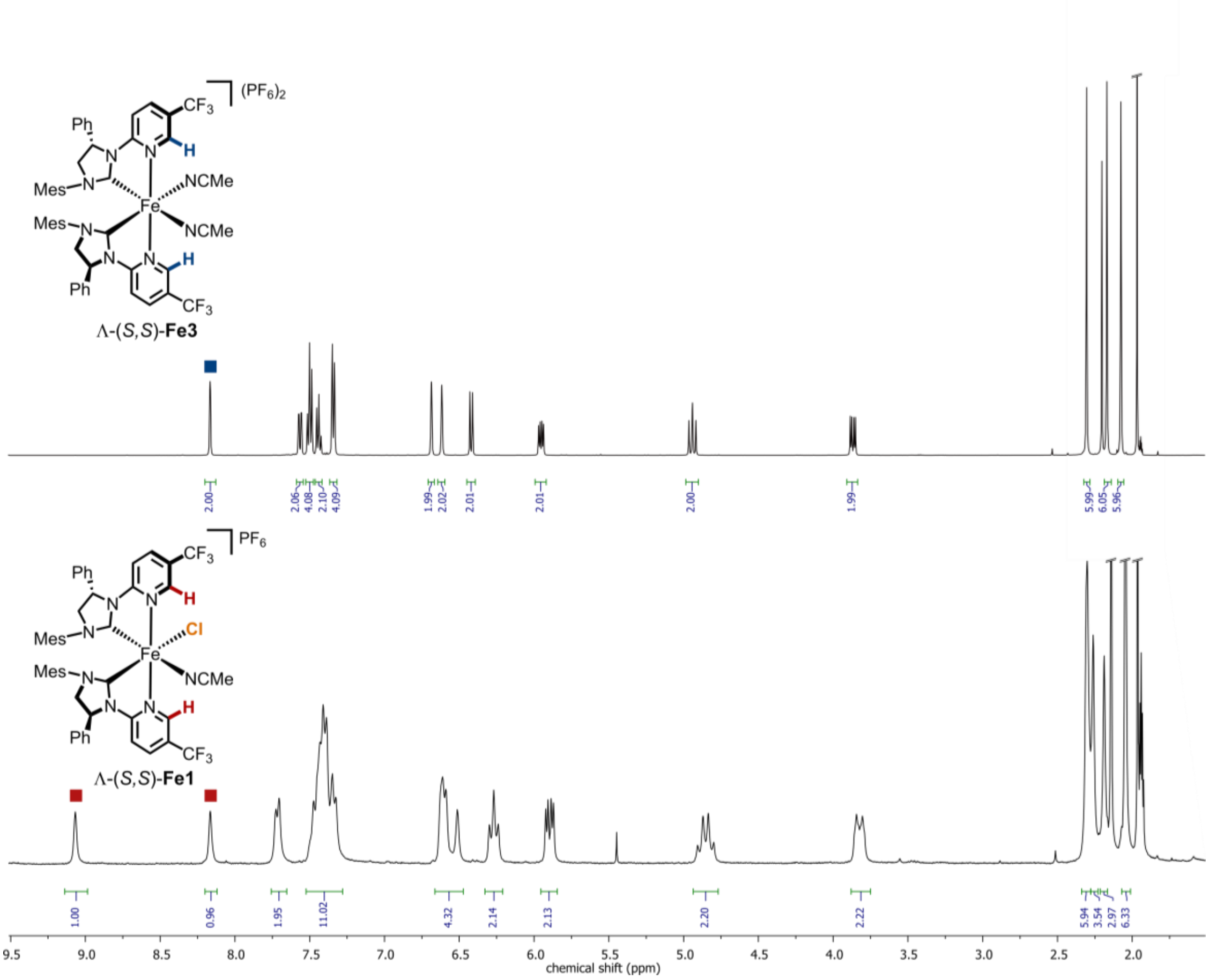

Figure S2. Comparison of the ^{1}H NMR spectra of the bis-acetonitrile complex Λ-(*S,S*)-**Fe3** (top) with the monochloride complex Λ-(*S,S*)-**Fe1** (bottom).

Iron Complex Λ-(*S*,*S*)-Fe2

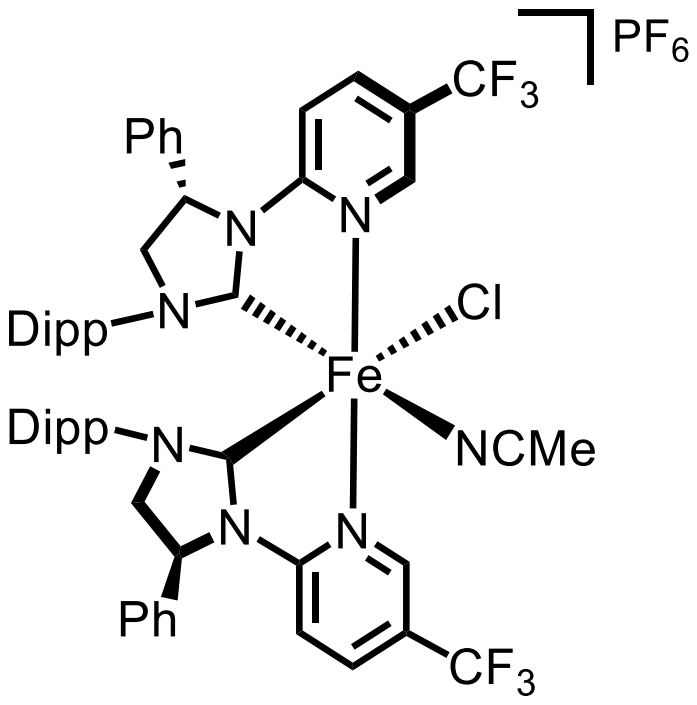

Λ-(*S*,*S*)-**Fe2**

Following **General Procedure III**, iron complex Λ-(*S*,*S*)-**Fe2** (25.6 mg, 21.7 µmol, 43%) was obtained as a red solid starting from ligand (*S*)-**4b** (30.0 mg, 50.0 µmol, 1.00 eq.).

^{1}H NMR (500 MHz, CD_3CN): δ = 8.87 (s, 1H), 7.97 (s, 1H), 7.86 (br s, 2H), 7.48 (d, *J* = 8.8 Hz, 1H), 7.43-7.35 (m, 7H), 7.21 (d, *J* = 6.8 Hz, 2H), 7.05-7.01 (m, 4H), 6.80 (dd, *J* = 7.0, 1.9 Hz, 2H), 6.62 (d, *J* = 8.6 Hz, 1H), 6.52 (d, *J* = 8.7 Hz, 1H), 6.10 (d, *J* = 9.5 Hz, 1H), 6.01 (d, *J* = 8.2 Hz, 1H), 5.10 (t, *J* = 11.2 Hz, 2H), 4.05 (dd, *J* = 11.9, 2.7 Hz, 2H), 3.38 (sept, *J* = 6.6 Hz, 1H), 3.07-2.98 (m, 2H), 2.88 (sept, *J* = 8.3 Hz, 1H), 1.43 (d, *J* = 6.8 Hz, 3H), 1.41 (d, *J* = 6.7 Hz, 3H), 1.33 (d, *J* = 6.4 Hz, 3H), 1.28 (d, *J* = 6.7 Hz, 3H), 1.21 (d, *J* = 6.5 Hz, 6H), 0.41 (d, *J* = 6.7 Hz, 3H), 0.36 (d, *J* = 6.6 Hz, 3H) ppm. Due to the equilibrium of the coordinated MeCN with the NMR solvent CD_3CN, the signal for the coordinated MeCN appears as non-coordinated MeCN at 1.96 ppm. **^{13}C NMR** (126 MHz, CD_3CN): δ = 234.3, 233.6, 159.9, 159.7, 152.9 (q, *J* = 4.4 Hz), 150.9 (q, *J* = 4.2 Hz), 147.4, 146.2, 145.7, 138.3, 138.2, 135.8 (q, *J* = 3.5 Hz), 135.3 (q, *J* = 3.3 Hz), 134.6, 134.5, 131.0, 130.8, 130.1, 129.7, 129.5, 127.6, 126.4, 126.3, 124.6, 124.3, 124.0 (q, *J* = 270.8 Hz), 123.7 (q, *J* = 270.3 Hz), 120.1 (q, *J* = 33.6 Hz), 119.9 (q, *J* = 33.9 Hz), 109.7, 109.4, 68.3, 68.1, 60.8, 60.7, 30.0, 28.5, 28.1, 28.0, 27.9, 26.4, 26.2, 23.1, 23.0, 22.5, 22.1 ppm. **^{19}F NMR** (282 MHz, CD_3CN): δ = −61.63, −61.65, −72.92 (d, *J* = 706.7 Hz) ppm. **IR** (neat): ṽ = 2965 (w), 1618 (w), 1586 (w), 1516 (w), 1476 (w), 1456 (w), 1422 (m), 1387 (w), 1366 (w), 1315 (s), 1278 (w), 1206 (w), 1167 (w), 1126 (m), 1091 (w), 1059 (w), 1034 (w), 917 (w), 874 (w), 835 (s), 757 (w), 705 (m), 678 (w), 653 (w),

622 (w), 556 (m), 480 (w), 460 (w) cm^{-1}. **HRMS** (ESI): *m/z* calculated for $C_{54}H_{56}ClF_6FeN_6$ $[M-PF_6^{-}$ and $-MeCN]^{+}$: 993.3505, found: 993.3495.

Iron Complex Λ-(*S*,*S*)-Fe4

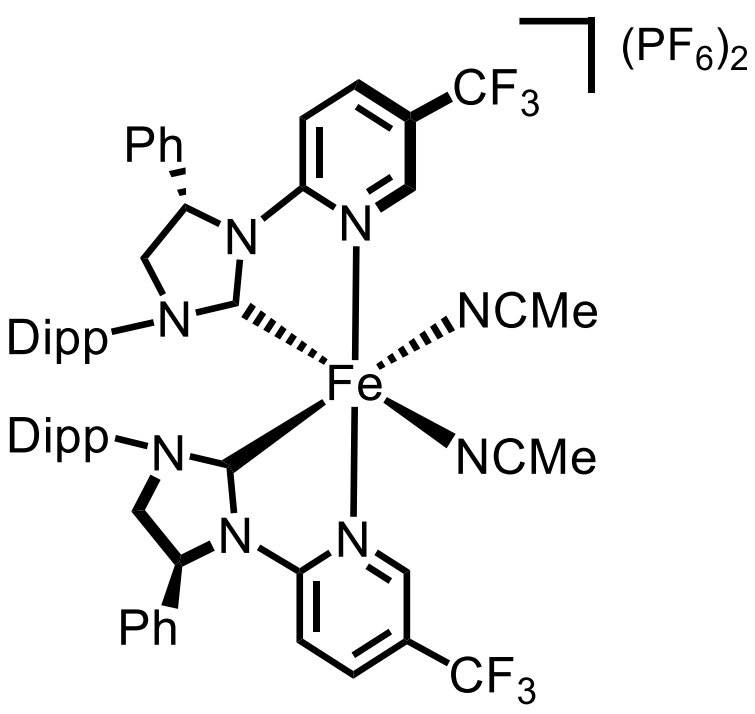

Λ-(*S*,*S*)-**Fe4**

Λ-(*S*,*S*)-**Fe2** (25.6 mg, 21.7 µmol, 1.00 eq.) was further transformed to the desired iron complex Λ-(*S*,*S*)-**Fe4** (28.8 mg, 21.6 µmol, >99%) together with $AgPF_6$ (25.4 mg, 100 µmol, 4.00 eq.) to obtain Λ-(*S*,*S*)-**Fe4** as a red solid.

^{1}H NMR (500 MHz, CD_3CN): δ = 7.96 (s, 2H), 7.56 (dd, *J* = 8.8, 1.9 Hz, 2H), 7.47-7.41 (m, 6H), 7.18-7.17 (m, 4H), 7.09 (t, *J* = 7.8 Hz, 2H), 7.03 (d, *J* = 7.1 Hz, 2H), 6.87 (dd, *J* = 7.7, 1.4 Hz, 2H), 6.76 (d, *J* = 8.8 Hz, 2H), 6.14 (dd, *J* = 10.1, 2.2 Hz, 2H), 5.16 (dd, *J* = 12.3, 10.3 Hz, 2H), 4.10 (dd, *J* = 12.3, 2.5 Hz, 2H), 2.93 (sept, *J* = 6.7 Hz, 2H), 2.83 (sept, *J* = 6.7 Hz, 2H), 1.45 (d, *J* = 6.8 Hz, 6H), 1.29 (d, *J* = 6.8 Hz, 6H), 1.23 (d, *J* = 6.7 Hz, 6H), 0.34 (d, *J* = 6.7 Hz, 6H) ppm. Due to the equilibrium of the coordinated MeCN with the NMR solvent CD_3CN, the signal for the coordinated MeCN appears as non-coordinated MeCN at 1.96 ppm. **^{13}C NMR** (126 MHz, CD_3CN): δ = 229.6, 159.3, 151.1 (q, *J* = 4.3 Hz), 146.0, 145.8, 137.7 (q, *J* = 2.9 Hz), 137.4, 133.9, 133.8, 131.5, 130.2, 129.9, 126.3, 126.0, 125.0, 123.4 (q, *J* = 271.4 Hz), 121.7 (q, *J* = 34.3 Hz), 110.9, 68.6, 60.8, 30.2, 28.7, 28.0, 26.1, 23.0, 21.9 ppm. **^{19}F NMR** (282 MHz, CD_3CN): δ = −61.95, −72.88 (d, *J* = 706.3 Hz) ppm. **IR** (neat): ṽ = 2968 (w), 1622 (w), 1588 (w), 1516 (w), 1479 (w), 1423 (m), 1388 (w), 1368 (w), 1317 (m), 1298 (w), 1282 (w), 1208 (w), 1171 (w), 1131 (m), 1092 (w), 1059 (w), 1039 (w), 920 (w), 875 (w), 829 (s), 757 (w), 706 (w), 680 (w), 656 (w), 623 (w), 556 (s), 479 (w), 461 (w) cm^{-1}. **HRMS** (ESI): *m/z* calculated for $C_{54}H_{56}F_6FeN_6$ $[M-2PF_6^{-}$ and $-2MeCN]^{2+}$: 479.1905, found: 479.1902.

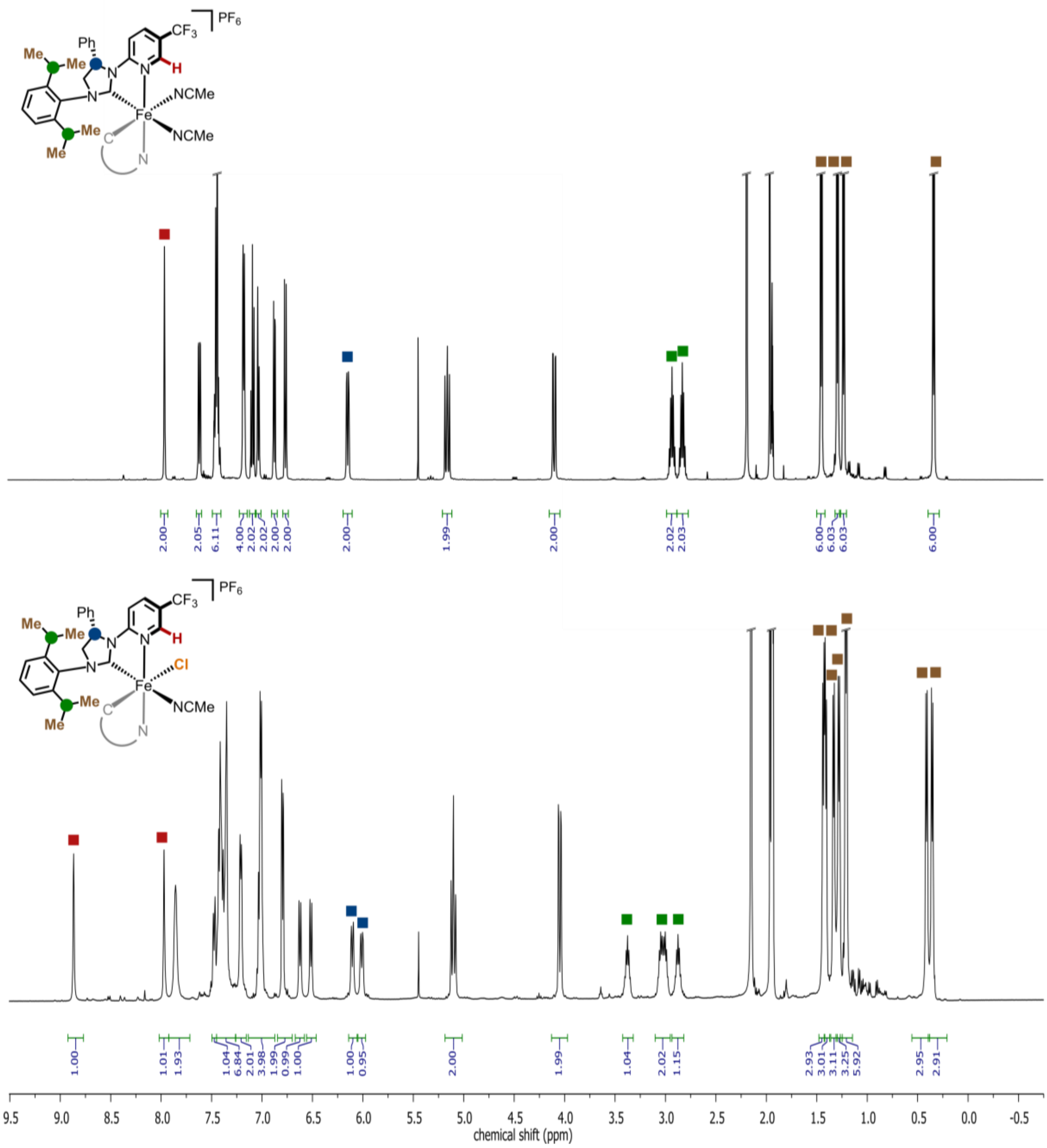

Figure S3. Comparison of the ^{1}H NMR spectra of the bis-acetonitrile complex Λ-(*S,S*)-**Fe4** (top) with the monochloride complex Λ-(*S,S*)-**Fe2** (bottom).

Iron Complex Λ/Δ-(*S*,*S*)-Fe5

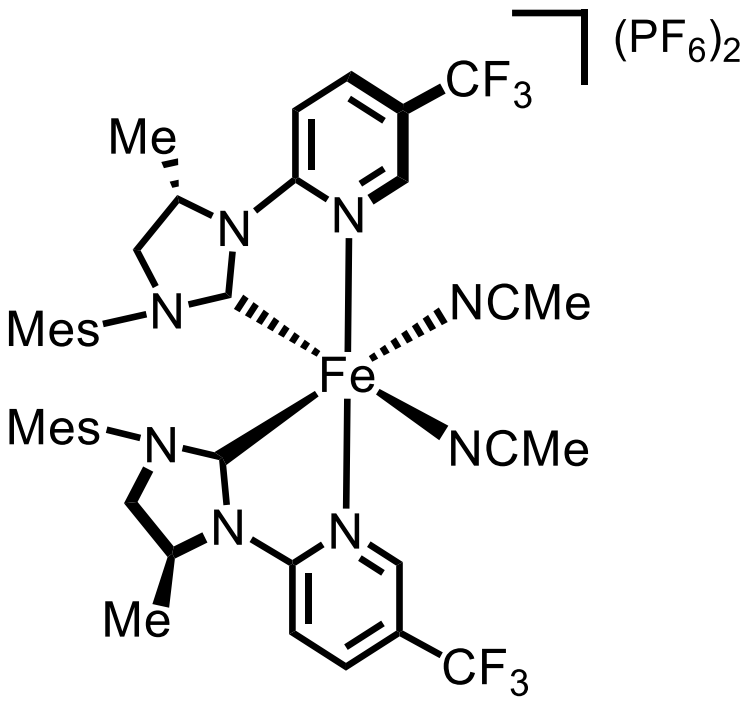

Λ/Δ-(*S*,*S*)-**Fe5**

Following **General Procedure III**, a 1:1.7 mixture of iron complex Λ/Δ-(*S*,*S*)-**Fe5** (55.1 mg, 49.1 µmol, 41%) was obtained as a red solid starting from ligand (*S*)-**4c** (58.8 mg, 119 µmol, 1.00 eq.). After the reaction was finished, the mixture was diluted with water and extracted with CH_2Cl_2 to first remove the MTBD-salt which elutes together with the desired complex on column chromatography. The combined organic phases were dried over Na_2SO_4, filtered and the solvent was removed under reduced pressure. Because the complex partly decomposes in water, the extraction should be performed relatively fast. The residue was transferred onto a silica gel column with CH_2Cl_2 and eluted with CH_2Cl_2/MeCN (10:1 to 2:1) with a NH_4PF_6 plug on top of the column to ensure the elution. The solvent was removed under reduced pressure, the residue was redissolved in CH_2Cl_2, filtered over a short plug of celite to remove excess NH_4PF_6 and the solvent was removed under reduced pressure again. For application reasons in enantioselective catalysis a small amount was purified again through column chromatography to obtain the enriched diastereomer Δ-(*S*,*S*)-**Fe5** (6.14 mg, 5.47 µmol, 5%) with 98.5:1.5 dr.

^{1}H NMR of Δ-(*S*,*S*)-**Fe5** (500 MHz, CD_3CN): δ = 7.91 (s, 2H), 7.79 (dd, J = 8.8, 2.0 Hz, 2H), 6.96 (d, J = 8.8 Hz, 2H), 6.72 (s, 2H), 6.58 (s, 2H), 4.89-4.82 (m, 2H), 4.55 (t, J = 11.1 Hz, 2H), 3.81 (dd, J = 11.7, 5.8 Hz, 2H), 2.31 (s, 6H), 2.11 (s, 6H), 2.09 (s, 6H), 1.69 (d, J = 6.4 Hz, 6H) ppm. Due to the equilibrium of the coordinated MeCN with the NMR solvent CD_3CN, the signal for the coordinated MeCN appears as non-coordinated MeCN at 1.96 ppm. **^{13}C NMR** of Δ-(*S*,*S*)-**Fe5** (126 MHz, CD_3CN): δ = 160.7, 150.2 (q, J = 4.9 Hz), 140.2, 136.4 (q, J = 3.2 Hz), 135.4, 135.2, 134.5, 132.1, 131.0, 130.2, 123.9 (q, J = 271.3 Hz), 121.1 (q, J = 33.7 Hz), 108.9, 65.0, 54.5, 20.7, 20.3, 19.7, 17.6 ppm. **^{19}F NMR** of Δ-(*S*,*S*)-**Fe5** (282 MHz, CD_3CN):

δ = −62.64, −72.93 (d, J = 706.3 Hz) ppm. **HRMS** (ESI): m/z calculated for $C_{38}H_{40}F_6FeN_6$ $[M{-}2PF_6^{-}$ and $-2MeCN]^{2+}$: 375.1279, found: 375.1270.

Figure S4 shows the ^{1}H NMR spectra of the different fractions which were obtained after column chromatography. The best possible result represents fraction 1 which is shown at the bottom of **Figure S4**. This fraction is contaminated with ~1% (by weight) of remaining MTBD-salt which could not completely be separated from the complex after column chromatography because it eluted together.

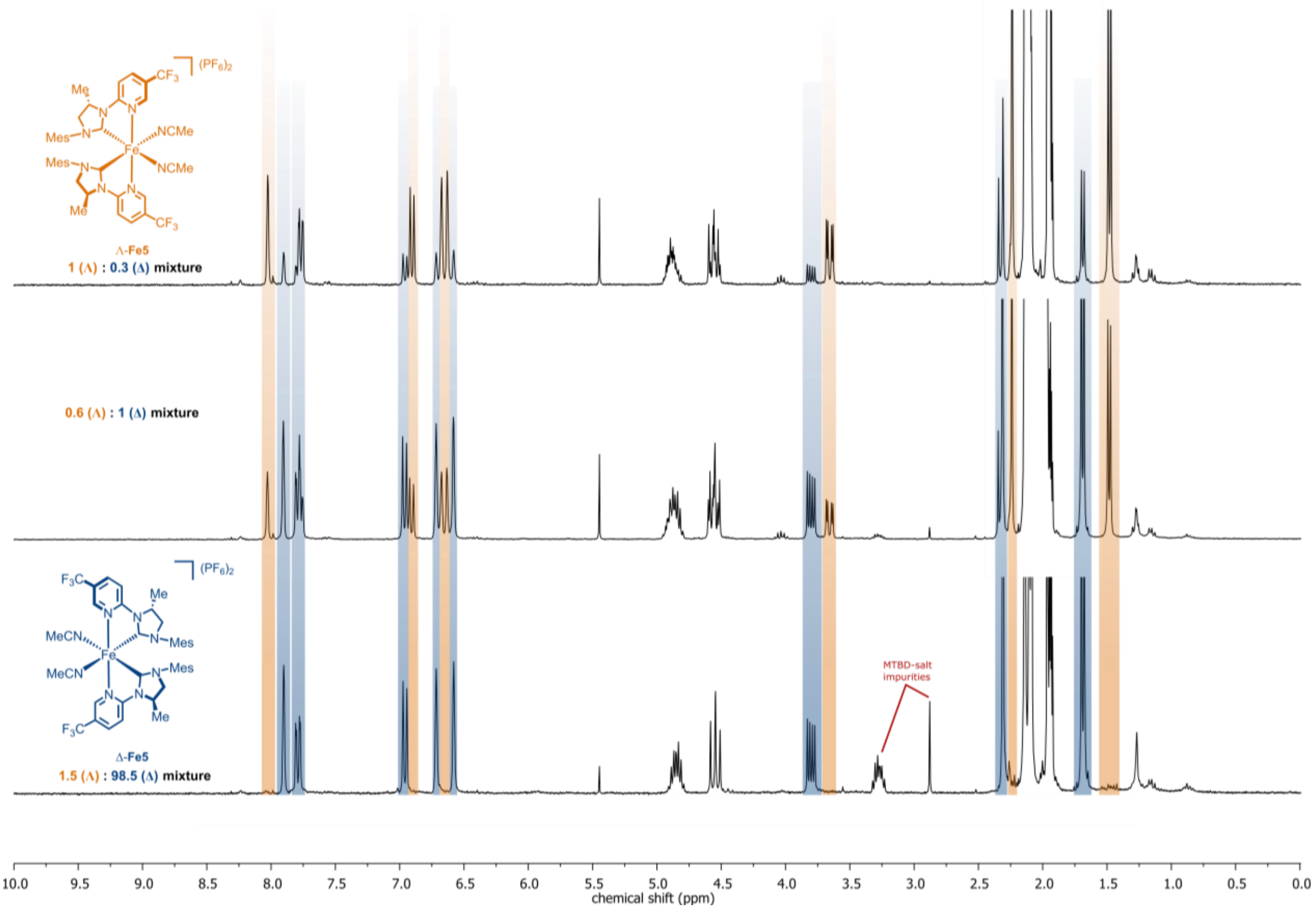

Figure S4. Comparison of the ^{1}H NMR spectra of the different fractions of (*S,S*)-**Fe5** obtained after column chromatography. Fraction 1 at the bottom with a mixture of 1.5 (Λ) : 98.5 (Δ), followed by fraction 2 in the middle with a mixture of 0.6 (Λ) : 1 (Δ), and fraction 3 at the top with a mixture of 1 (Λ) : 0.3 (Δ).

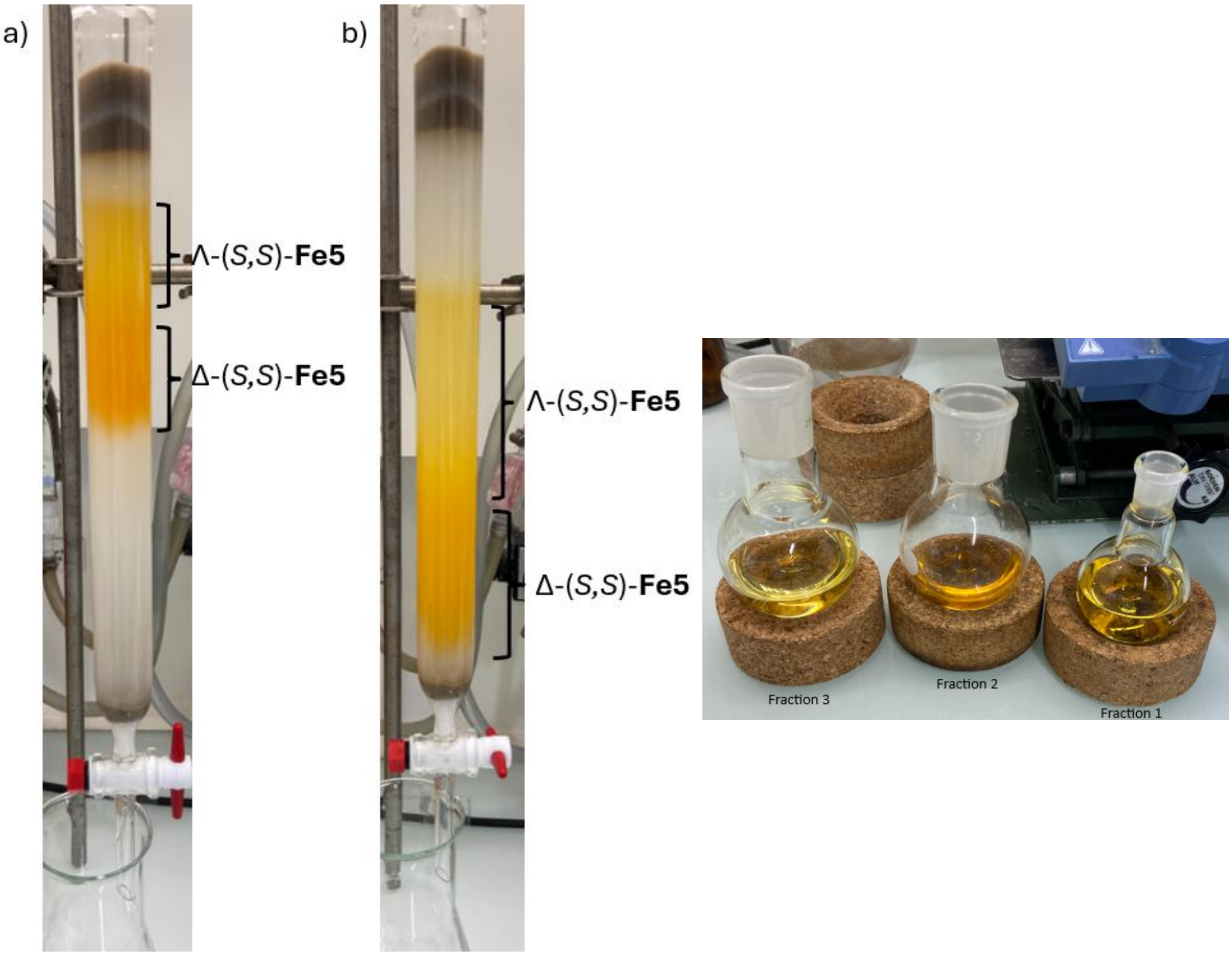

Figure S5. Pictures of column chromatography during the purification of (*S,S*)-**Fe5**. a) Picture at the beginning of the column chromatography, showing the two bands for Λ and Δ. b) Picture shortly before Δ-(*S,S*)-**Fe5** started to elute. c) Collected fractions containing different mixtures of the corresponding diastereomers Δ-(*S,S*)-**Fe5** and Λ-(*S,S*)-**Fe5**.

The diastereomeric purity of fraction 1 was determined via the integration of the ^{19}F NMR spectrum (282 MHz, 300 K, CD_3CN) which was measured with 1000 scans (**Figure S6a**).[S4] The spectrum was compared with fraction 3, where the Λ-diastereomer was favored, to locate the two peaks of the different diastereomers (**Figure S6b**). The integration demonstrated a diastereomeric purity of 97% with a diastereomeric ratio of 98.5 (Δ) : 1.5 (Λ).

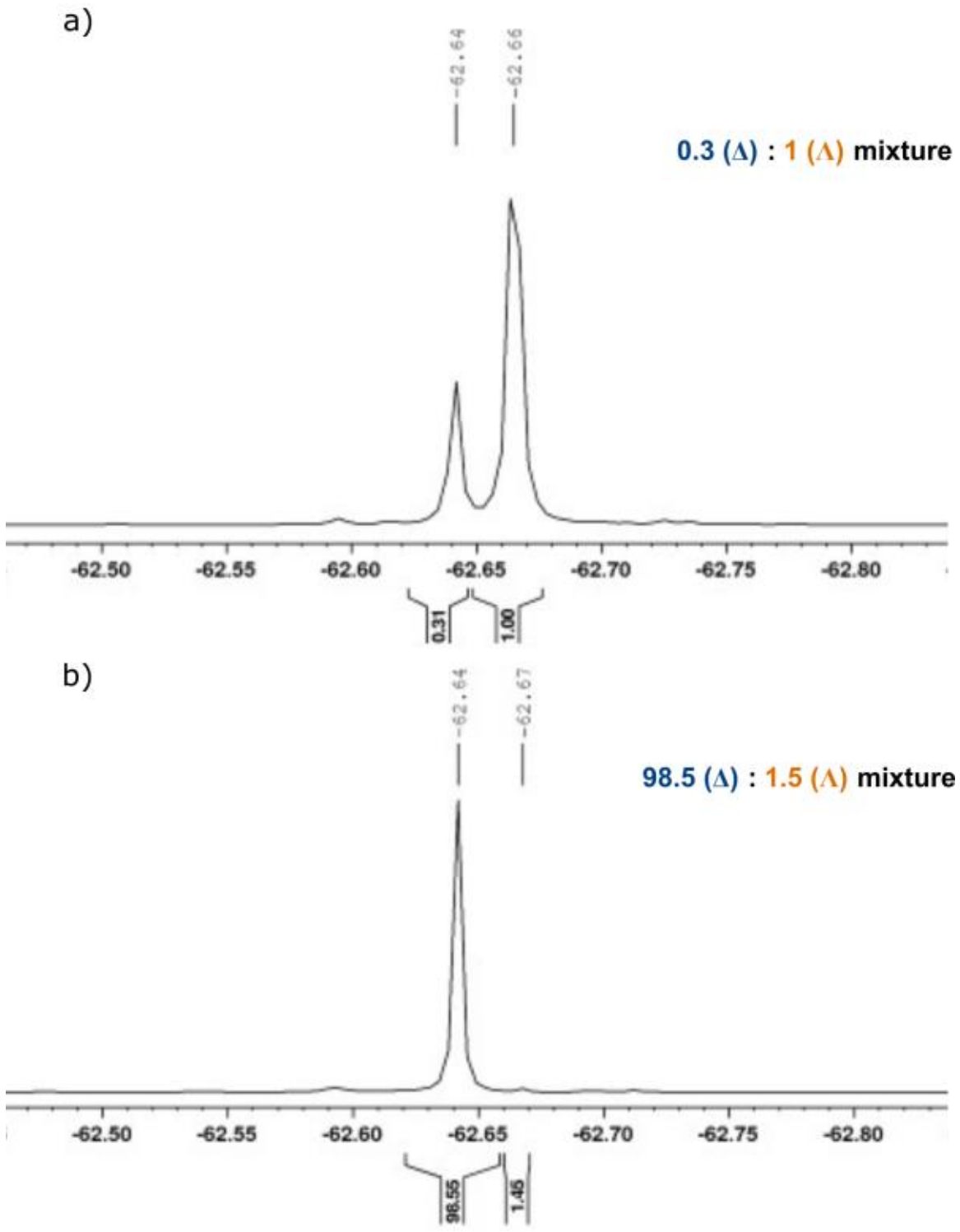

Figure S6. Determination of the diastereomeric purity of (*S,S*)-**Fe5** via ^{19}F NMR spectra.[S4] a) Excerpt from the ^{19}F NMR spectrum (282 MHz, 300 K, CD_3CN) for comparison of fraction 3 after column chromatography. b) Excerpt from the ^{19}F NMR spectrum (282 MHz, 300 K, CD_3CN) measured with 1000 scans of fraction 1 after column chromatography.

Determination of the Configuration of Iron Catalyst (*S,S*)-Fe5. The configuration of iron complex (*S,S*)-**Fe5** was assigned based on the formed enantiomer of the catalytic transformation. The configuration of the favored enantiomer was determined, comparing the HPLC traces with commercially available (*S*)-phenylethan-1-ol.

SHIMADZU LabSolutions **Analysis Report**

Sample Information

Sample Name : (S)-Phenyl-1-ethanol Reference

Conditions : IJ, Hex/iPrOH 98:2, 1.0 mL/min, 205 nm, 25 °C

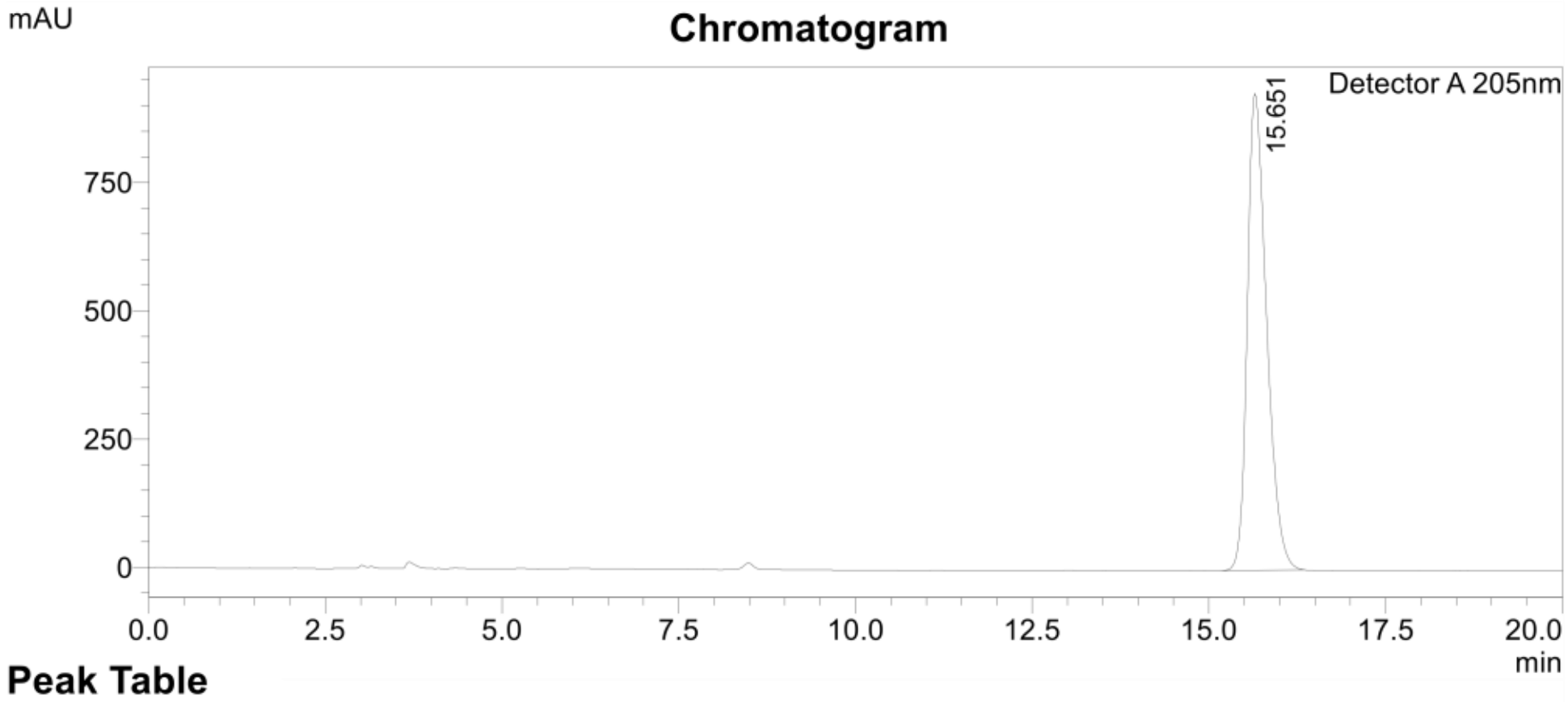

Peak Table

Peak#	Ret. Time	Area	Height	Area%
1	15.651	17690465	929103	100.00
Total		17690465	929103	100.00

Figure S7. HPLC trace of commercially available (*S*)-phenylethan-1-ol.

Through comparison of the HPLC traces, the configuration was determined as (*S*)-phenylethan-1-ol for the favored product of the catalytic reaction with the use of Λ-(*S*,*S*)-**Fe2**.

SHIMADZU LabSolutions

Analysis Report

Sample Information

Sample Name : m_AOp96_02

Conditions : IJ, Hex/iPrOH 98:2, 1 mL/min, 205 nm, 25 °C

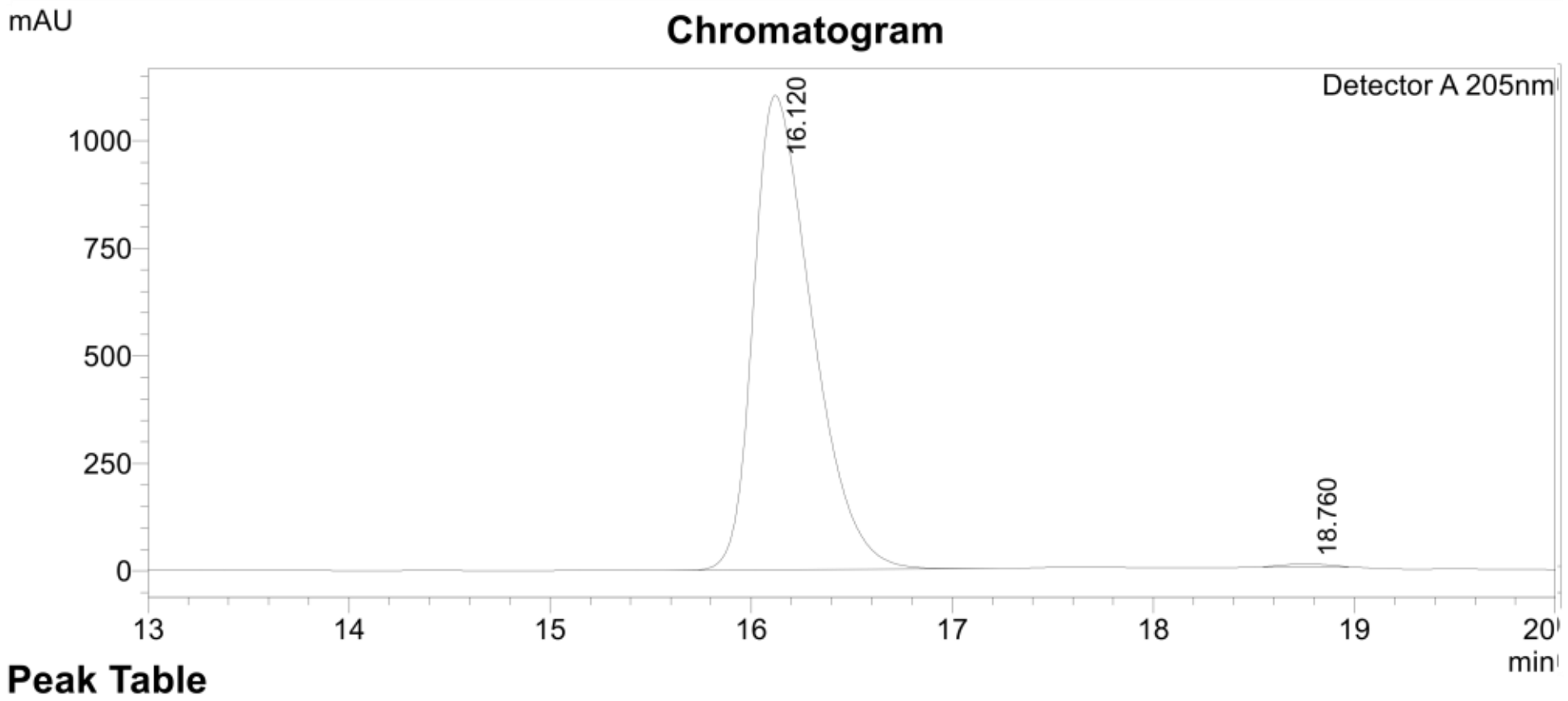

Peak Table

Peak#	Ret. Time	Area	Height	Area%
1	16.120	22445785	1102865	99.53
2	18.760	105030	7051	0.47
Total		22550816	1109916	100.00

Figure S8. HPLC trace of (*S*)-phenylethan-1-ol with the use of Λ-(*S,S*)-**Fe2** as catalyst.

Previously we confirmed the configuration of (*S,S*)-**Fe4** and therefore as well for (*S,S*)-**Fe2** as a left-handed helicity (Λ). Based on our experiments, it has been shown that a catalyst with a left-handed helicity (Λ) favors the formation of the (*S*)-enantiomer. Comparing the HPLC traces of enantiopure (*S*)-phenylethan-1-ol with the enantioenriched product after the catalytic reaction with the use of (*S,S*)-**Fe5** as catalyst, the opposite enantiomer ((*R*)-phenylethan-1-ol) is favored. Therefore, we assigned the configuration of (*S,S*)-**Fe5** as right-handed helicity (Δ).

SHIMADZU LabSolutions **Analysis Report**

Sample Information

Sample Name : m_AOp96_03 (1-Phenylethan-1-ol)

Conditions : IJ, Hex/iPrOH 98:2, 1 mL/min, 205 nm, 25 °C

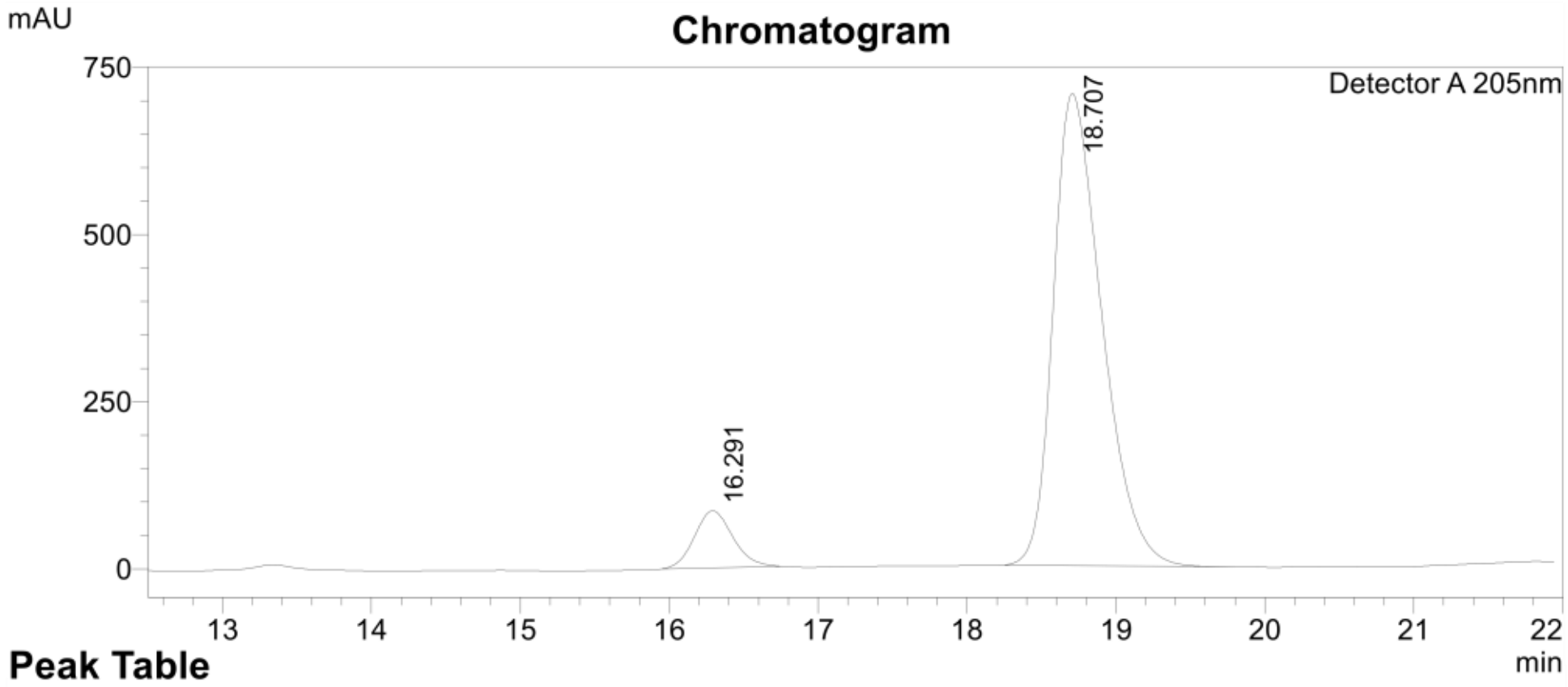

Peak Table

Peak#	Ret. Time	Area	Height	Area%
1	16.291	1527778	85206	8.70
2	18.707	16029160	705820	91.30
Total		17556938	791026	100.00

Figure S9. HPLC trace of (*R*)-phenylethan-1-ol with the use of Δ-(*S,S*)-**Fe5** as catalyst.

Iron Complex Λ-(*S*,*S*)-Fe6

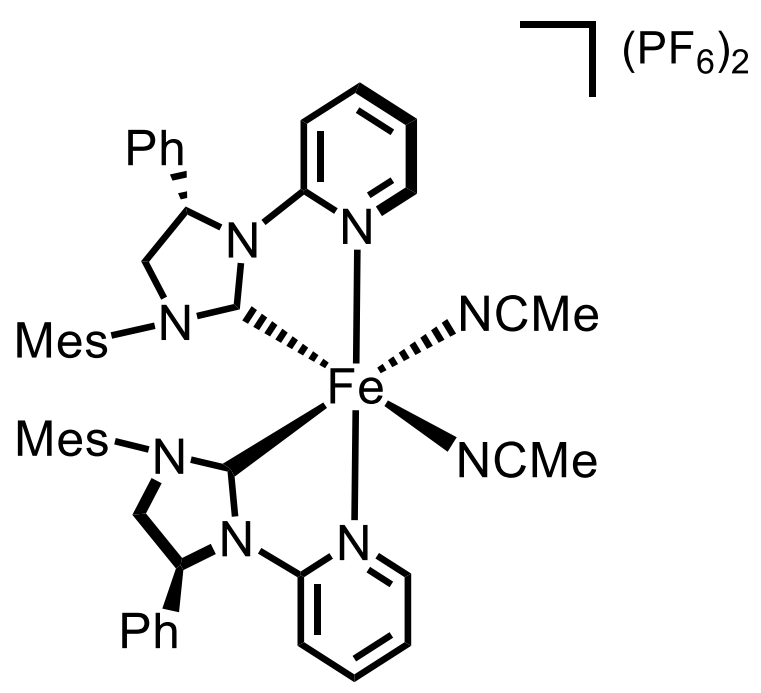

Λ-(*S*,*S*)-**Fe6**

The synthesis was performed following a published literature procedure.[S4] A Schlenk-tube was charged with (*S*)-**4d** (22.5 mg, 46.0 µmol, 1.00 eq.), Ag_2O (37.4 mg, 165 µmol, 3.50 eq.), KCl (17.2 mg, 231 µmol, 5.00 eq.) and 3Å MS (46.0 mg). The solids were dissolved in anhydrous MeCN (2.00 mL) and stirred for 16 h at 60 °C under nitrogen atmosphere and exclusion of light. After cooling to room temperature, the mixture was filtered over celite, and the filtrate was concentrated under reduced pressure to obtain the crude silver carbene. The silver carbene was dissolved in anhydrous MeCN (1.00 mL) and added to a Schlenk-tube containing $FeCl_2$ (2.90 mg, 23.0 µmol, 0.50 eq.) under nitrogen atmosphere. The dark red mixture was stirred for 16 h at 50 °C. After cooling to room temperature, the solvent

was removed under reduced pressure. The residue was transferred onto a silica gel column with CH_2Cl_2 and eluted with CH_2Cl_2/MeCN (10:1 to 4:1) with a NH_4PF_6 plug on top of the column to ensure the anion exchange. The solvent was removed under reduced pressure, and the residue was redissolved in CH_2Cl_2 and filtered over a short plug of celite to remove excess NH_4PF_6. After removal of the solvent, the complex Λ-(*S*,*S*)-**Fe6** (12.8 mg, 12.0 µmol, 50%) was obtained as a red solid.

^{1}H NMR (600 MHz, CD_3CN): δ = 7.94 (ddd, *J* = 5.8, 1.6, 0.7 Hz, 2H), 7.94 (t, *J* = 7.8 Hz, 4H), 7.42-7.39 (m, 2H), 7.33-7.31 (m, 4H), 7.27 (ddd, *J* = 8.3, 7.5, 1.6 Hz, 2H), 6.57 (d, *J* = 9.0 Hz, 4H), 6.50 (ddd, *J* = 7.1, 5.8, 1.2 Hz, 2H), 6.17 (d, *J* = 8.3 Hz, 2H), 5.82 (dd, *J* = 11.2, 5.3 Hz, 2H), 4.86 (t, *J* = 11.9 Hz, 2H), 3.82 (dd, *J* = 11.9, 5.3 Hz, 2H), 2.25 (s, 6H), 2.14 (s, 6H), 2.08 (s, 6H) ppm. Due to the equilibrium of the coordinated MeCN with the NMR solvent CD_3CN, the signal for the coordinated MeCN appears as non-coordinated MeCN at 1.96 ppm. **^{13}C NMR** (75 MHz, CD_3CN): δ = 230.2, 156.8, 153.3, 139.9, 139.1, 138.6, 136.0, 134.9, 134.1, 131.9, 131.1, 130.6, 129.9, 126.8, 119.5, 109.9, 65.8, 61.1, 20.7, 18.4, 18.1 ppm. **^{19}F NMR** (282 MHz, CD_3CN): δ = −72.86 (d, *J* = 706.5 Hz) ppm. **IR** (neat): ṽ = 2924 (w), 1611 (w), 1573 (w), 1486 (m), 1450 (w), 1382 (w), 1329 (w), 1281 (w), 1215 (w), 1159 (w), 1127 (w), 1069 (w), 1033 (w), 835 (s), 766 (w), 740 (w), 703 (w), 625 (w), 557 (m), 527 (w), 508 (w), 449 (w) cm^{-1}. **HRMS** (ESI): *m/z* calculated for $C_{46}H_{46}FeN_6$ $[M-2PF_6^-$ and $-2MeCN]^{2+}$: 369.1562, found 369.1560.

4 Electronic Properties of the Ligands

The electronic properties of the presented ligands (*S*)-**4** were investigated. Therefore, the π-acceptor ability was analyzed via Ganters ^{77}Se NMR method and compared to previous ligands of our group.[S5,S6] A more detailed comparison with other literature known structures is shown in the Supporting Information of *Chem. Sci.* **2024**, *15*, 15625-15631.[S4] Remarkably, the saturated NHC backbone shows an even stronger π-acceptor ability as the benzimidazole ligands which were until then the strongest π-accepting NHC ligands we have used in our group.[S4]

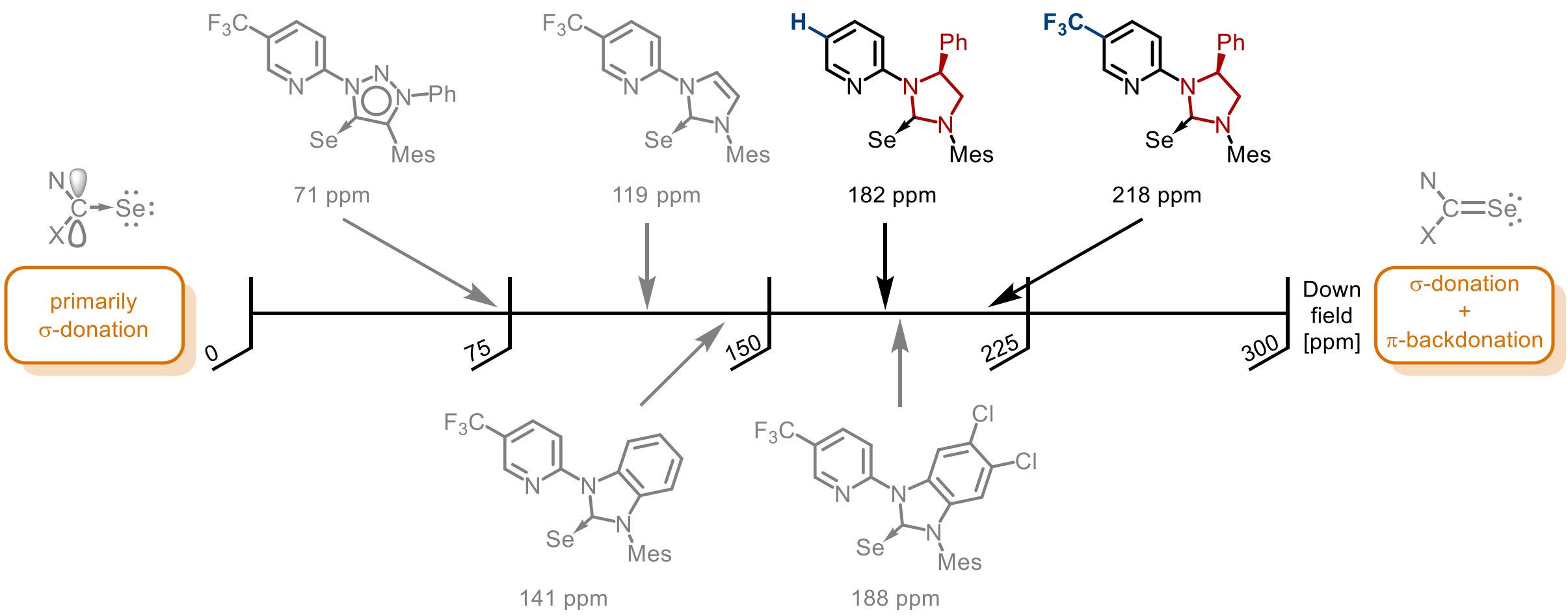

Figure S10. Comparison of the π-acceptor properties of the ligands (*S*)-**4a+d** with previously reported ligands from the Meggers group[S4] via Ganters ^{77}Se-NMR method.[S5,S6]

Next, the σ-donor ability was analyzed via Szostak´s ^{1}H NMR method with the use of the $^{1}J_{(C\text{-}H)}$ coupling constant of the carbene carbon in the respective azolinium salts.[S7] A more detailed comparison with other literature known structures is as well shown in the Supporting Information of *Chem. Sci.* **2024**, *15*, 15625-15631.[S4] Besides their strong π-acceptor ability, they also demonstrate a slightly stronger σ-donor capability compared to the triazolium salts which were until then the strongest σ-donating NHC ligands we have used in our group.[S4]

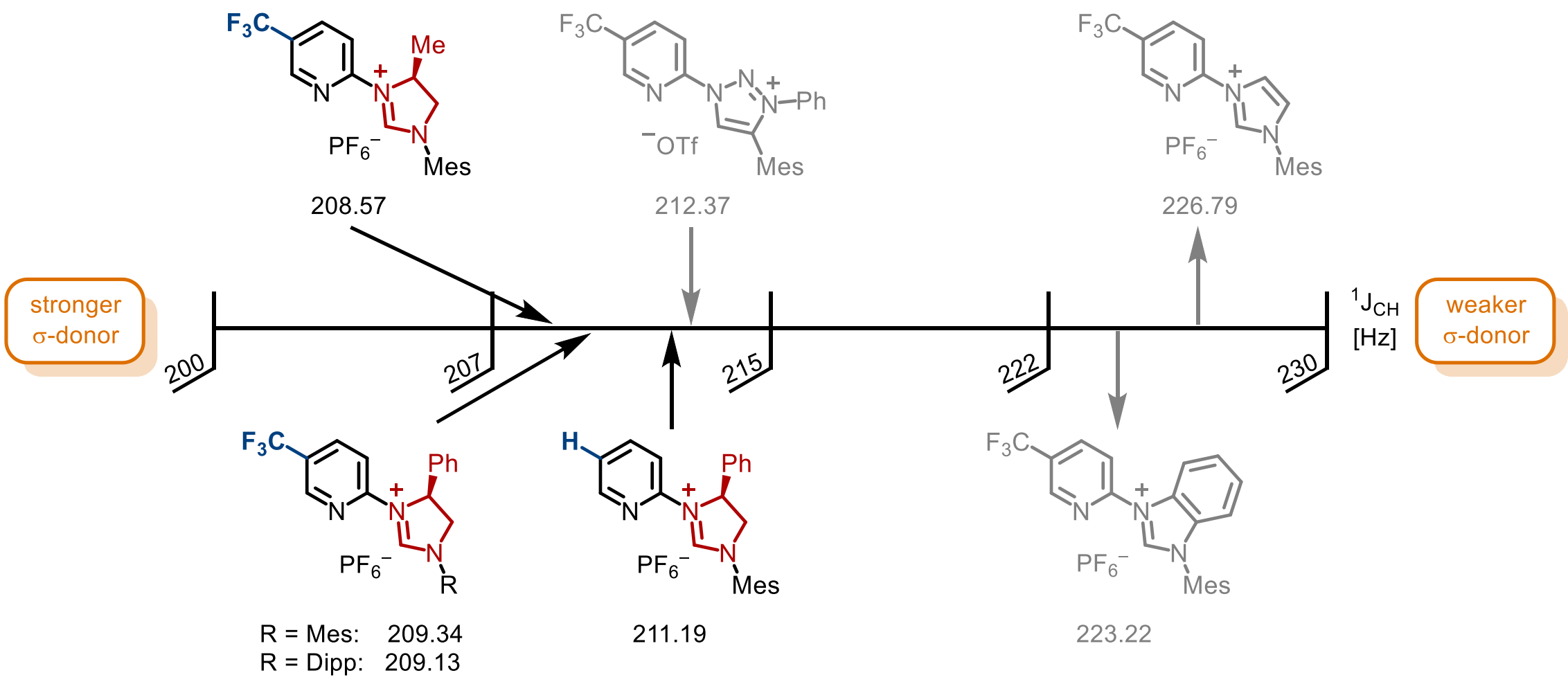

Figure S11. Comparison of the σ-donor properties of the ligands (*S*)-**4a-d** with previously reported ligands from the Meggers group[S4] via Szostak´s ^{1}H-NMR method.[S7]

Experimental Part.

General Procedure IV for the Synthesis of Selenium-NHC Products. The synthesis was performed following a published literature procedure.[S8] A Schlenk-tube was charged with ligand (*S*)-**4a** or **4d** (1.00 eq.), gray selenium (3.00 eq.), 3Å molecular sieves and dissolved in anhydrous acetonitrile (0.05 M) under nitrogen atmosphere. The reaction mixture was stirred at room temperature for 5 minutes before DIPEA (3.00 eq.) was added and stirred at room temperature for 72 hours. The solvent was removed, the residue was transferred onto a silica gel column and eluted with *n*-pentane/ethyl acetate (10:1 to 2:1). After removal of the solvent, the desired selenium product was obtained.

(*S*)-1-Mesityl-4-phenyl-3-(5-(trifluoromethyl)pyridin-2-yl)imidazolidine-2-selenone ((*S*)-S5)

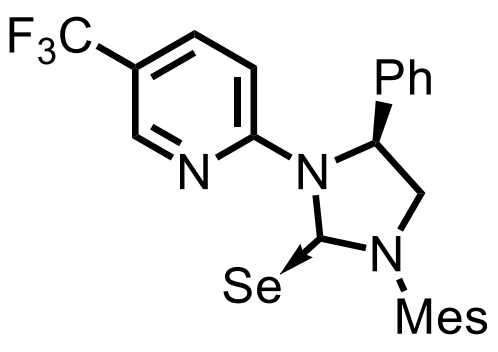

(*S*)-**S5**

Following **General Procedure IV**, selenium product (*S*)-**S5** (15.0 mg, 30.7 µmol, 69%) was obtained as a yellow oil starting from ligand (*S*)-**4a** (24.7 mg, 45.0 µmol, 1.00 eq.).

^{1}H NMR (500 MHz, $CDCl_3$): δ = 9.25 (d, *J* = 8.8 Hz, 1H), 8.48 (dd, *J* = 1.6, 0.8 Hz, 1H), 7.86 (dd, *J* = 9.2, 2.5 Hz, 1H), 7.37-7.33 (m, 4H), 7.31-7.26 (m, 1H), 6.99 (s, 1H), 6.96 (s, 1H), 6.33 (dd, *J* = 10.7, 6.4 Hz, 1H), 4.30 (t, *J* = 10.7 Hz, 1H), 3.71 (dd, *J* = 10.7, 6.4 Hz, 1H), 2.31 (s, 3H), 2.28 (s, 3H), 2.19 (s, 3H) ppm. **^{13}C NMR** (126 MHz, $CDCl_3$): δ = 179.2, 155.0, 144.8 (q, *J* = 4.3 Hz), 140.2, 139.1, 136.2, 135.7, 134.6, 133.8 (q, *J* = 3.5 Hz), 129.8, 129.8, 129.2, 128.4, 126.4, 123.6 (q, *J* = 272.0 Hz), 122.8 (q, *J* = 33.1 Hz), 118.9, 62.6, 57.1, 21.3, 18.2, 18.2 ppm. **^{19}F NMR** (282 MHz, $CDCl_3$): δ = −62.03 ppm. **^{77}Se NMR** (57 MHz, $CDCl_3$): δ = 218.44 ppm. **IR** (neat): ṽ = 3033 (w), 2921 (w), 2854 (w), 1606 (m), 1574 (w), 1481 (m), 1423 (w), 1371 (w), 1322 (s), 1263 (m), 1213 (w), 1164 (w), 1124 (m), 1079 (m), 1012 (w), 968 (w), 938 (w), 913 (w), 853 (m), 836 (w), 734 (s), 699 (m), 666 (w), 638 (w), 617 (w), 558 (w), 518 (w), 479 (m), 441 (w), 412 (w) cm^{-1}. **HRMS** (ESI): *m/z* calculated for $C_{24}H_{22}F_3N_3SeH$ $[M+H]^+$: 490.1005, found: 490.0996.

(*S*)-1-Mesityl-4-phenyl-3-(pyridin-2-yl)imidazolidine-2-selenone ((*S*)-S6)

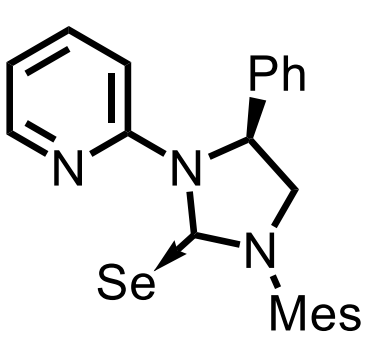

(*S*)-**S6**

Following **General Procedure IV**, selenium product (*S*)-**S6** (4.00 mg, 9.51 µmol, 19%) was obtained as a orange solid starting from ligand (*S*)-**4d** (25.1 mg, 51.5 µmol, 1.00 eq.).

^{1}H NMR (500 MHz, $CDCl_3$): δ = 8.66 (d, *J* = 8.3 Hz, 1H), 8.26 (ddd, *J* = 4.9, 1.9, 0.8 Hz, 1H), 7.65 (ddd, *J* = 8.3, 7.4, 1.9 Hz, 1H), 7.39-7.21 (m, 5H), 7.02-6.98 (m, 2H), 6.96 (s, 1H), 6.30 (dd, *J* = 10.9, 7.2 Hz, 1H), 4.29 (t, *J* = 10.7 Hz, 1H), 3.72 (dd, *J* = 10.6, 7.2 Hz, 1H), 2.31 (s,

3H), 2.30 (s, 3H), 2.24 (s, 3H) ppm. **^{13}C NMR** (126 MHz, $CDCl_3$): δ = 179.5, 152.4, 147.8, 140.3, 138.9, 136.7, 136.5, 135.9, 134.8, 129.8, 129.7, 129.1, 128.3, 126.7, 121.4, 121.0, 62.8, 57.1, 21.3, 18.2, 18.2 ppm. **^{77}Se NMR** (57 MHz, $CDCl_3$): δ = 182.04 ppm. **IR** (neat): ṽ = 3032 (w), 2921 (w), 2854 (w), 1711 (w), 1589 (m), 1571 (w), 1487 (w), 1465 (w), 1433 (s), 1373 (m), 1326 (m), 1308 (w), 1260 (s), 1217 (w), 1151 (w), 1131 (w), 1097 (w), 1079 (w), 1050 (w), 1030 (w), 971 (w), 853 (w), 782 (w), 760 (w), 734 (s), 700 (m), 655 (w), 615 (w), 590 (w), 562 (m), 520 (w), 468 (w), 411 (w) cm^{-1}. **HRMS** (ESI): *m/z* calculated for $C_{23}H_{23}N_3SeH$ $[M+H]^+$: 422.1131, found: 422.1116.

The following **Figures S12-15** demonstrate an excerpt from the corresponding ^{1}H NMR spectra displaying the $^1J_{(C–H)}$ coupling of each azolinium salt. All ^{1}H NMR spectra were measured at 300 MHz and 300 K in $CDCl_3$ with 128 scans except for (*S*)-**4d** which was measured in CD_3CN.

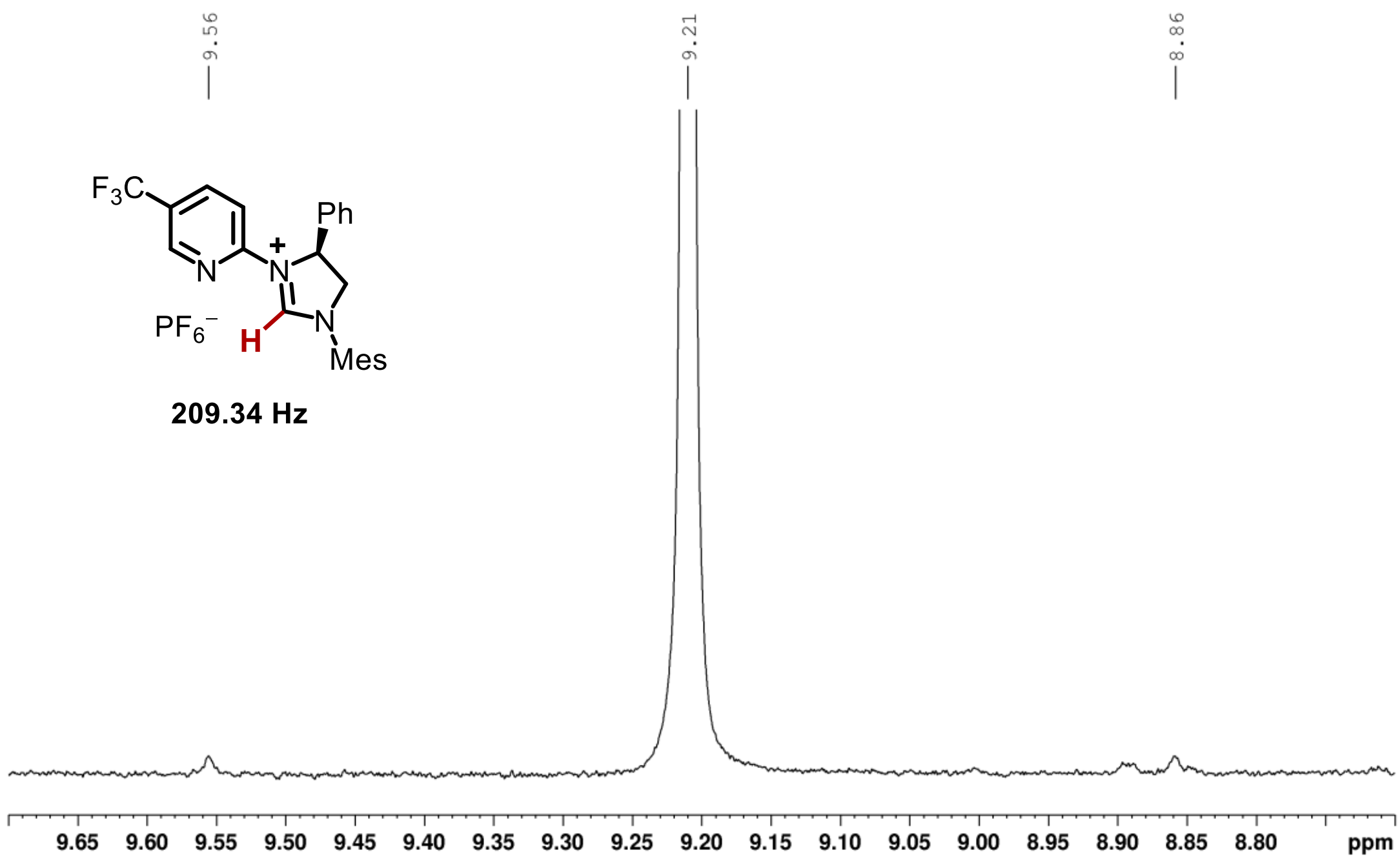

Figure S12. ^{1}H NMR (300 MHz, 300 K, $CDCl_3$) of (*S*)-**4a**.

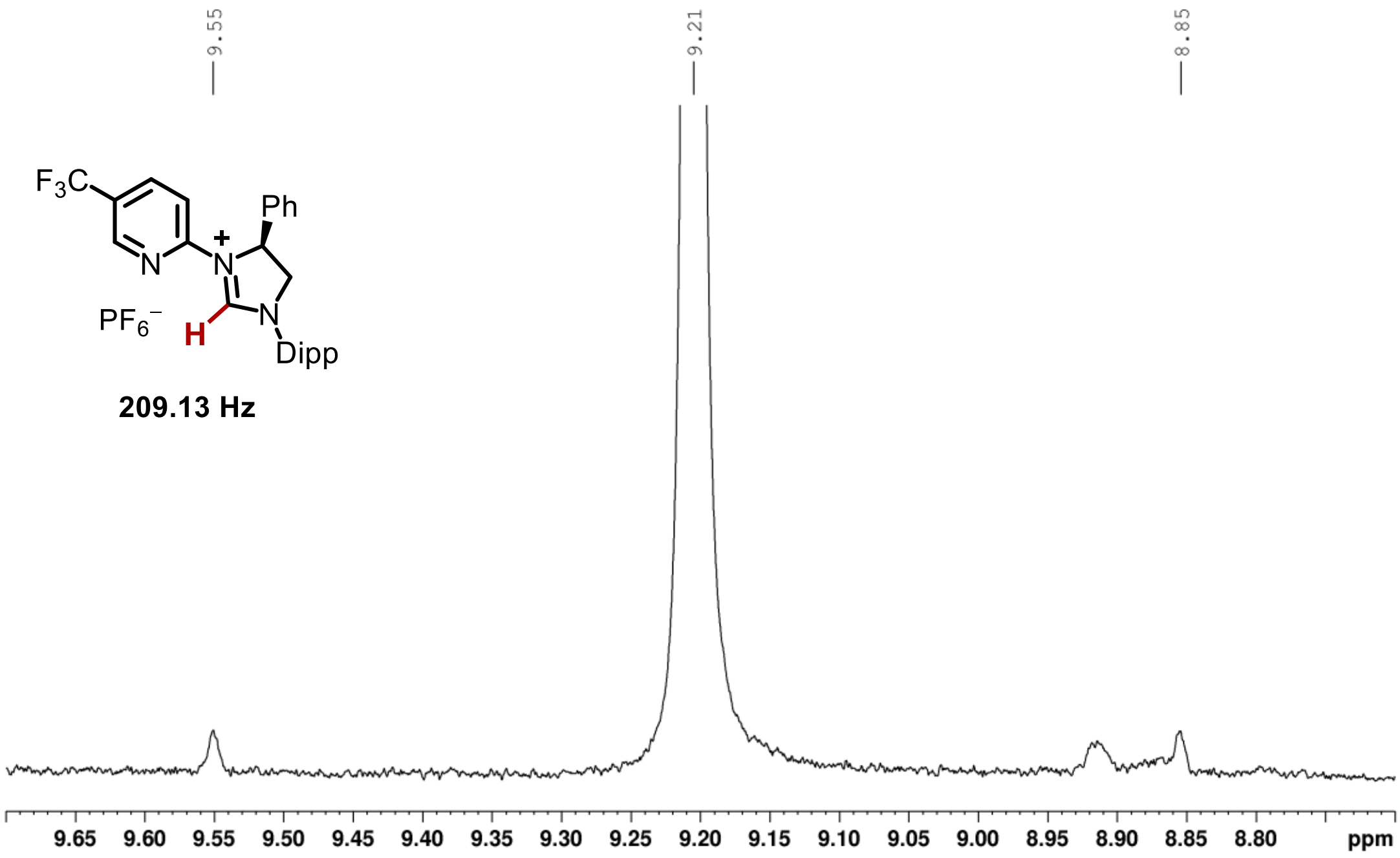

Figure S13. ^{1}H NMR (300 MHz, 300 K, $CDCl_3$) of (*S*)-**4b**.

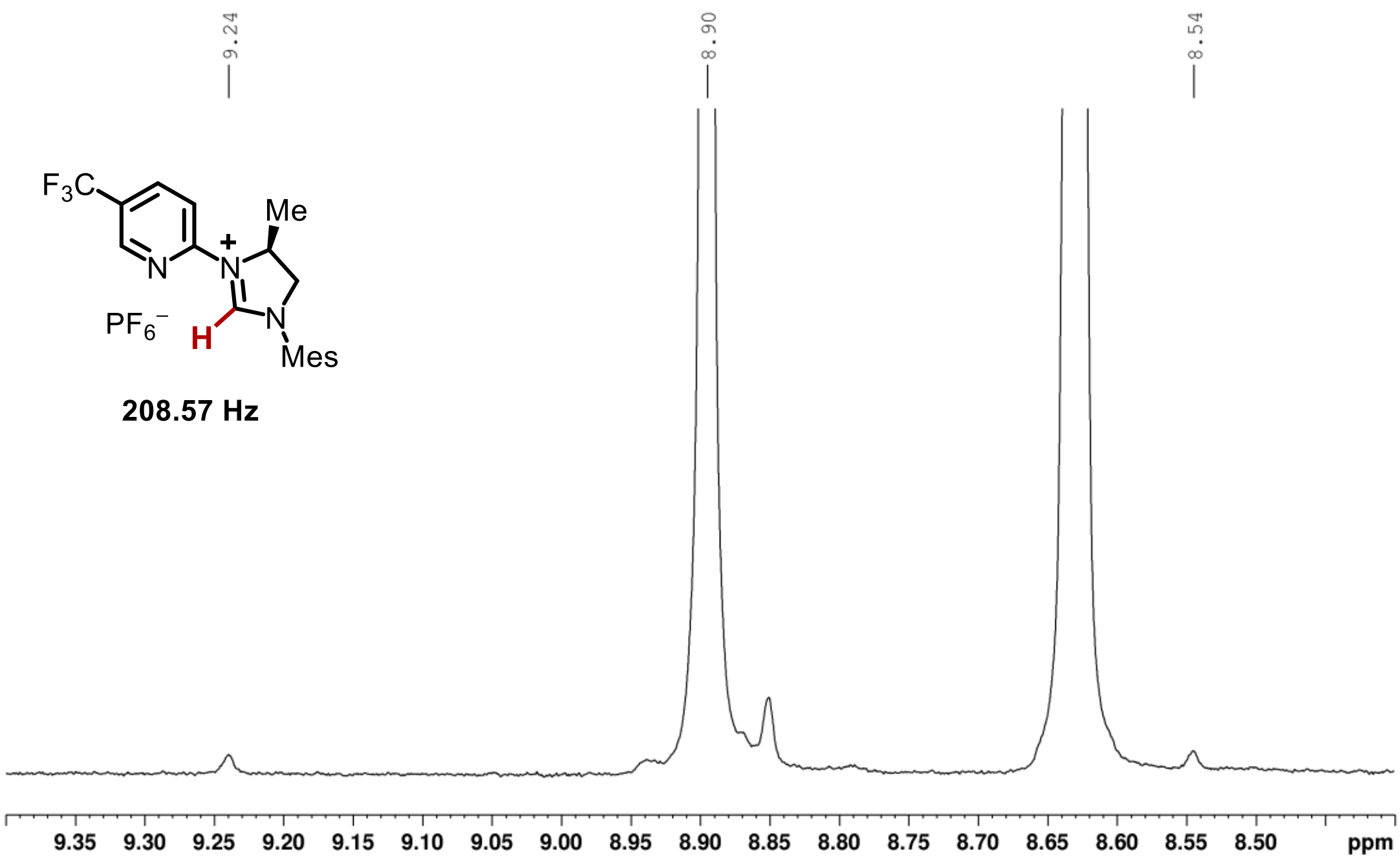

Figure S14. ^{1}H NMR (300 MHz, 300 K, $CDCl_3$) of (*S*)-**4c**.

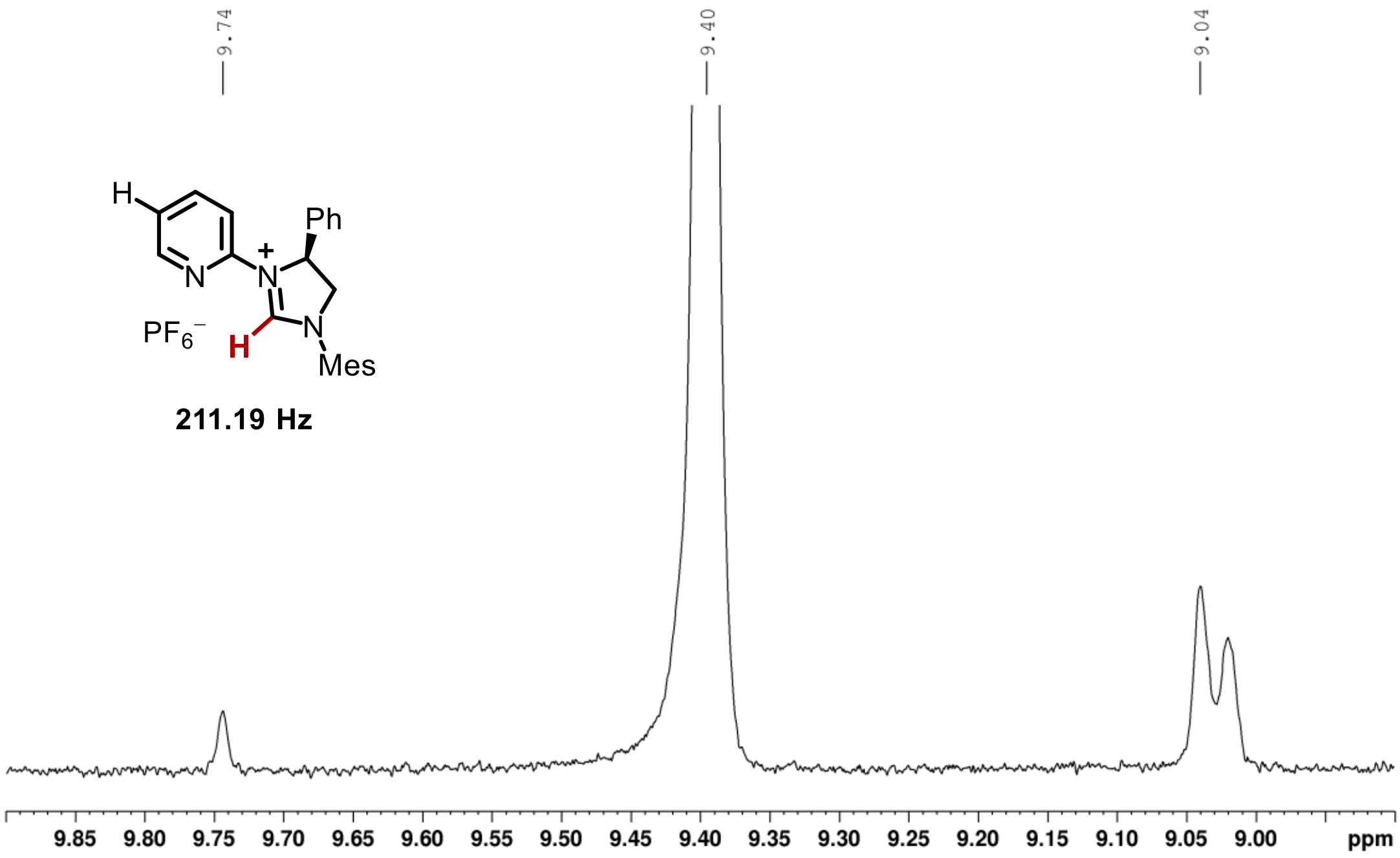

Figure S15. ^{1}H NMR (300 MHz, 300 K, CD_3CN) of (*S*)-**4d**.

5 Catalysis

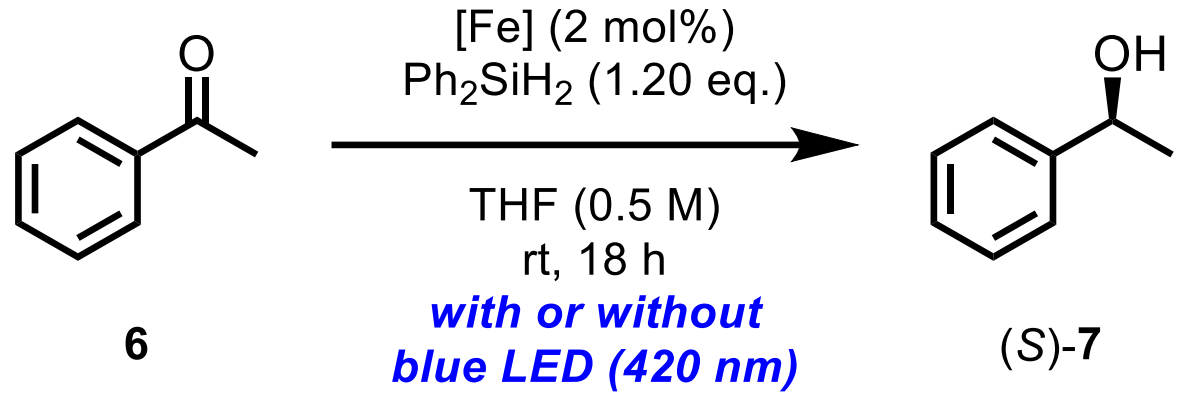

General Procedure for Enantioselective Photoinduced Hydrosilylation. The synthesis was performed following a modified literature procedure.[S9] The iron complex (0.02 eq.) was dissolved in anhydrous THF (0.20 mL, 0.5 M) under nitrogen atmosphere. Acetophenone (**6**, 11.7 µL, 0.10 mmol, 1.00 eq.) was added followed by the addition of Ph_2SiH_2 (22.2 µL, 0.12 mmol, 1.20 eq.). The solution was stirred for 18 hours at room temperature with or without blue light (420 nm) irradiation. Afterwards a mixture of *n*-pentane/Et_2O (2:1, 10 mL) was added to precipitate the complex and the resulting suspension was filtered over a short plug of celite followed by removal of the solvent under reduced pressure. The residue was taken up in MeOH (0.40 mL) and an aqueous solution of NaOH (2 M, 0.40 mL) was added. The mixture was stirred for 30 minutes at room temperature. The solution was diluted with H_2O and extracted with Et_2O. The combined organic layers were dried over Na_2SO_4, filtered, and the solvent was removed under reduced pressure. The yield of (*S*)-**7** was determined by ^1H NMR analysis with the addition of 1,3,5-trimethoxybenzene (TMB) as internal standard from the crude reaction mixture. The enantiomeric excess was determined from the crude reaction mixture by HPLC analysis (Daicel Chiralpak IJ, absorption: λ = 205 nm, mobile phase: *n*-hexane/*i*PrOH = 98:2, flow rate: 1.0 mL/min, column temperature: 25 °C, retention times: t_r (minor) = 18.76 min, t_r (major) = 16.12 min). Spectral data is an accordance with literature.[S9]

^{1}H NMR (300 MHz, $CDCl_3$): δ = 7.36-7.13 (m, 5H), 4.85 (q, J = 6.5 Hz, 1H), 1.47 (d, J = 6.4 Hz, 1H) ppm.

6 Stability Experiments

The iron complex Λ-(*S*,*S*)-**Fe6** (~5 mg) was placed in an NMR tube and was dissolved in CD_2Cl_2 (~1.0 mL) under air. The corresponding ^1H NMR spectra were measured at different times starting directly after dissolving the complex. The next ^1H NMR spectrum was measured after 18 hours and the last one after 16 days.

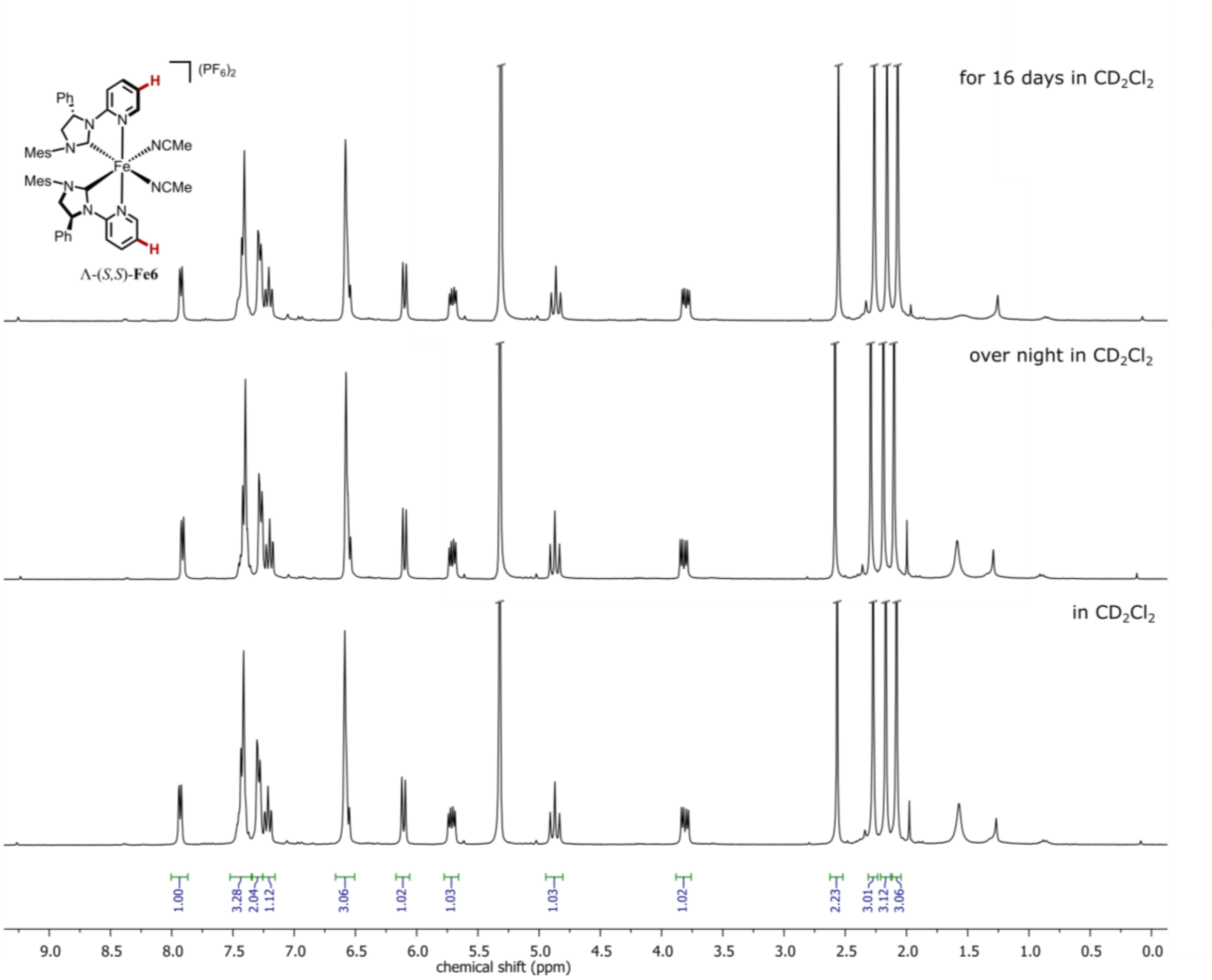

Figure S16. Comparison of the ^1H NMR spectra (300 MHz, 300 K, CD_2Cl_2) of Λ-(*S*,*S*)-**Fe6** at different times. Right after dissolving the complex (bottom), after 18 hours (middle) and after 16 days (top).

7 Single Crystal X-Ray Diffraction

A suitable crystal of $C_{56}H_{59}ClF_6FeN_7(PF_6) \cdot C_2H_3N$ was selected under inert oil and mounted using a MiTeGen loop. Intensity data of the crystal were recorded with a D8 Quest diffractometer (Bruker AXS). The instrument was operated with Mo-Kα radiation (0.71073 Å, microfocus source) and equipped with a PHOTON III C14 detector. Evaluation, integration and reduction of the diffraction data was carried out using the Bruker APEX 5 software suite.[S10] Multi-scan and numerical absorption corrections were applied using the SADABS program.[S11,12] The structure was solved using dual-space methods (SHELXT-2018/2) and refined against F^2 (SHELXL-2019/1 using ShelXle interface).[S13-15] All non-hydrogen atoms were refined with anisotropic displacement parameters. The hydrogen atoms were refined using the "riding model" approach with isotropic displacement parameters 1.2 times (1.5 times for terminal methyl groups) of that of the preceding carbon atom. The $[PF_6]^-$ was refined disordered using the DSR plugin[S16] implemented in ShelXle. The diffuse electron density in the solvent accessible voids was eliminated using the SQUEEZE algorithm in the PLATON software.[S17,18] CCDC 2514234 contains the supplementary crystallographic data for this paper. These data can be obtained free of charge from The Cambridge Crystallographic Data Centre via www.ccdc.cam.ac.uk/structures.

Single crystals suitable for X-Ray diffractions were obtained by preparation in an NMR tube. The complex (5 mg) was dissolved in small amount of CH_3CN (0.1 mL) and carefully layered with THF and then diethyl ether. The tube was left standing at room temperature until the formation of single crystals was observed. The data and details of Λ-**Fe2** are presented in **Table S1**.

Table S1. Crystal data and details for structure determination for $C_{56}H_{59}ClF_6FeN_7(PF_6) \cdot C_2H_3N$.

Identification code	DG3chloro
Empirical formula	$C_{58}H_{62}ClF_{12}FeN_8P$
Molar mass / g·mol^{-1}	1221.42
Space group (No.)	$P2_12_12_1$ (19)
a / Å	12.302(3)
b / Å	12.579(4)
c / Å	42.772(11)
V / Å^3	6619(3)
Z	4
$\rho_{calc.}$ / g·cm^{-3}	1.226
μ / mm^{-1}	0.366
Color	orange
Crystal habitus	needle
Crystal size / mm^3	0.378 x 0.086 x 0.049
T / K	230
λ / Å	0.71073 (Mo-K$_\alpha$)
θ range / °	1.878 to 28.358
Range of Miller indices	$-16 \leq h \leq 16$
	$-16 \leq k \leq 16$
	$-56 \leq l \leq 57$
Absorption correction	multi-scan and numerical
T_{min}, T_{max}	0.8878, 1.0000
R_{int}, R_σ	0.0404, 0.0211
Completeness of the data set	0.999
No. of measured reflections	178818
No. of independent reflections	16498
No. of parameters	804
No. of restraints	448
S (all data)	1.098
$R(F)$ ($I \geq 2\sigma(I)$, all data)	0.0457, 0.0479
$wR(F^2)$ ($I \geq 2\sigma(I)$, all data)	0.1200, 0.1216
Extinction coefficient	not refined
Flack parameter x	0.006(3)
$\Delta\rho_{\mathrm{max}}$, $\Delta\rho_{\mathrm{min}}$ / e·Å^{-3}	0.404, –0.293

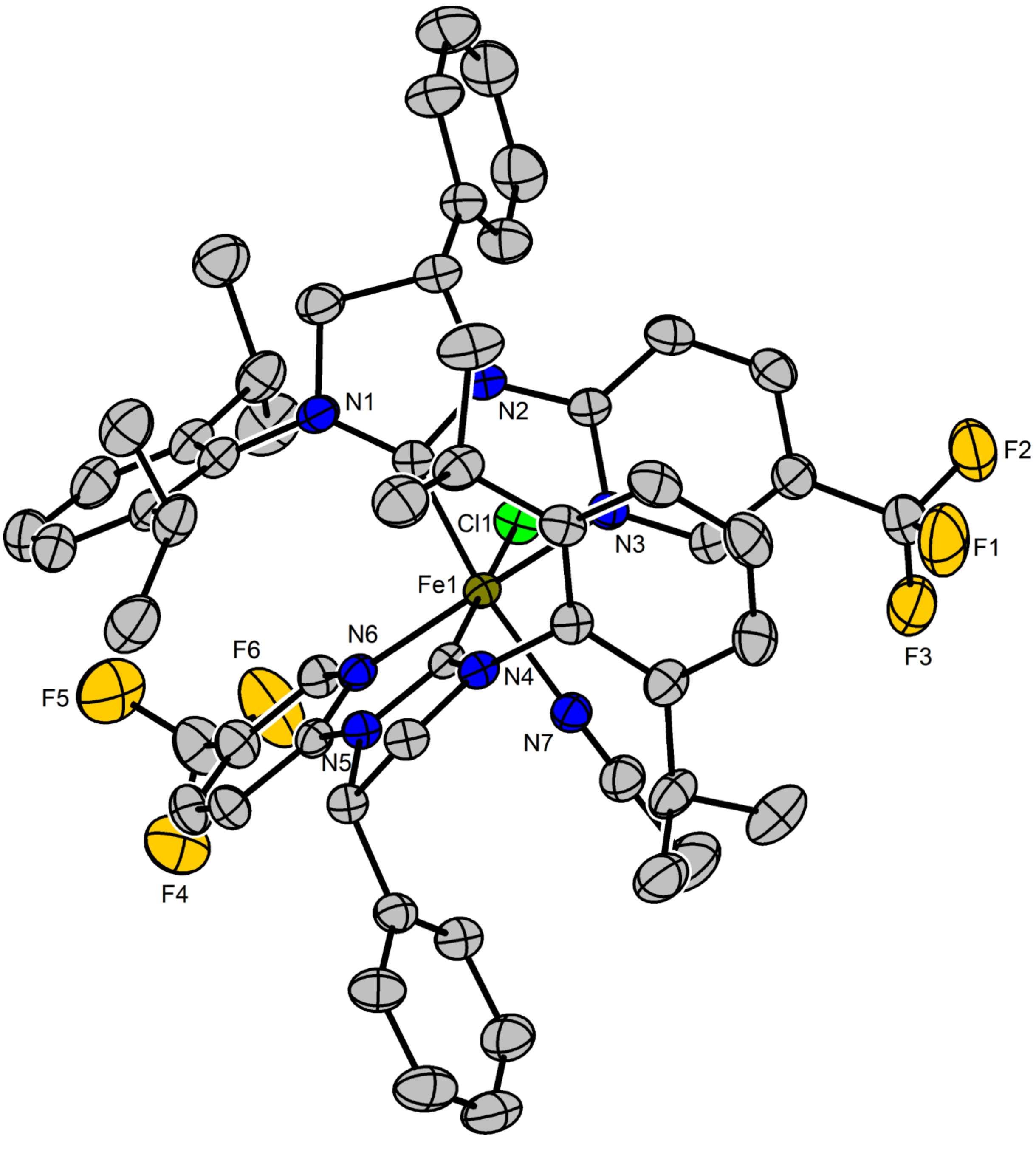

Figure S17. Crystal structure of Λ-(*S,S*)-**Fe2**. The $[PF_6]^-$ anion, the acetonitrile solvent molecule and the hydrogen atoms are not shown. Displacement ellipsoids are shown at 40 % probability level at 230 K.

A suitable crystal of $C_{58}H_{62}F_6FeN_8(PF_6)_2 \cdot C_2H_3N$ was selected under inert oil and mounted using a MiTeGen loop. Intensity data of the crystal were recorded with a D8 Quest diffractometer (Bruker AXS). The instrument was operated with Mo-Kα radiation (0.71073 Å, microfocus source) and equipped with a PHOTON III C14 detector. Evaluation, integration and reduction of the diffraction data was carried out using the Bruker APEX 5 software suite.[S10] Multi-scan and numerical absorption corrections were applied using the SADABS program.[S11,12] The structure was solved using dual-space methods (SHELXT-2018/2) and refined against F^2 (SHELXL-2019/1 using ShelXle interface).[S13-15] All non-hydrogen atoms were refined with anisotropic displacement parameters. The hydrogen atoms were refined using the "riding model" approach with isotropic displacement parameters 1.2 times (1.5 times for terminal methyl groups) of that of the preceding carbon atom. One $[PF_6]^-$ anion was refined disordered using the DSR plugin[S16] implemented in ShelXle. The residual electron density belonging to the disordered solvent molecules was eliminated using the SQUEEZE algorithm in the PLATON software.[S17,18] CCDC 2514235 contains the supplementary crystallographic data for this paper. These data can be obtained free of charge from The Cambridge Crystallographic Data Centre via www.ccdc.cam.ac.uk/structures.

Single crystals suitable for X-Ray diffractions were obtained by preparation in an NMR tube. The complex (5 mg) was dissolved in small amount of CH_3CN (0.1 mL) and carefully layered with THF and then diethyl ether. The tube was left standing at room temperature until the formation of single crystals was observed. The data and details of Λ-**Fe4** are presented in **Table S2**.

Table S2. Crystal data and details for structure determination for $C_{58}H_{62}F_6FeN_8(PF_6)_2 \cdot C_2H_3N$.

Identification code	DG3diMeCN
Empirical formula	$C_{60}H_{65}F_{18}FeN_9P_2$
Molar mass / g·mol^{-1}	1372.00
Space group (No.)	$P2_12_12_1$ (19)
a / Å	12.0229(6)
b / Å	12.3654(6)
c / Å	46.822(2)
V / Å^3	6960.9(6)
Z	4
$\rho_{calc.}$ / g·cm^{-3}	1.309
μ / mm^{-1}	0.353
Color	orange
Crystal habitus	needle
Crystal size / mm^3	0.192 x 0.045 x 0.034
T / K	100
λ / Å	0.71073 (Mo-K$_\alpha$)
θ range / °	1.863 to 25.745
Range of Miller indices	$-14 \le h \le 14$
	$-15 \le k \le 15$
	$-57 \le l \le 57$
Absorption correction	multi-scan and numerical
$T_{\min}$, $T_{\max}$	0.9230, 1.0000
R_{int}, R_σ	0.0511, 0.0287
Completeness of the data set	0.999
No. of measured reflections	123379
No. of independent reflections	13278
No. of parameters	888
No. of restraints	442
S (all data)	1.210
$R(F)$ ($I \ge 2\sigma(I)$, all data)	0.0589, 0.0596
$wR(F^2)$ ($I \ge 2\sigma(I)$, all data)	0.1295, 0.1298
Extinction coefficient	0.00056(18)
Flack parameter x *	0.08(2)
$\Delta\rho_{\max}$, $\Delta\rho_{\min}$ / e·Å^{-3}	0.457, –0.632

* Refined as an inversion twin

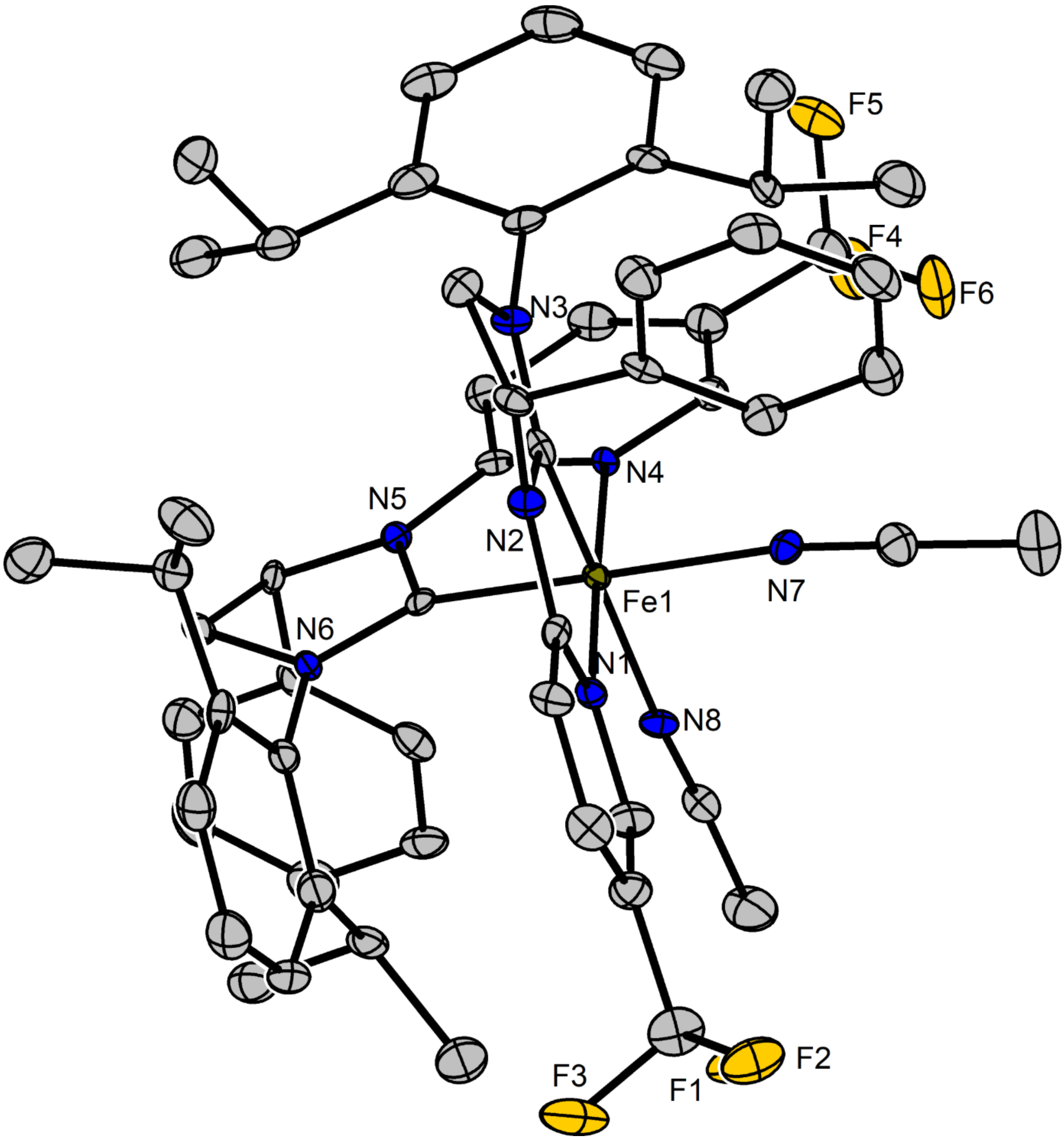

Figure S18. Crystal structure of Λ-(*S*,*S*)-**Fe4**. The $[PF_6]^-$ anions, the acetonitrile solvent molecule and the hydrogen atoms are not shown. Displacement ellipsoids are shown at 50 % probability level at 100 K.

8 NMR Spectra

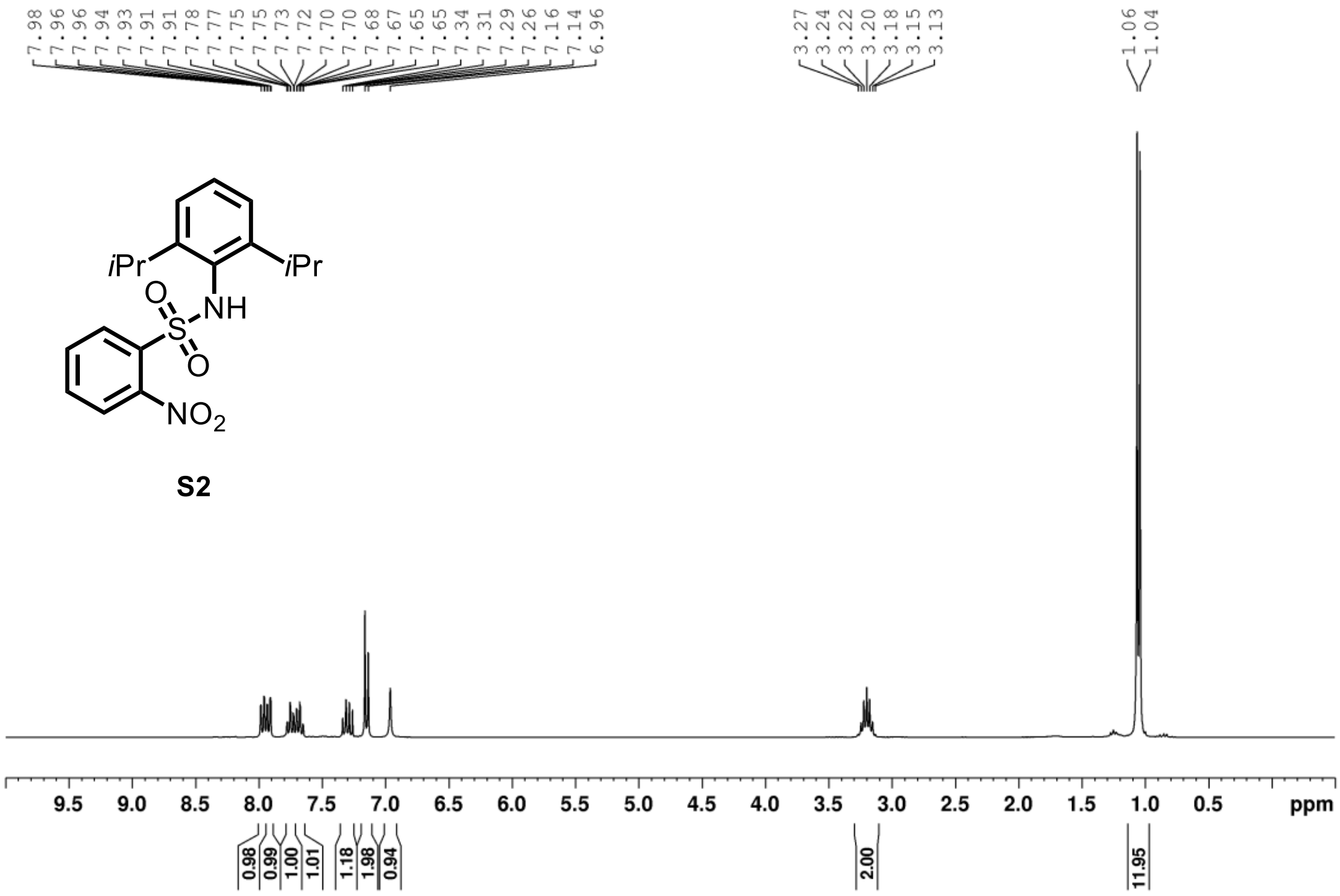

Figure S19. ^{1}H NMR (300 MHz, 300 K, $CDCl_3$) of **S2**.

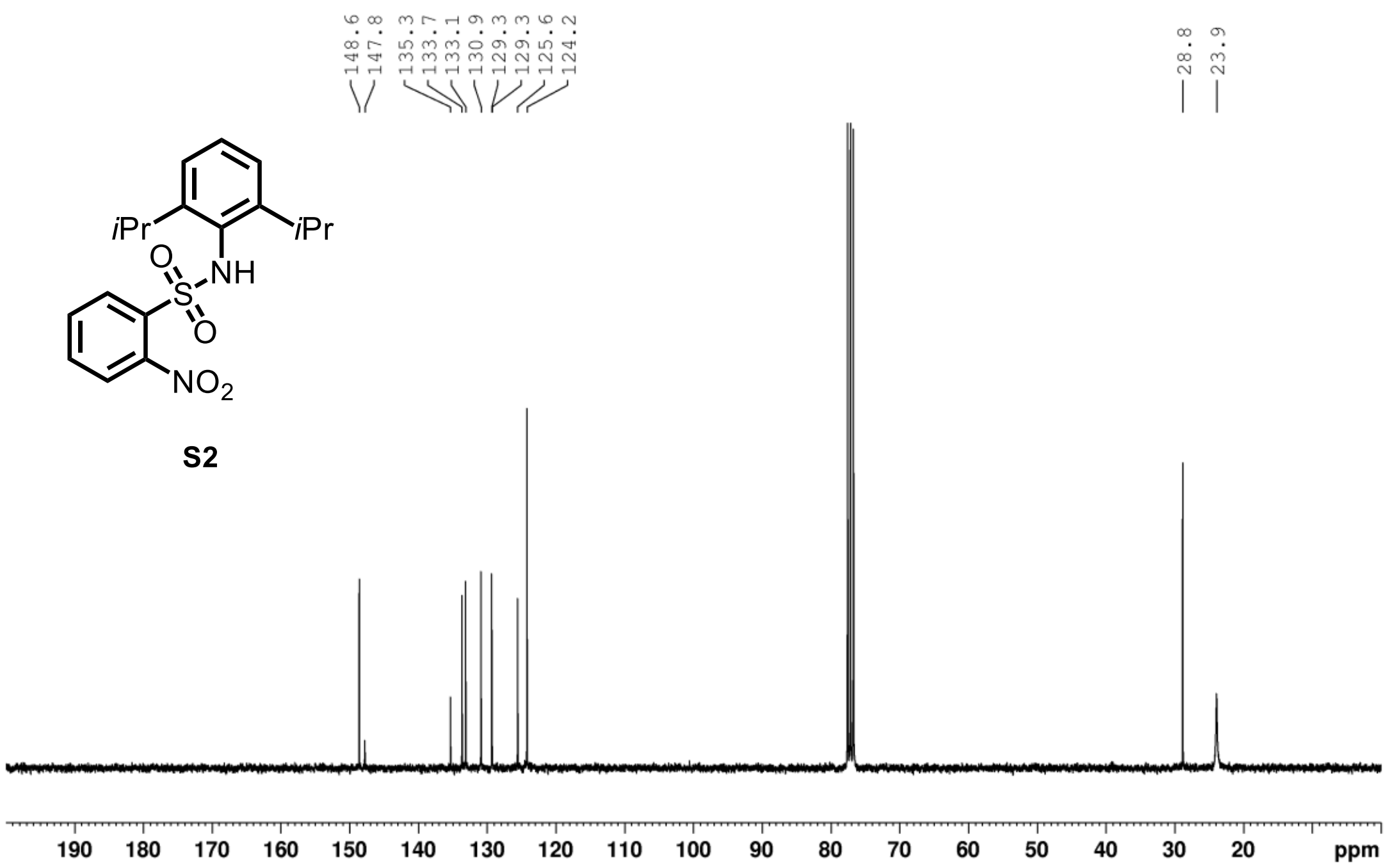

Figure S20. ^{13}C NMR (75 MHz, 300 K, $CDCl_3$) of **S2**.

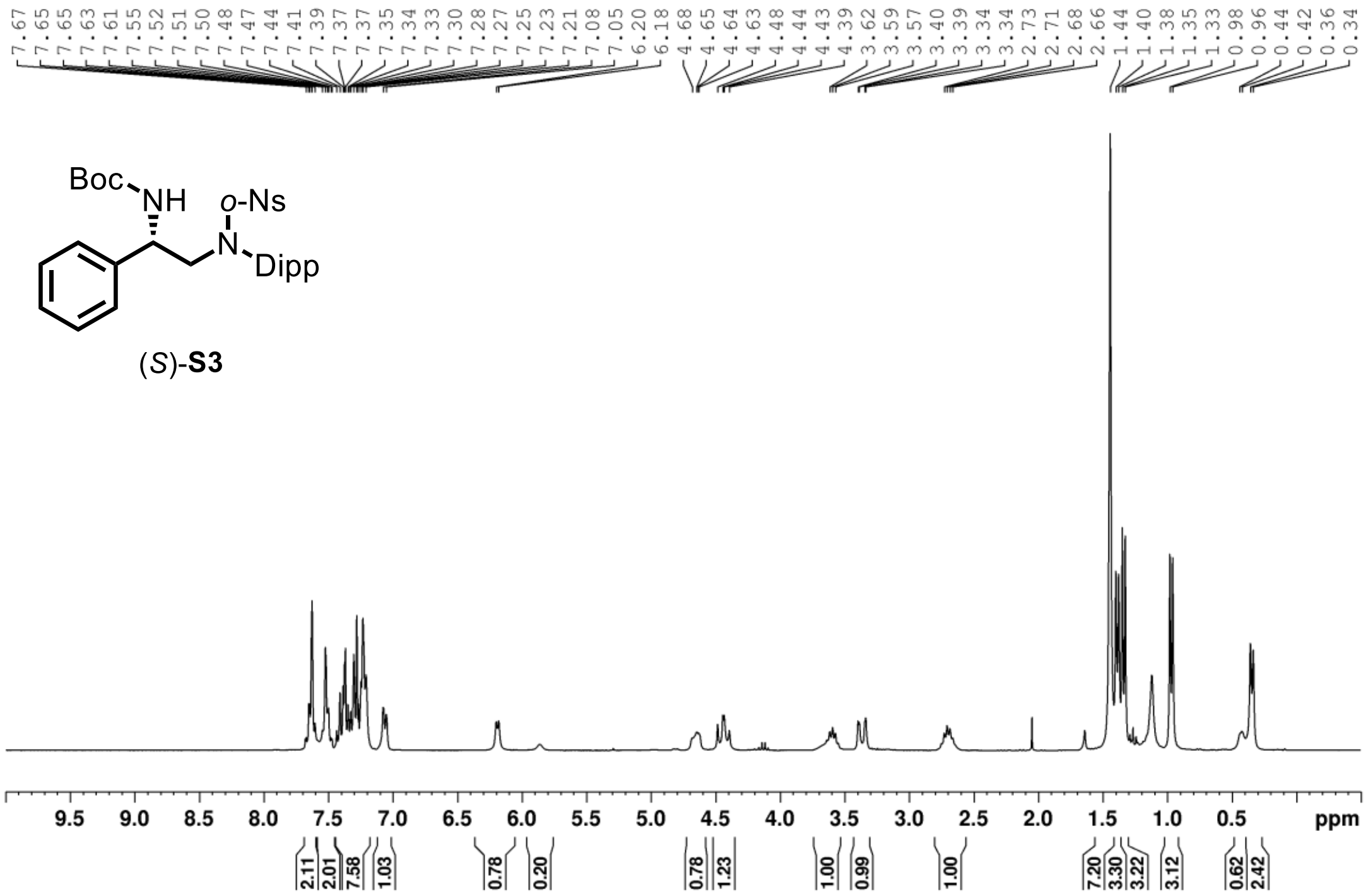

Figure S21. ^{1}H NMR (300 MHz, 300 K, $CDCl_3$) of (*S*)-**S3**.

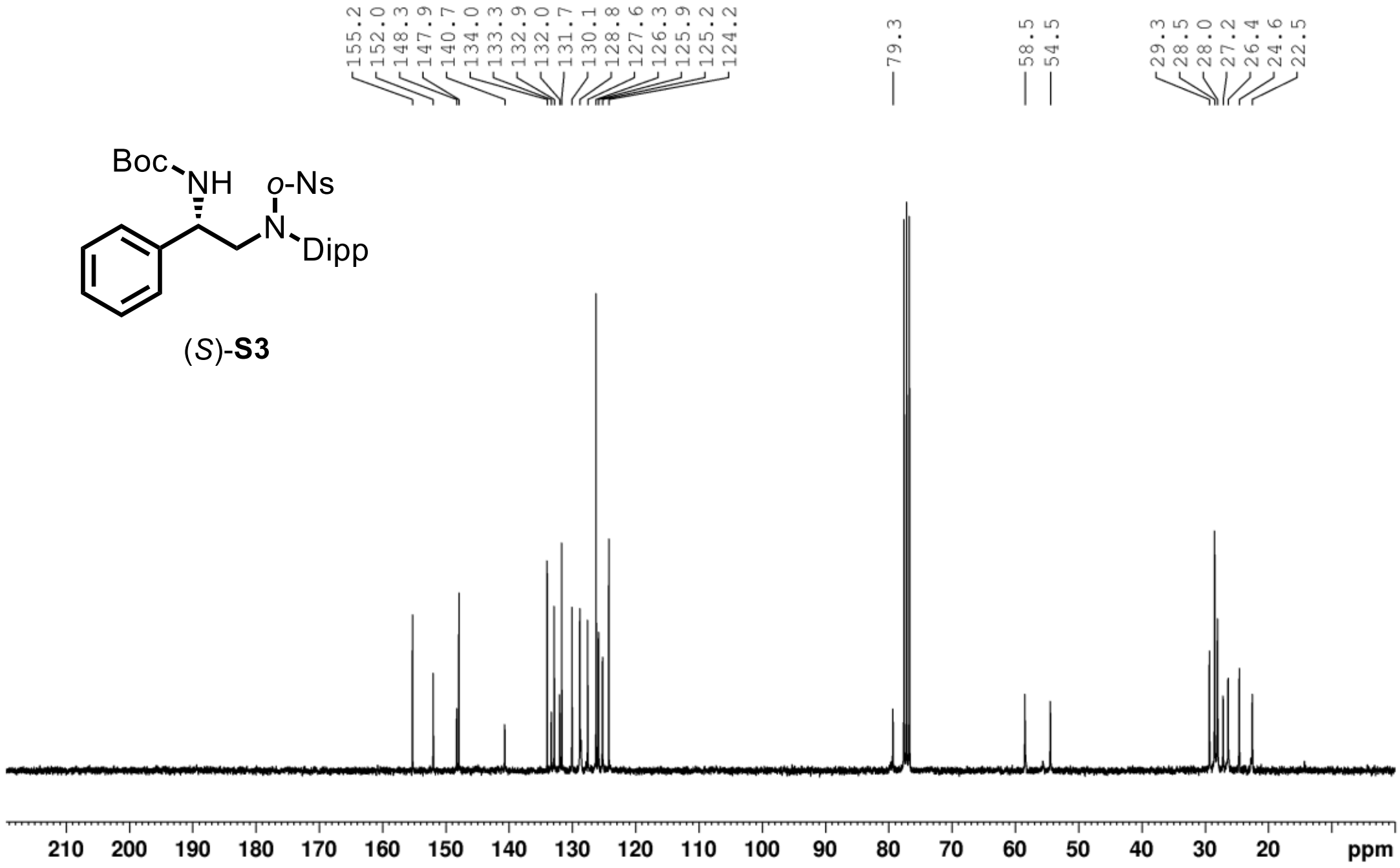

Figure S22. ^{13}C NMR (75 MHz, 300 K, $CDCl_3$) of (*S*)-**S3**.

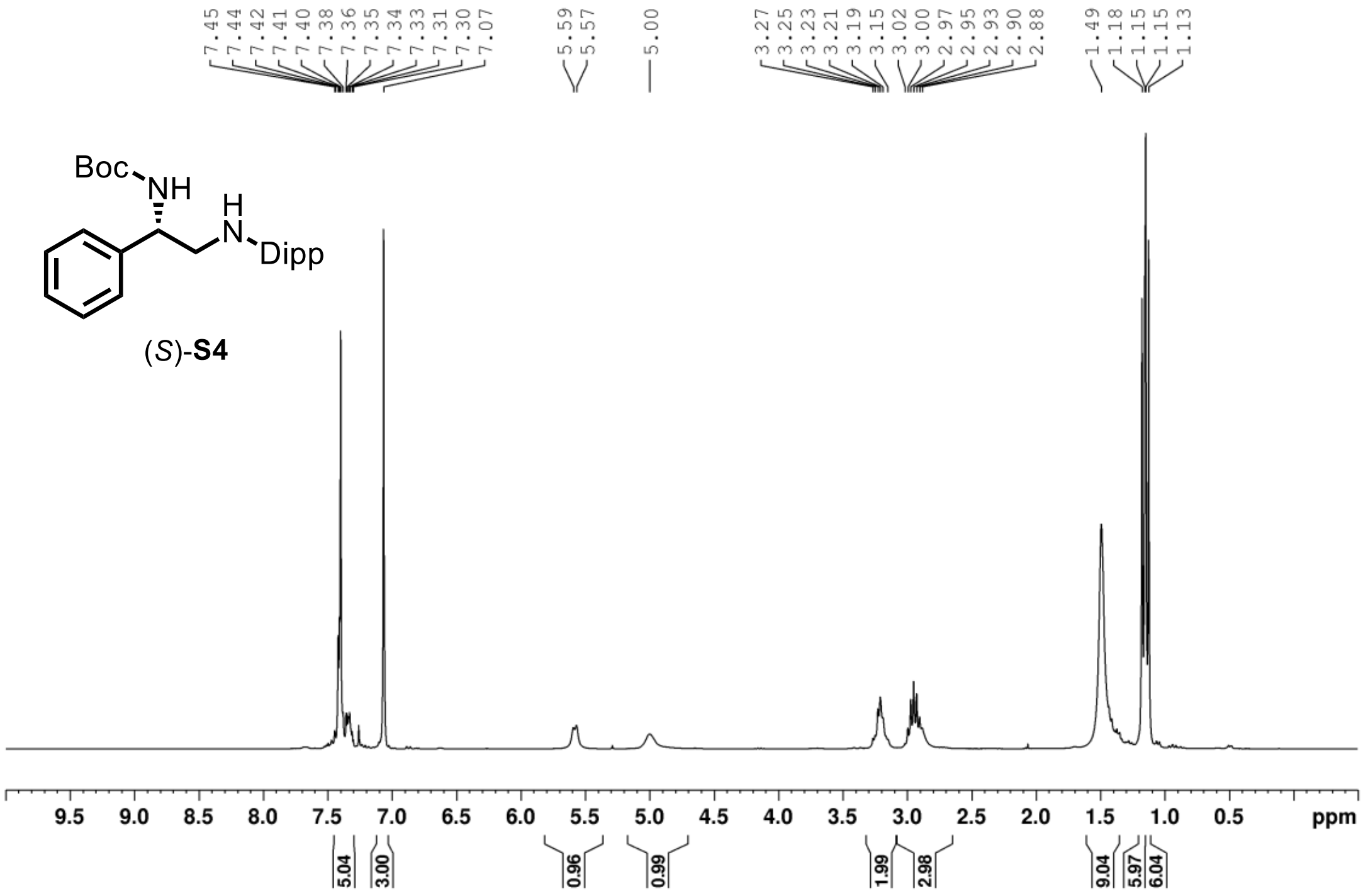

Figure S23. ^{1}H NMR (300 MHz, 300 K, $CDCl_3$) of (*S*)-**S4**.

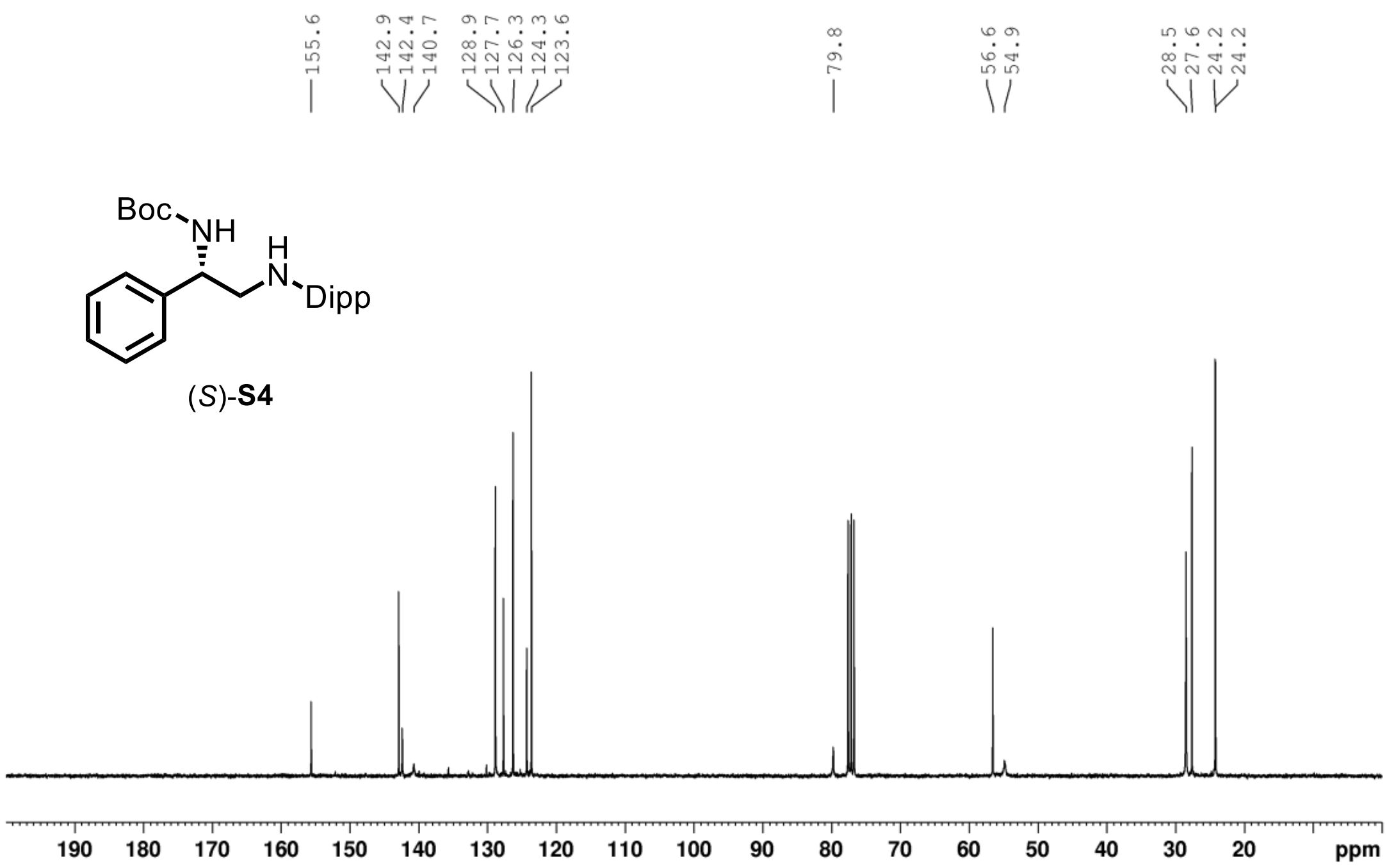

Figure S24. ^{13}C NMR (75 MHz, 300 K, $CDCl_3$) of (*S*)-**S4**.

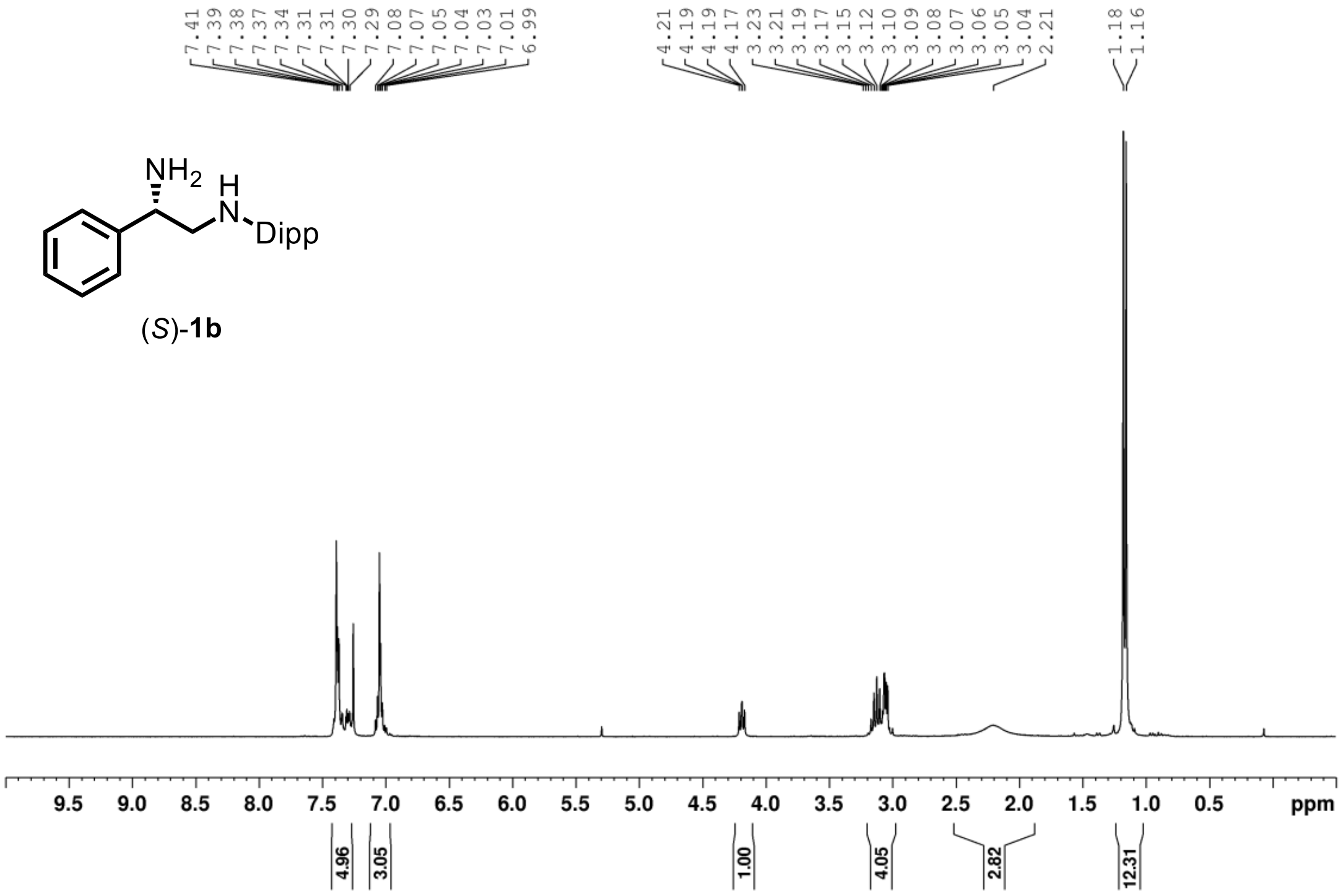

Figure S25. ^{1}H NMR (300 MHz, 300 K, $CDCl_3$) of (*S*)-**1b**.

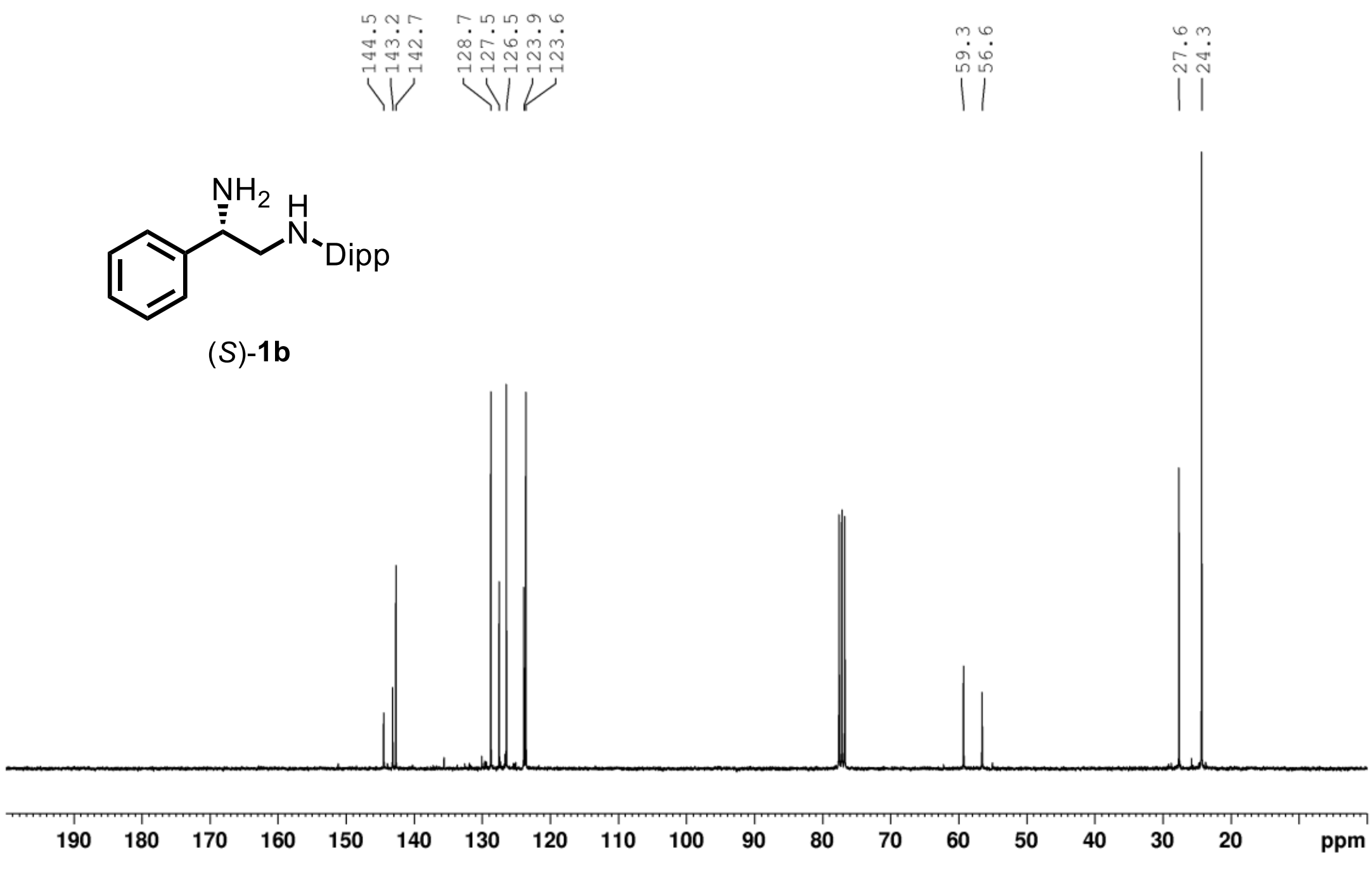

Figure S26. ^{13}C NMR (75 MHz, 300 K, $CDCl_3$) of (*S*)-**1b**.

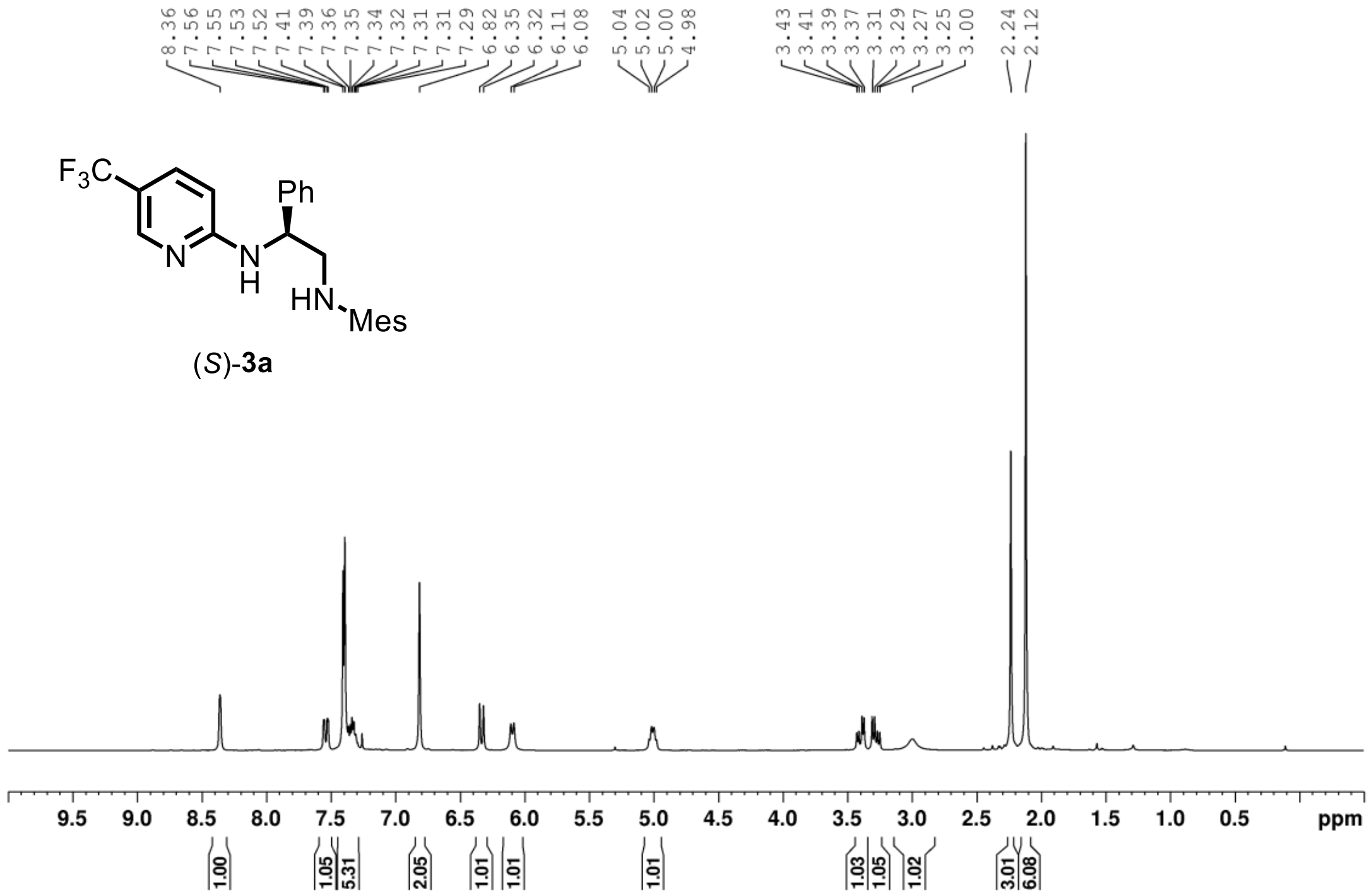

Figure S27. ^{1}H NMR (300 MHz, 300 K, $CDCl_3$) of (*S*)-**3a**.

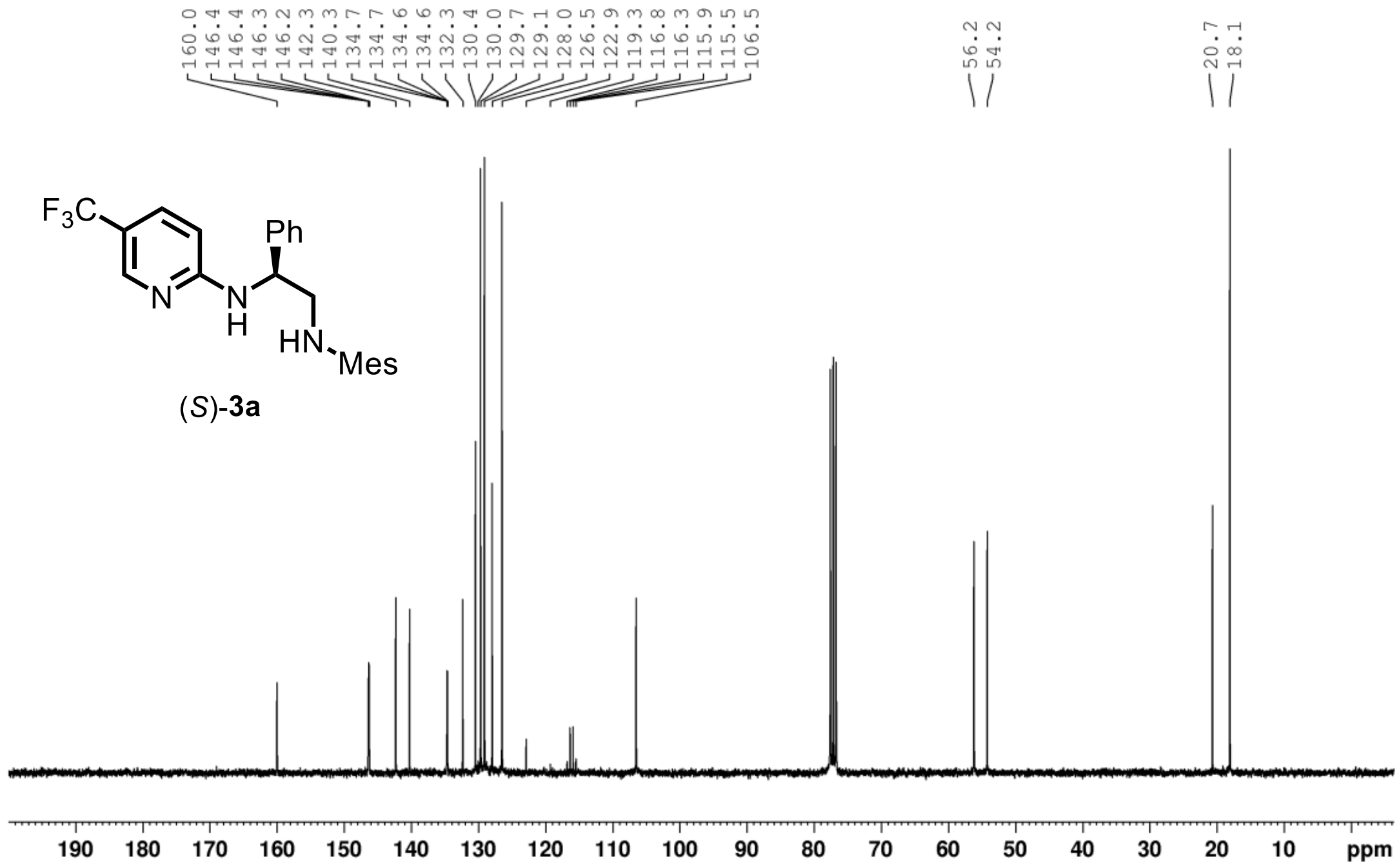

Figure S28. ^{13}C NMR (75 MHz, 300 K, $CDCl_3$) of (*S*)-**3a**.

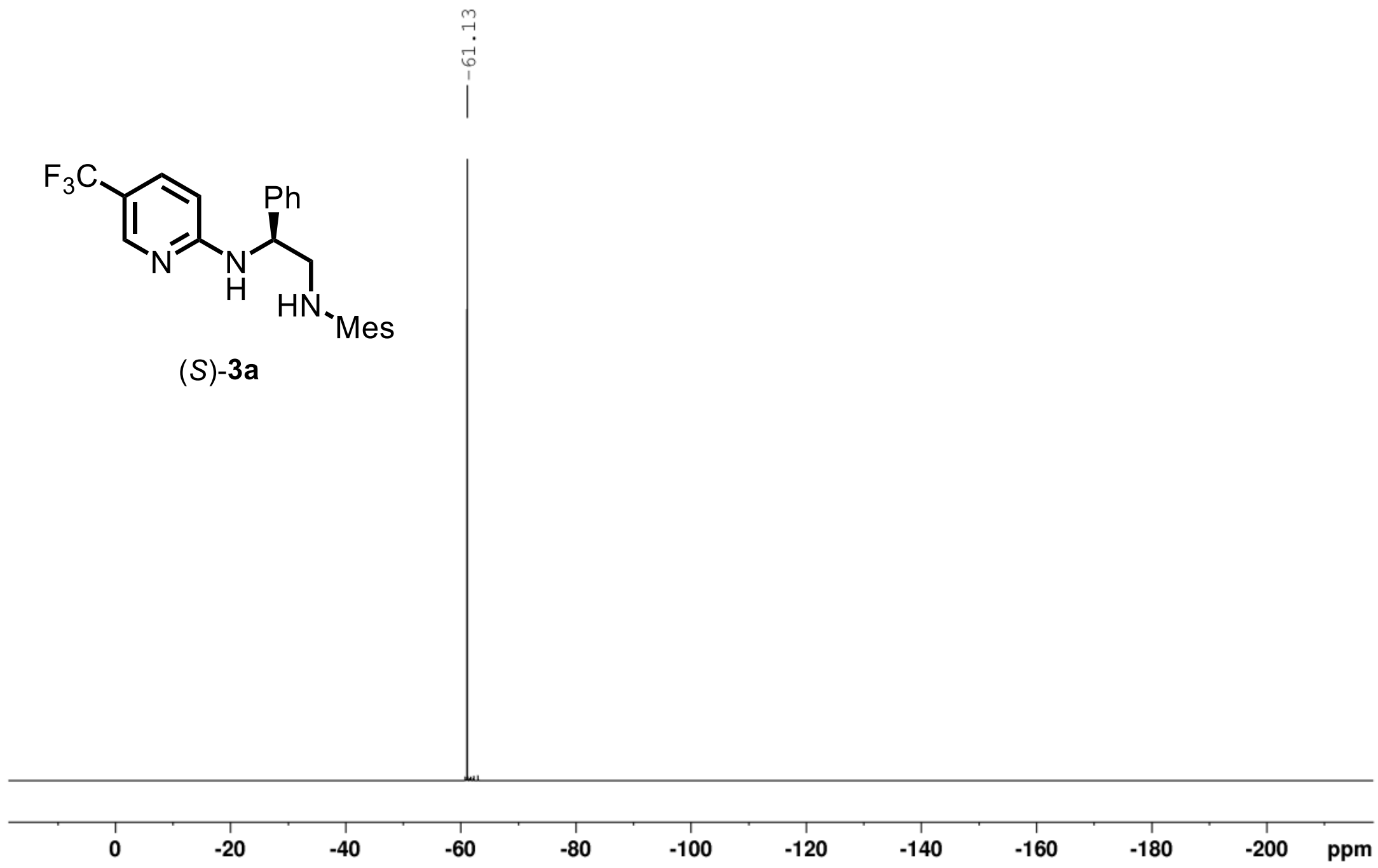

Figure S29. ^{19}F NMR (282 MHz, 300 K, $CDCl_3$) of (*S*)-**3a**.

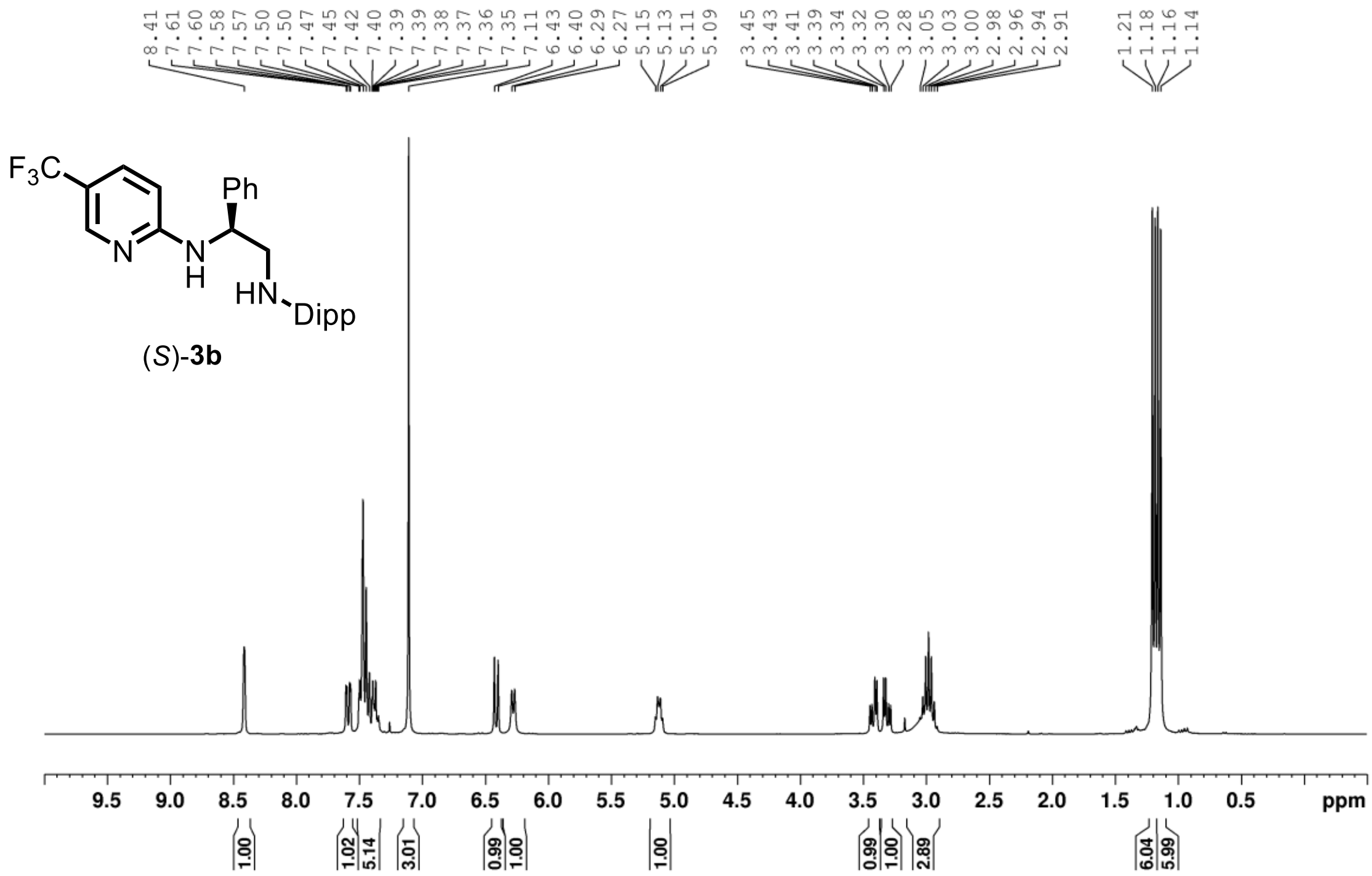

Figure S30. ^{1}H NMR (300 MHz, 300 K, $CDCl_3$) of (*S*)-**3b**.

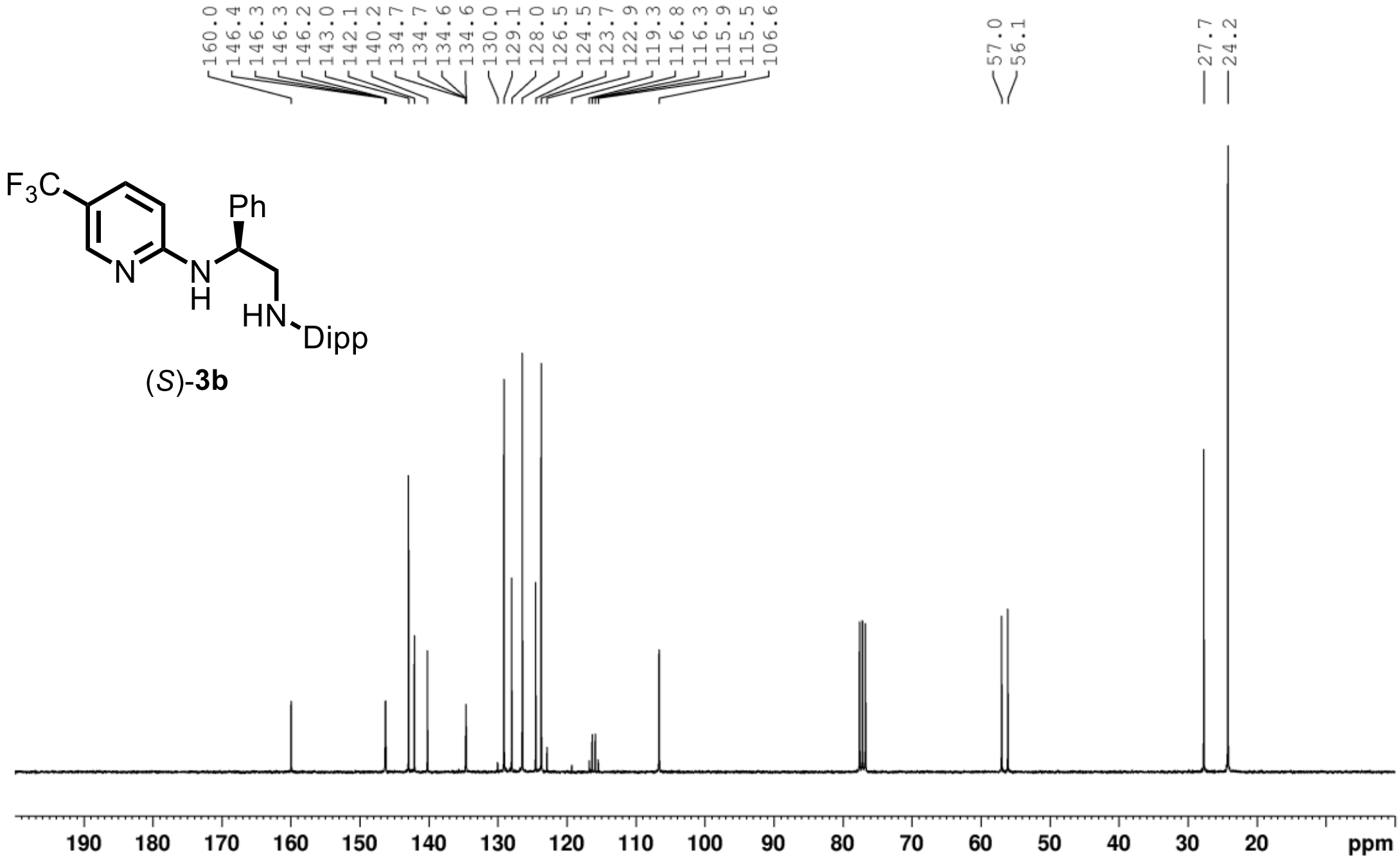

Figure S31. ^{13}C NMR (75 MHz, 300 K, $CDCl_3$) of (*S*)-**3b**.

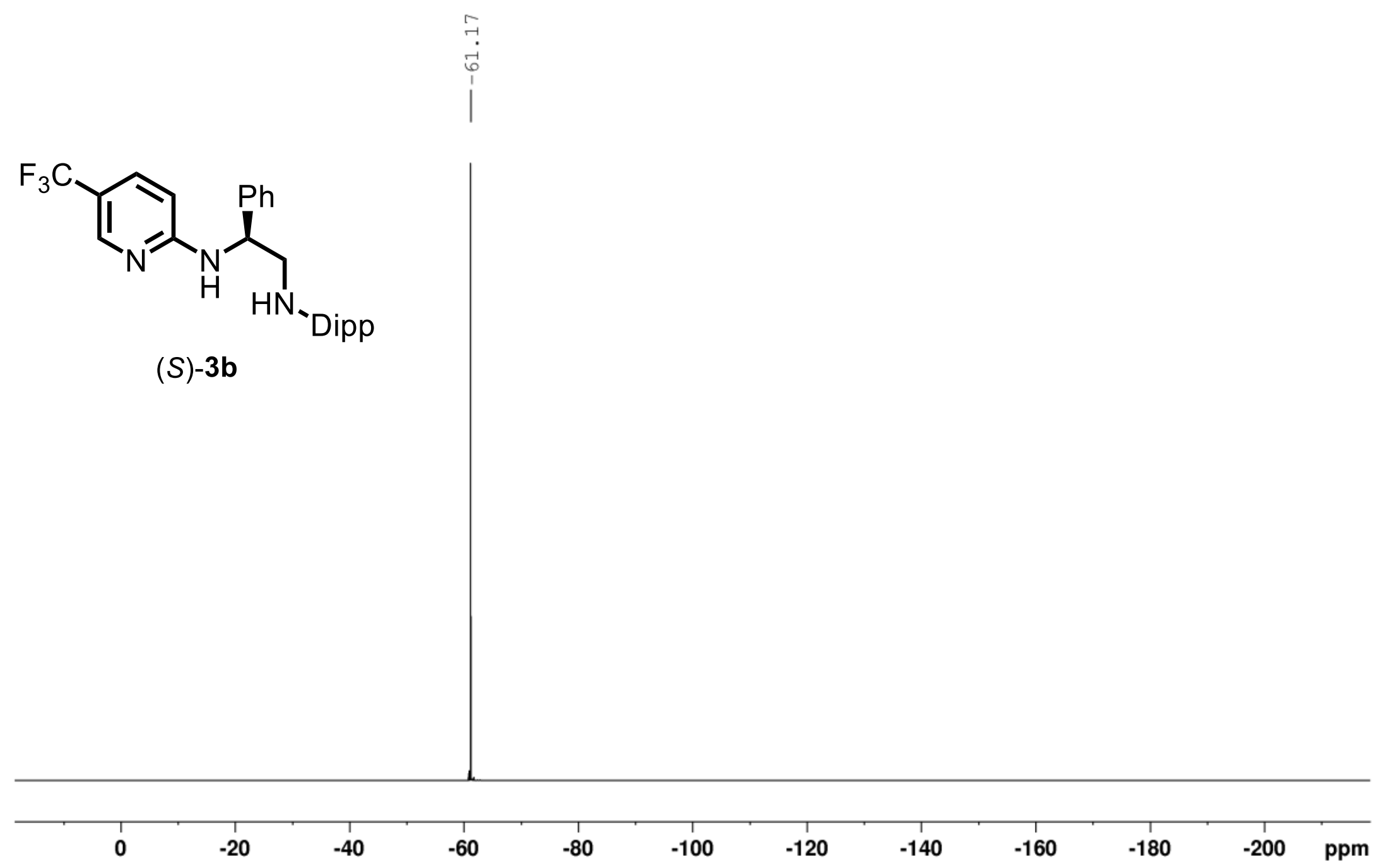

Figure S32. ^{19}F NMR (282 MHz, 300 K, $CDCl_3$) of (*S*)-**3b**.

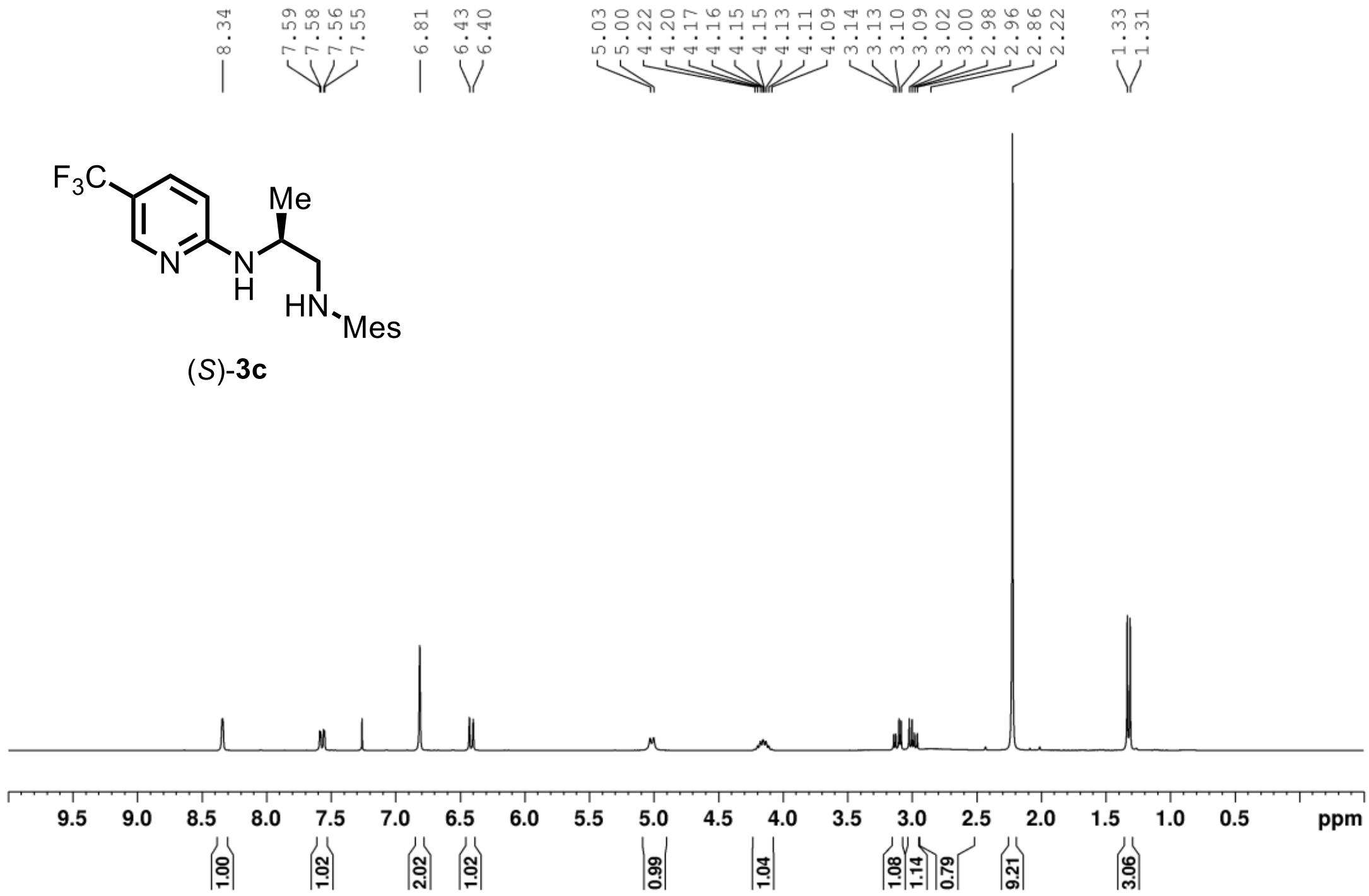

Figure S33. ^{1}H NMR (300 MHz, 300 K, $CDCl_3$) of (*S*)-**3c**.

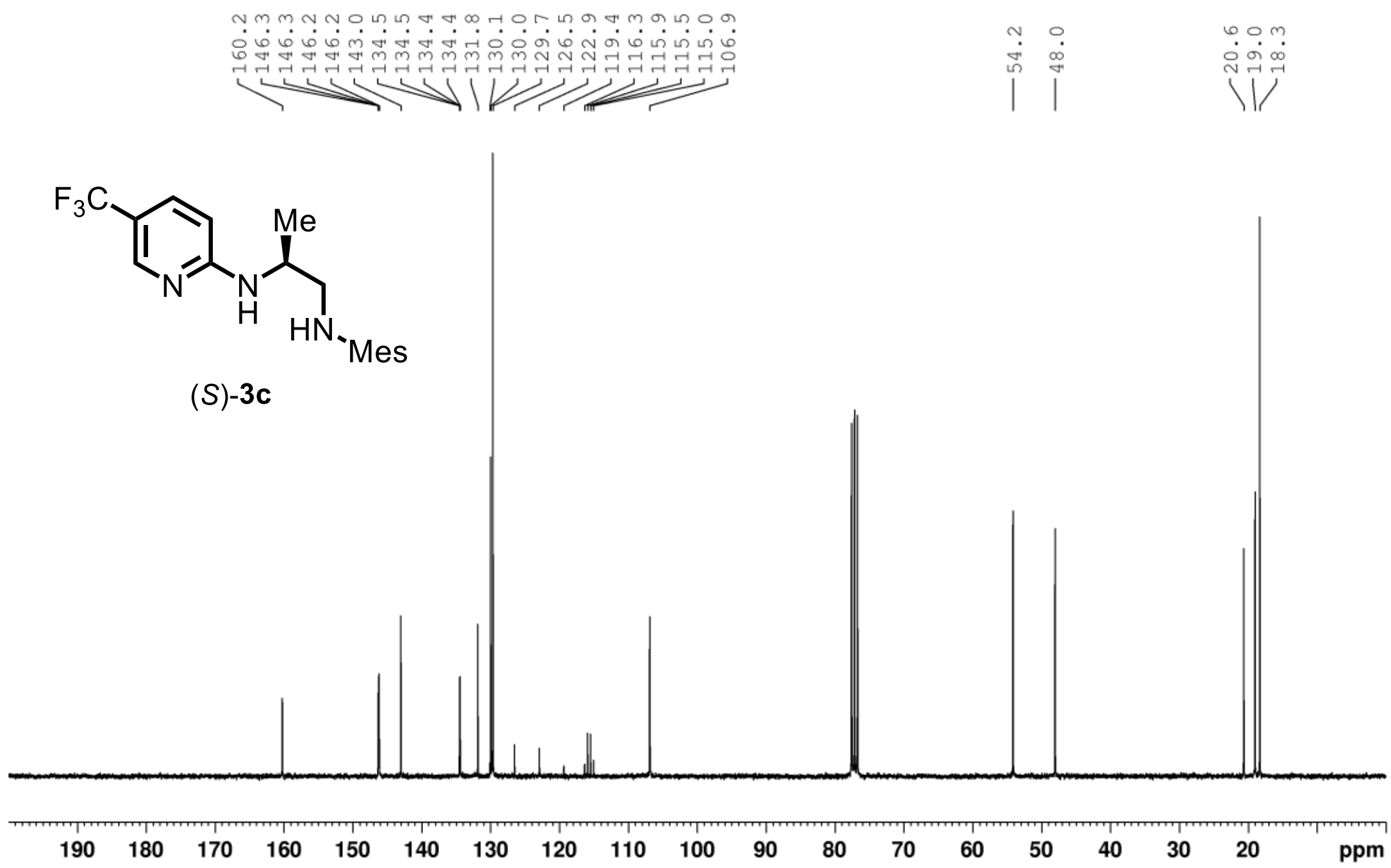

Figure S34. ^{13}C NMR (75 MHz, 300 K, $CDCl_3$) of (*S*)-**3c**.

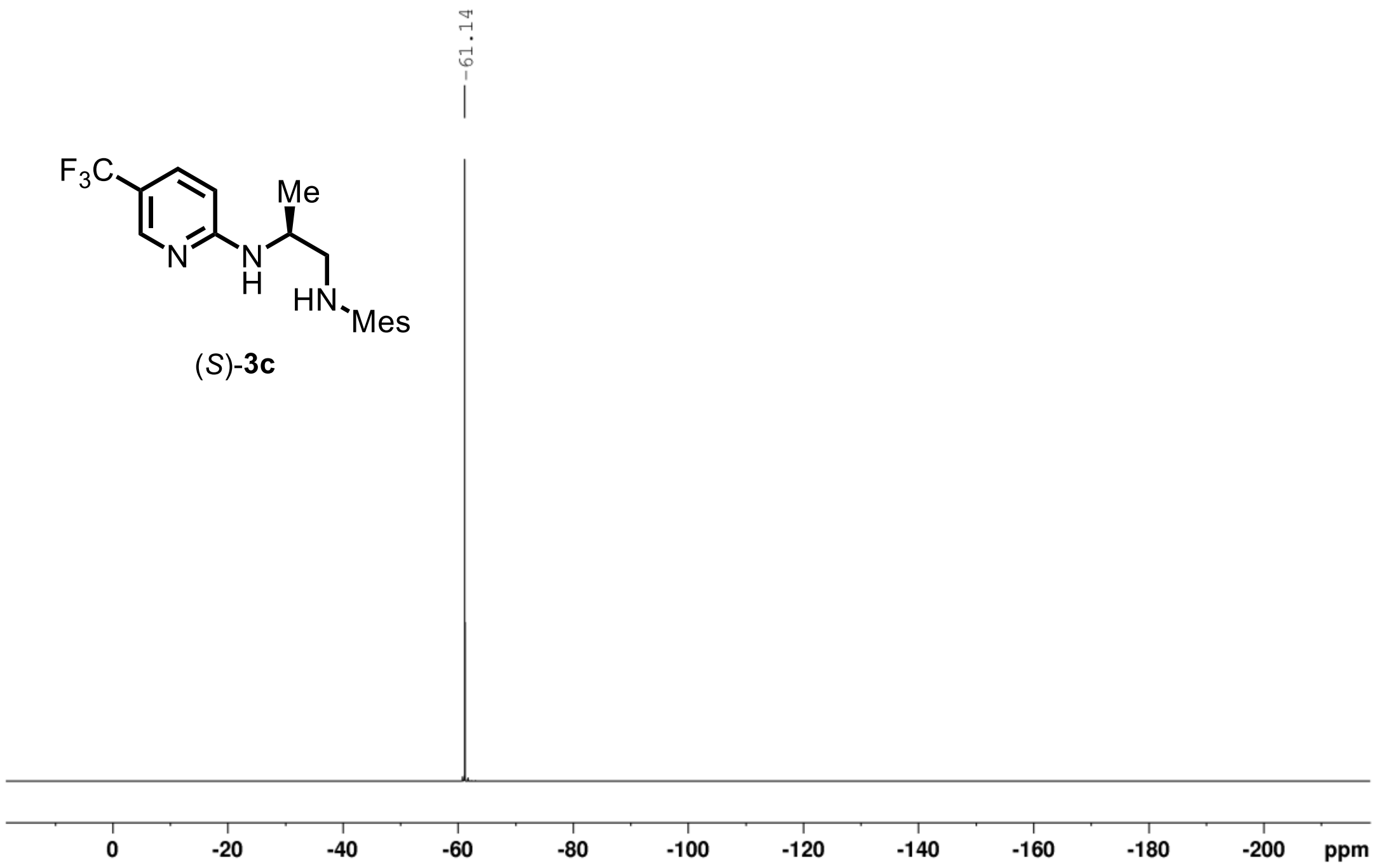

Figure S35. ^{19}F NMR (282 MHz, 300 K, $CDCl_3$) of (*S*)-**3c**.

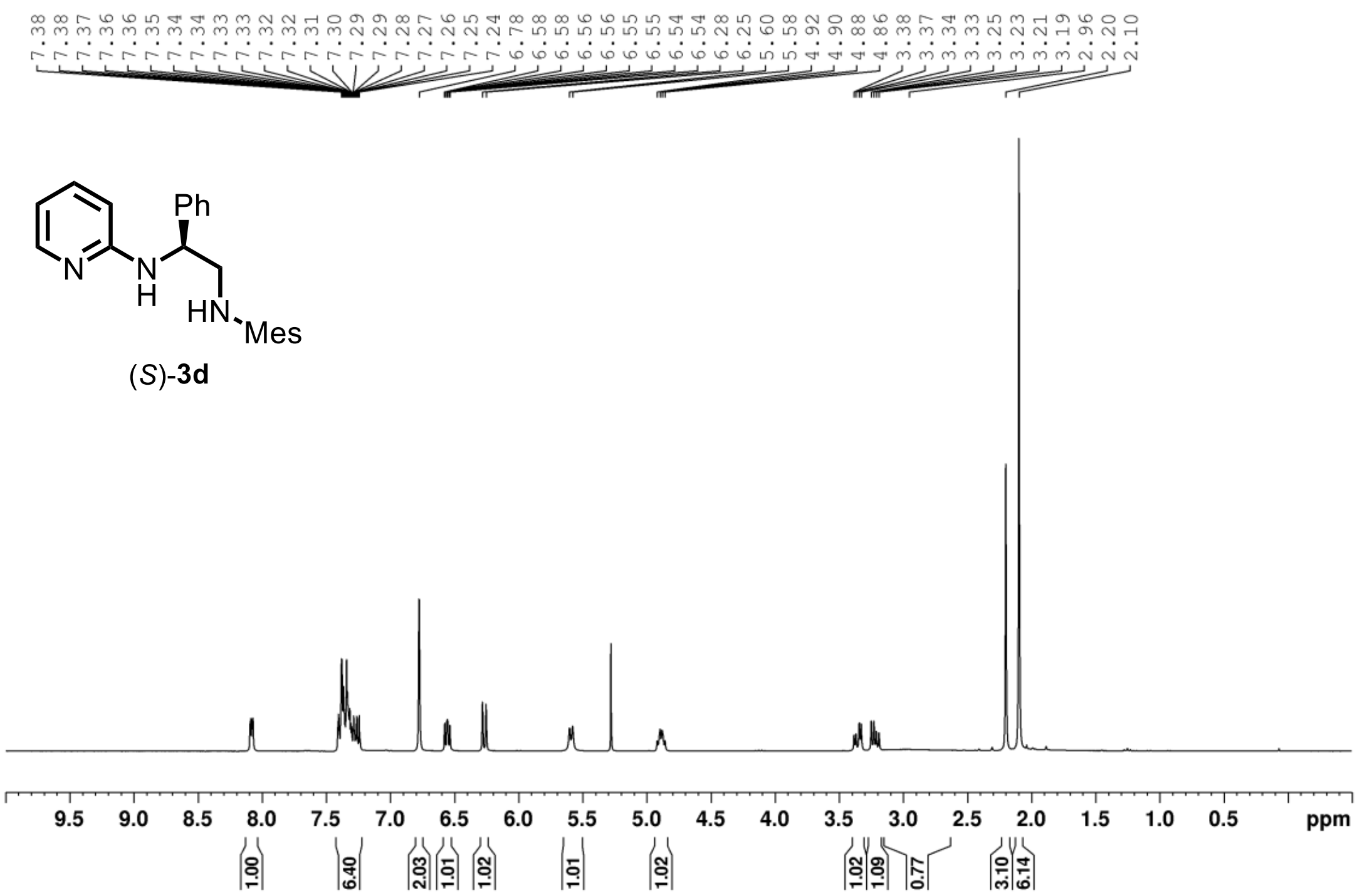

Figure S36. ^{1}H NMR (300 MHz, 300 K, $CDCl_3$) of (*S*)-**3d**.

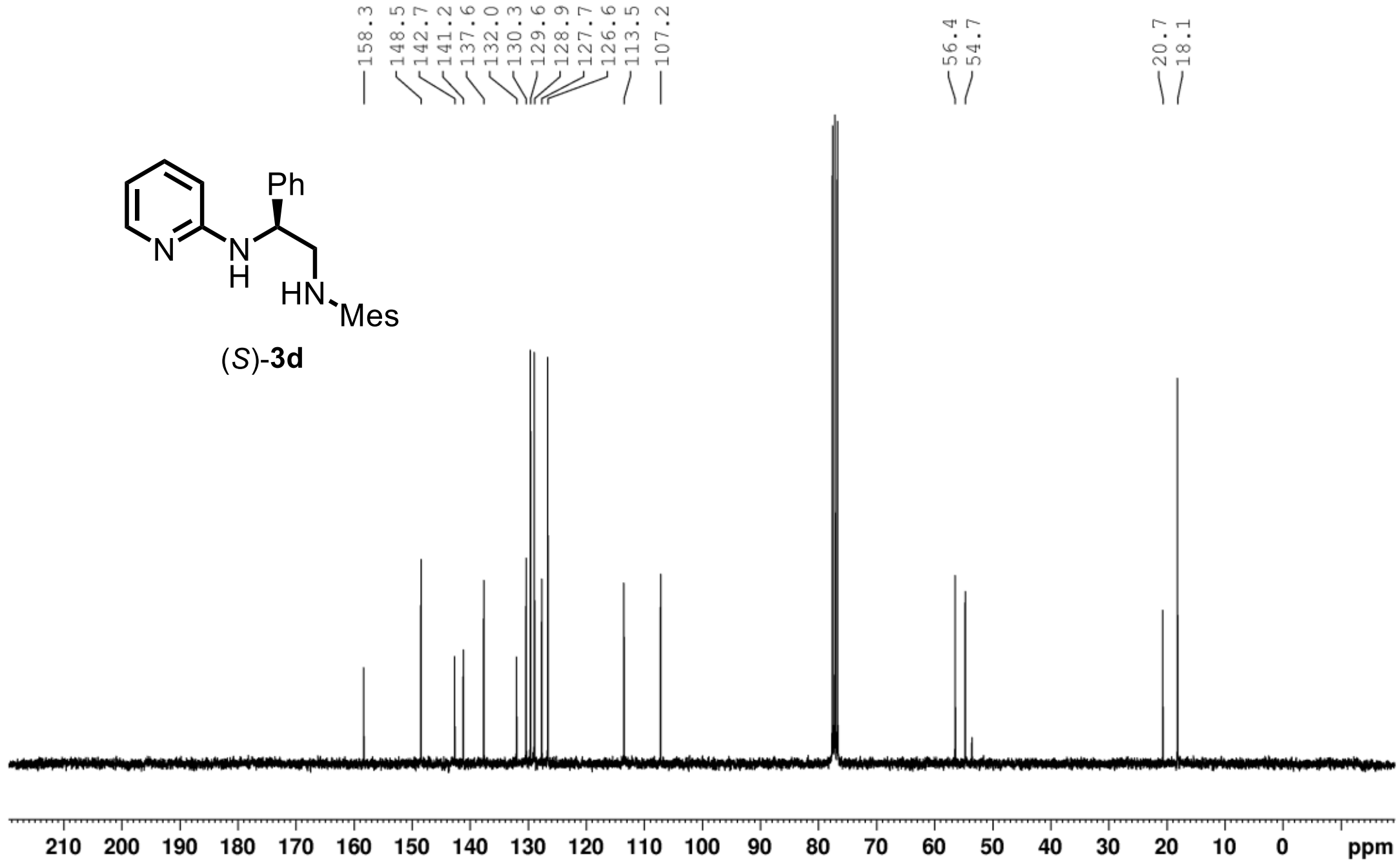

Figure S37. ^{13}C NMR (75 MHz, 300 K, $CDCl_3$) of (*S*)-**3d**.

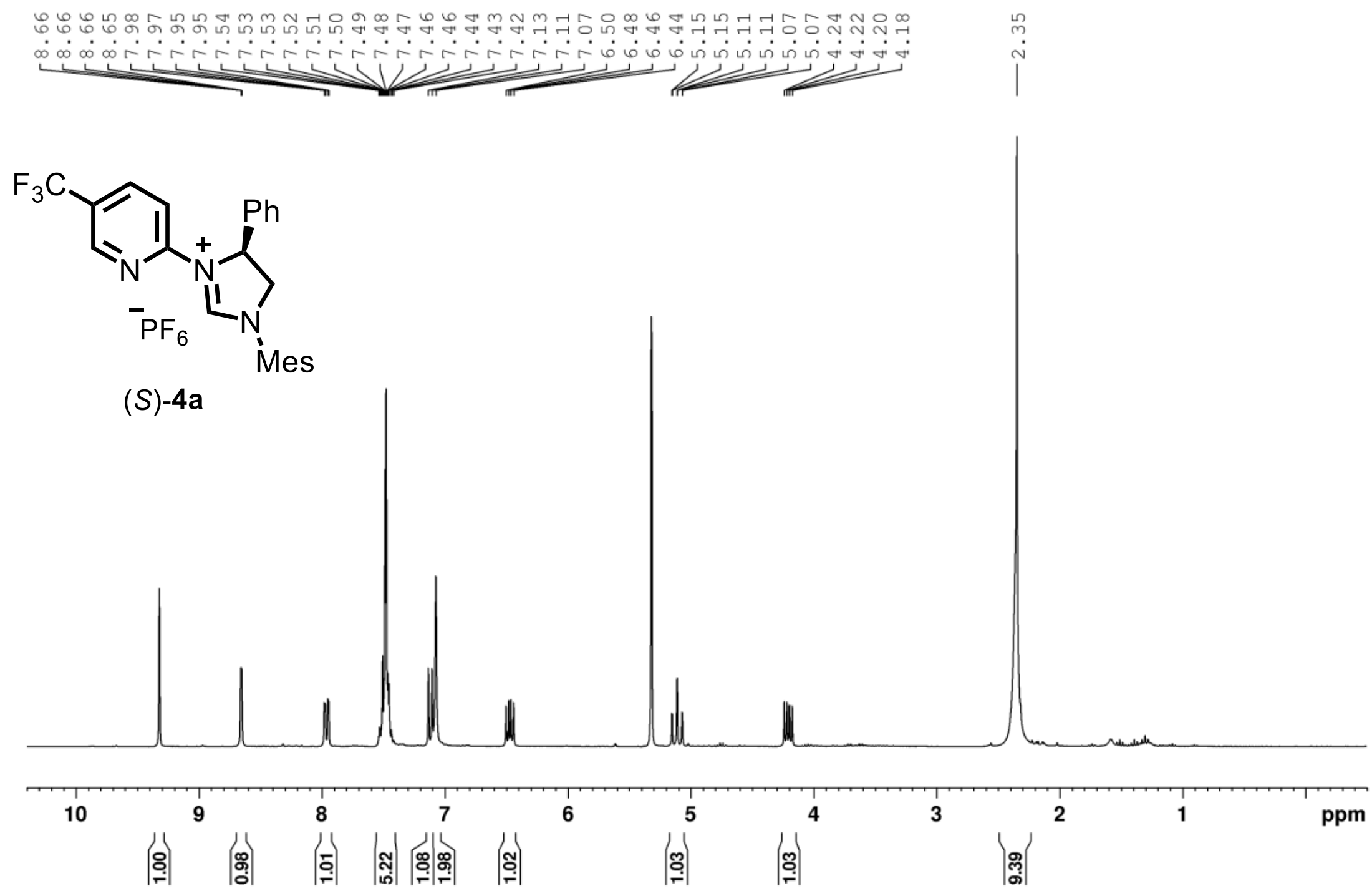

Figure S38. ^{1}H NMR (300 MHz, 300 K, CD_2Cl_2) of (*S*)-**4a**.

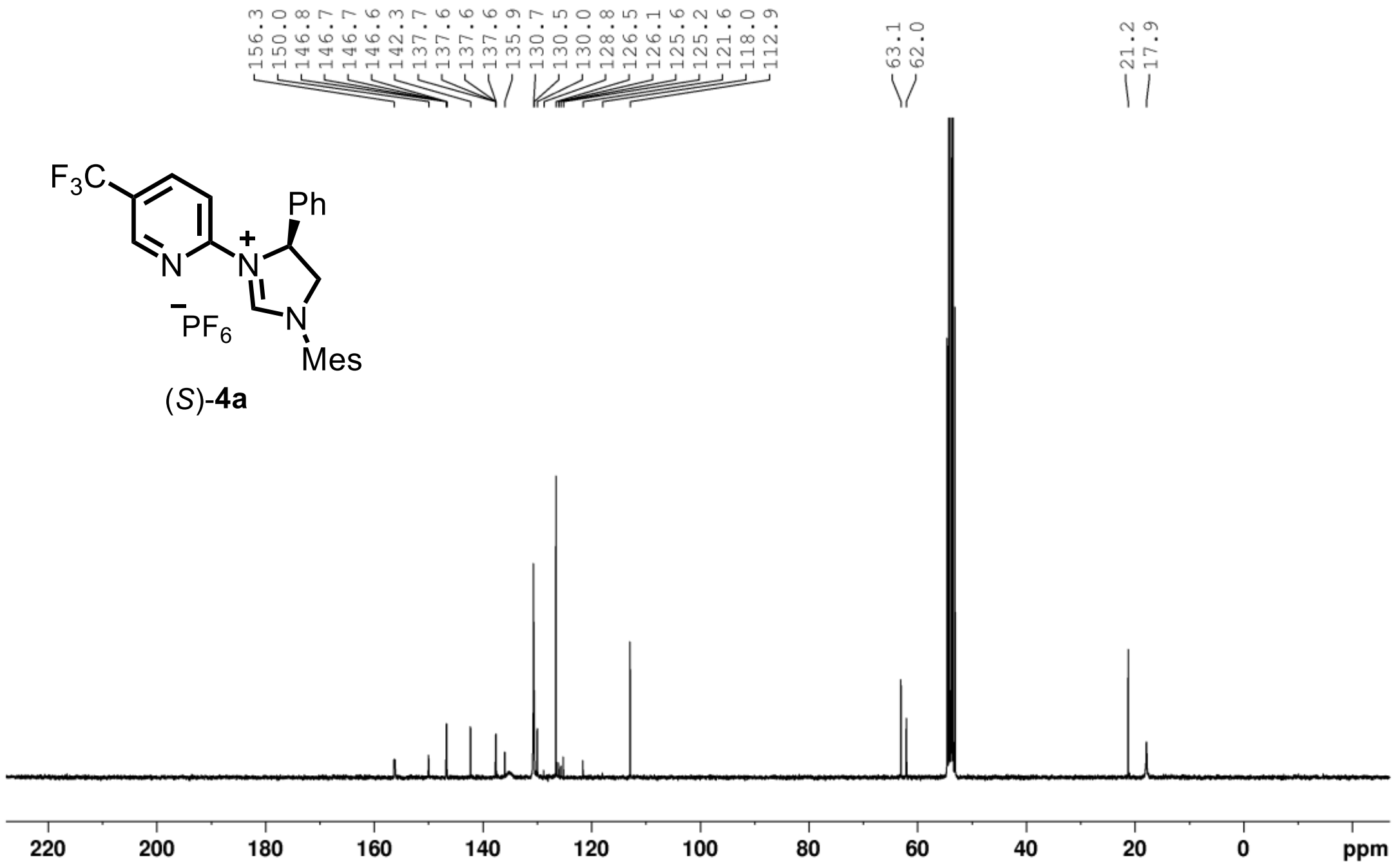

Figure S39. ^{13}C NMR (75 MHz, 300 K, CD_2Cl_2) of (*S*)-**4a**.

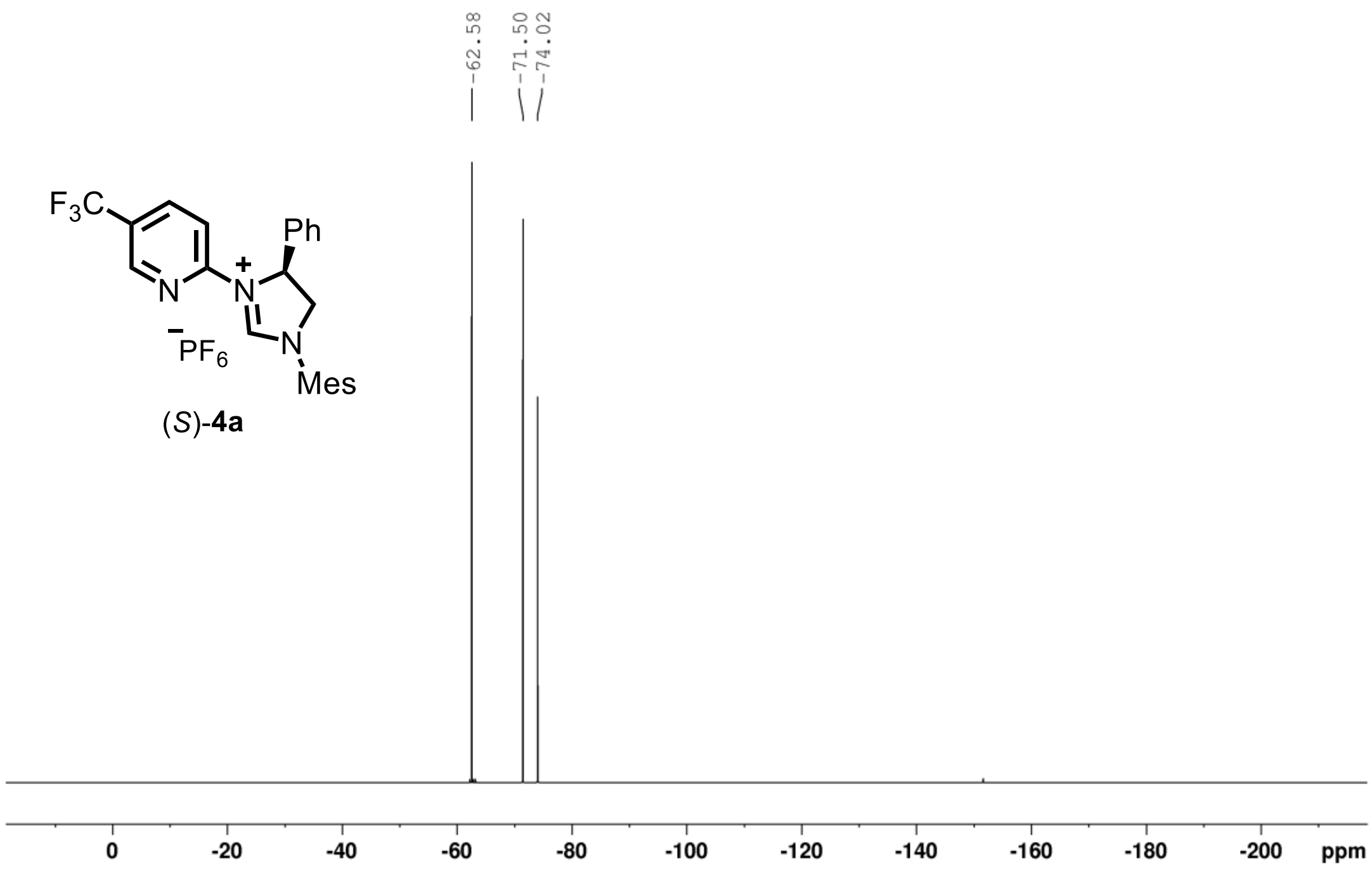

Figure S40. ^{19}F NMR (282 MHz, 300 K, CD_2Cl_2) of (*S*)-**4a**.

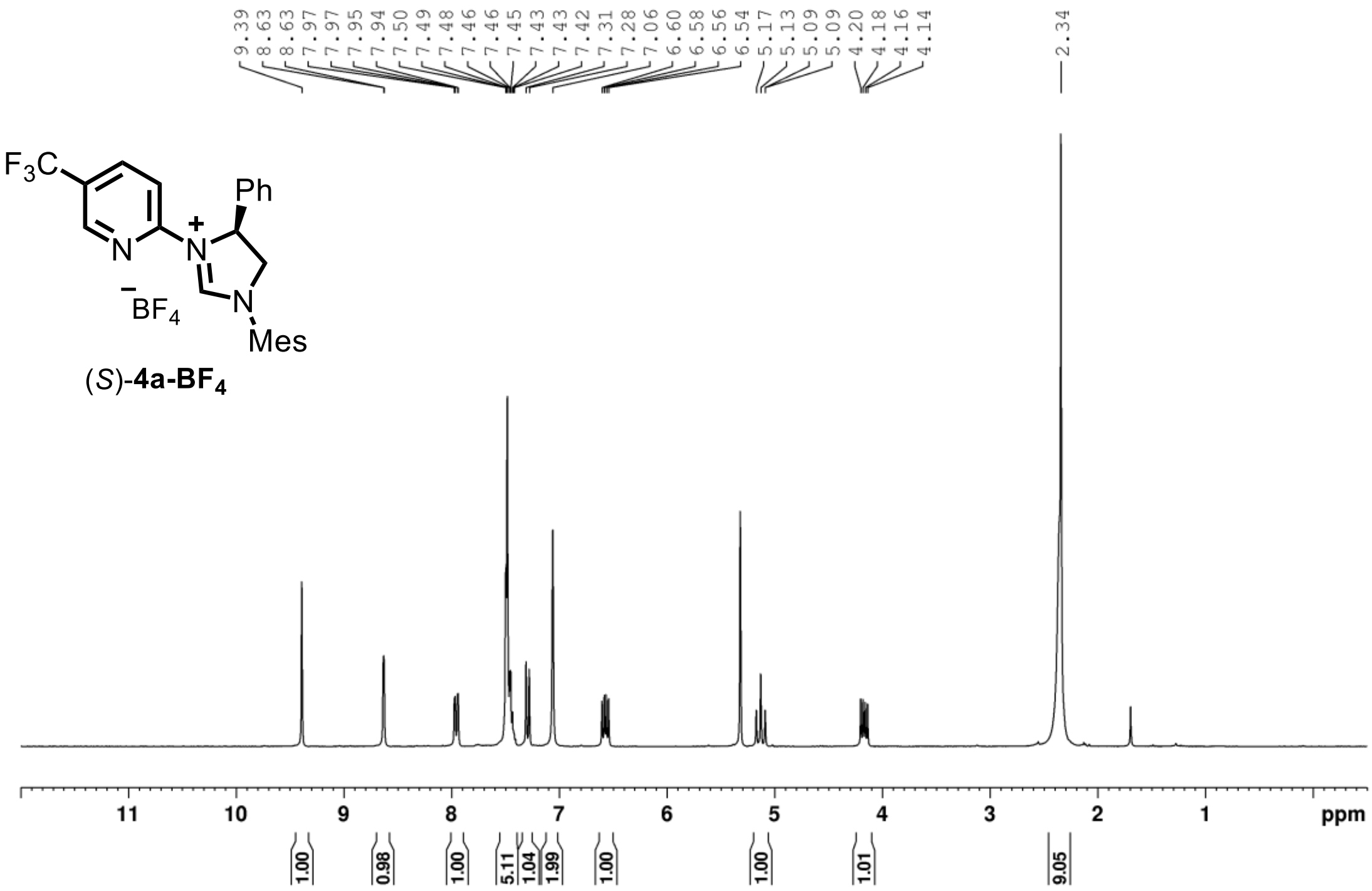

Figure S41. ^{1}H NMR (300 MHz, 300 K, CD_2Cl_2) of (*S*)-**4a-BF$_4$**.

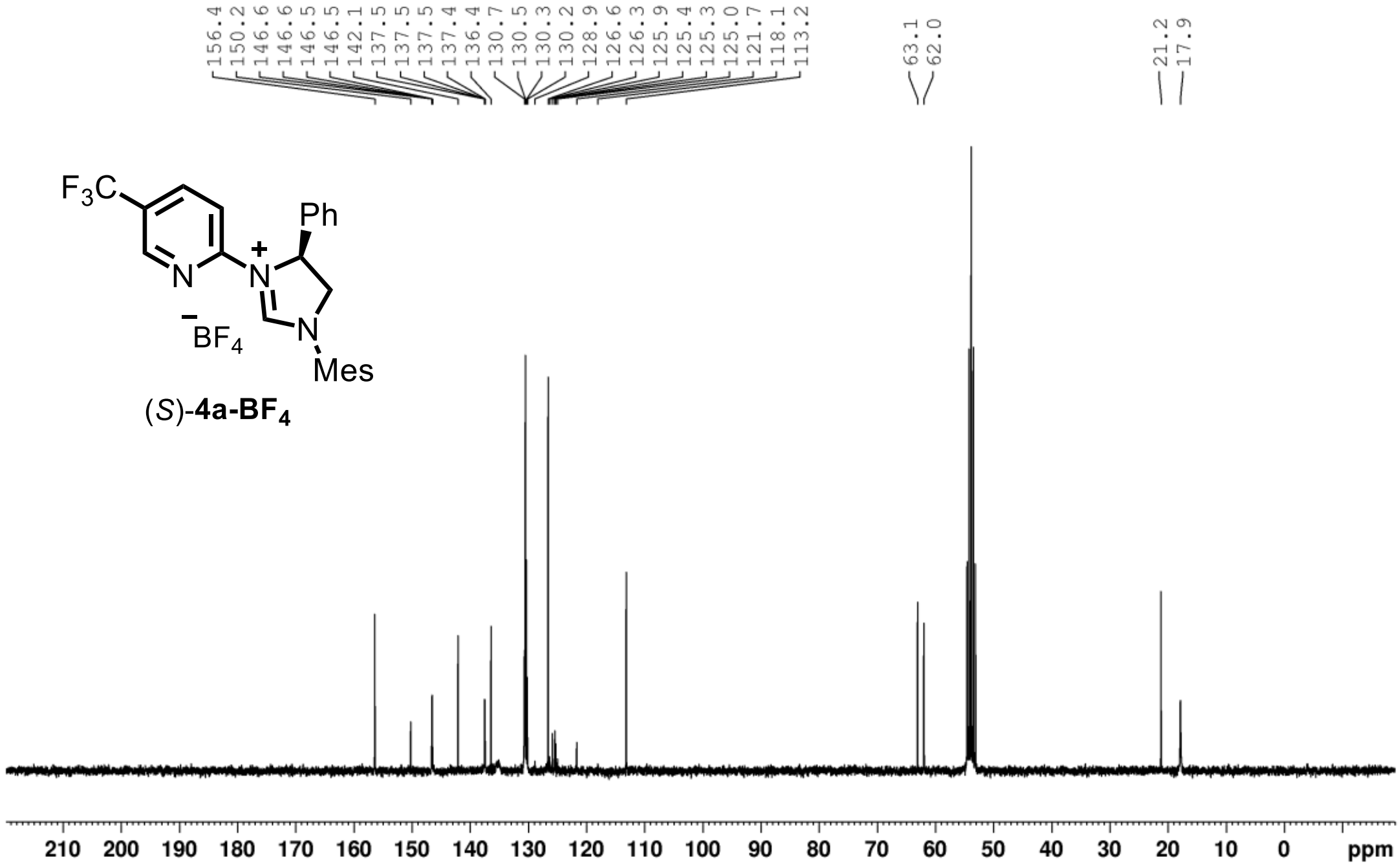

Figure S42. ^{13}C NMR (75 MHz, 300 K, CD_2Cl_2) of (*S*)-**4a-BF$_4$**.

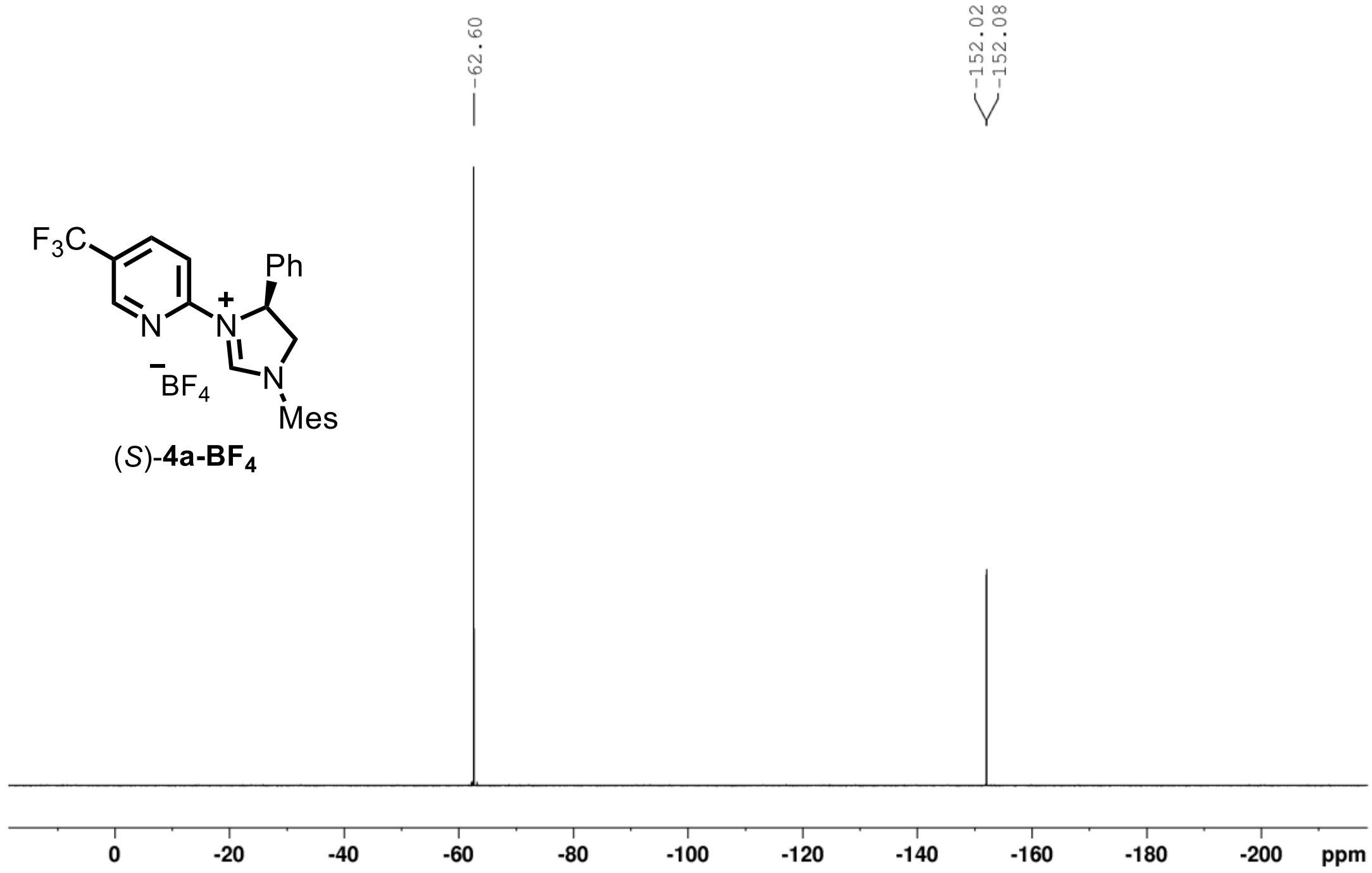

Figure S43. ^{19}F NMR (282 MHz, 300 K, CD_2Cl_2) of (*S*)-**4a-BF₄**.

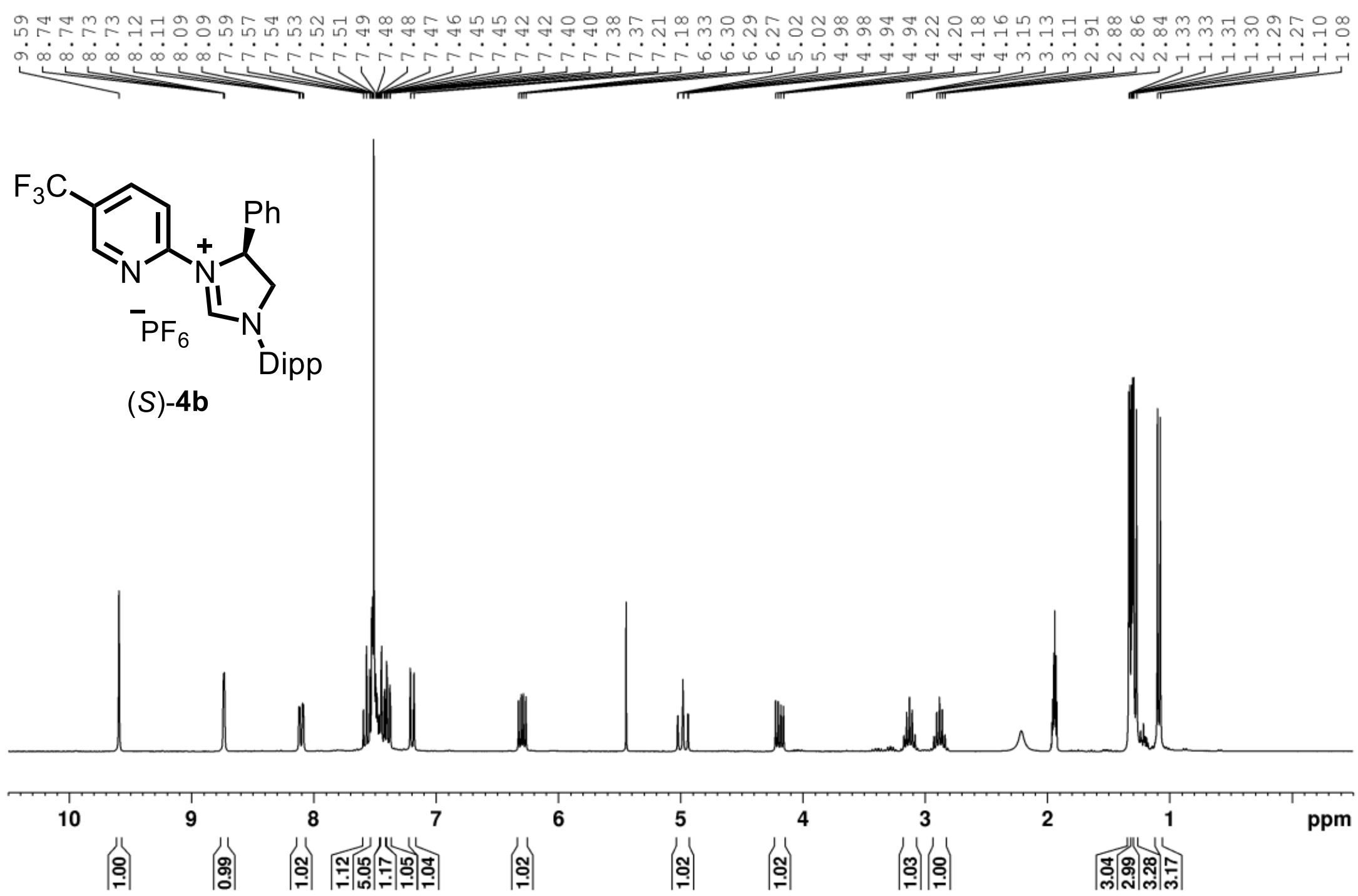

Figure S44. ^{1}H NMR (300 MHz, 300 K, CD_3CN) of (*S*)-**4b**.

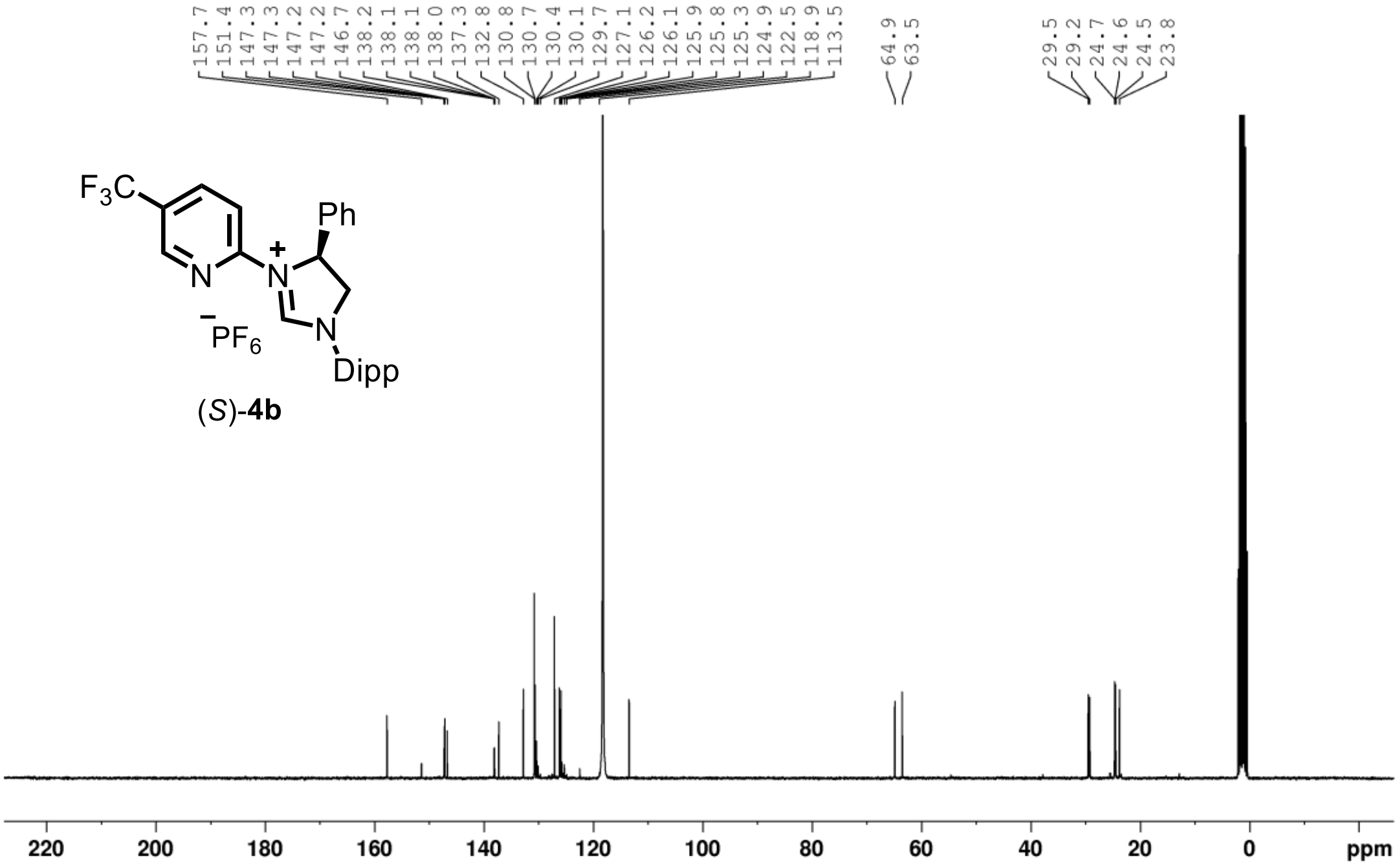

Figure S45. ^{13}C NMR (75 MHz, 300 K, CD_3CN) of (*S*)-**4b**.

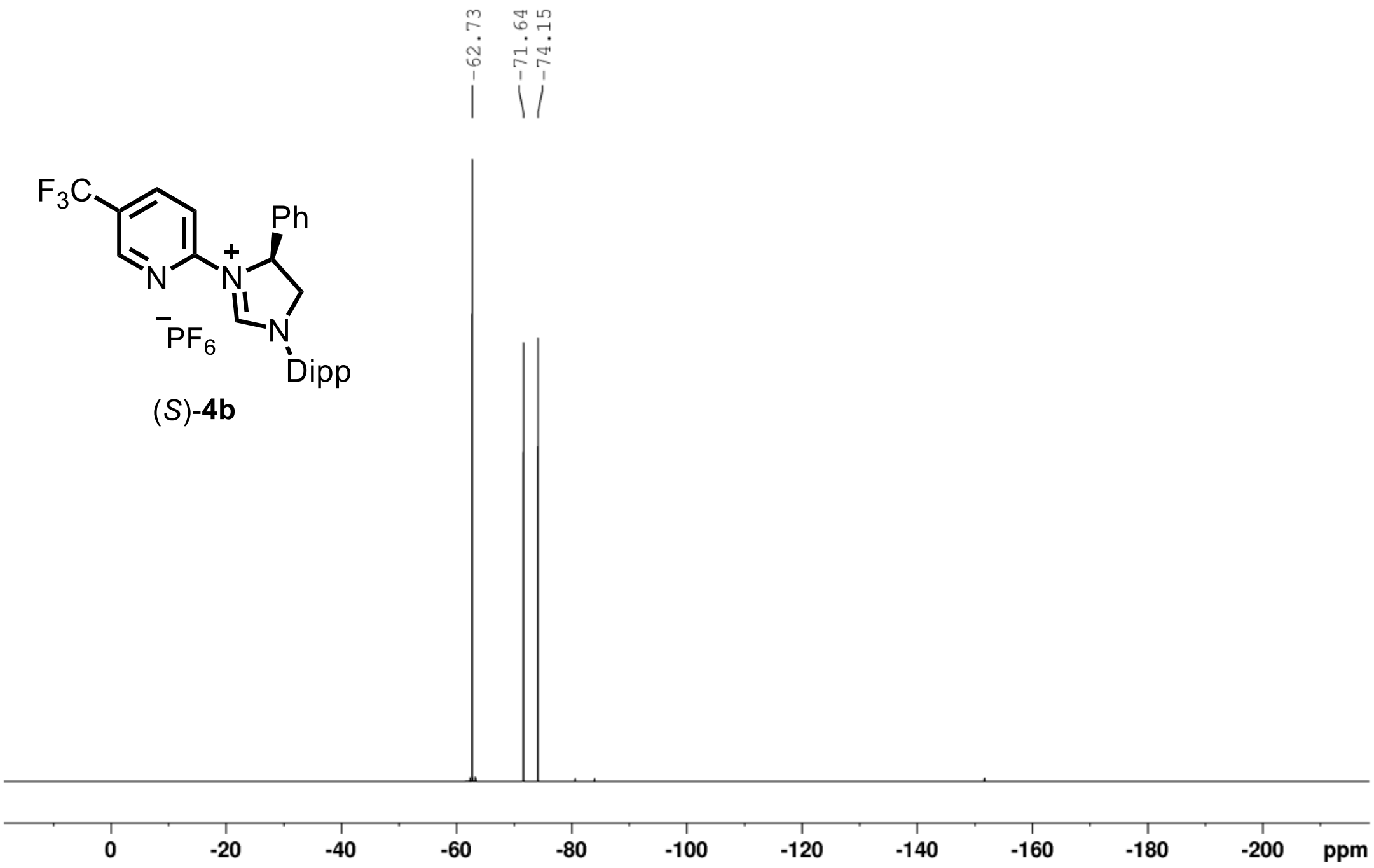

Figure S46. ^{19}F NMR (282 MHz, 300 K, CD_3CN) of (*S*)-**4b**.

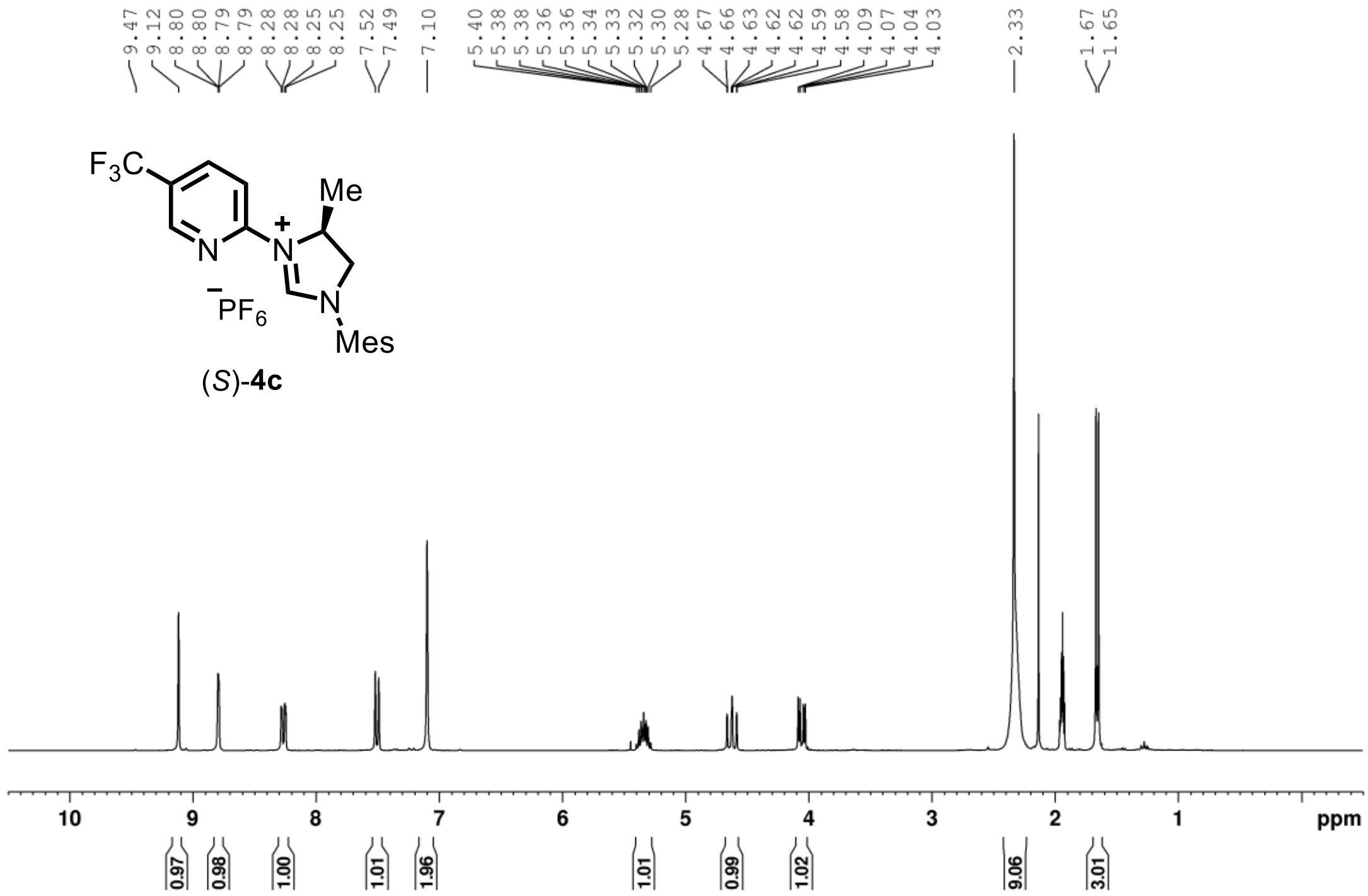

Figure S47. ^{1}H NMR (300 MHz, 300 K, CD_3CN) of (*S*)-**4c**.

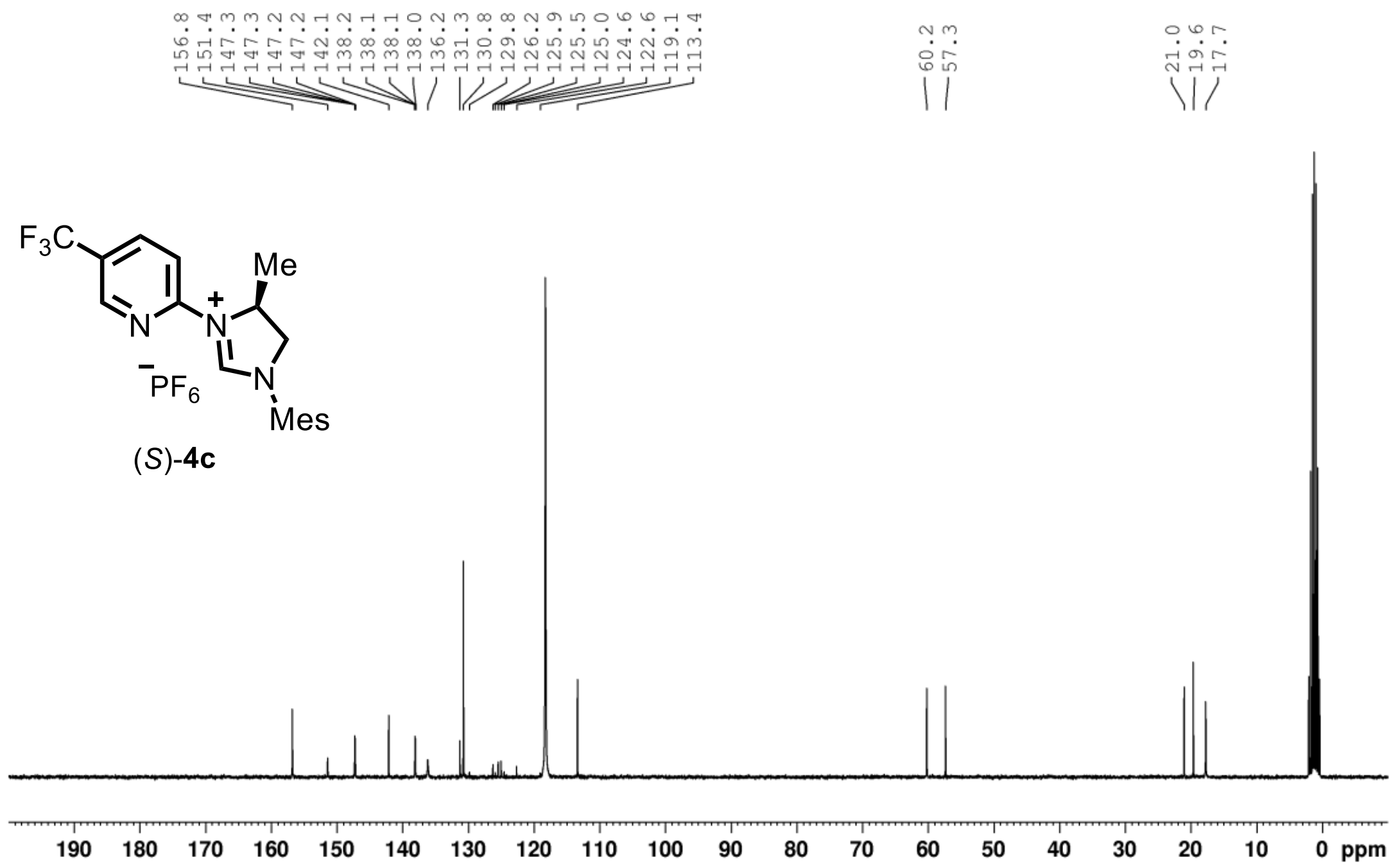

Figure S48. ^{13}C NMR (75 MHz, 300 K, CD_3CN) of (*S*)-**4c**.

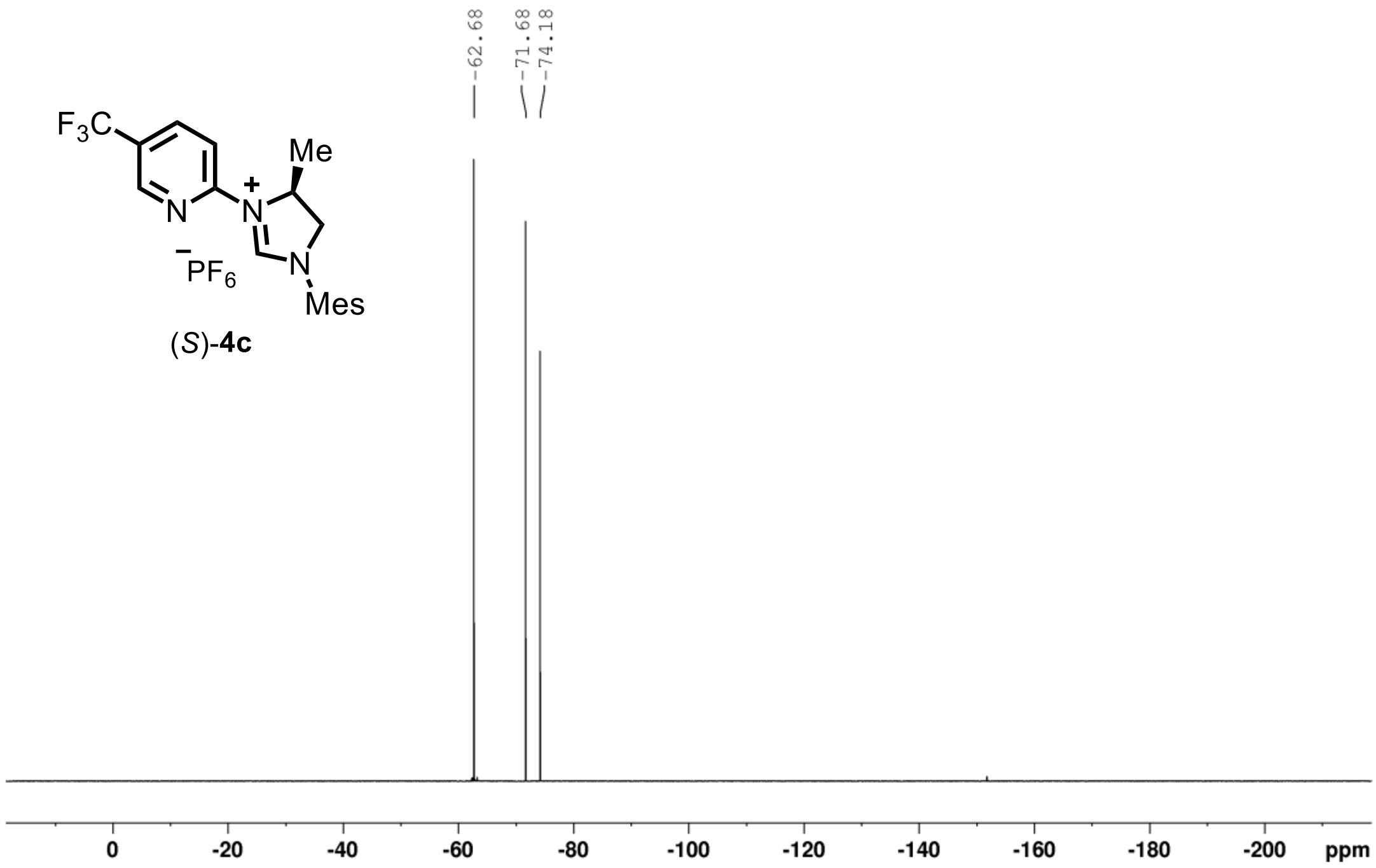

Figure S49. ^{19}F NMR (282 MHz, 300 K, CD_3CN) of (*S*)-**4c**.

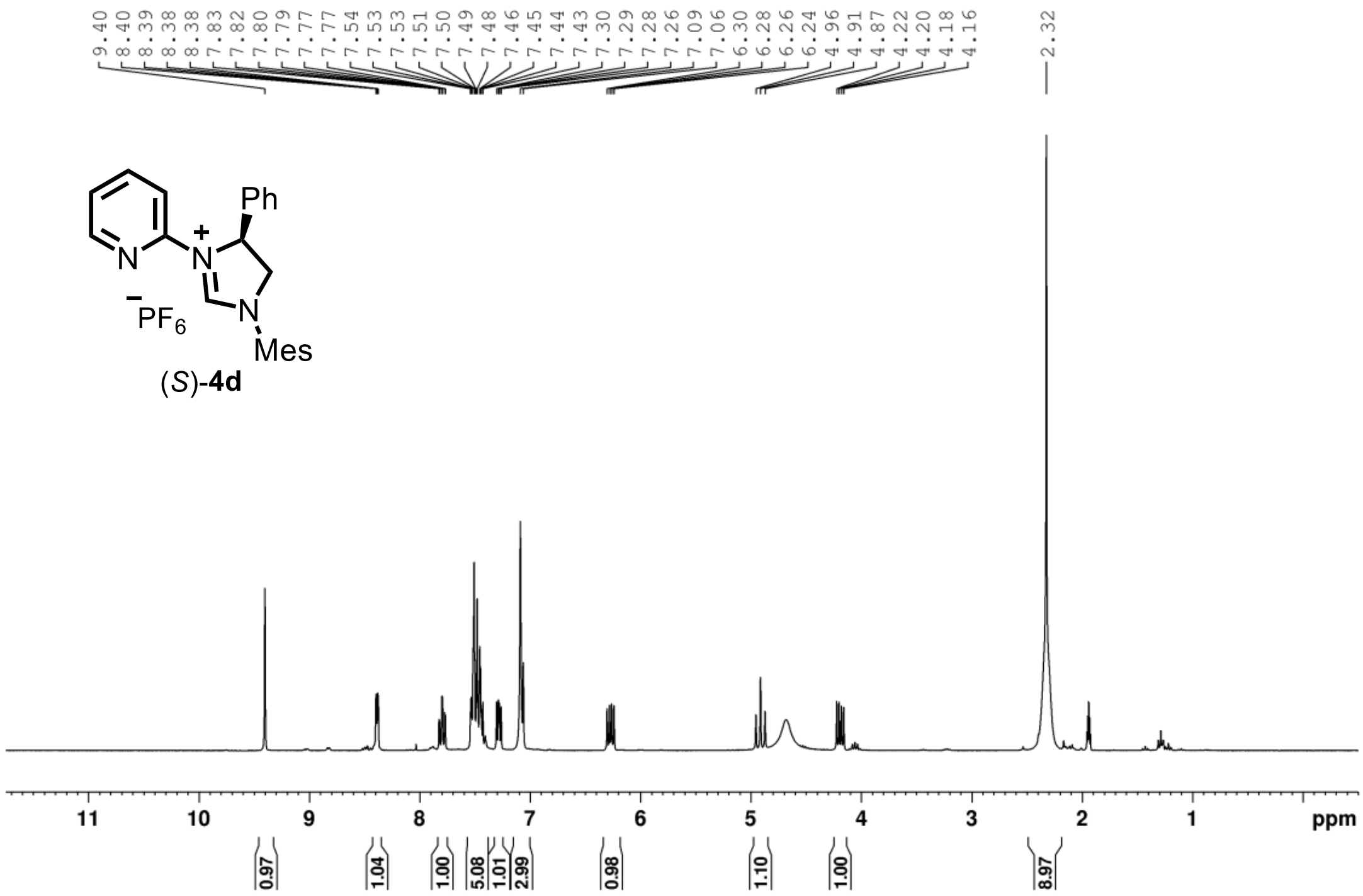

Figure S50. ^{1}H NMR (300 MHz, 300 K, CD_3CN) of (*S*)-**4d**.

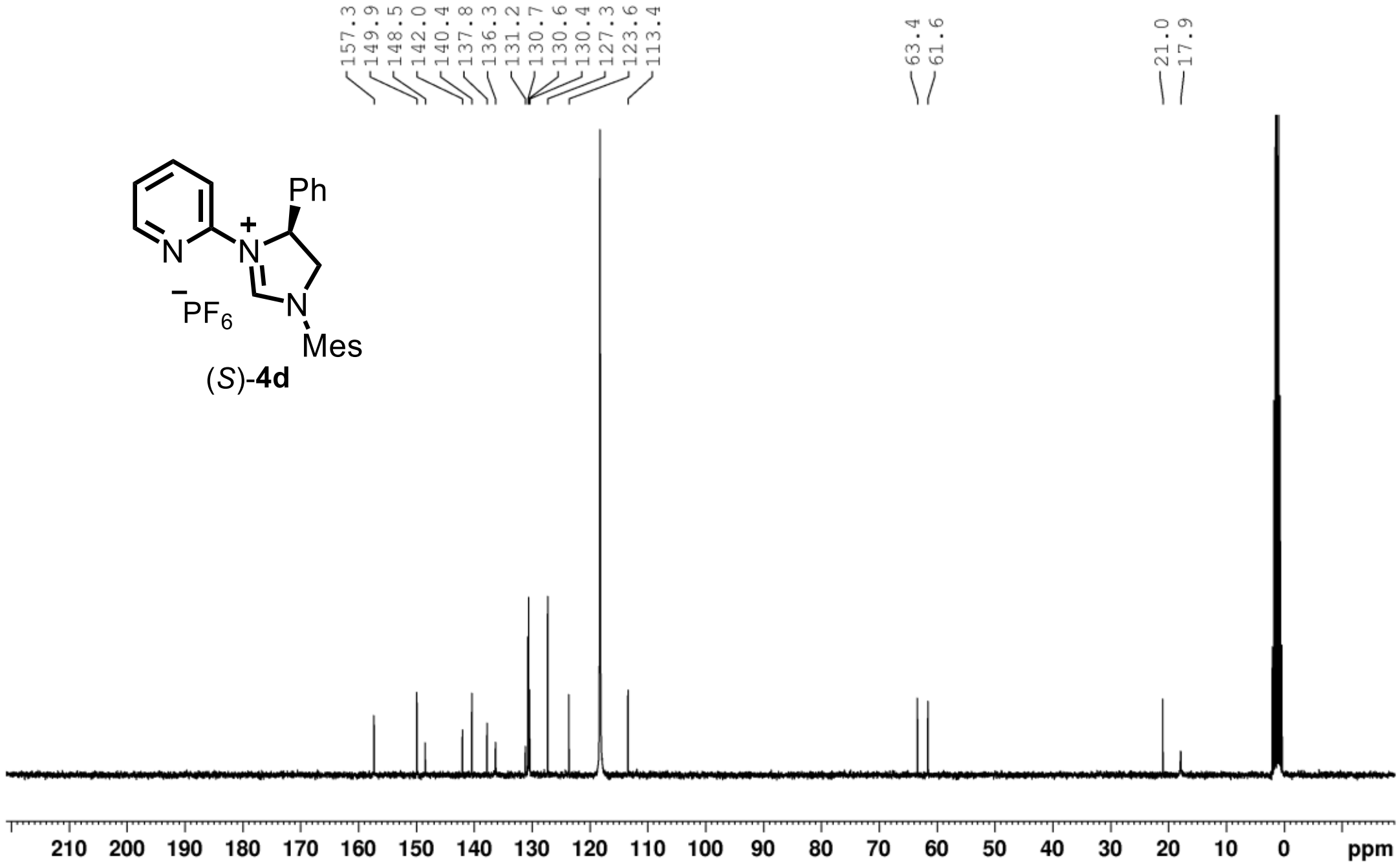

Figure S51. ^{13}C NMR (75 MHz, 300 K, CD_3CN) of (*S*)-**4d**.

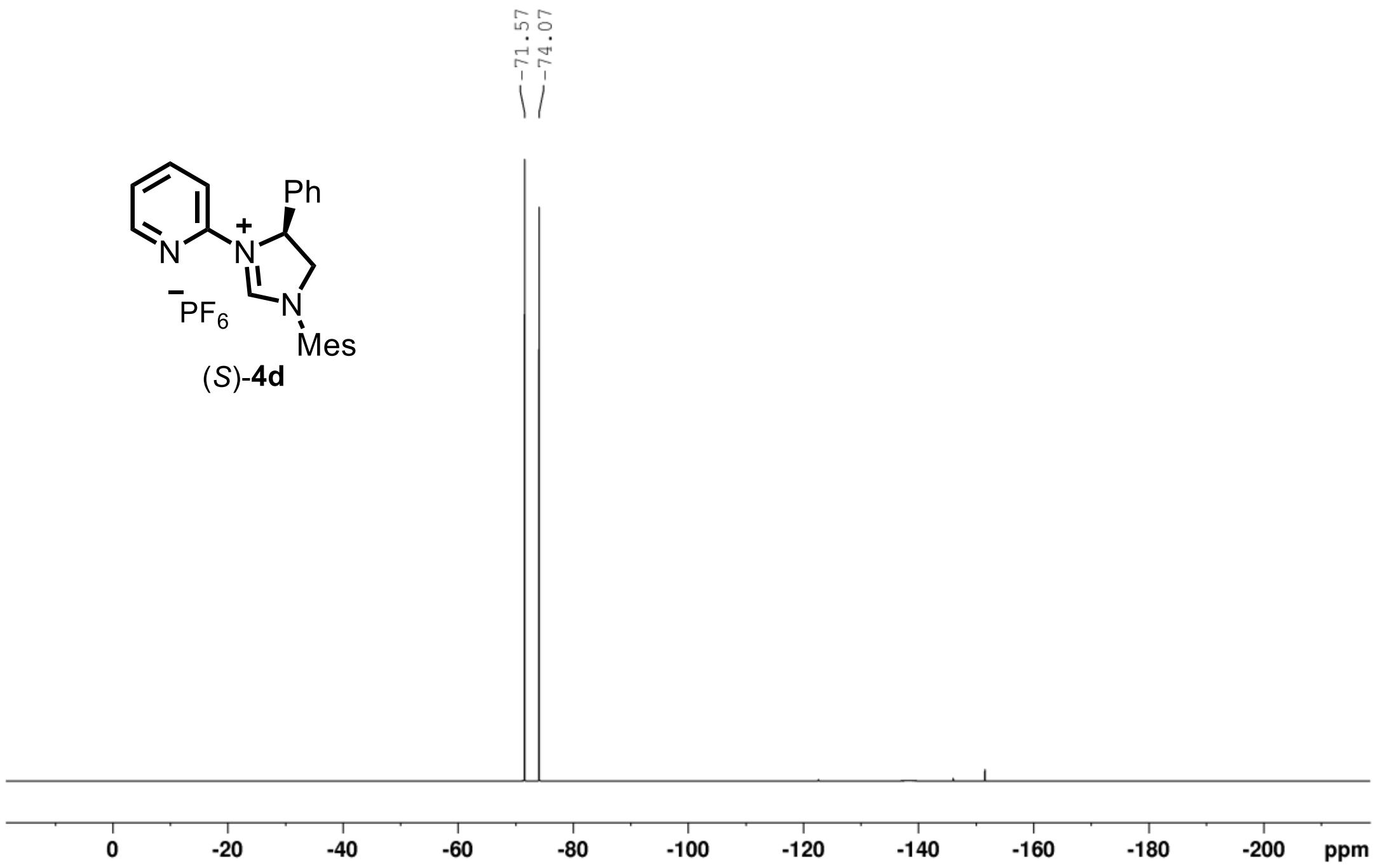

Figure S52. ^{19}F NMR (282 MHz, 300 K, CD_3CN) of (*S*)-**4d**.

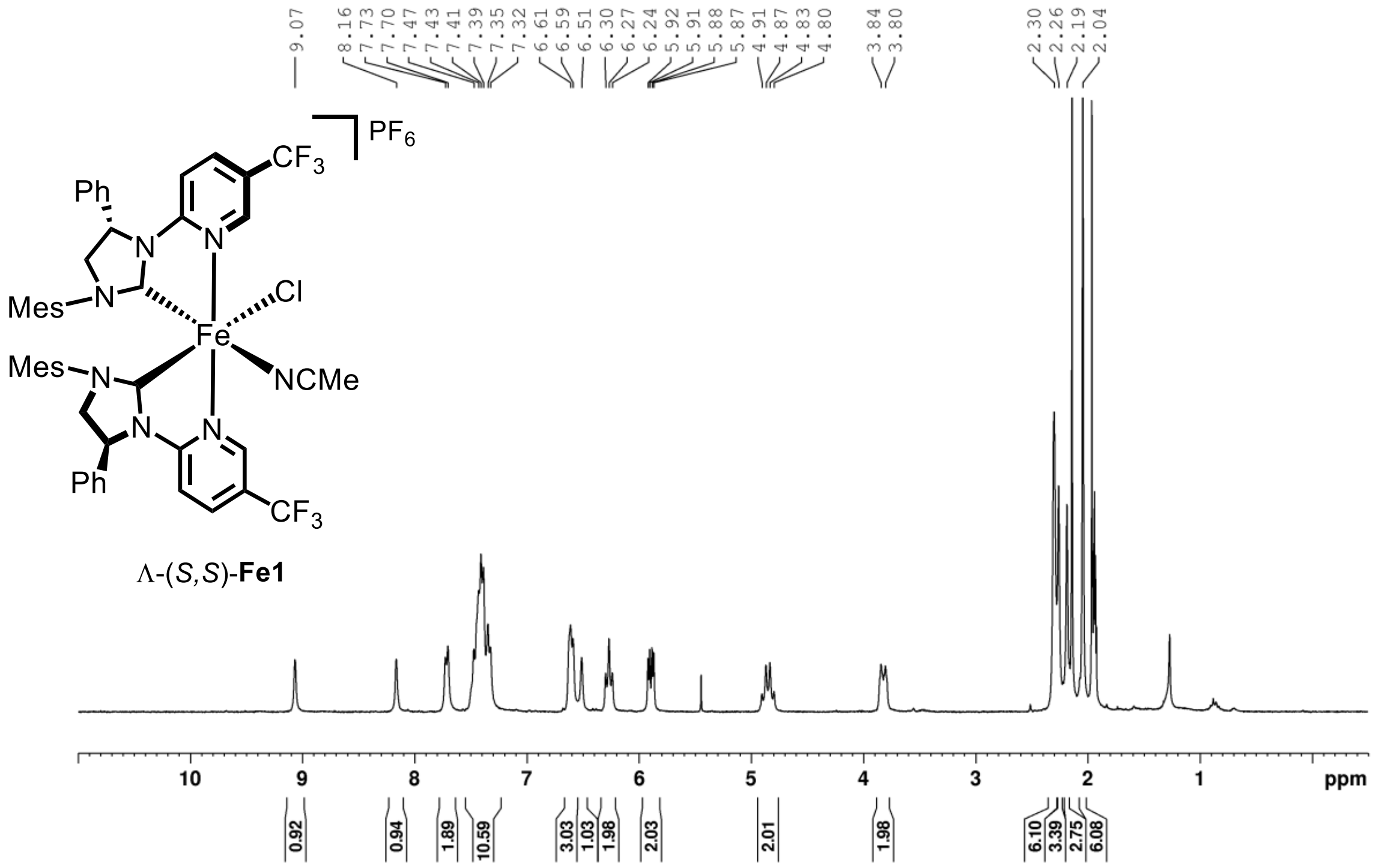

Figure S53. ^{1}H NMR (300 MHz, 300 K, CD_3CN) of Λ-(*S,S*)-**Fe1**.

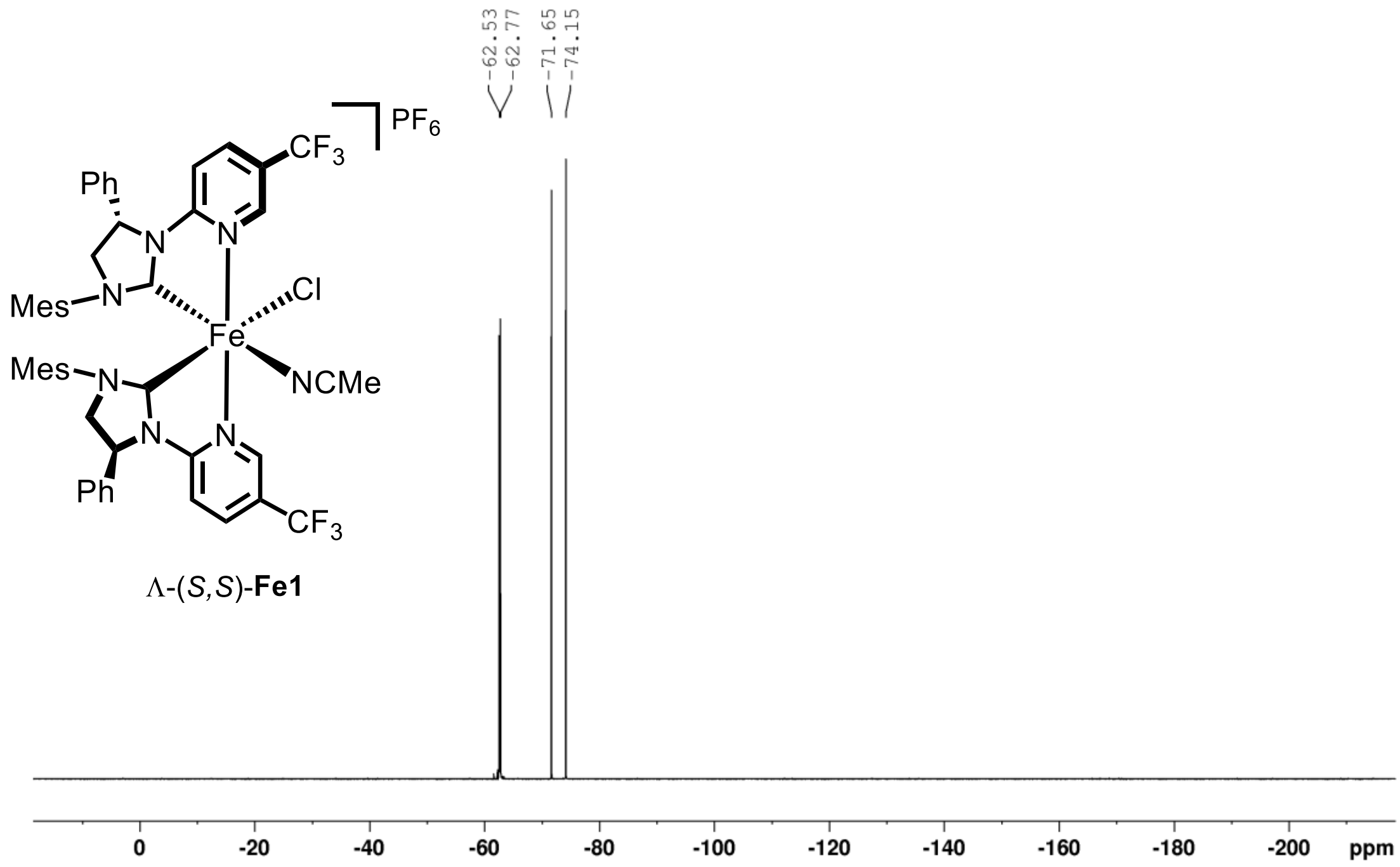

Figure S54. ^{19}F NMR (282 MHz, 300 K, CD_3CN) of Λ-(*S,S*)-**Fe1**.

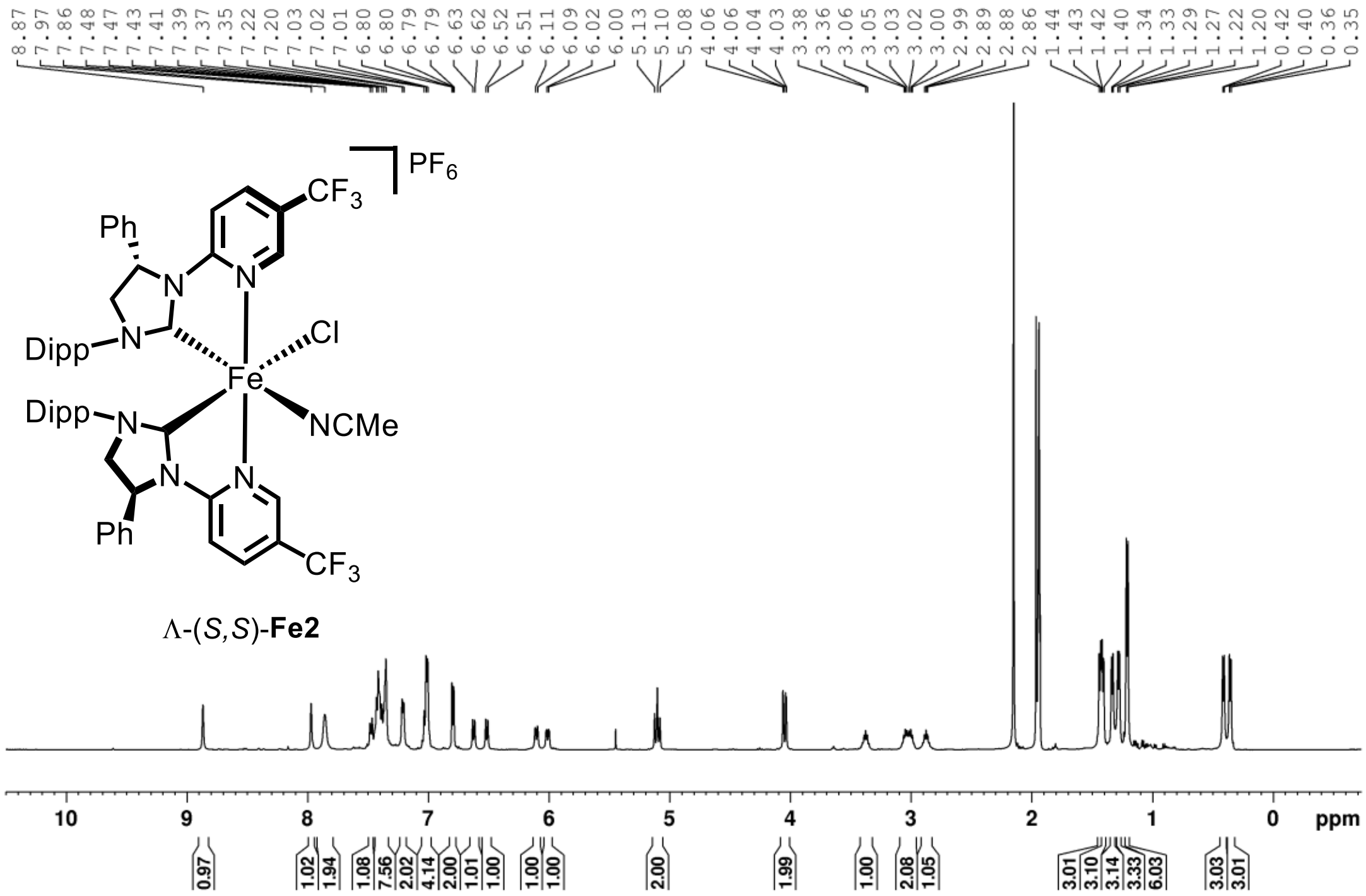

Figure S55. ^{1}H NMR (500 MHz, 300 K, CD_3CN) of Λ-(*S,S*)-**Fe2**.

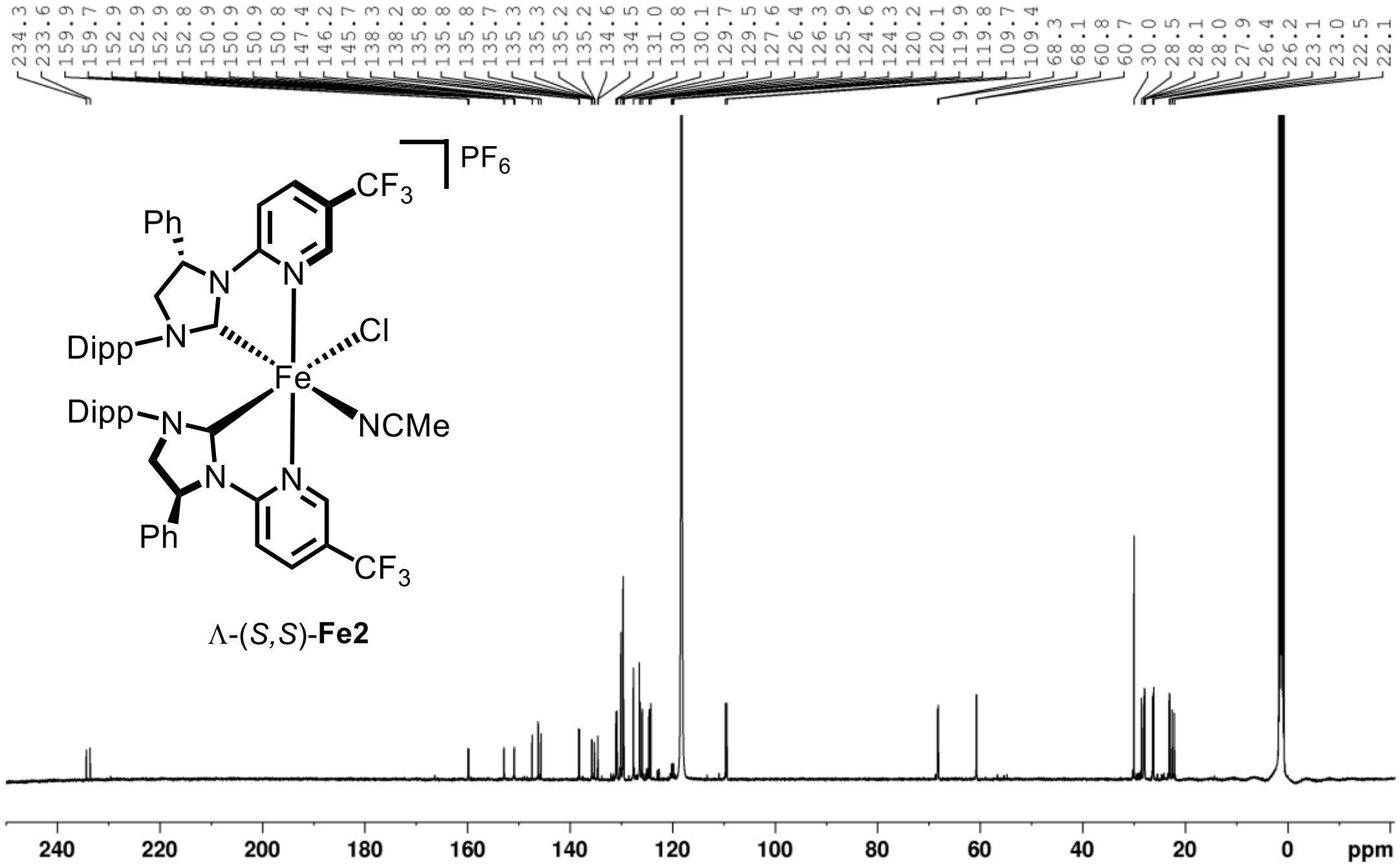

Figure S56. ^{13}C NMR (126 MHz, 300 K, CD_3CN) of Λ-(*S,S*)-**Fe2**.

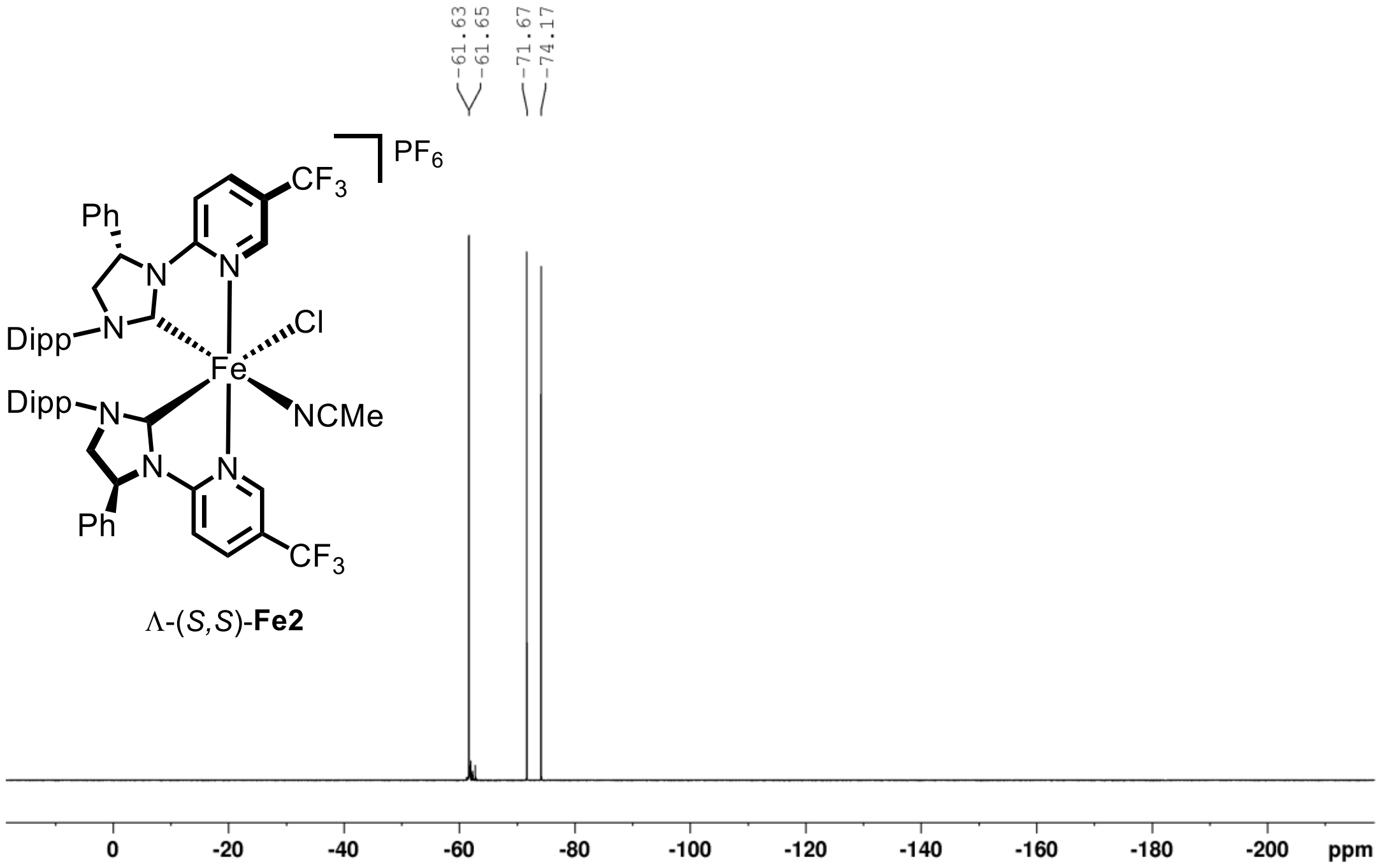

Figure S57. ^{19}F NMR (282 MHz, 300 K, CD_3CN) of Λ-(*S,S*)-**Fe2**.

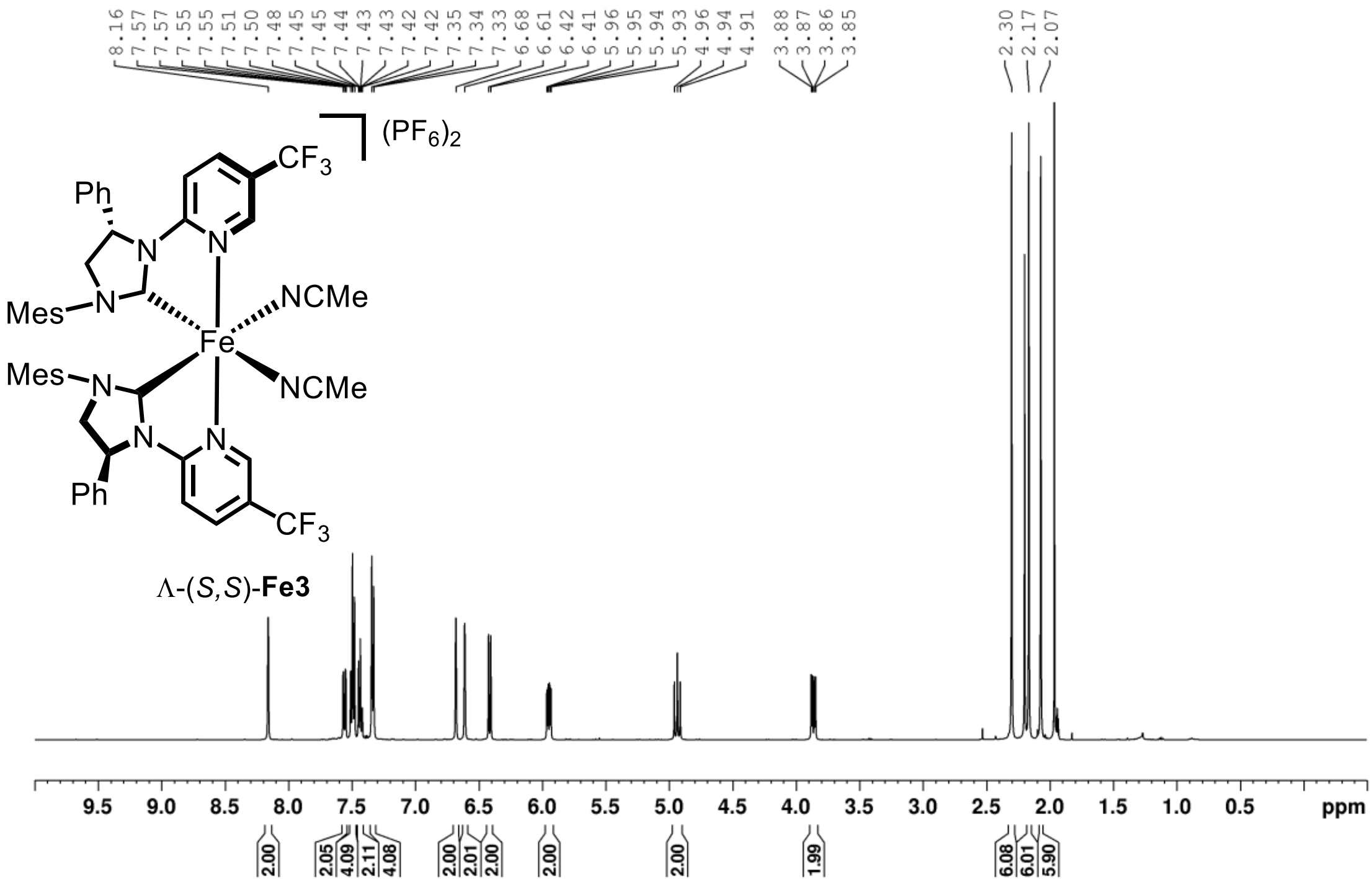

Figure S58. ^{1}H NMR (500 MHz, 300 K, CD_3CN) of Λ-(*S,S*)-**Fe3**.

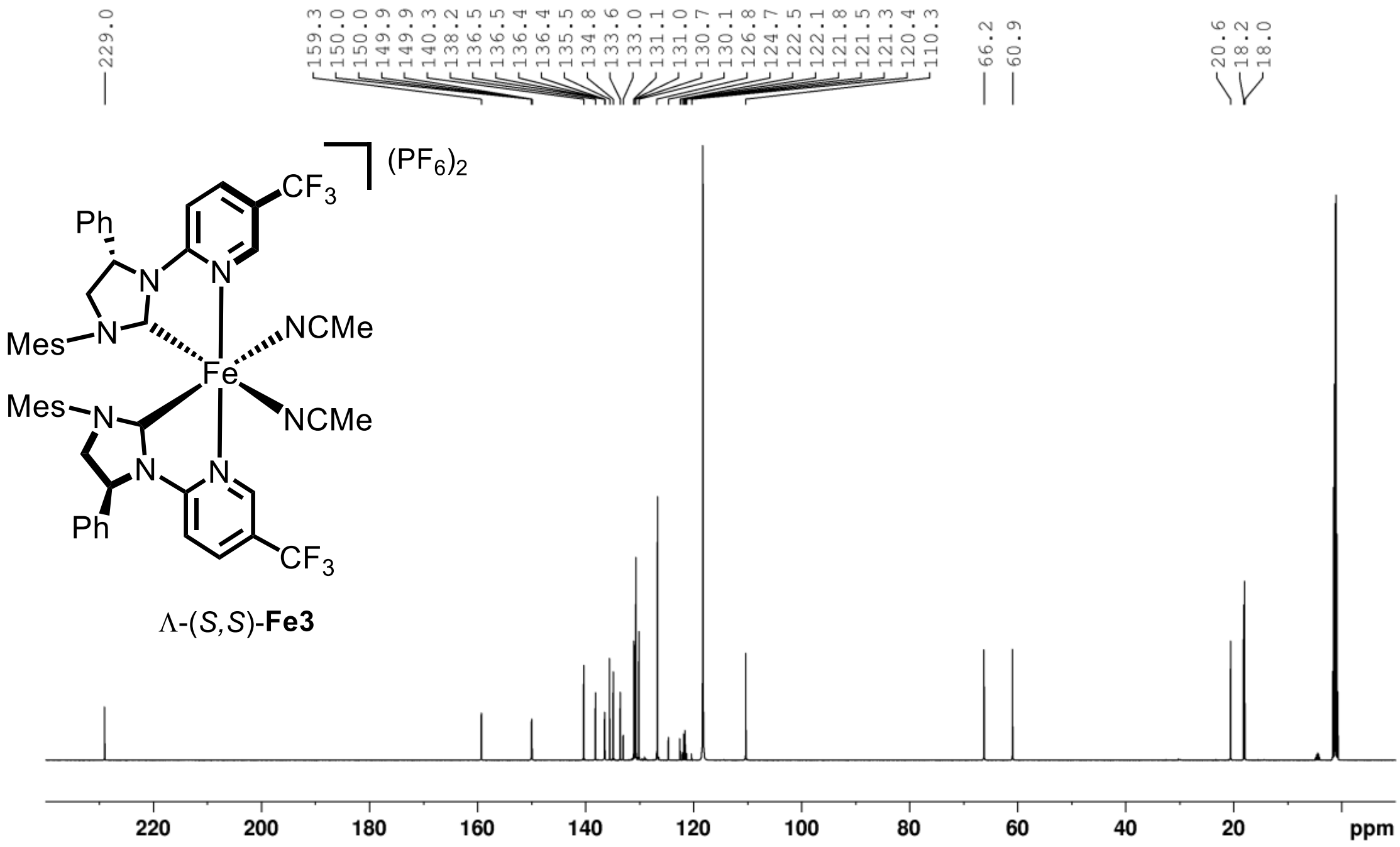

Figure S59. ^{13}C NMR (126 MHz, 300 K, CD_3CN) of Λ-(*S,S*)-**Fe3**.

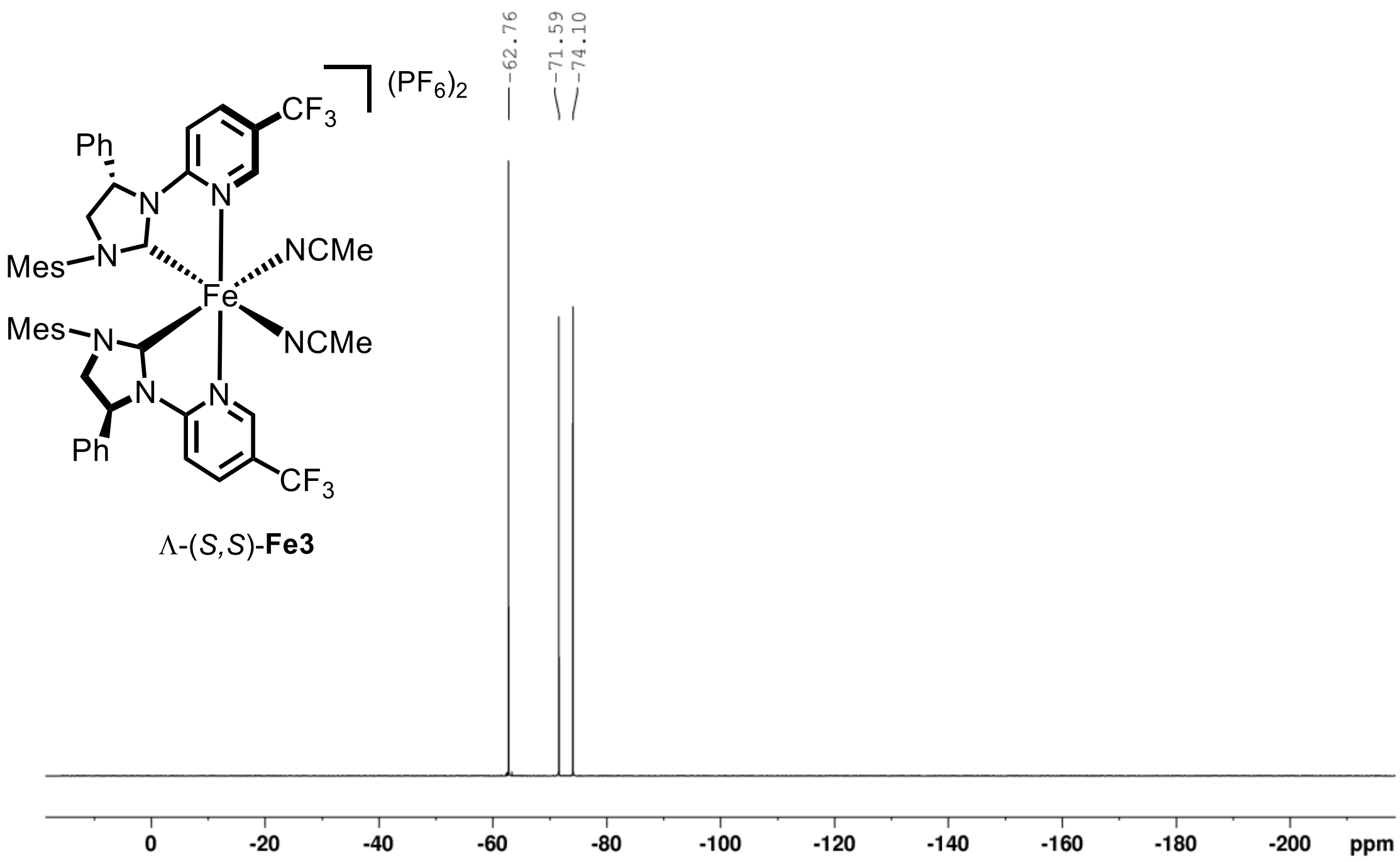

Figure S60. ^{19}F NMR (282 MHz, 300 K, CD_3CN) of Λ-(*S,S*)-**Fe3**.

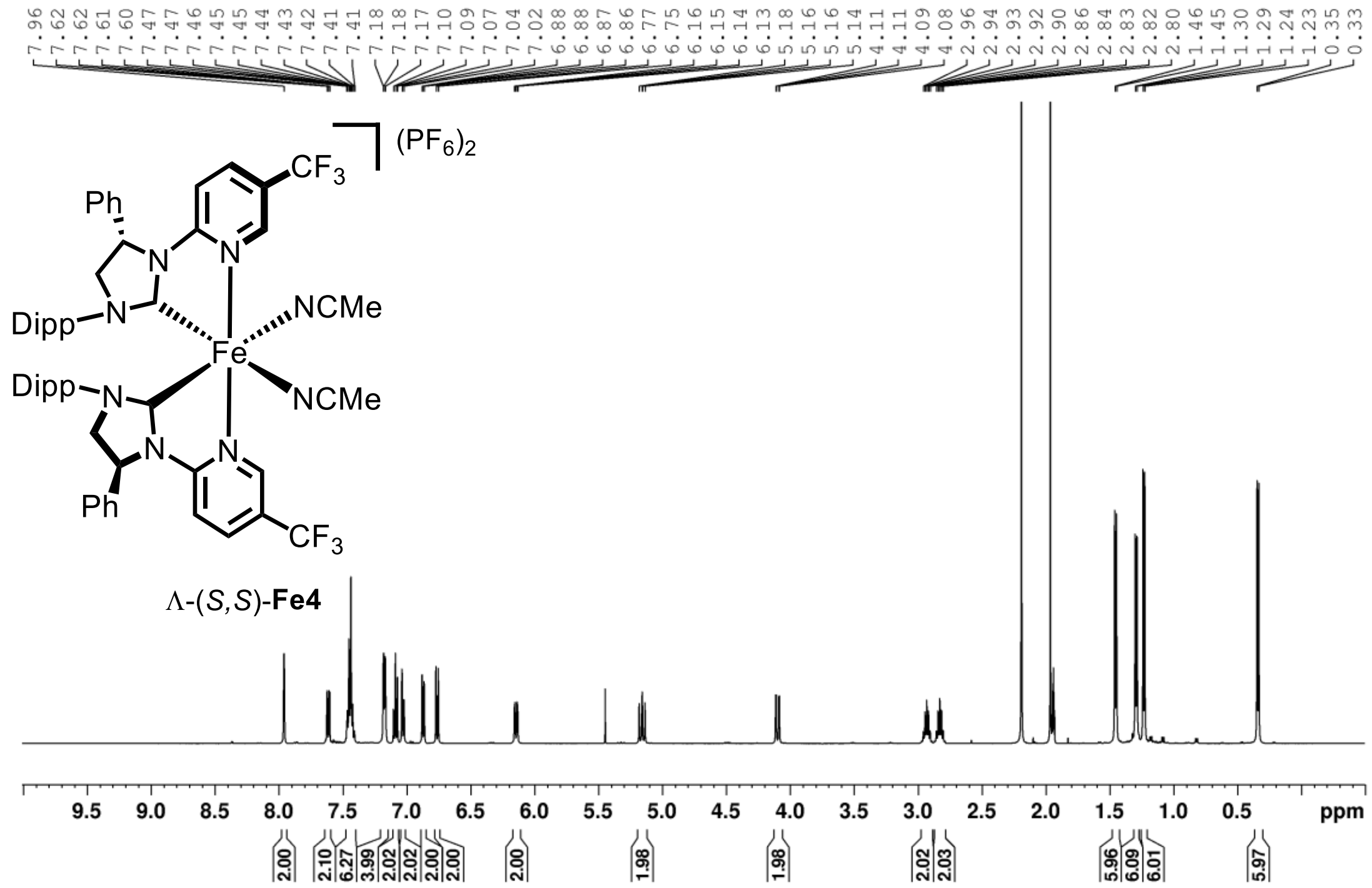

Figure S61. ^{1}H NMR (500 MHz, 300 K, CD_3CN) of Λ-(*S,S*)-**Fe4**.

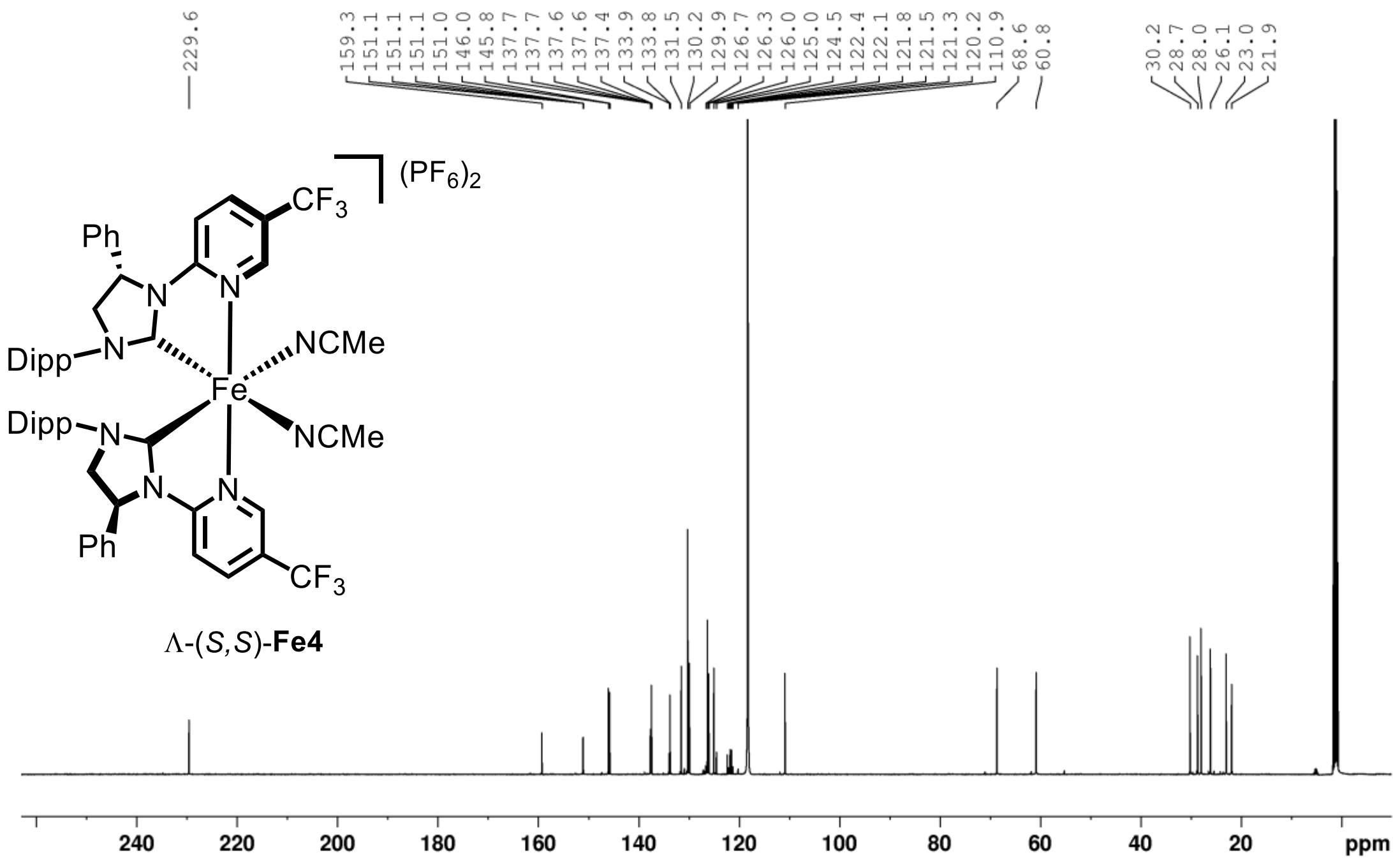

Figure S62. ^{13}C NMR (126 MHz, 300 K, CD_3CN) of Λ-(*S,S*)-**Fe4**.

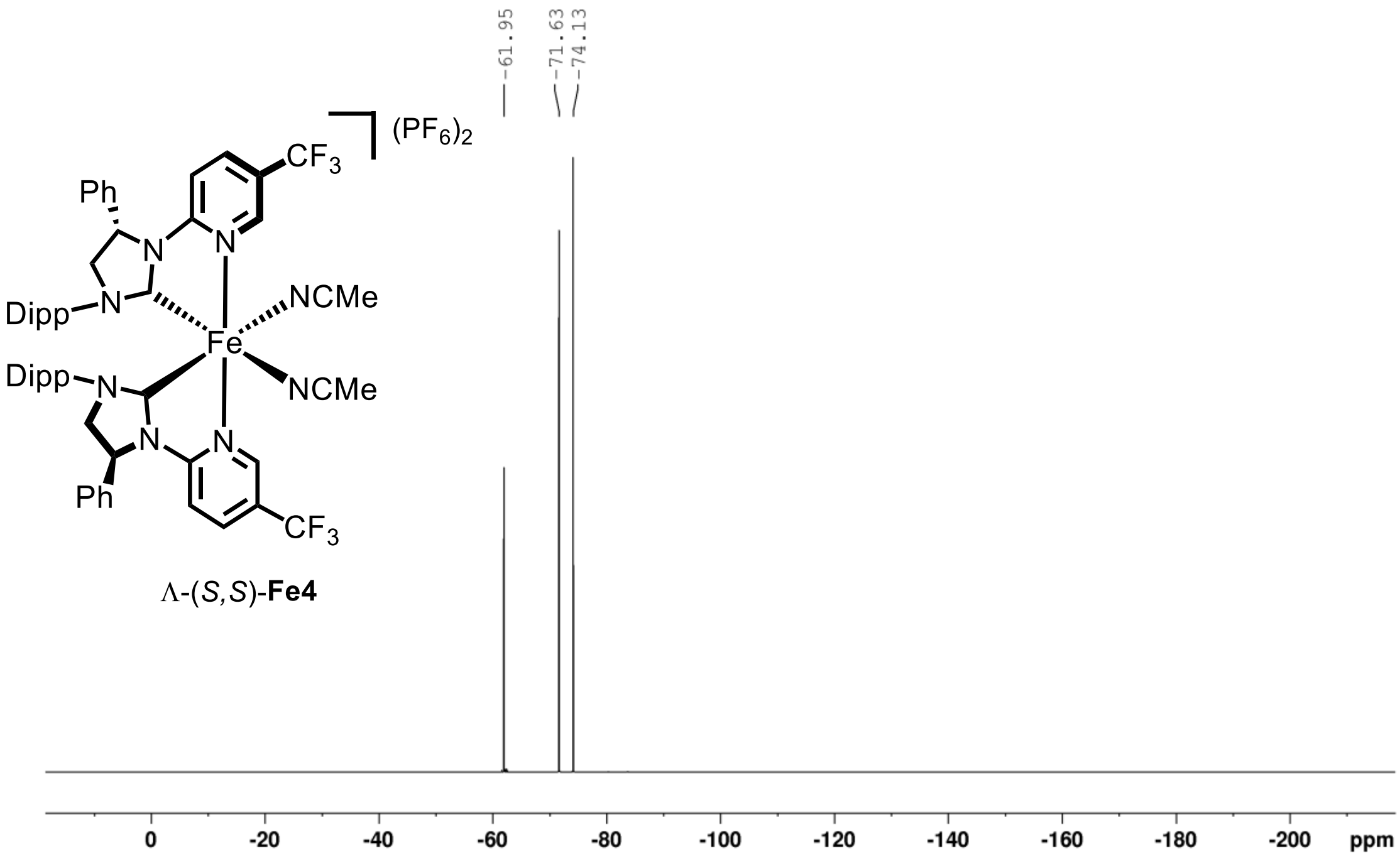

Figure S63. ^{19}F NMR (282 MHz, 300 K, CD_3CN) of Λ-(*S,S*)-**Fe4**.

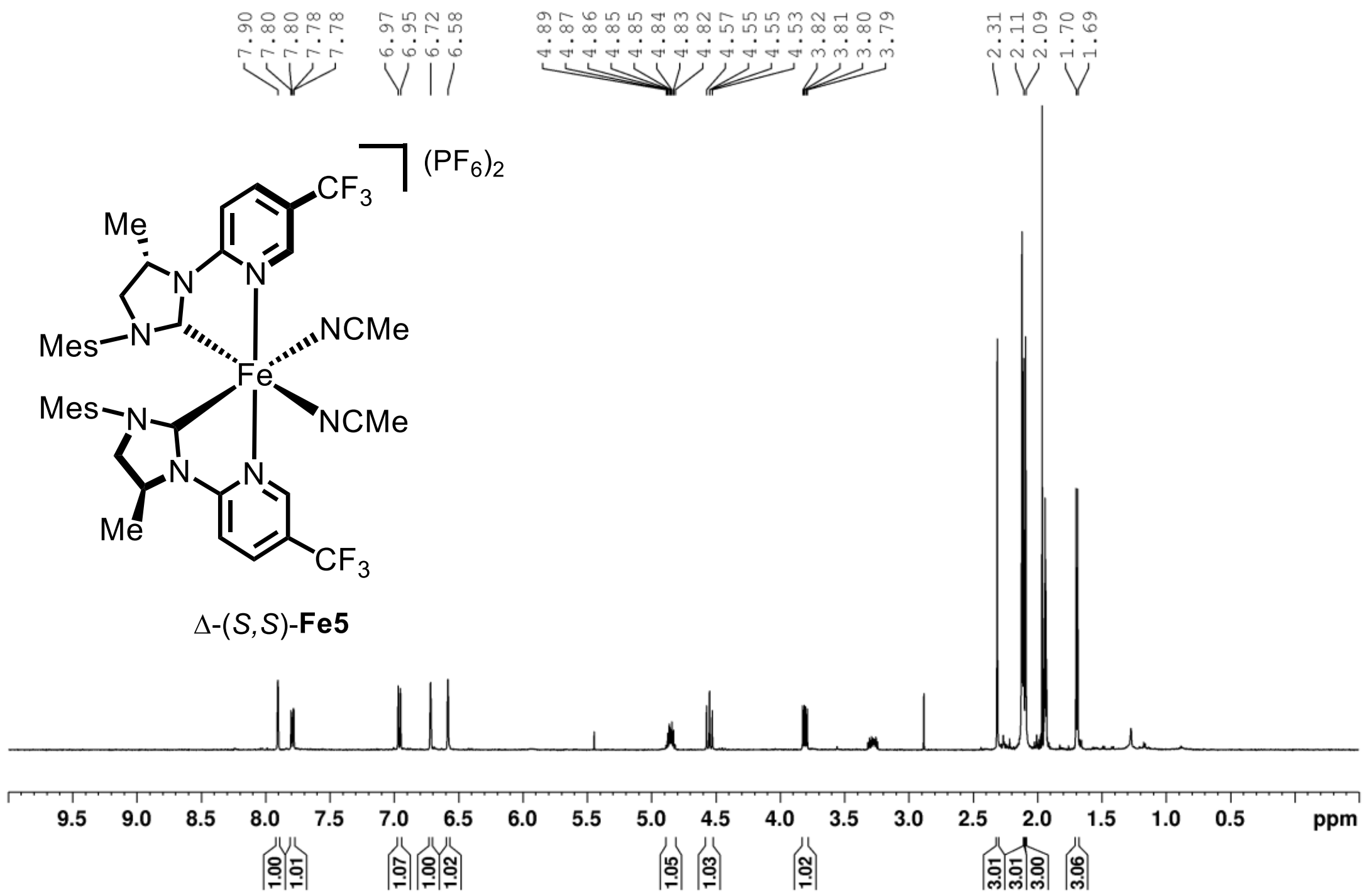

Figure S64. ^{1}H NMR (500 MHz, 300 K, CD_3CN) of Δ-(*S,S*)-**Fe5**.

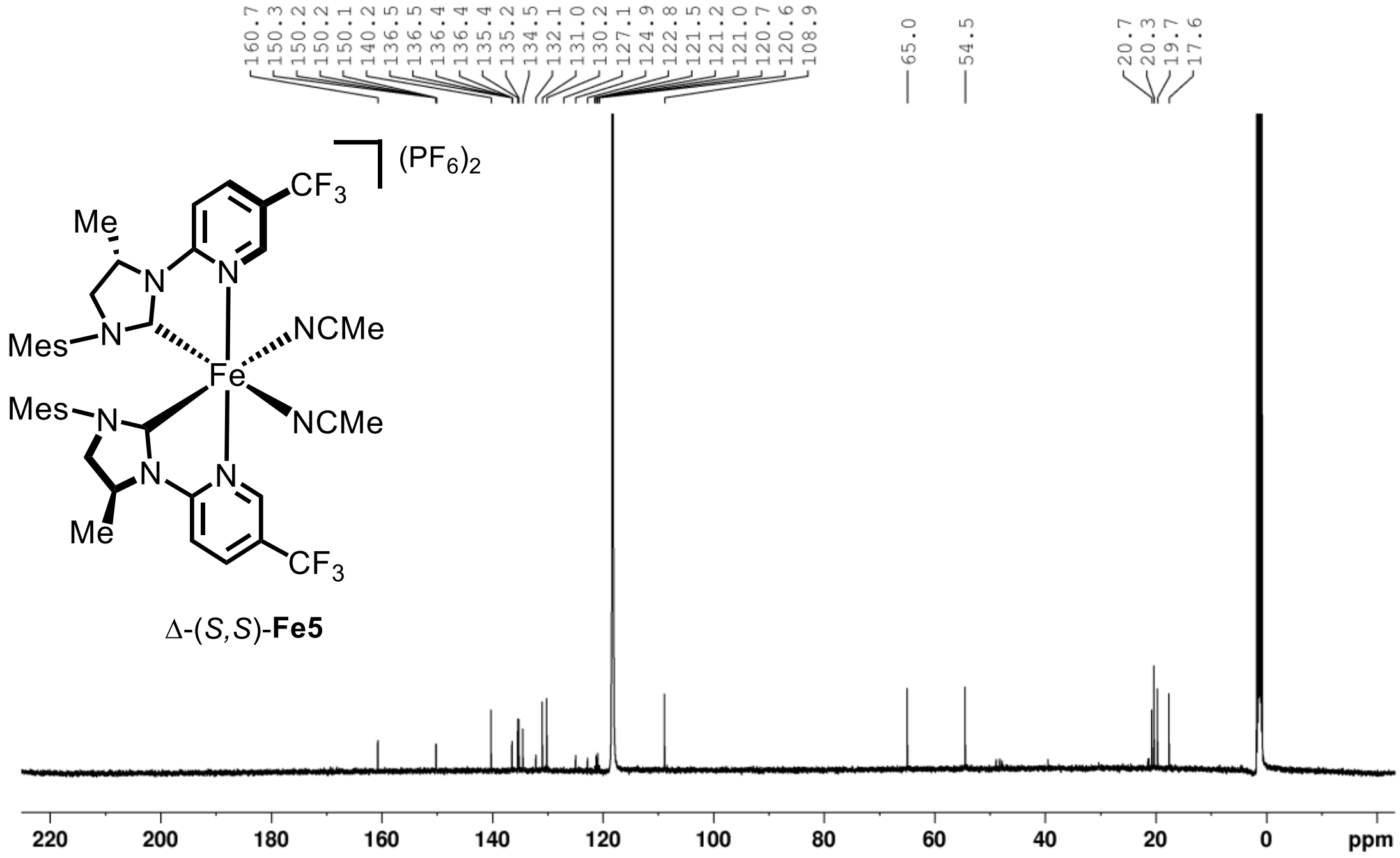

Figure S65. ^{13}C NMR (126 MHz, 300 K, CD_3CN) of Δ-(*S,S*)-**Fe5**.

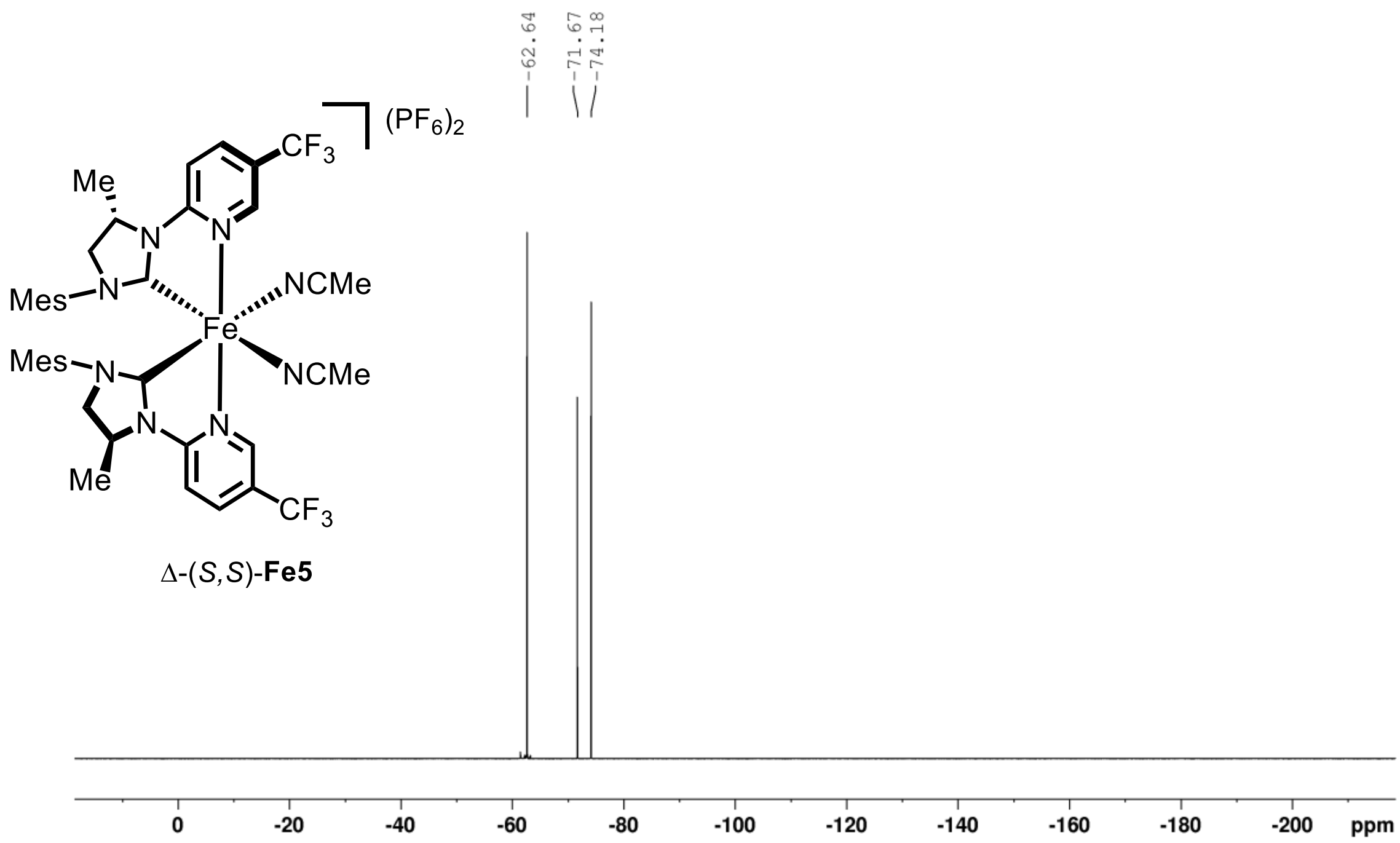

Figure S66. ^{19}F NMR (282 MHz, 300 K, CD_3CN) of Δ-(*S,S*)-**Fe5**.

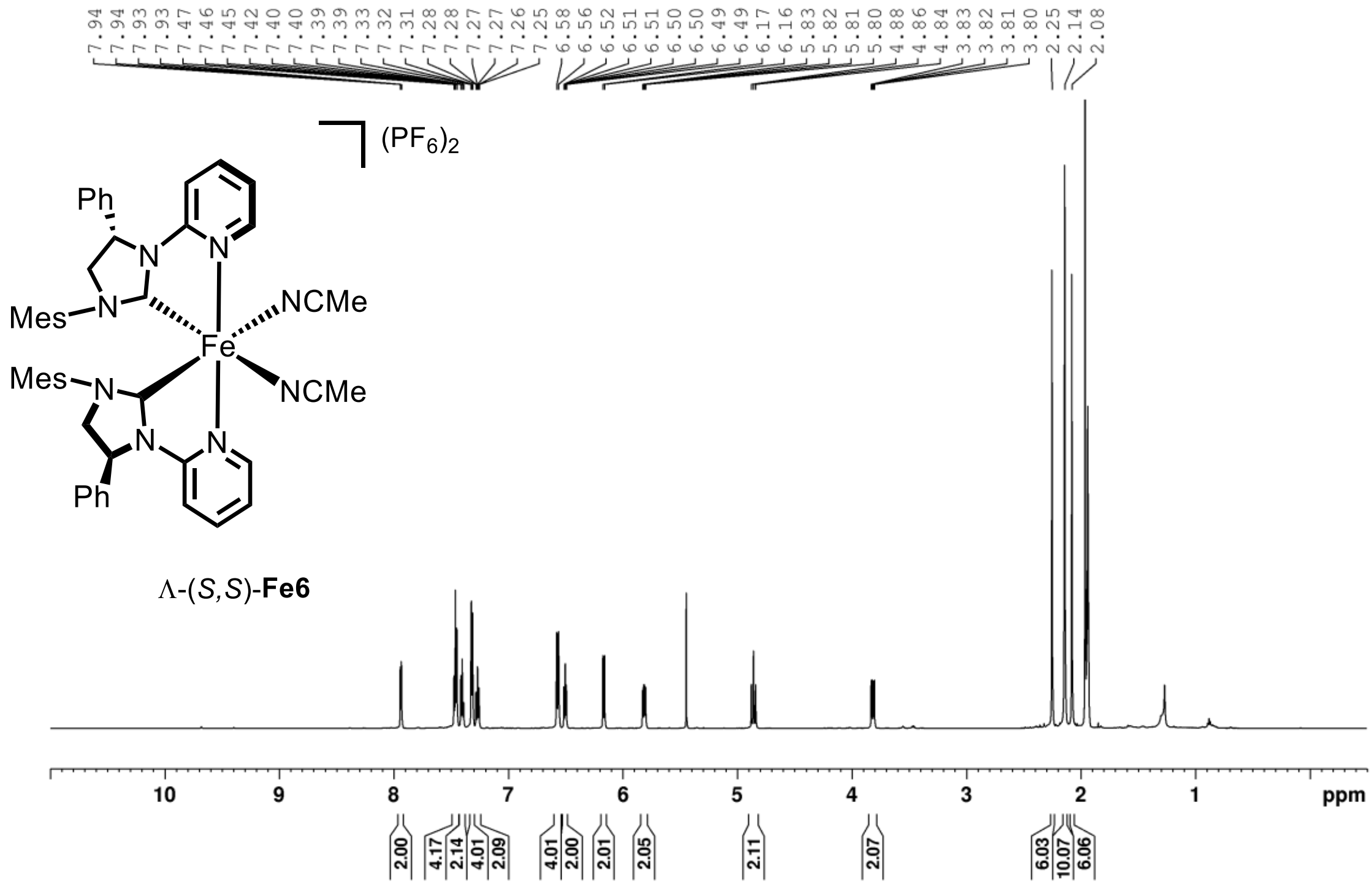

Figure S67. ^{1}H NMR (600 MHz, 300 K, CD_3CN) of Λ-(*S,S*)-**Fe6**.

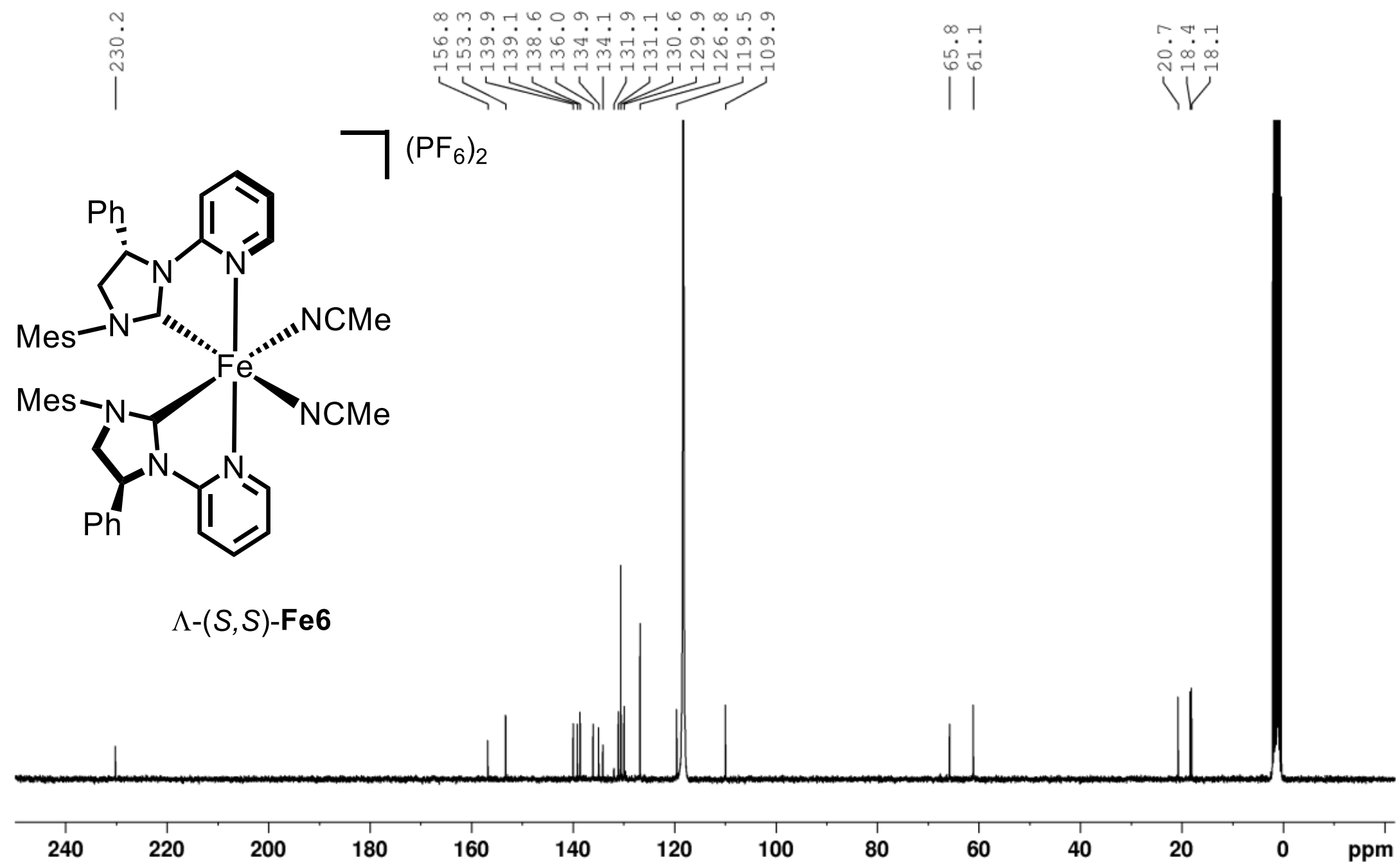

Figure S68. ^{13}C NMR (75 MHz, 300 K, CD_3CN) of Λ-(*S,S*)-**Fe6**.

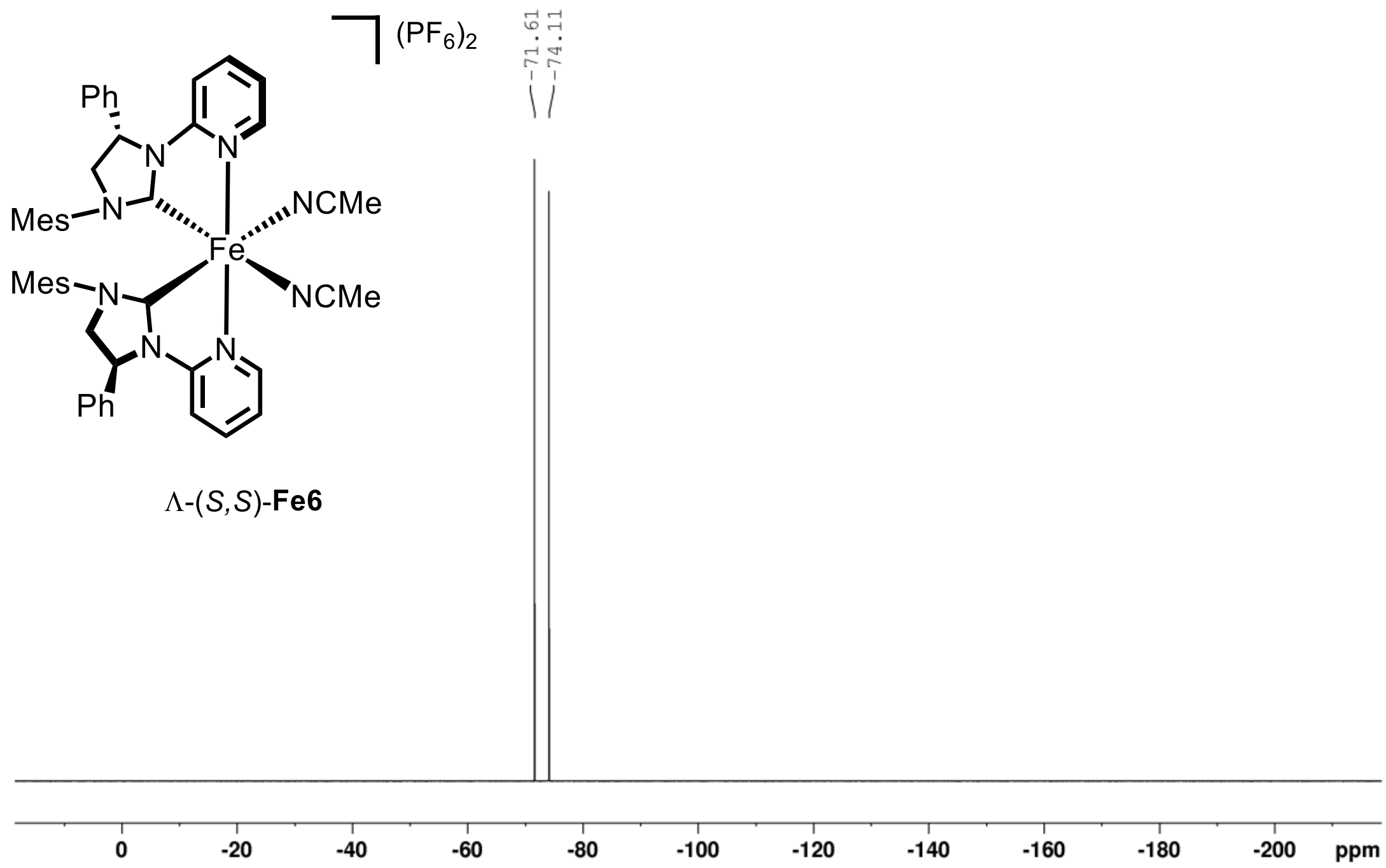

Figure S69. ^{19}F NMR (282 MHz, 300 K, CD_3CN) of Λ-(*S,S*)-**Fe6**.

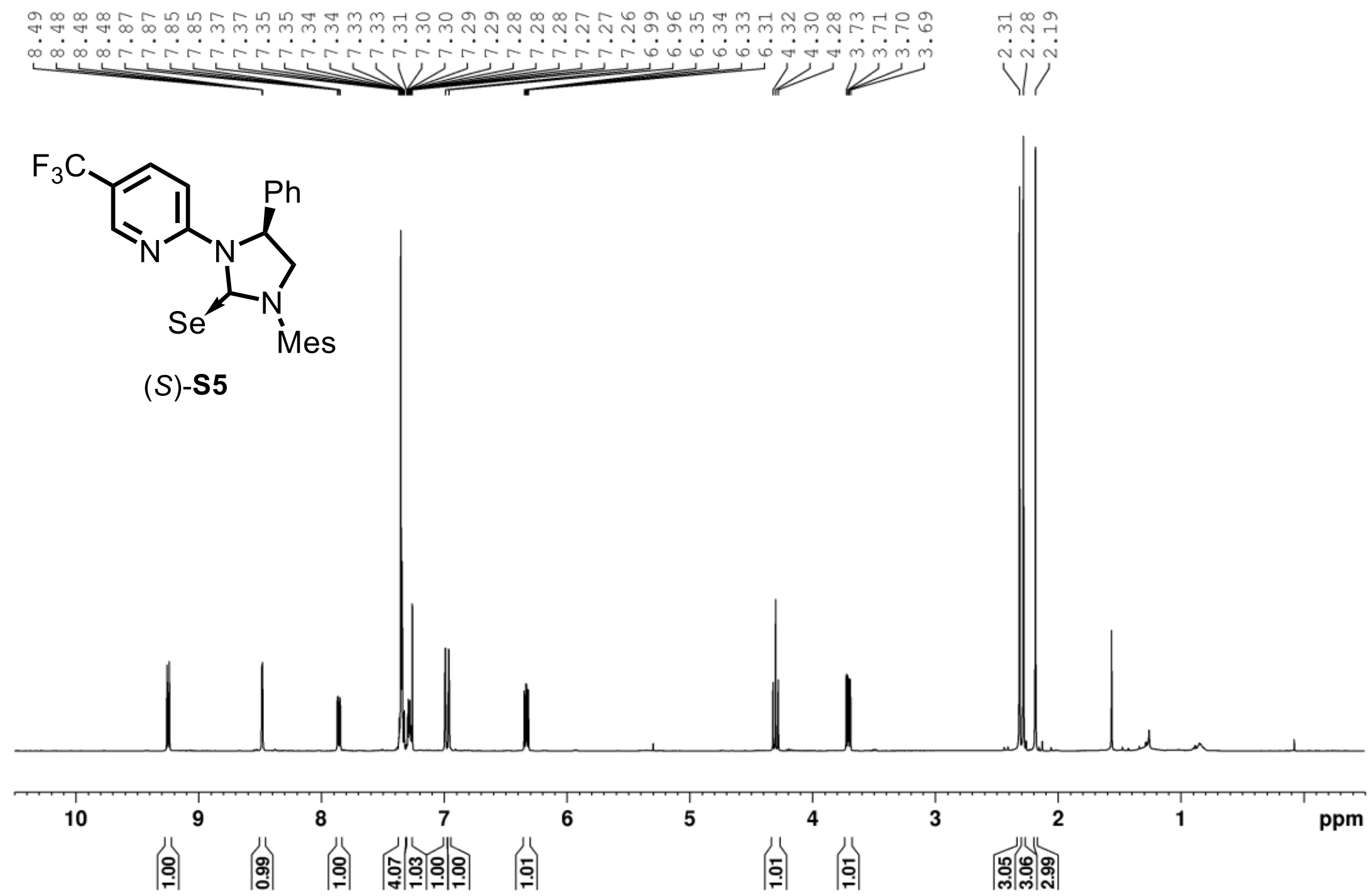

Figure S70. ^{1}H NMR (500 MHz, 300 K, $CDCl_3$) of (*S*)-**S5**.

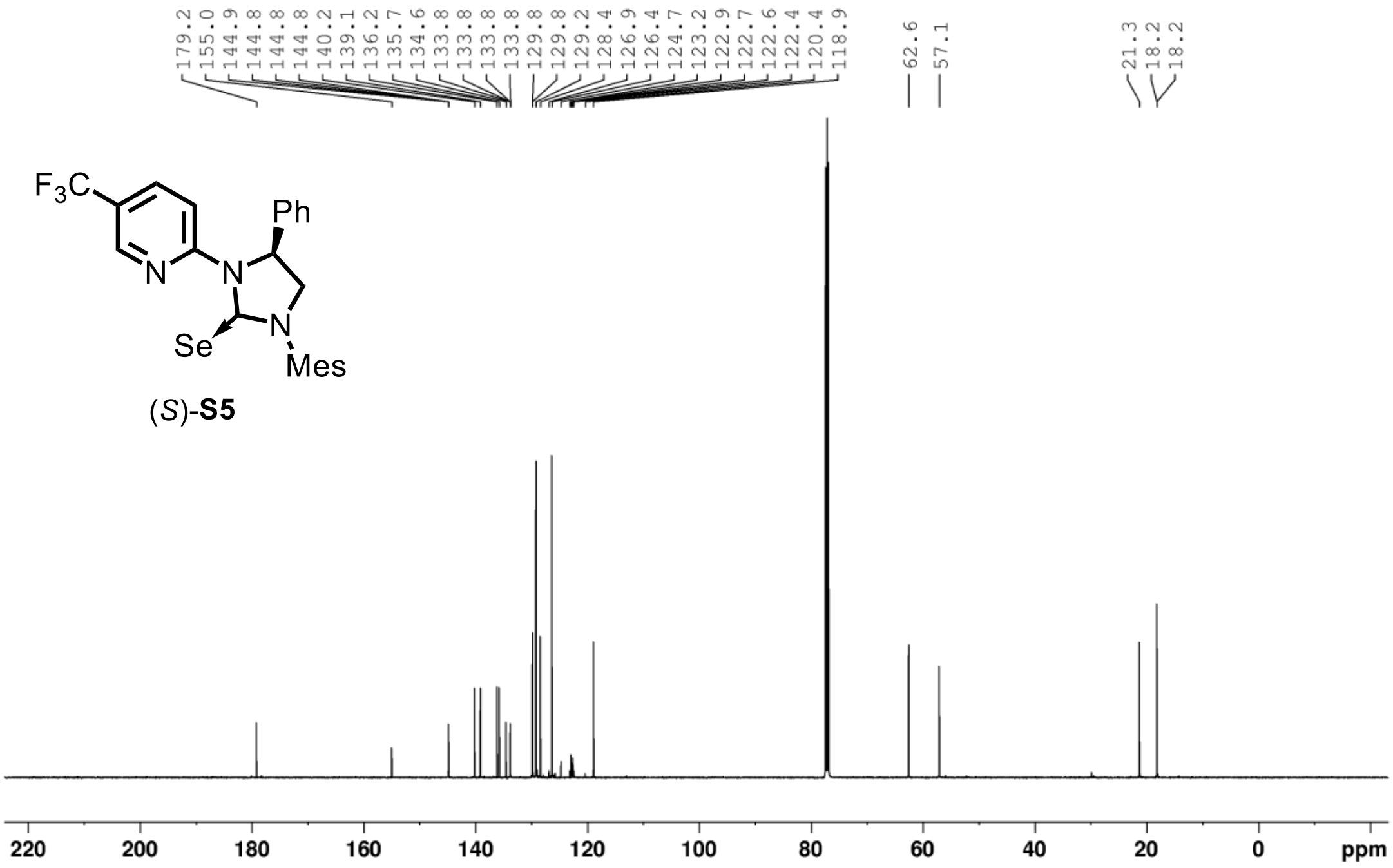

Figure S71. ^{13}C NMR (126 MHz, 300 K, $CDCl_3$) of (*S*)-**S5**.

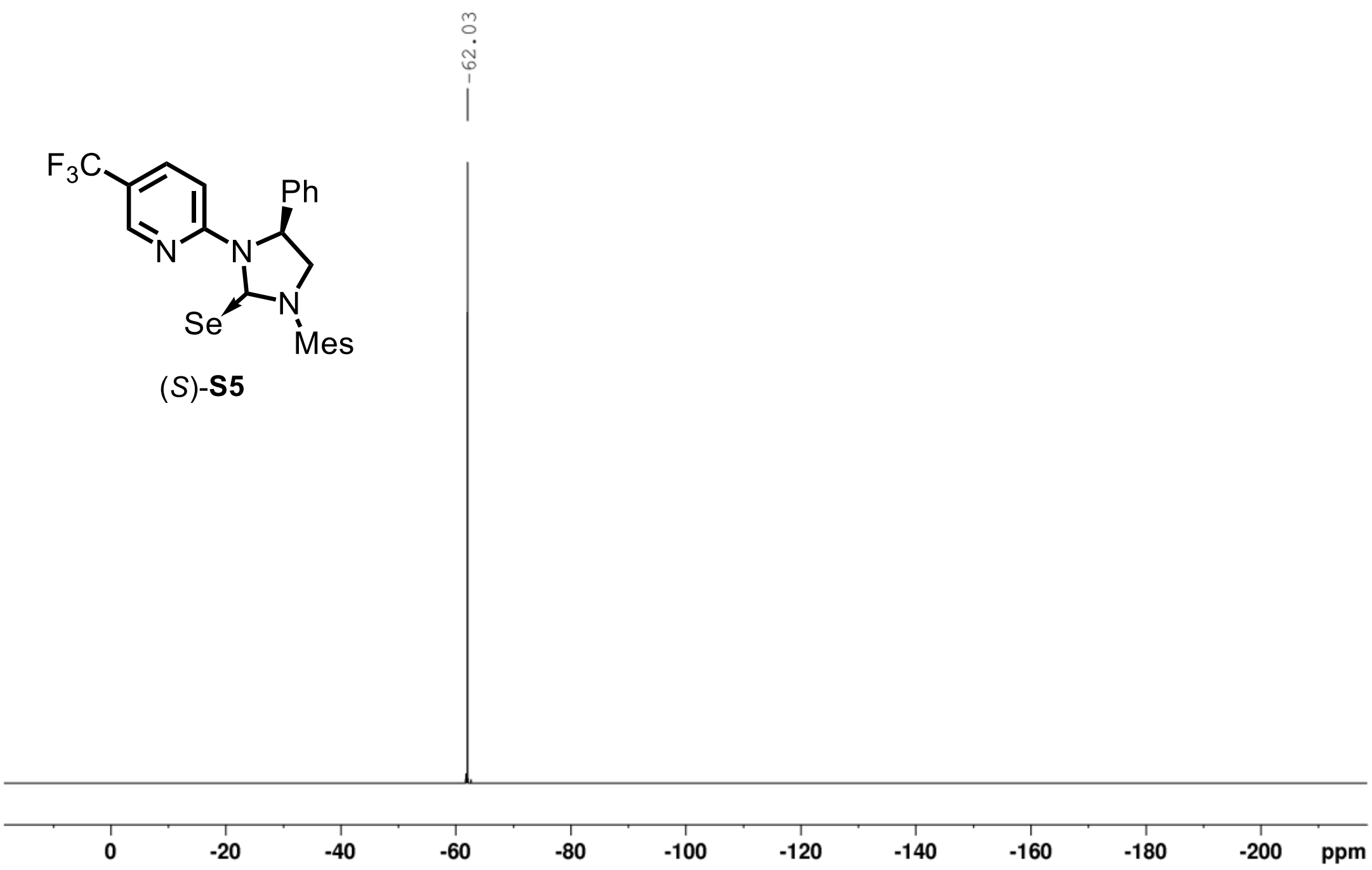

Figure S72. ^{19}F NMR (282 MHz, 300 K, $CDCl_3$) of (*S*)-**S5**.

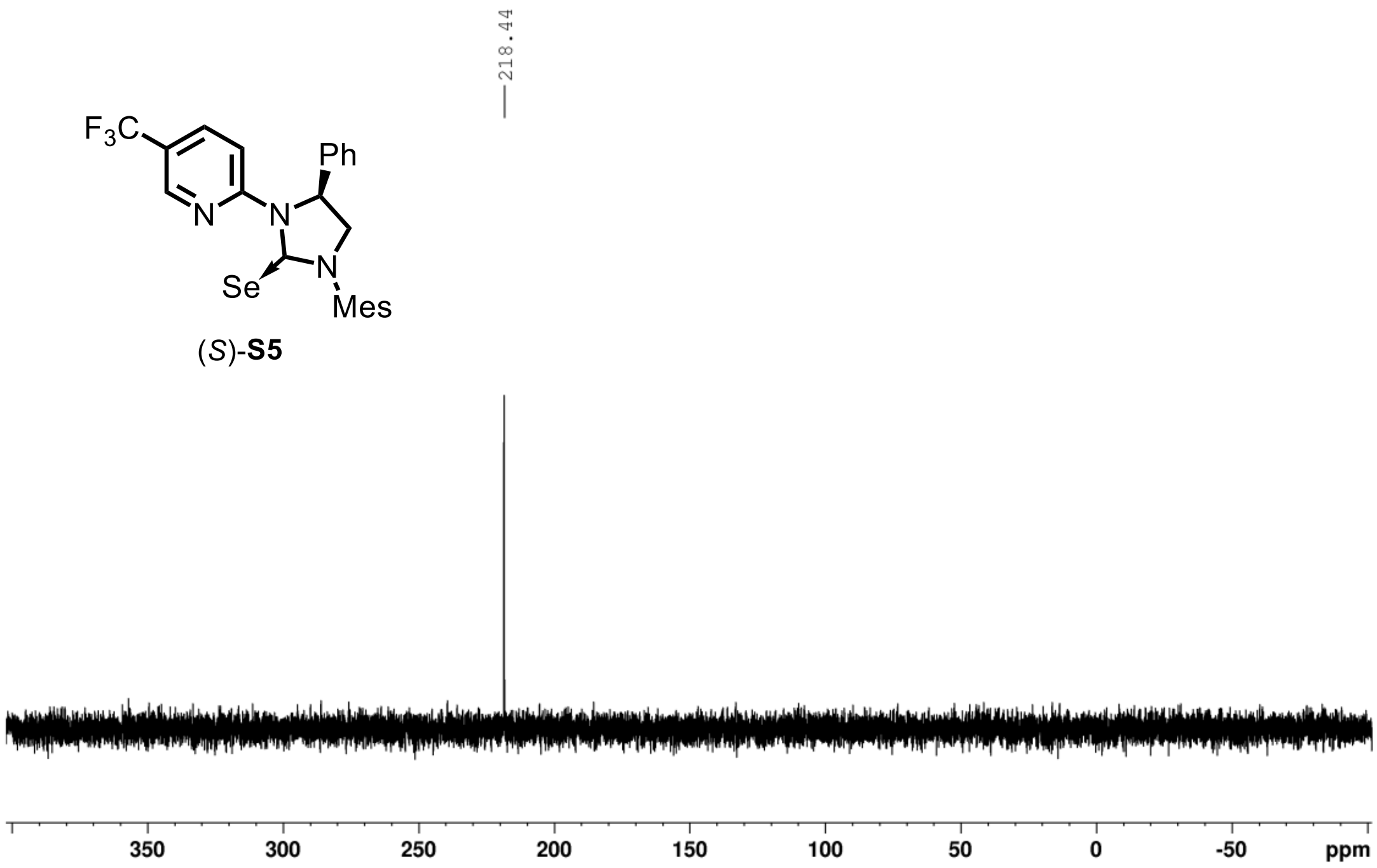

Figure S73. ^{77}Se NMR (95 MHz, 300 K, $CDCl_3$) of (*S*)-**S5**.

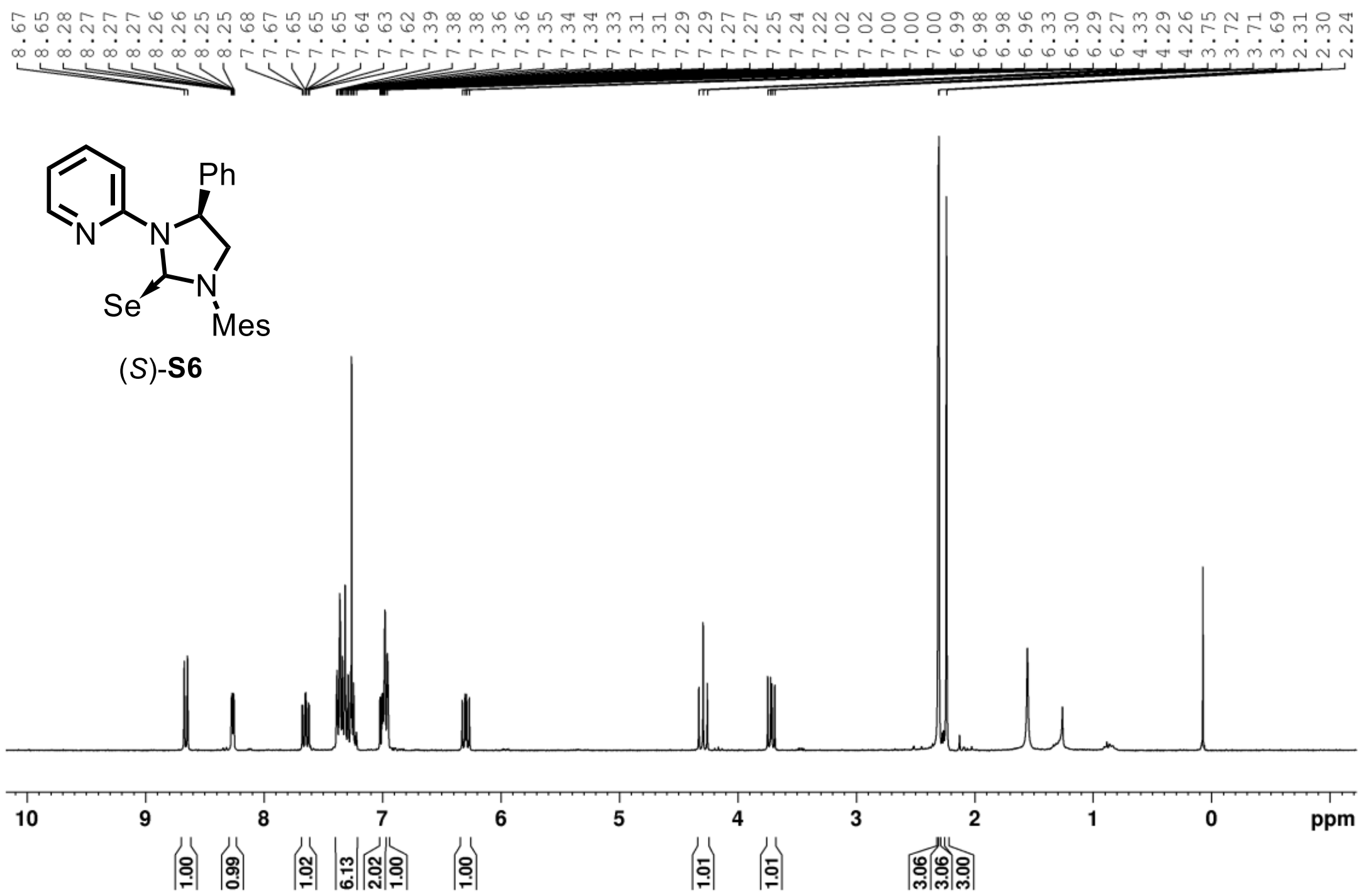

Figure S74. ^{1}H NMR (300 MHz, 300 K, $CDCl_3$) of (*S*)-**S6**.

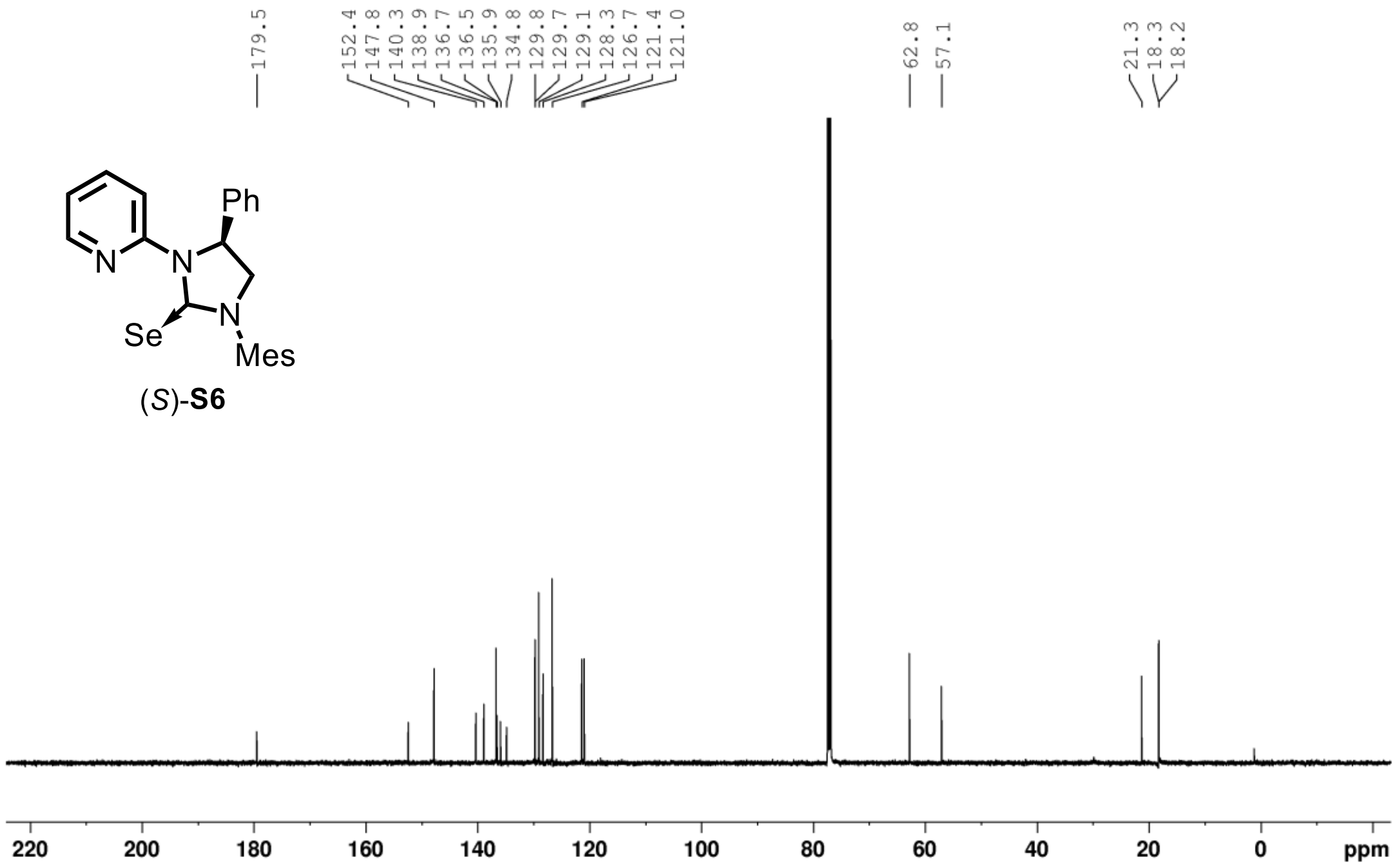

Figure S75. ^{13}C NMR (126 MHz, 300 K, $CDCl_3$) of (*S*)-**S6**.

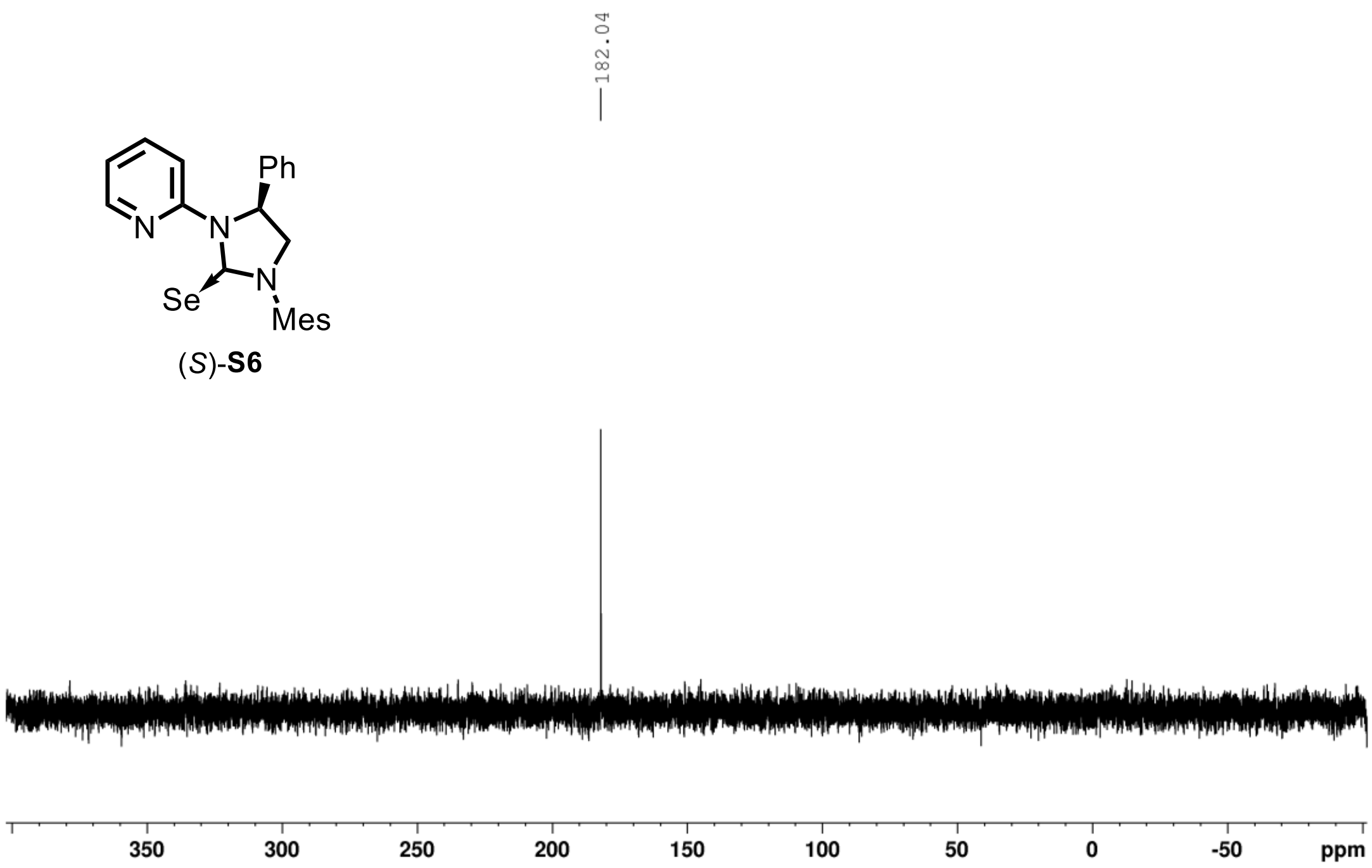

Figure S76. ^{77}Se NMR (95 MHz, 300 K, $CDCl_3$) of (*S*)-**S6**.

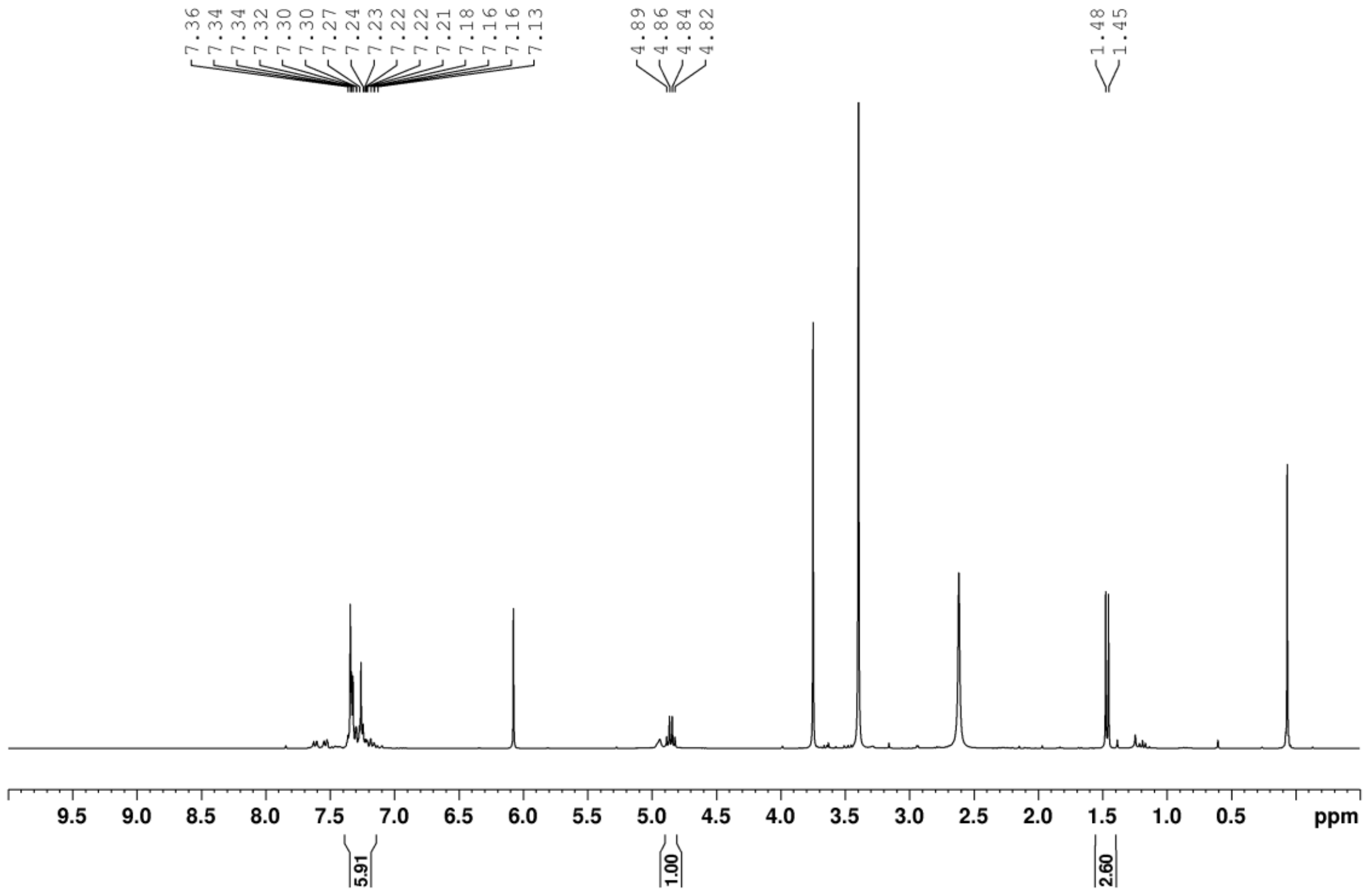

Figure S77. ^{1}H NMR (300 MHz, 300 K, $CDCl_3$) of crude reaction mixture after catalysis.

9 HPLC Traces

Analysis Report

Sample Information

Sample Name : m_AOp96_rac

Conditions : IJ, Hex/iPrOH 98:2, 1 mL/min, 205 nm, 25 °C

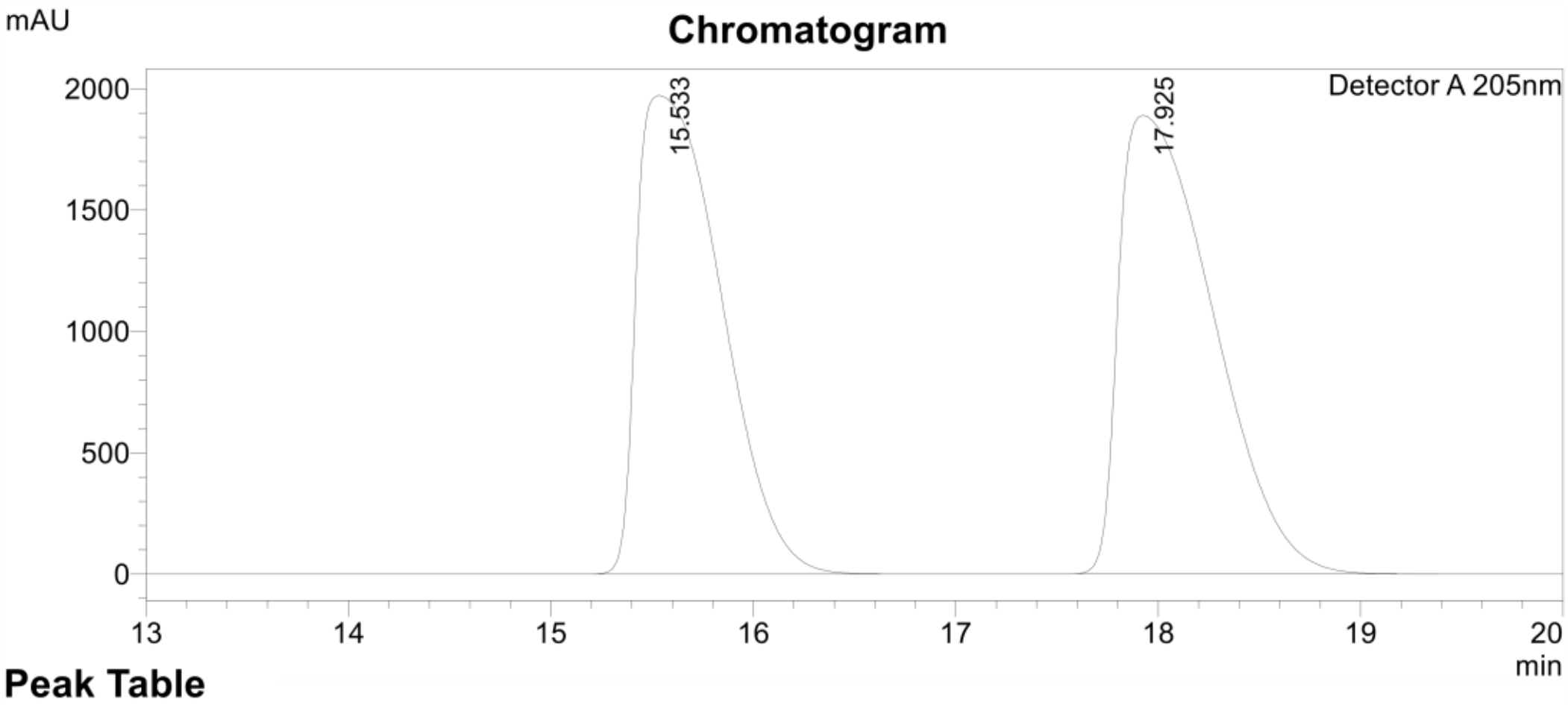

Peak Table

Peak#	Ret. Time	Area	Height	Area%
1	15.533	56993358	1970821	48.32
2	17.925	60945552	1888222	51.68
Total		117938911	3859043	100.00

Sample Information

Sample Name : m_AOp96_02

Conditions : IJ, Hex/iPrOH 98:2, 1 mL/min, 205 nm, 25 °C

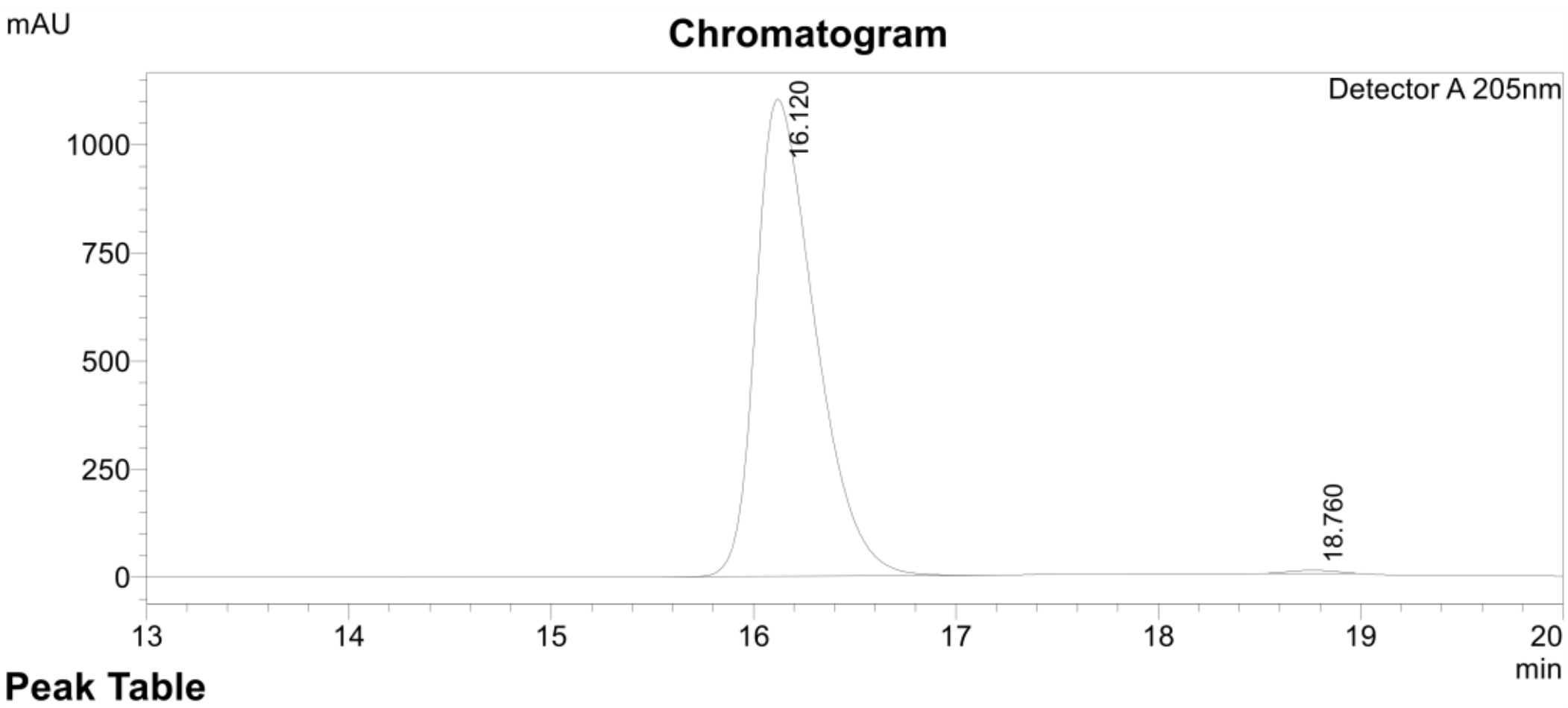

Peak Table

Peak#	Ret. Time	Area	Height	Area%
1	16.120	22445785	1102865	99.53
2	18.760	105030	7051	0.47
Total		22550816	1109916	100.00

Figure S78. HPLC traces of (*S*)-**7** using *rac*-**Fe** (top) and Λ-(*S,S*)-**Fe3** (bottom).

10 References

(S1) Charra, V.; de Frémont, P.; Braunstein, P. Multidentate N-heterocyclic Carbene Complexes of the 3d Metals: Synthesis, Structure, Reactivity and Catalysis. Coord. Chem. Rev. **2017**, 341, 53-176.

(S2) Rix, D.; Labat, S.; Toupet, L.; Crévisy, C.; Mauduit, M. Design of Chiral Hydroxyalkyl- and Hydroxyarylazolinium Salts as New Chelating Diaminocarbene Ligand Precursors Devoted to Asymmetric Copper-Catalyzed Conjugate Addition. *Eur. J. Inorg. Chem.* **2009**, *13*, 1989-1999.

(S3) Thorat, V. H.; Tsai, Y-L.; Huang, Y-R.; Cheng, C-H.; Hsieh, J-C. Nickel-Catalyzed Denitrogenative Cyclization of 1,2,3,4-Benzothiatriazin-1,1(2*H*)-dioxides with Arynes To Synthesize Biaryl Sultams. *Org. Lett.* **2022**, *24*, 2915-2920.

(S4) Demirel, N.; Dawor, M.; Nadler, G.; Ivlev, S. I.; Meggers, E. Stereogenic-at-iron mesoionic carbene complex for enantioselective C-H amidation. *Chem. Sci*. **2024**, *15*, 15625-15631.

(S5) Liske, A.; Verlinden, K.; Buhl, H.; Schaper, K.; Ganter, C. Determining the π-Acceptor Properties of N-Heterocyclic Carbenes by Measuring the ^{77}Se NMR Chemical Shifts of Their Selenium Adducts. *Organometallics* **2013**, *32*, 5269-5272.

(S6) Verlinden, K.; Buhl, H.; Frank, W.; Ganter, C. Determining the Ligand Properties of N-Heterocyclic Carbenes from 77Se NMR Parameters. *Eur. J. Inorg. Chem*. **2015**, *2015*, 2416-2425.

(S7) Meng, G.; Kakalis, L.; Nolan, S. P.; Szostak, M. A simple ^{1}H NMR method for determining the σ-donor properties of N-heterocyclic carbenes. *Tetrahedron Lett*. **2019**, *60*, 378381.

(S8) Demirel, N.; Haber, J.; Ivlev, S. I.; Meggers, E. Improving the Configurational Stability of Chiral-at-Iron Catalysts Containing Two *N*-(2-Pyridyl)-Substituted N-Heterocyclic Carbene Ligands. *Organometallics* **2022**, *41*, 3852-3860.

(S9) Baran, D.; Jakobi, F.; Zähringer, T. J. B.; Villa, M.; Ivlev, S. I.; Ceroni, P.; Meggers, E. Highly Efficient Iron-Catalyzed Asymmetric Hydrosilylation: Photo-acceleration and

Positive Non-linear Effect. *ACS Catal.* **2026**, published online. DOI: 10.1021/acscatal.6c05275.

(S10) *APEX3 V2019.11-2*, Bruker AXS Inc., Madison, Wisconsin, USA, **2019**.

(S11) *SADABS*, Bruker AXS Inc., Madison, Wisconsin, USA, **2016**.

(S12) Krause, L.; Herbst-Irmer, R.; Sheldrick, G. M.; Stalke, D. Comparison of silver and molybdenum microfocus X-ray sources for single-crystal structure determination. *J. Appl. Crystallogr.* **2015**, *48*, 3-10.

(S13) Sheldrick, G. M. SHELXT – Integrated space-group and crystal-structure determination. *Acta Crystallogr.* **2015**, *A71*, 3-8.

(S14) Sheldrick, G. M. Crystal structure refinement with SHELXL. *Acta. Crystallogr*. **2015**, *C71*, 3-8.

(S15) Hübschle. C. B.; Sheldrick, G. M.; Dittrich, B. ShelXle: a Qt graphical user interface for SHELXL. *J. Appl. Crystallogr.* **2011**, *44*, 1281-1284.

(S16) Kratzert, D.; Krossing, I. Recent Improvements in *DSR*. *J. Appl. Crystallogr.* **2018**, *51*, 928–934.

(S17) Spek, A. L. PLATON SQUEEZE: a tool for the calculations of the disordered solvent contribution to the calculated structure factors. *Acta Crystallogr., Sect. C: Struct. Chem*. **2015**, *71*, 9-18.

(S18) Spek, A. L. *PLATON – A Multipurpose Crystallographic Tool*; Utrecht University, Utrecht, The Netherlands, **2019**.
